# Supplementary material for: Particle-resolved topological defects of smectic colloidal liquid crystals in extreme confinement
Source: Nat Commun. 2021 Jan 27;12:623. doi: 10.1038/s41467-020-20842-5 (PMC7840983; doi:10.1038/s41467-020-20842-5)
Supplement: Supplementary file 1 — Supplementary Information [file 41467_2020_20842_MOESM1_ESM.pdf]

Particle-resolved topological defects of smectic colloidal liquid crystals in extreme confinement

René Wittmann,[1] Louis B. G. Cortes,[2,3] Hartmut Löwen,[1] and Dirk G. A. L. Aarts[2]
[1)] *Institut für Theoretische Physik II: Weiche Materie, Heinrich-Heine-Universität Düsseldorf, D-40225 Düsseldorf, Germany*
[2)] *Department of Chemistry, Physical and Theoretical Chemistry Laboratory, University of Oxford, South Parks Road, Oxford OX1 3QZ, United Kingdom*
[3)] *School of Applied and Engineering Physics, Cornell University, Ithaca, NY 14853, USA*

Supplementary Note 1. Details on the experimental and theoretical methods

Sedimentation of silica rods. To experimentally create confined quasi-two-dimensional smectic structures, we take advantage of the phase stacking of silica rods in sedimentation equilibrium[1,2]. The bare dimensions of the rods are measured directly from scanning electron microscopy images. The mean length is $W = 5.3\,\mu$m with a standard deviation $\sigma_W = 0.5\,\mu$m. The rods are dispersed into a 1 mM NaCl water solution to ensure stability through double layer repulsion, whose range is characterized by the Debye length $\kappa^{-1} = 0.01\,\mu$m. Moreover, the phase behavior of our charged rods can be mapped onto the phase behavior of hard rods[3–6] by introducing the effective rod length $W_{\text{eff}} = 5.4\,\mu$m, diameter $D_{\text{eff}} = 470$nm and aspect ratio $p_{\text{eff}} = 10.6$ to account for the Debye screening.

An illustration of the experimental cell can be found in Fig. 1a of the main manuscript. The confining cavities in the shape of hollow cylinders are molded on the bottom coverslip using home made Polydimethylsiloxane (PDMS) stamps and Norland Optical Adhesive[1]. In practice, several chambers are fitted in a single cell. After preparation, the rod solution is left in the tube to sediment for at least 12 hours. During sedimentation, the concentration of particles gradually increases along the direction of the gravity field leading to the successive formation of isotropic, nematic and smectic phases. After a few hours, sedimentation diffusion equilibrium is reached and the three phases coexist in the cavities.

The smectic structures are observed by means of bright-field microscopy in direct vicinity of the bottom wall. We use a 1.42 numerical aperture apochromat oil immersion objective mounted on an Olympus IX73 microscope and coupled to a Ximea CMOS xiQ camera, which allows an optical resolution comparable to the rod diameter. Due to degenerate planar anchoring at the bottom wall, the system can be considered a quasi-two-dimensional fluid in annular confinement. We choose the total amount of rods such that there is no crystalline state and the rods in direct contact with the bottom wall exhibit smectic order. The effective (three-dimensional) volume fraction $\phi_{\text{eff}} \approx 45-50\%$ at the bottom of the cell is measured indirectly by comparing the height of the smectic region to the sedimentation equilibrium of our rod system in bulk[2] and assuming that the rods behave like hard spherocylinders[7]. For this setup, we measure $\lambda_0 \approx 1.3\,W_{\text{eff}}$ for the effective two-dimensional bulk layer spacing at the bottom wall.

Density functional theory (DFT). Classical DFT[8] is a powerful and versatile tool to access the structure of inhomogeneous fluids on the particle scale. Here, we study by free minimization of a DFT in two dimensions hard discorectangles with rectangular length L, total length $W = L + D$ and unit circular diameter D (see Fig. 1c of the main manuscript). If not denoted otherwise, we use $L = 10D$, so that the aspect ratio $p = L/D = 10$ well reflects the experimental parameters. The energy unit is set by $\beta^{-1} := k_{\text{B}}T$, where k_{B} and T are Boltzmann's constant and the temperature, respectively. Note that the structural transition of perfectly hard particles are completely driven by entropy, since β only appears as a trivial scaling factor. The key quantity in our theory is the one-body density profile $\rho(\mathbf{r}, \phi)$, providing the probability to find a particle with the center-of-mass position $\mathbf{r}$ and its symmetry axis oriented along an angle ϕ.

The general form of the density functional reads[8]

$$\Omega[\rho] = \mathcal{F}[\rho] + \int \mathrm{d}\mathbf{r} \int_0^{2\pi} \frac{\mathrm{d}\phi}{2\pi}\, \rho(\mathbf{r}, \phi)(V_{\text{ext}}(\mathbf{r}, \phi) - \mu)\,, \quad (1)$$

where the external potential $V_{\text{ext}}(\mathbf{r}, \phi)$ imposes the annular confinement through a hard-wall potential and μ denotes the chemical potential. The intrinsic Helmholtz free energy functional

$$\beta\mathcal{F}[\rho] = \int \mathrm{d}\mathbf{r} \int_0^{2\pi} \frac{\mathrm{d}\phi}{2\pi}\, \rho(\mathbf{r}, \phi) \left(\ln(\rho(\mathbf{r}, \phi)\Lambda^2) - 1\right) + \beta\mathcal{F}_{\text{ex}}[\rho] \quad (2)$$

consists of an ideal-gas term (Λ denotes the irrelevant thermal wave length) and the excess free energy functional $\mathcal{F}_{\text{ex}}[\rho]$, which describes the interactions and correlations between the individual particles. Here, the latter is constructed as an extension of fundamental measure theory to account for anisotropic particle shapes. These geometrical functionals derived from first principles are exact in the low-density limit and have proven very reliable for highly packed systems.

The employed excess functional[9]

$$\beta\mathcal{F}_{\text{ex}}[\rho] = \int \mathrm{d}\mathbf{r} \left(-n_0 \ln(1 - n_2) + \frac{N}{2(1 - n_2)}\right)\,, \quad (3)$$

of the two dimensional fundamental mixed measure theory is constructed as a function of weighted densities

$$n_\nu(\mathbf{r}) = \int \mathrm{d}\mathbf{r}_1 \int_0^{2\pi} \frac{\mathrm{d}\phi}{2\pi}\, \rho(\mathbf{r}_1,\phi)\, \omega^{(\nu)}(\mathbf{r}-\mathbf{r}_1,\phi)\,. \tag{4}$$

These are calculated by convolution of the density and the one-body geometrical measures $\omega^{(\nu)}$, representing a local area ($\nu=2$), circumference ($\nu=1$) and boundary curvature ($\nu=0$) of the particles. The mixed weighted density $N(\mathbf{r})$ generally depends on the geometry of two bodies. To efficiently study long rods in large systems, we use the approximate representation

$$N \approx \frac{2+a}{6\pi} n_1 n_1 + \frac{a-4}{6\pi} n_{1,\alpha} n_{1,\alpha} + \frac{2-2a}{6\pi} n_{1,\alpha\beta} n_{1,\beta\alpha}\,, \tag{5}$$

found by an expansion introducing vectorial $n_{1,\alpha}$ and tensor-valued weighted densities $n_{1,\alpha\beta}$ up to rank-two, where we use the convention of summation over repeated indices $\alpha,\beta \in \{1,2\}$. The correction parameter $a=4$ allows to qualitatively describe the smectic phase over the full range of aspect ratios[9,10].

Generally, the density $\rho(\mathbf{r},\phi)$ of a (meta-) stable state is found by iteratively solving the extremal condition $\delta\Omega[\rho]/\delta\rho = 0$. In practice, an individual density profile $\rho_i(\mathbf{r},\phi_i)$ is considered for each discrete orientation ϕ_i. After each iteration step j, the current densities $\rho_i^{(j)}$ are updated according to a Picard iteration scheme[8,11]. This involves a formal solution of

$$\frac{\delta\Omega[\rho_i^{(j)}]}{\delta\rho_i^{(j)}} = 0 \tag{6}$$

for a new $\tilde{\rho}_i^{(j)}$ and calculating $\rho_i^{(j+1)} = (1-\gamma)\rho_i^{(j)} + \gamma\tilde{\rho}_i^{(j)}$ with a dynamical mixing parameter γ, initially set to $\gamma = 0.08$. If the value of the local packing fraction $n_2(\mathbf{r})$ (see Supplementary Equation (4)) exceeds one for any coordinate $\mathbf{r}$, the step is rejected and γ is decreased by a factor of 0.2. After each 200 steps, the program tries to gradually increase γ by factors of 2 to speed up the iteration. The average area fraction

$$\eta = N\,\frac{4LD + D^2\pi}{4\pi R_{\mathrm{out}}^2(1-b^2)} \tag{7}$$

is kept fixed throughout the iteration by adapting μ in each step. Hence, the stable state corresponds to a global minimum of the free energy $\mathcal{F}[\rho]$.

The minimization is initialized by creating some appropriate random density profiles with different symmetries. If the value of $n_2(\mathbf{r})$ exceeds one for an initial guess, the density profiles are renormalized to obtain a valid profile. In the course of the minimization, the packing fraction η is then gradually increased back to its input value. We iterate until the free energy differences between different structures, which typically are of the order 10^{-5} in units of the thermal energy, can be sufficiently resolved. This is usually the case when the free energy changes by less than $5\cdot 10^{-7}$ in the last 1000 iteration steps. We thus assume that the numerical error in the free energy is of the order $5\cdot 10^{-6}$. Since the true free energy is always smaller for each structure, most of this error occurs as a systematic shift, irrelevant when comparing relative free energies as we do here. In some cases, as for $R_{\mathrm{out}} = 6.3L$ and $b=0.29$ or $b=0.3$ in Fig. 6 of the main manuscript, there are competing structures with free energy differences of 10^{-6} and smaller, making it difficult to unambiguously determine the global minimum. However, the systematic errors due to the approximations in the density functional can be larger.

After equilibration of multiple structures, we compare the values of the free energy $\mathcal{F}[\rho]$ to distinguish between local and global minima and quantify the likelihood to observe a particular state in a corresponding experiment. Calculations are performed on a quadratic spatial grid with a high enough resolution $\Delta x = \Delta y = 0.2$ and $N_\phi = 96$ orientational angles (for $p=10$). Some structures were created with a smaller resolution and then further equilibrated for the given parameters.

The obtained density profiles $\rho(\mathbf{r},\phi)$ are usually graphically represented by a color scheme on a range from zero to three denoting the dimensionless orientationally averaged density

$$\bar{\rho}(\mathbf{r}) := \left(LD + \frac{D^2\pi}{4}\right)\int_0^{2\pi} \frac{\mathrm{d}\phi}{2\pi}\,\rho(\mathbf{r},\phi)\,. \tag{8}$$

Here we also display lines of length given by the orientational order parameter and orientation indicating the local orientational director field. These quantities can be extracted from the local order tensor

$$Q(\mathbf{r},\phi) = \frac{2\,\rho(\mathbf{r},\phi)}{\int_0^{2\pi}\mathrm{d}\phi\,\rho(\mathbf{r},\phi)} \begin{pmatrix} \cos^2\phi - \frac{1}{2} & \cos\phi\sin\phi \\ \cos\phi\sin\phi & \sin^2\phi - \frac{1}{2} \end{pmatrix} \tag{9}$$

as its largest Eigenvalue and the corresponding Eigenvector, respectively. An alternative graphical representation, used in Fig. 2 of the main manuscript, depicts the local packing fraction $n_2(\mathbf{r})$. As this quantity corresponds to the density weighted with the particle area, it directly illustrates the locations of the particles, while ranging between zero and one. Since the behavior is fluid-like within smectic layers, the local packing fraction is generally smeared-out, while the silhouettes of individual particles in the packed regions close to the walls are directly visualized.

Our benchmark calculation in bulk using the described DFT approximation locates the nematic–smectic transition around $\eta \approx 0.62$. As this two-dimensional area fraction is not directly comparable to the experimentally accesible volume fraction ϕ, our theoretical calculations in the confined system are generally carried out for $\eta = 0.65$. With this choice of the smectic density, the relative deviation from the bulk nematic–smectic transition density in experiment and theory are similar. At this area fraction, the optimal bulk layer spacing is $\lambda_0 = 12.56D = 1.142W$.

To determine the inclusion size ratio b_{t} at which a structural transition occurs in annular confinement, we

select two values b_1 and b_2, usually differing by $b_2 - b_1 = 0.01$, and check whether the sign of the free-energy difference between two states of interest is different. If so, we determine $b_1 < b_\mathrm{t} < b_2$ as the point where the linearly interpolated free energies are equal.

Supplementary Note 2. Classification and identification of the different smectic states

In general, we use the following criteria to distinguish the different smectic states through occuring defects and the arrangement of smectic layers. This is most easily done by observing the number and orientation of line disclinations and identifying connected layers, which span between two opposite sites of the outer boundary. Usually, there is a typical number of edge dislocations associated with Shubnikov structures, but a certain number of edge dislocations can also occur in other states. Microscopic details are not relevant for this first classifications.

Laminar (or bridge) state $\mathcal{L}$ (or $\mathcal{B}$): there is at least one connected layer at each side of the inclusion. Usually, this is equivalent to observing two anti-radial disclination lines close to the outer boundary. Domain state $\mathcal{D}$: there are exactly three radially oriented disclination lines, separated by 120 degrees; the layers from different domains meet at an angle of 90 degrees. Shubnikov state $\mathcal{S}$: there is neither a connected layer nor any disclination line; all layers span between the inner and outer boundary. Laminar–domain composite state $\mathcal{C}^{\mathcal{LD}}$: there are one or two radial disclination lines and at least one connected layer (or anti-radial disclination line) at only one side of the inclusion. Laminar–Shubnikov composite state $\mathcal{C}^{\mathcal{LS}}$: there are no radial disclination lines and at least one connected layer (or anti-radial disclination line) at only one side of the inclusion. Domain–Shubnikov composite state $\mathcal{C}^{\mathcal{DS}}$: there are one or two radial disclination lines and no connected layer.

For each intact experimental chamber, we identify the dominant state by visual inspection of our particle-resolved images according to the above criteria. In the smallest experimental chambers considered it is not always possible to clearly associate an observed structure with one of these states. Strongly deformed theoretical structures are not taken into further consideration, since it is clear from their large free energy that they are irrelevant in the search for the most stable mesoscopic state.

Supplementary Note 3. Phenomenological model for defect energies

The existence of the laminar–Shubnikov transition in Fig. 3 of the main manuscript can be understood in the light of a phenomenological model. Making some minimal assumptions, we estimate the energy penalty resulting from the characteristic defects in each state. Let us first denote the energy of a disclination line with unit length by δ and of each edge discolation by u_ed. We then assume that the two anti-radial disclination lines in the laminar state have the length R_out and that each radial domain boundary is a straight line of length $R_\mathrm{out} - R_\mathrm{in}$. The number

$$N_\mathrm{out} - N_\mathrm{in} \approx \frac{2\pi R_\mathrm{out} - 2\pi R_\mathrm{in}}{\lambda_0} \tag{10}$$

of edge dislocations in the Shubnikov state is assumed as the difference of the outer and inner wall perimeters, divided by the bulk layer spacing λ_0. The resulting energies read

$$\begin{aligned} U_\mathcal{L} &= 2\,\delta\,R_\mathrm{out}\,, \\ U_\mathcal{D} &= 3\,\delta\,R_\mathrm{out}\,(1-b)\,, \\ U_\mathcal{S} &= \frac{2\pi}{\lambda_0} R_\mathrm{out}\,(1-b)\,u_\mathrm{ed} \end{aligned} \tag{11}$$

for the laminar, domain and Shubnikov state, respectively.

For all choices of the fit parameters δ and u_ed, the state diagram predicted by this simple model is independent of the size of the annulus, because all energies in Supplementary Equation (11) scale with R_out. Moreover, there is exactly *one* stable transition for increasing b, namely from a laminar state, at small inclusions, to either a domain or Shubnikov state at large inclusion. One of the two latter states is always metastable, since both $U_\mathcal{D}$ and $U_\mathcal{S}$ are proportional to $(1-b)$. These energies further decrease to zero for an infinitely thin annulus ($b \to 1$), while the energy $U_\mathcal{L}$ assumed for the laminar state remains constant when increasing b. Therefore, the laminar state always becomes metastable for large inclusion sizes. A stable laminar–domain transition may occur at $b = \frac{1}{3}$ if $U_\mathcal{D} < U_\mathcal{S}$, which means that $3\delta\lambda < 2\pi u_\mathrm{ed}$. In the opposite case, we expect a stable laminar–Shubnikov transition at $b = 1 - \frac{\lambda\delta}{\pi u_\mathrm{ed}} < \frac{1}{3}$, while the domain state is only metastable.

Although this phenomenological model does not capture all theoretical and experimental observations, it nicely reflects the competition between domain- and Shubnikov states, which possess the same director topology at the outer wall, and rationalizes the laminar–Shubnikov transition, which is one of our main results. Using as an input to the presented model that this transition can be roughly observed at $b_0 = 0.3$, we can determine the ratio

$$\frac{\lambda\delta}{u_\mathrm{ed}} = \pi\,(1-b_0) \approx 2.2 \tag{12}$$

of the two energy parameters entering in Supplementary Equation (11). The transition lines for these parameters are drawn into the state diagram in Fig. 3 of the main manuscript.

This model calculation provides a rough interpretation of some experimental findings, but has limited predictive

power, e.g., it does not feature any dependence of the location of the transitions on the total confinement size. What is neglected in this simple model are all types of elastic deformations, the interaction of line and point defects, preferences in the wall alignment, a locally adaptable smectic layer spacing and density differences within the smectic layers close to the walls or to defects. Altogether, these effects occur on the order of the correlation length, which is typically on the particle scale. This underlines the importance of a fully microscopic theoretical treatment to comprehensively characterize the emerging structures in extreme confinement, which is illustrated in Fig. 4 of the main manuscript.

Supplementary Note 4. Optimal microscopic arrangement of layers

Associating an observed structure with a particular smectic state does not account for the full microscopic information. In particular, different structures can mainly be distinguished by counting the explicit numbers of smectic layers. The optimal microscopic structure which is most stable for a given state itself depends on the competition between different driving forces, which we detail below.

Laminar state $\mathcal{L}_{N_{\mathrm{con}},N_{\mathrm{dis}}}$. In a given laminar state $\mathcal{L}_{N_{\mathrm{con}},N_{\mathrm{dis}}}$, there are precisely N_{con} connected layers spanning throughout the confinement and N_{dis} layers disconnected by the inclusion, summing up to a total number $N_{\mathrm{tot}} = N_{\mathrm{con}} + N_{\mathrm{dis}}$ of layers (where $N_{\mathrm{tot}} = N_{\mathrm{con}}$ in the bridge state), see Supplementary Fig. **1**. Depending on the geometry, there may further be two anti-radial disclination lines close to the boundary. We consider the shape of the disclination line and the number of layers in the separated domains as structural details of next order, since the theory automatically finds the optimal structure corresponding to $\mathcal{L}_{N_{\mathrm{con}},N_{\mathrm{dis}}}$ in a given confinement (compare the different structures with the same N_{con} and N_{dis} for different inclusion sizes in Supplementary Figs. **6** and **7**).

In general, the theory predicts only relatively small deviations of the laminar layer spacing from the bulk value λ_0. As a consequence, the total number N_{tot} of smectic layers in the parallel domain, fluctuates as a function of the inclusion size ratio b, as shown in Supplementary Fig. **1**, which also applies to the length and shape of the disclination lines (compare Supplementary Figs. **6** and **7**). The optimal microscopic laminar structure is then determined by the integer multiples of λ_0 which are closest to the respective dimensions of the confinement. For example, N_{dis} is usually bounded by the diameter $2R_{\mathrm{in}}/\lambda_0$ of the inclusion to avoid additional defects or strong deformations near the wall.

Shubnikov state $\mathcal{S}_{N_{\mathrm{in}},N_{\mathrm{out}}}$. the most significant structural property of a given Shubnikov state $\mathcal{S}_{N_{\mathrm{in}},N_{\mathrm{out}}}$

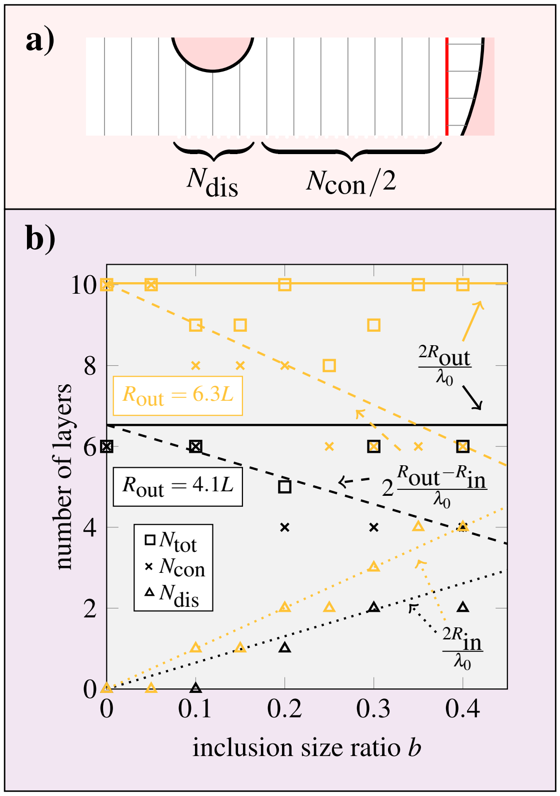

Supplementary Figure 1. Geometry dependence of the layers in the laminar state; **a)** schematic definition of connected/disconnected layer numbers $N_{\mathrm{con/dis}}$, in total $N_{\mathrm{tot}} = N_{\mathrm{dis}} + N_{\mathrm{con}}$ and **b)** nonmonotonic dependence of layer numbers (symbols at integer values) on the inclusion size ratio b for two outer radii R_{out} (colors as labeled). For comparison, the lines indicate the characteristic geometrical dimensions of the annulus divided by the bulk layer spacing λ_0 (as labeled).

is the number $N_{\mathrm{out}} - N_{\mathrm{in}}$ of point defects, which is is determined by the numbers N_{in} and N_{out} of layers in direct contact with the inner and outer wall, respectively. The exact radial (and relative) location of these defects are structural details of next order, which can be accounted for by creating and comparing a large number of different structures. As indicated in Supplementary Fig. **2**, we further consider a local layer spacing

$$\lambda_{\mathrm{in}} = \frac{\pi(2R_{\mathrm{in}} + D)}{N_{\mathrm{in}}} \tag{13}$$

at the inner and

$$\lambda_{\mathrm{out}} = \frac{\pi\sqrt{(2R_{\mathrm{out}} - D)^2 - L^2}}{N_{\mathrm{out}}} \tag{14}$$

at the outer wall, explicitly calculated here from the circumference of the line connecting the particle centers in direct vicinity of each wall. The effective layer spacing associated with the closest planar packing (assuming that all rods are aligned perfectly tangential to the wall) corresponds to the length of the arc through the particle

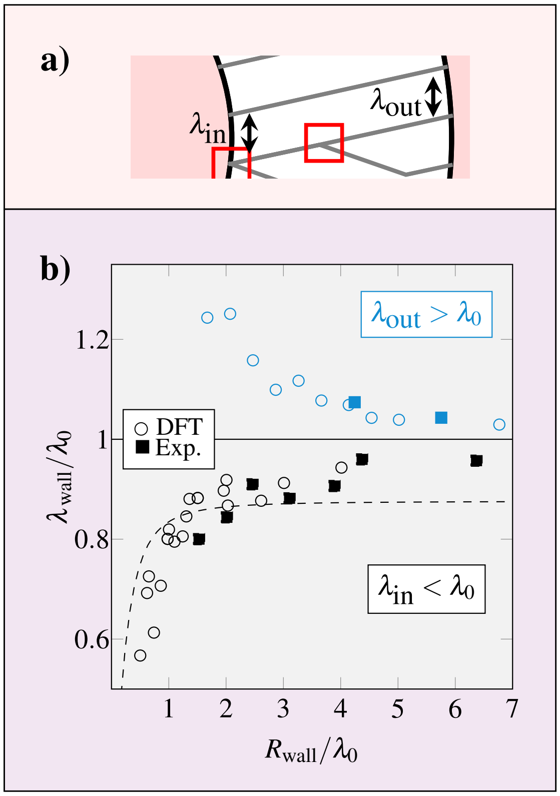

Supplementary Figure 2. Geometry dependence of the layers in the Shubnikov state; **a)** schematic definition of the local layer spacing λ_{in} and λ_{out} at the inner and outer wall and **b)** local layer spacing λ_{wall} as a function of the respective radius R_{wall} of the inner ('wall' = 'in') or outer ('wall' = 'out') wall, normalized by λ_0 to values smaller or larger than one, respectively, in different theoretical (circles) and experimental (squares) geometries. The dashed line represents a lower bound for rods packed at the inner wall with a perfectly planar alignment.

center between the two radial lines tangential to the particle and reads

$$\lambda_{\text{in}}^* = (2R_{\text{in}} + D)\left(\arctan\left(\frac{L}{2R_{\text{in}} + D}\right) + \arcsin\left(\frac{D}{\sqrt{(2R_{\text{in}} + D)^2 + L^2}}\right)\right) \quad (15)$$

This will serve as a reference value.

To quantify the variation in the local layer spacing, we determine λ_{wall} (the subscript 'wall' either stands for 'in' or 'out') for several theoretical and experimental structures (compare Supplementary Figs. **8**, **9** and **10**). According to Supplementary Fig. **2**, we find that λ_{in} is much smaller and λ_{out} is much larger than the bulk value λ_0 in both theory and experiment. This means that the optimal microscopic Shubnikov structure results from the competition between optimizing the layer spacing and minimizing the number $N_{\text{out}} - N_{\text{in}}$ of point defects. This deviation is most significant in extreme confinement. For small inclusions, N_{in} can even become larger than the maximal number of rods that can be packed around the wall with a planar orientation, i.e., $\lambda_{\text{in}} < \lambda_{\text{in}}^*$, which results in the tilt shown in Fig. 4f of the main manuscript. Moreover, there are extreme structures without any defects for sufficiently small distances between the walls, e.g., the theory predicts a stable $\mathcal{S}_{19,19}$ for $R_{\text{out}} = 4.1L$ and $b = 0.8$.

Laminar–Shubnikov composite state $\mathcal{C}^{\mathcal{LS}}_{N_{\text{con}},N_{\text{in}},N_{\text{out}}}$. In a given Laminar–Shubnikov composite state $\mathcal{C}^{\mathcal{LS}}_{N_{\text{con}},N_{\text{in}},N_{\text{out}}}$, there is a precise number N_{con} of connected layers, reflecting the characteristics of the laminar state. The number of perpendicular layers separated by the adjacent disclination line (if present) constitutes here an important detail, since the location of the connected layers relative to the center of the annulus is not fixed by symmetry, in contrast to the laminar state. Therefore, this composite state is characterized by two further numbers N_{in} and N_{out}, i.e., the total number of contacts with the two walls, reflecting the characteristics for the Shubnikov state.

The stability of such a laminar–Shubnikov composite state can be explained by a mutual relaxation of the external constraints, which we understand as follows. The innermost connected layer in the laminar part can take the optimal position relative to the inclusion that minimizes the penalty arising from deformations and false wall alignment. In contrast, the arrangement of the layers in an ordinary laminar state is dictated by its axial symmetry. The same is true in view of the optimal layer spacing at the boundaries in the Shubnikov part of the structure, which is restricted to discrete values in the ordinary Shubnikov state. Further note that the intersection of the two half-structures is smooth, i.e., it does not introduce additional domains or deformations.

Supplementary Note 5. Topological protection of mesoscopic defect structures

Here we make some more statements regarding the stability of the different states deduced from their topological details. As elaborated in the main manuscript, the total topological charge Q, which is the sum of the charge of all occurring defects (compare Fig. 5 of the main manuscript), reflects the Euler characteristic χ of the bounding domain. Hence, in annular confinement we have $Q = \chi = 0$. Due to the typically different types of defects and their spatial distribution (oppositely charged defects are separated by rigid layers), the $\mathcal{L}$, $\mathcal{C}^{\mathcal{LS}}$ and $\mathcal{S}$ states are topologically protected, i.e., they cannot be transformed into one another upon a smooth variation of the particle distribution.

In contrast, we find that the theoretically created domain structures are not topologically protected. In fact,

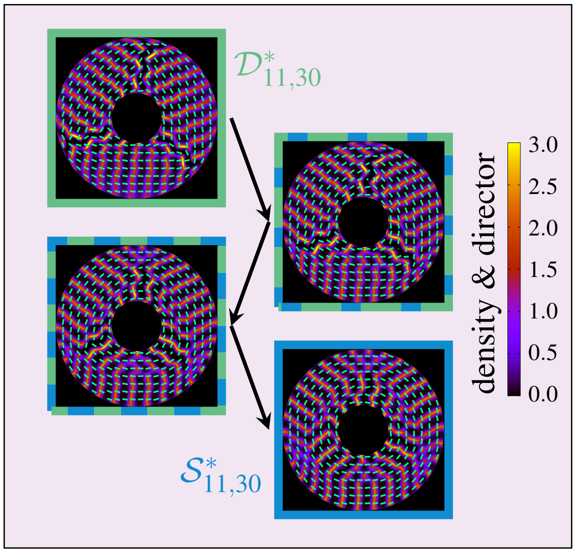

Supplementary Figure 3. Equilibration of a density profile initialized as a domain structure for $R_{\text{out}} = 6.3L$ and $b = 0.3$. The sequence of intermediate results indicated by the arrows shows a continuous evolution into a Shubnikov structure with the same numbers of layers. Each plot depicts a heat map of the orientationally averaged center-of-mass density $\bar{\rho}(\mathbf{r})$, given by Supplementary Equation (8), and arrows indicating the orientational director field prescribed by the local order tensor from Supplementary Equation (9).

they are unstable with respect to topologically equivalent Shubnikov structures, as depicted in Supplementary Fig. **3**. In the course of the minimization of DFT, the end-point defects of the radial disclination lines gradually annihilate, while the layers at the boundary become larger, eventually resulting in a uniform orientational order. The chosen annular geometry thus prefers the Shubnikov state. However, keeping in mind the experimental observations, we suspect that a domain state can become stable, e.g., by increasing the density or adding some polydispersity. In general, it should become more likely to observe a domain state in other confining geometries respecting the threefold symmetry.

An alternative and somewhat more insightful interpretation of the topologically protected states can be made by considering the inclusion as a topological defect itself, which possesses an own winding number k. In the Shubnikov state $\mathcal{S}$, we have $k = 1$ representing the uniformly bent director field parallel to the wall. Whenever the rods are misaligned along the integration path around the inclusion k is reduced by $-1/2$. Thus we have $k = 1/2$ for $\mathcal{C}^{\mathcal{LS}}$ and $k = 0$ for $\mathcal{L}$. Thereby, the negative half-integer boundary charges described in the main manuscript are absorbed into k and do not contribute to the total charge $\tilde{Q}$, which is therefore different in these three states when choosing the current interpretation. Here, the charge conservation resulting in topological protection becomes directly apparent. However, the conservation law between $\tilde{Q}$ and the geometric quantity χ has to be generalized to $\chi = \tilde{Q} + k - h$, where h denotes the number of holes in the confining domain ($h = 0$ in circular and $h = 1$ in annular confinement) and thus still holds. Further note that for the domain state, it is not possible to unambiguously define the winding number k due to the presence of the radial line disclinations, which underlines its topological instability.

Supplementary Note 6. Response of microscopic layering to geometric changes

The identified topologically protected states ($\mathcal{L}$, $\mathcal{C}^{\mathcal{LS}}$ and $\mathcal{S}$) even remain metastable over a large range of inclusion sizes as analyzed in Fig. 6 of the main manuscript. However, we are further interested in the particular microscopic layer structure corresponding to a certain state, whose stability is discussed below in more detail.

An initial intrinsic laminar structure $\mathcal{L}_{N_{\text{con}},N_{\text{dis}}}$ remains invariant upon smoothly changing b until the layers do not fit the geometry any more, e.g., if $N_{\text{con}}\lambda_0 > R_{\text{out}} - R_{\text{in}}$, which then results in an irreversible formation of defects. Hence, there exist some (metastable) laminar–laminar transitions, as also observed in Fig. 6 of the main manuscript.

In contrast, the number of edge dislocations in a given Shubnikov structure $\mathcal{S}_{N_{\text{in}},N_{\text{out}}}$ is not topologically protected and thus adapts to the geometrical changes as soon as the local layer spacing near a boundary differs too much from the bulk value. This comes along with some hysteresis effects upon first decreasing b and then returning to its original value. In particular, Supplementary Fig. **14** depicts that, for all structures considered in Fig. 6 of the main manuscript, the number N_{in} of layers in contact with the inclusion decreases from $N_{\text{in}} = 11$ to $N_{\text{in}} = 10$ upon decreasing the inclusion size ratio from $b = 0.28$ to $b = 0.27$, Upon a subsequent reversal from $b = 0.27$ back to $b = 0.28$ the contact number $N_{\text{in}} = 10$ remains the same for two of the structures considered (shown in the two top rows), whose energy is then clearly larger than at the beginning ($b = 0.28$). The other structure (bottom row) gradually develops another contact and becomes similar to the original one with $N_{\text{in}} = 11$. Further increasing the size of the inclusion to $b = 0.29$, all structures have $N_{\text{in}} = 11$ contacts, which closes all hysteresis loops. In fact, their energy lies slightly below that of the original structures for $b = 0.29$, which indicates that a fluctuating confinement aids the equilibration. On the other hand, starting with the metastable structure $\mathcal{S}_{12,30}$ for $b = 0.3$, the hysteresis loop (shown in Supplementary Fig. **15**) via $b = 0.29$ includes in a new structure $\mathcal{S}_{11,30}$ for $b = 0.3$ which is much more stable than the original one. This is not surprising, since the global minimum for $b = 0.3$ also has $N_{\text{in}} = 11$.

The composite states $\mathcal{C}^{\mathcal{LS}}_{N_{\text{con}},N_{\text{in}},N_{\text{out}}}$ do not show any microscopic changes in the number of edge dislocations over the range of b considered in Fig. 6 of the main

manuscript, since the value of the local layer spacing $\lambda_{\rm in}$ at the inclusion in the Shubnikov part of the structure can be balanced by a deformation of the adjacent laminar layer. If a hysteresis loop similar to the Shubnikov state exists here, it thus must be much larger.

Supplementary Note 7. Dependence on intrinsic parameters

Apart from the external topological and geometrical constraints, the formation of smectic structures can also be controlled by indirectly tuning the preferred layer spacing λ_0 in bulk. This intrinsic structural property of the smectic liquid crystal depends on the size and aspect ratio of the particles, as well as, the total density. In the following, we briefly explore this extended parameter space within DFT in more detail.

Changes in density and particle shape. Increasing the density, the bulk layer spacing decreases and packing effects become more important. To explore the effect on the smectic states, we consider again the density profiles for $R_{\rm out} = 6.3L$ and $b = 0.3$ shown in Fig. 6 of the main manuscript (see also Supplementary Fig. **13**). Increasing the density, the global minimum gradually shifts from a Shubnikov to a composite state, as shown in Fig. 7a of the main manuscript and then to a laminar state (not shown). The optimal microscopic structure of the different states also changes for a higher density, since, for example, a larger value of $N_{\rm out}$ becomes more favorable.

The dependence of the state diagram on the absolute particle size can be extracted from the scaling used in Fig. 3 of the main manuscript. Decreasing now the aspect ratio of the rods to $p = 5$ for a fixed density and relative system size $R_{\rm out} = 6.3L$, we find that the Shubnikov state is still stable for $b = 0.26$, cf. Supplementary Fig. 7b, although the absolute system size $R_{\rm out}$ has accordingly decreased by a factor two. The laminar–Shubnikov transition occurs thus at a smaller value $b \approx 0.259$ than for more elongated rods. Hence, we conclude that the Shubnikov state is most stable when considering systems of small and short rods at a low density within the smectic regime.

Relation to nematic states in an annulus. As discussed in the main manuscript, some of the observed smectic states possess topologically equivalent nematic states at lower density. Here, we elaborate on two further questions. Can we infer the existence of additional smectic states from nematic ones with the same symmetry? How does the transition from nematic to smectic states occur upon increasing the density?

To answer the first question, we undertook some efforts to stabilize a three-fold symmetric smectic structure that behaves to the nematic D_3 state[12] like the laminar state to the nematic N_2. This means that there are

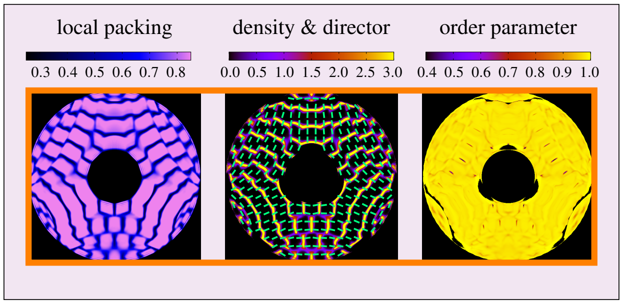

Supplementary Figure 4. Metastable smectic state with threefold symmetry analog to a D_3 nematic state for $R_{\rm out} = 6.3L$, $b = 0.3$ and $\eta = 0.75$, depicted in the same representations as in Fig. 2 of the main manuscript (mind the differences due to the higher overall density): local packing fraction $n_2(\mathbf{r})$, compare Supplementary Equation (4), orientationally averaged density $\bar{\rho}(\mathbf{r})$ and director field, compare Supplementary Equations (8) and (9), and the local order parameter, i.e., the largest Eigenvalue of the order tensor $Q(\mathbf{r}, \phi)$, compare Supplementary Equation (9). Note that the free energy is much higher than for the other states reported in the main manuscript using the same parameters, as there is a large number of deformations required to fit into the given geometry.

three anti-radial disclination lines of charge $q = 1/2$ and three regions of $q = -1/2$ charged misalignment at the inclusion, whose winding number would be $k = -1/2$ (compare Supplementary Note 5). We show in Supplementary Fig. **4** that such a state indeed exists. However, it is highly metastable as the layers are subject to a strong bend. Even with this expensive deformation, there is not enough space for the layers hosting the rods oriented tangentially to the inclusion, to extend towards the outer wall without forming additional defects. We can only speculate that such a structure can be stabilized in different geometries possessing a threefold symmetry, as, for example, one with hexagonal walls.

The second question is addressed in Supplementary Fig. **5**, where we created five distinct nematic states at an area fraction $\eta = 0.5$ and subsequently increased the density toward the bulk nematic–smectic transition threshold $\eta = 0.62$ in our theory. Note that most of the shown structures are not fully equilibrated, as for example, the nematic states D_4 and D_5 would destabilize at the low nematic densities. This procedure allows us to mimic, in a rough way, the nonequilibrium densification observed during the sedimentation process in the experiment. We make two main observations. First, the density at which we observe an onset of smectization depends on the nematic state. More precisely, it appears that this density is lower if the deformations of the nematic director field are stronger, as in D_0 and D_5. Second, the initially emerging smectic patterns still follow the orientational director field of the nematic states and thus become frustrated in their positional order. This nicely underlines that the distinct director field of the equilibrated smectic structures is favorable and underlines our suspicion that,

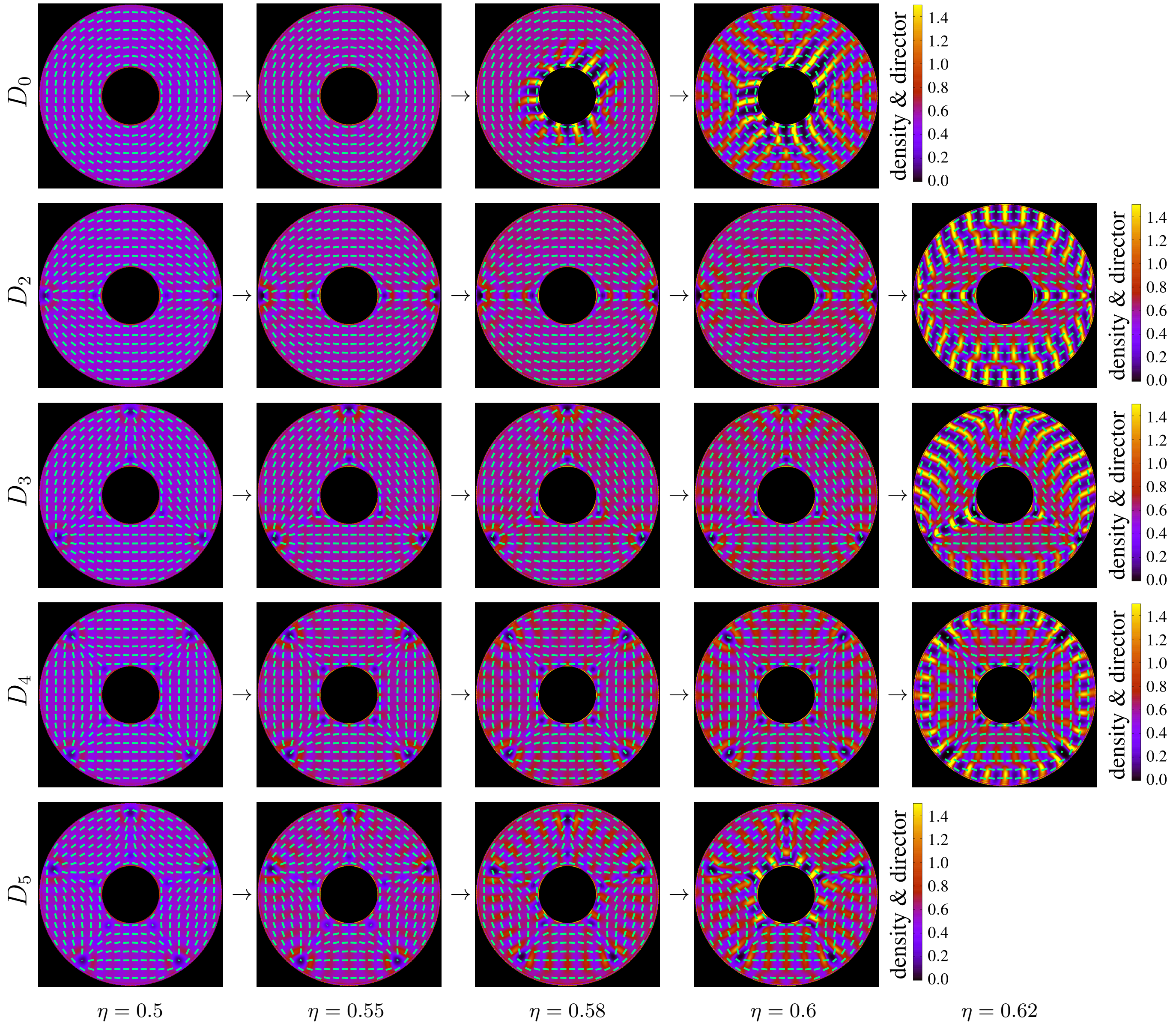

Supplementary Figure 5. Onset of smectic order emerging from different nematic states D_n upon increasing the density in discrete steps until the bulk transition density $\eta = 0.62$ is reached (see labels) for $R_{\text{out}} = 6.3L$ and $b = 0.3$. Not all structures are fully equilibrated, such that the otherwise unstable structures with $n = 4$ and $n = 5$ defect pairs could also be included. Color bar and arrows denote the orientationally averaged density and the director field, compare Supplementary Equations (8) and (9), respectively. Note that the color bar only has half the range as in the all other density plots throughout the Supplementary Information and the main manuscript.

in experimental sedimentation equilibrium, the structure of the nematic states is influenced by the smectic states below and not vice versa.

Supplementary Note 8. Collection of analyzed data

In this section we show the collected theoretical (Supplementary Figs. **6** to **15**) and experimental data (Supplementary Figs. **16** to **25**) and provide some additional background information. Note that the experimental snapshots are compressed to reduce the file size.

Theory. Supplementary Figs. **6** and **7** depict the laminar structures $\mathcal{L}_{N_{\text{con}},N_{\text{dis}}}$ from which the numbers N_{con} and N_{dis} of connected and disconnected layers, shown in Supplementary Fig. **1**, can be counted. Supplementary Figs. **8**, **9** and **10** depict the Shubnikov structures $\mathcal{S}_{N_{\text{in}},N_{\text{out}}}$ from which the numbers N_{in} and N_{out} of inner and outer layers in contact with the two boundaries, shown in Supplementary Fig. **2**, can be counted.

Supplementary Figs. **11** and **12** depict the stable laminar (smaller inclusion size ratio b_1) and Shubnikov (larger inclusion size ratio b_2) structures used to locate transition between these two states, which is shown in Fig. 3 of the main manuscript. To estimate the intermediate value of b at which the transition occurs we first create the metastable Shubnikov structure for b_1 and the metastable laminar structure for b_2 by accordingly adapting the confining geometry for the depicted global minima. Then we approximate the free energy for $b_1 \leq b \leq b_2$ of both states as linear functions determined by the values at b_1 and b_2. The value b_t where both interpolated functions are equal serves as our estimate for the location of the laminar–Shubnikov transition.

Supplementary Fig. **13** depicts a representative selection of structures whose relative free energy is compared in Figs. 6 and 7 of the main manuscript.

Supplementary Figs. **14** and **15** illustrate the hysteresis effects in the Shubnikov state discussed in Supplementary Note 6.

Experiment. The experimental snapshots are organized by the total chamber size R_out, reaching from the smallest (Supplementary Figs. **16** and **17**) to the largest confinements (Supplementary Figs. **24** and **25**). For each chamber size, we organize the structures by the inclusion size ratio b, with the smallest value at the top. The color of the frame reflects the particular state according to the legend of the phase diagram in Fig. 3 of the main manuscript. From low to high gray levels, we distinguish between the laminar state (yellowish), composite laminar-domain, domain (greenish), composite domain-Shubnikov, Shubnikov (blue), and composite Shubnikov-laminar. Chambers that were damaged during the fabrication process are not shown resulting in a smaller amount of data for some b.

All experimental snapshots are provided in two forms: (i) raw bright field images (Supplementary Figs. **16**, **18**, **20**, **22** and **24**) and (ii) processed bright field images (Supplementary Figs. **17**, **19**, **21**, **23** and **25**). By processing images we are able to measure the position and orientation of a majority of the rods present in experimental snapshots. This information is used to color the rods as a function of their orientation relative to the nearest wall, given by an angle ranging from 0 to π, where 0 and π are identified. We use a periodic color scheme, where light blue indicates anti-radially oriented rods (0 and π) and red indicates radially oriented rods ($\pi/2$). This image processing eases the identification of defects. The anti-radial disclination lines (in the laminar state and some composite states) can be identified by a 'rainbow-like' color pattern, see e.g. the first row of Supplementary Fig. **21**. On the other hand, the radially orientated disclination lines (in the domain state and some composite states) generate a 'dark-blue/green' bi-color pattern, see e.g., the third row of Supplementary Fig. **21**. When the color is homogeneous, there are no line defects and only edge dislocations characteristic to the Shubnikov state occur.

Supplementary References

[1] Cortes, L. B. G., Gao, Y., Dullens, R. P. A. & Aarts, D. G. A. L. Colloidal liquid crystals in square confinement: isotropic, nematic and smectic phases. *J. Phys. Condens. Matter* **29**, 064003 (2016).

[2] Cortes, L. *Colloidal liquid crystals: phase behavior, dynamics and confinement.* Ph.D. thesis, University of Oxford (2019).

[3] Onsager, L. The effects of shape on the interaction of colloidal particles. *Ann. NY Acad. Sci.* **51**, 627–659 (1949).

[4] Grelet, E. Hard-rod behavior in dense mesophases of semiflexible and rigid charged viruses. *Phys. Rev. X* **4**, 021053 (2014).

[5] Stroobants, A., Lekkerkerker, H. N. W. & Odijk, T. Effect of electrostatic interaction on the liquid crystal phase transition in solutions of rodlike polyelectrolytes. *Macromolecules* **19**, 2232–2238 (1986).

[6] van der Beek, D. & Lekkerkerker, H. N. W. Liquid crystal phases of charged colloidal platelets. *Langmuir* **20**, 8582–8586 (2004).

[7] Bolhuis, P. & Frenkel, D. Tracing the phase boundaries of hard spherocylinders. *J. Chem. Phys.* **106**, 666–687 (1997).

[8] Evans, R. The nature of the liquid-vapour interface and other topics in the statistical mechanics of non-uniform, classical fluids. *Adv. Phys.* **28**, 143–200 (1979).

[9] Wittmann, R., Sitta, C. E., Smallenburg, F. & Löwen, H. Phase diagram of two-dimensional hard rods from fundamental mixed measure density functional theory. *J. Chem. Phys.* **147**, 134908 (2017).

[10] Wittmann, R., Marechal, M. & Mecke, K. Fundamental measure theory for smectic phases: scaling behavior and higher order terms. *J. Chem. Phys.* **141**, 064103 (2014).

[11] Roth, R. Fundamental measure theory for hard-sphere mixtures: a review. *J. Phys. Condens. Matter* **22**, 063102 (2010).

[12] Gârlea, I. C., Mulder, P., Alvarado, J., Dammone, O. J., Aarts, D. G. A. L., Lettinga, M. P., Koenderink, G. H. & Mulder, B. M. Finite particle size drives defect-mediated domain structures in strongly confined colloidal liquid crystals. *Nat. Commun.* **7**, 12112 (2016).

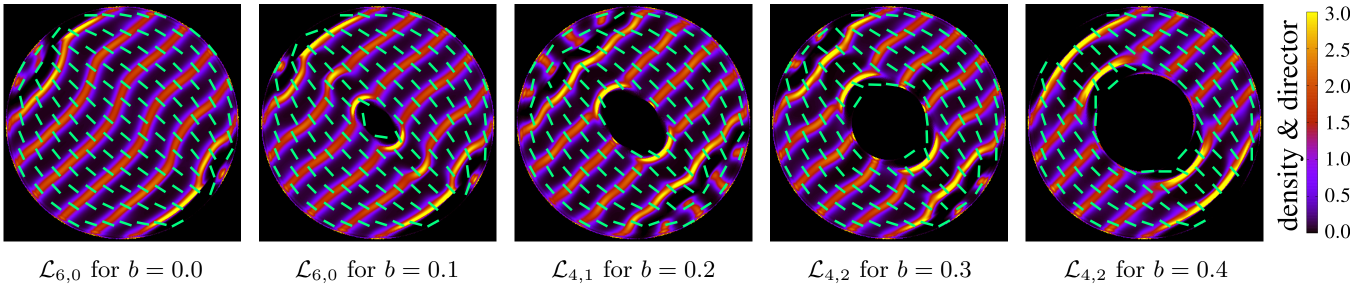

Supplementary Figure 6. Optimal laminar states for fixed $R_{\text{out}} = 4.1L$ and b as indicated. Note that the structures shown for $b \geq 0.3$ are metastable. Color bar and arrows denote the orientationally averaged density and the director field, compare Supplementary Equations (8) and (9), respectively.

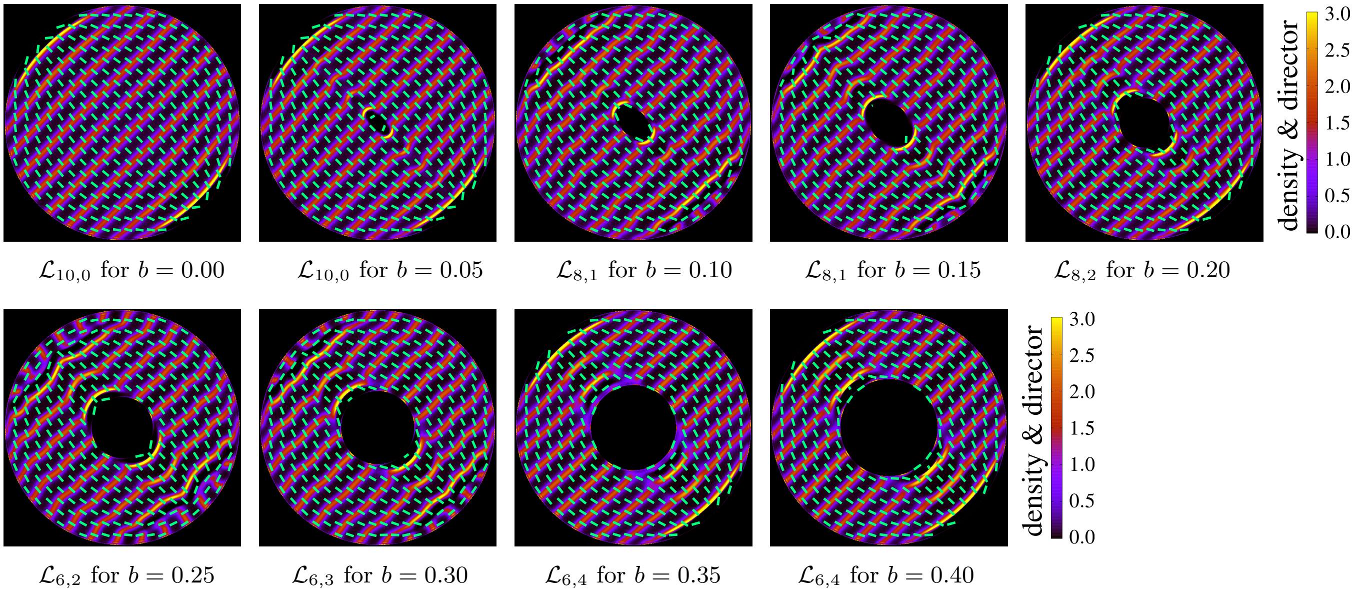

Supplementary Figure 7. Optimal laminar states for fixed $R_{\text{out}} = 6.3L$ and b as indicated. Note that the structures shown for $b \geq 0.3$ are metastable. Color bar and arrows denote the orientationally averaged density and the director field, compare Supplementary Equations (8) and (9), respectively.

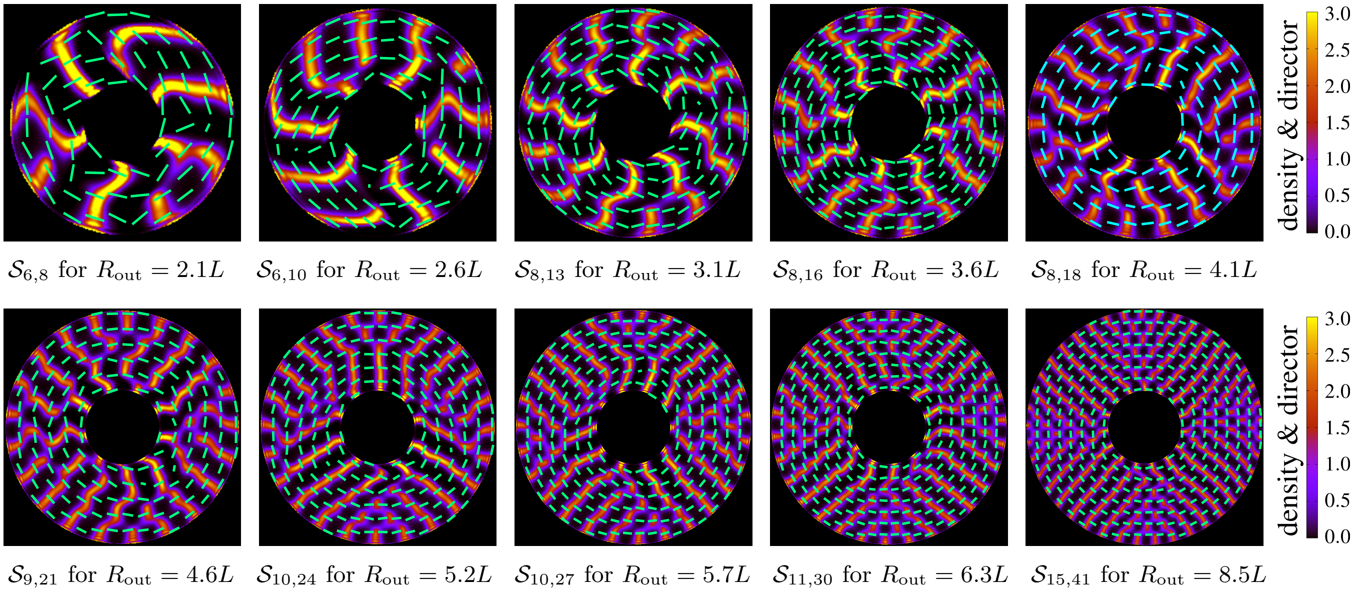

$\mathcal{S}_{6,8}$ for $R_{\rm out} = 2.1L$ $\mathcal{S}_{6,10}$ for $R_{\rm out} = 2.6L$ $\mathcal{S}_{8,13}$ for $R_{\rm out} = 3.1L$ $\mathcal{S}_{8,16}$ for $R_{\rm out} = 3.6L$ $\mathcal{S}_{8,18}$ for $R_{\rm out} = 4.1L$

$\mathcal{S}_{9,21}$ for $R_{\rm out} = 4.6L$ $\mathcal{S}_{10,24}$ for $R_{\rm out} = 5.2L$ $\mathcal{S}_{10,27}$ for $R_{\rm out} = 5.7L$ $\mathcal{S}_{11,30}$ for $R_{\rm out} = 6.3L$ $\mathcal{S}_{15,41}$ for $R_{\rm out} = 8.5L$

Supplementary Figure 8. Optimal Shubnikov states for $R_{\rm out}$ as indicated and fixed $b = 0.3$. Note that some of the structures shown for $R_{\rm out} \leq 5.7L$ may be metastable. Color bar and arrows denote the orientationally averaged density and the director field, compare Supplementary Equations (8) and (9), respectively.

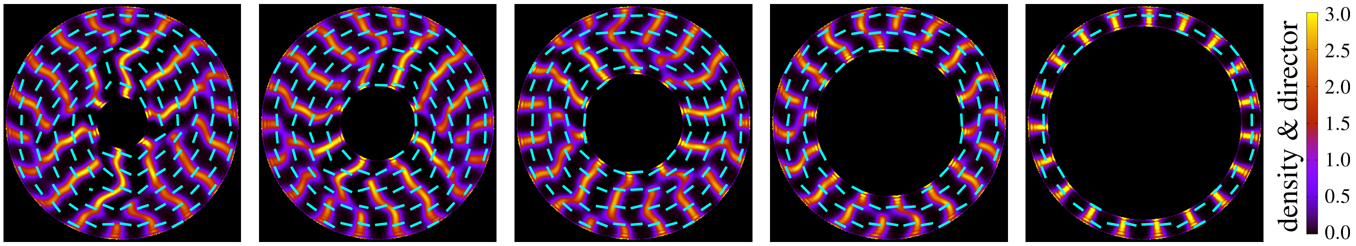

$\mathcal{S}_{6,18}$ for $b = 0.2$ $\mathcal{S}_{8,18}$ for $b = 0.3$ $\mathcal{S}_{10,19}$ for $b = 0.4$ $\mathcal{S}_{14,18}$ for $b = 0.6$ $\mathcal{S}_{19,19}$ for $b = 0.8$

Supplementary Figure 9. Optimal Shubnikov states for fixed $R_{\rm out} = 4.1L$ and b as indicated. Note that the structure shown for $b = 0.2$ is metastable. Color bar and arrows denote the orientationally averaged density and the director field, compare Supplementary Equations (8) and (9), respectively.

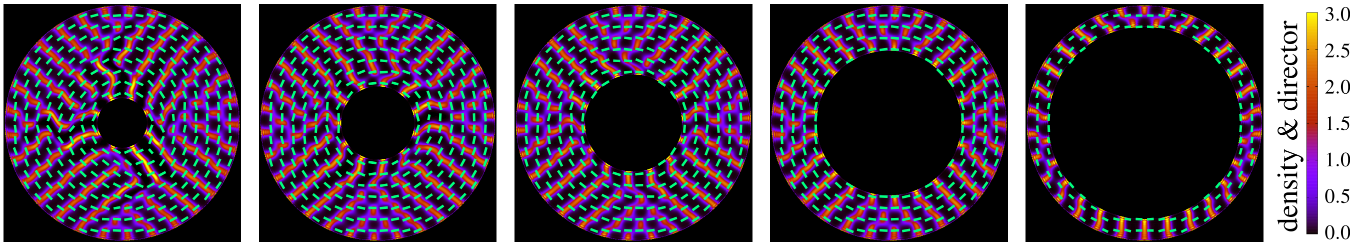

$\mathcal{S}_{8,29}$ for $b = 0.2$ $\mathcal{S}_{11,30}$ for $b = 0.3$ $\mathcal{S}_{14,30}$ for $b = 0.4$ $\mathcal{S}_{21,29}$ for $b = 0.6$ $\mathcal{S}_{27,30}$ for $b = 0.8$

Supplementary Figure 10. Optimal Shubnikov states for fixed $R_{\rm out} = 6.3L$ and b as indicated. Note that the structure shown for $b = 0.2$ is metastable. Color bar and arrows denote the orientationally averaged density and the director field, compare Supplementary Equations (8) and (9), respectively.

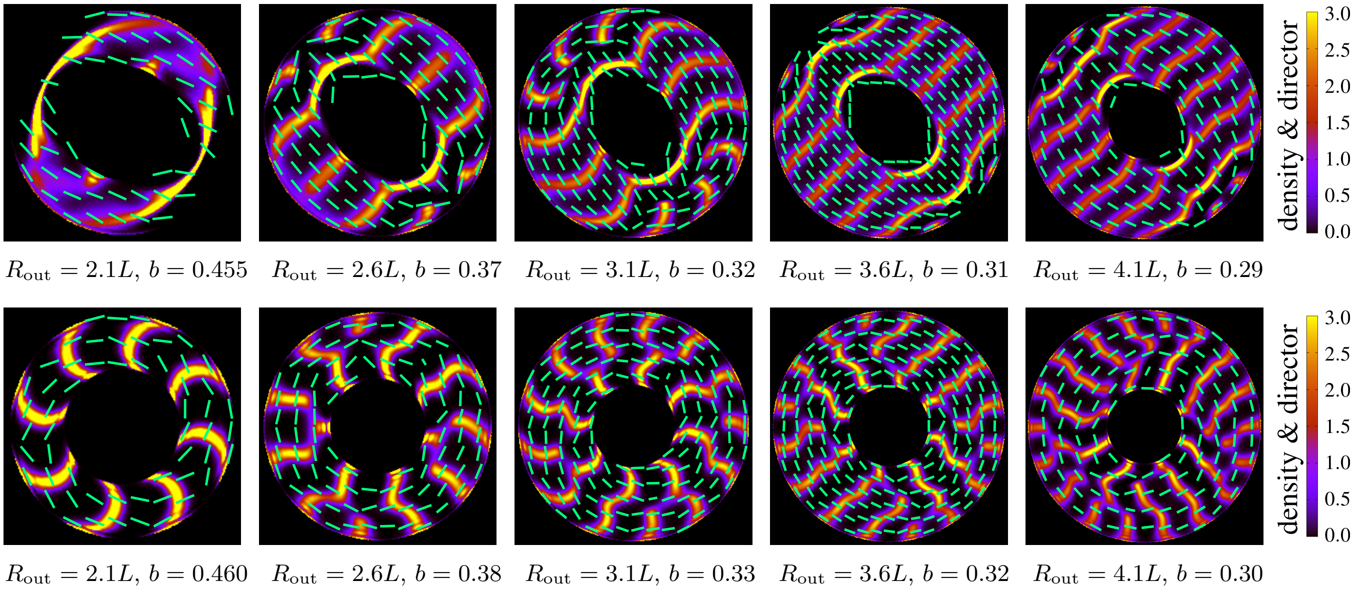

Supplementary Figure 11. Stable states close to the theoretical laminar–Shubnikov transition. For the different outer radii R_{out}, the first row shows the stable laminar state at indicated inclusion size ratio b and the second row shows the stable laminar state at a slightly larger value of b. Color bar and arrows denote the orientationally averaged density and the director field, compare Supplementary Equations (8) and (9), respectively.

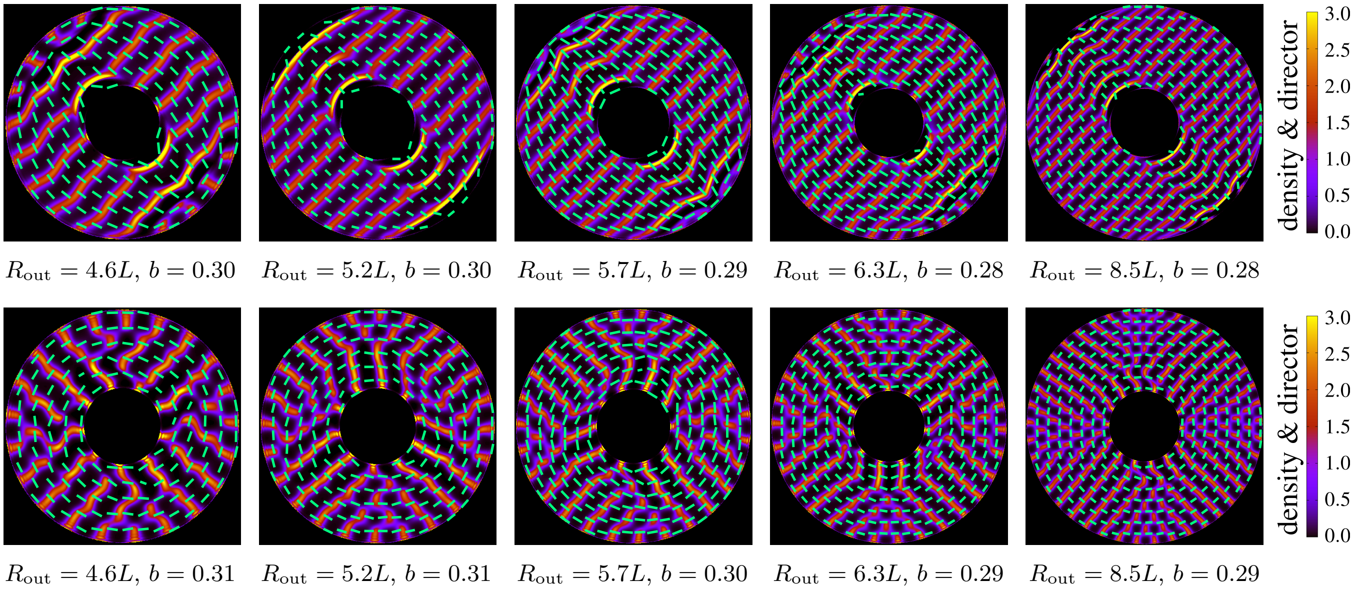

Supplementary Figure 12. Stable states close to the theoretical laminar–Shubnikov transition, as Supplementary Fig. **11**, but for different outer radii R_{out}. Color bar and arrows denote the orientationally averaged density and the director field, compare Supplementary Equations (8) and (9), respectively.

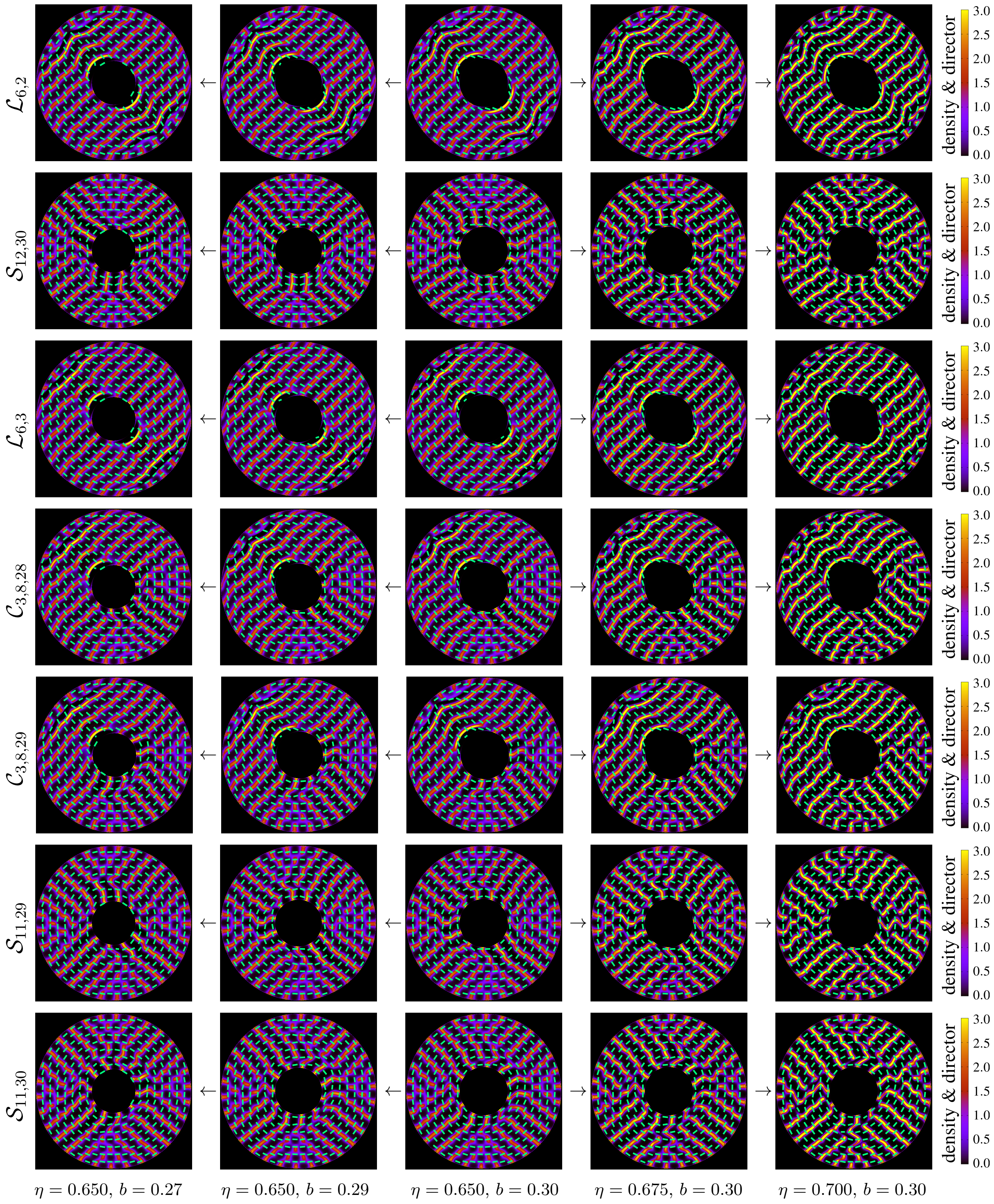

Supplementary Figure 13. Selection of stable and metastable structures for different inclusion size ratios b and packing fractions η. Each row corresponds a group of structures obtained from the initial structure for $\eta = 0.65$ and $b = 0.30$ (third column), as labeled on the left, by either decreasing b for fixed η (first two columns) or increasing η for fixed b (last two columns), as indicated by the arrows. Color bar and arrows denote the orientationally averaged density and the director field, compare Supplementary Equations (8) and (9), respectively.

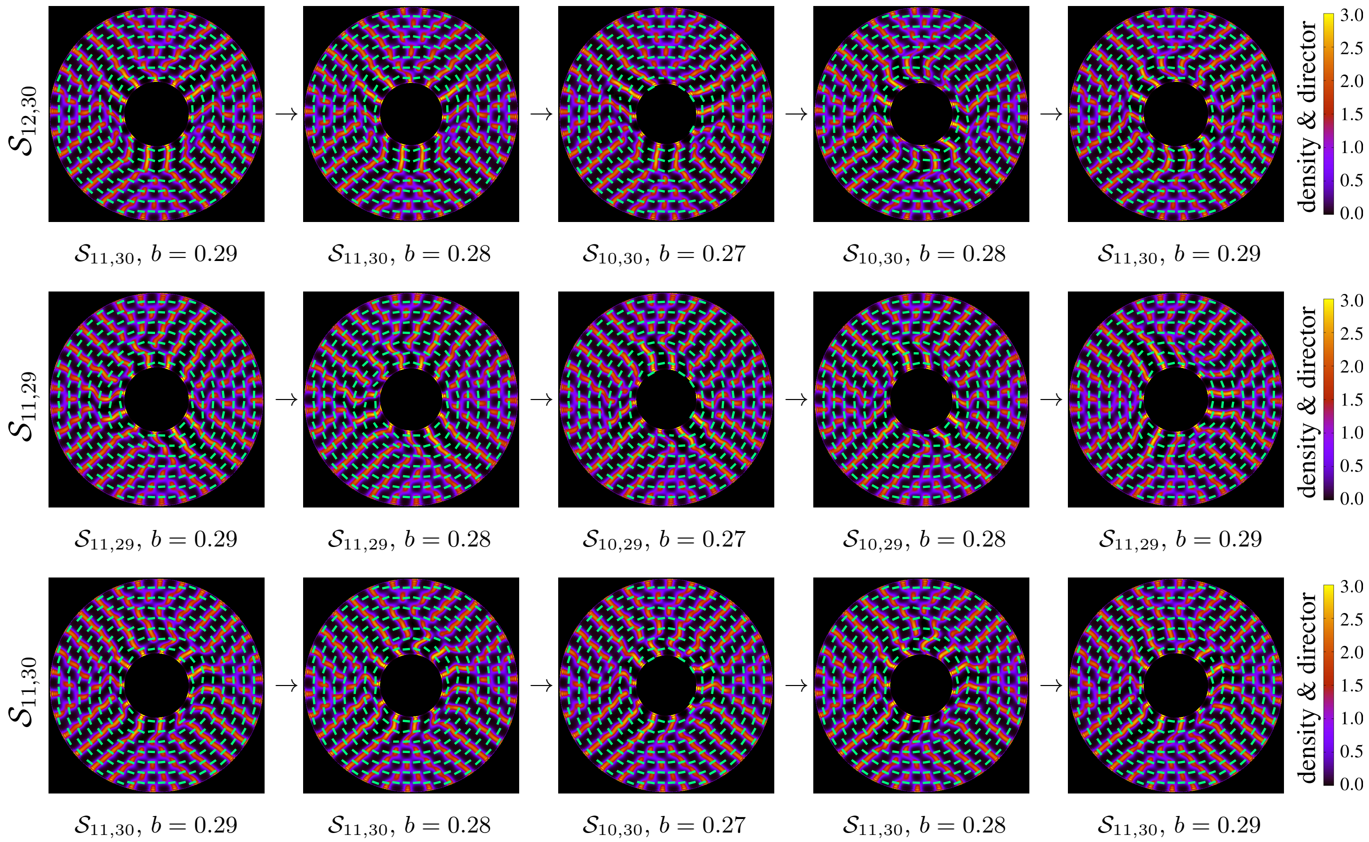

Supplementary Figure 14. Hysteresis in the Shubnikov state. Shown are the structures obtained by first decreasing the inclusion size ratio b from $b = 0.29$ down to $b = 0.27$ and then increasing b up to $b = 0.29$, in steps of 0.01. The initial structures for $b = 0.29$ are obtained from those for $b = 0.3$, shown in the third column of Supplementary Fig. **13** and labeled on the left (see Supplementary Fig. **15** for the transition from $\mathcal{S}_{12,30}$ to $\mathcal{S}_{11,30}$ omitted in the first row). Color bar and arrows denote the orientationally averaged density and the director field, compare Supplementary Equations (8) and (9), respectively.

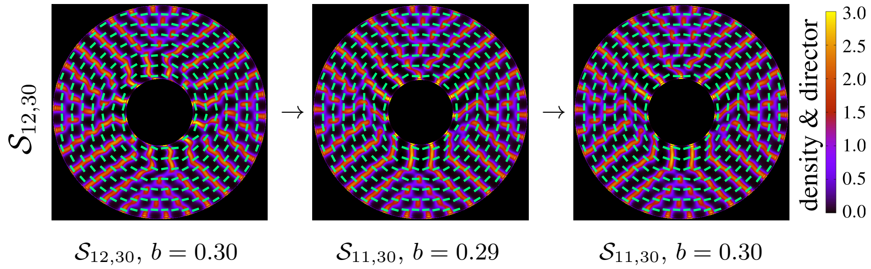

Supplementary Figure 15. Hysteresis in the Shubnikov state. Shown are the structures obtained from $\mathcal{S}_{12,30}$ by first decreasing the inclusion size ratio b from $b = 0.3$ down to $b = 0.29$ and increasing b back to $b = 0.3$. Color bar and arrows denote the orientationally averaged density and the director field, compare Supplementary Equations (8) and (9), respectively.

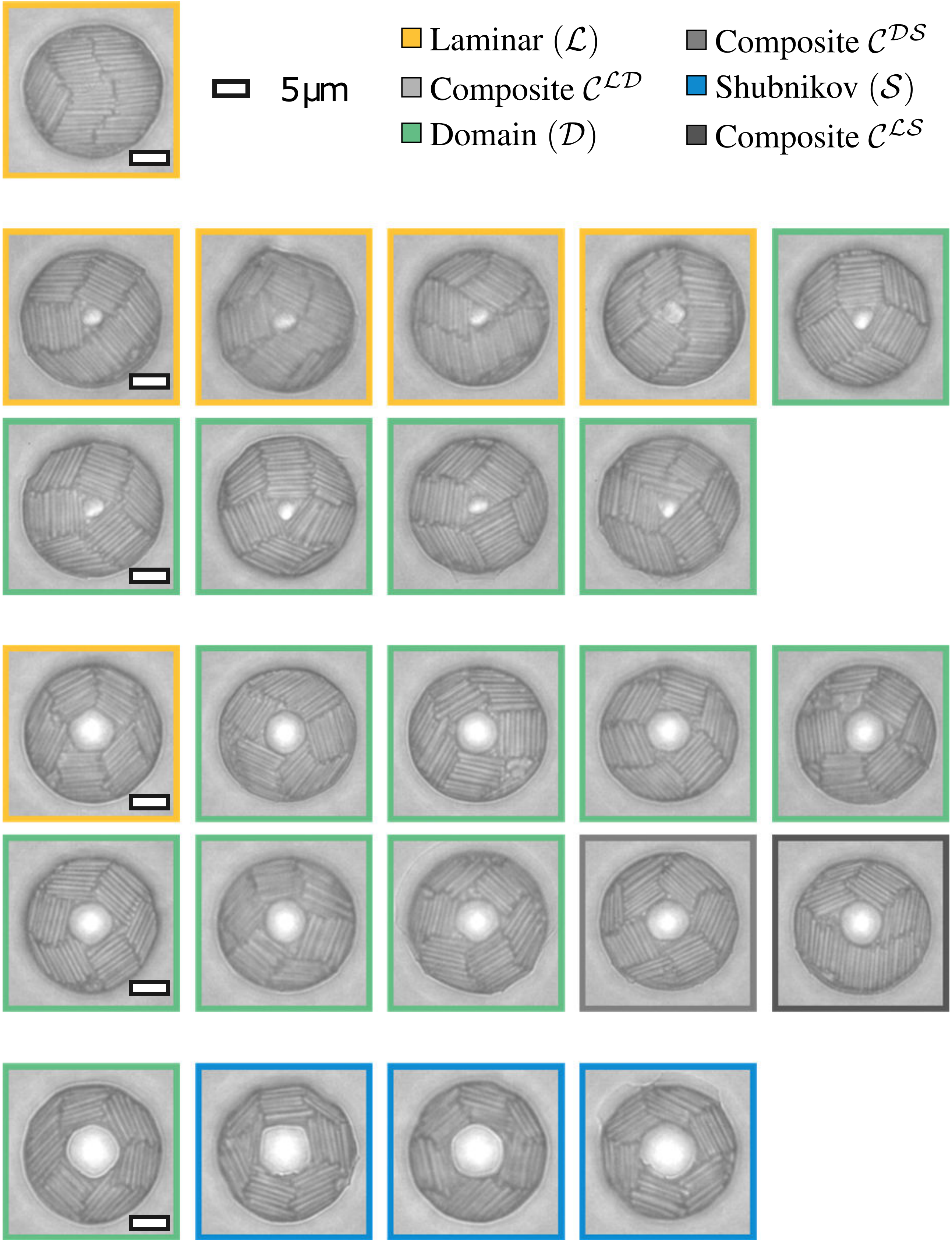

Supplementary Figure 16. Experimental snapshots for $R_{\text{out}} = 1.9L$. The color of the frame indicates the state according to the legend. Estimated inclusion size ratio from top to bottom: $b = 0$, $b = 0.15$, $b = 0.3$, $b = 0.41$.

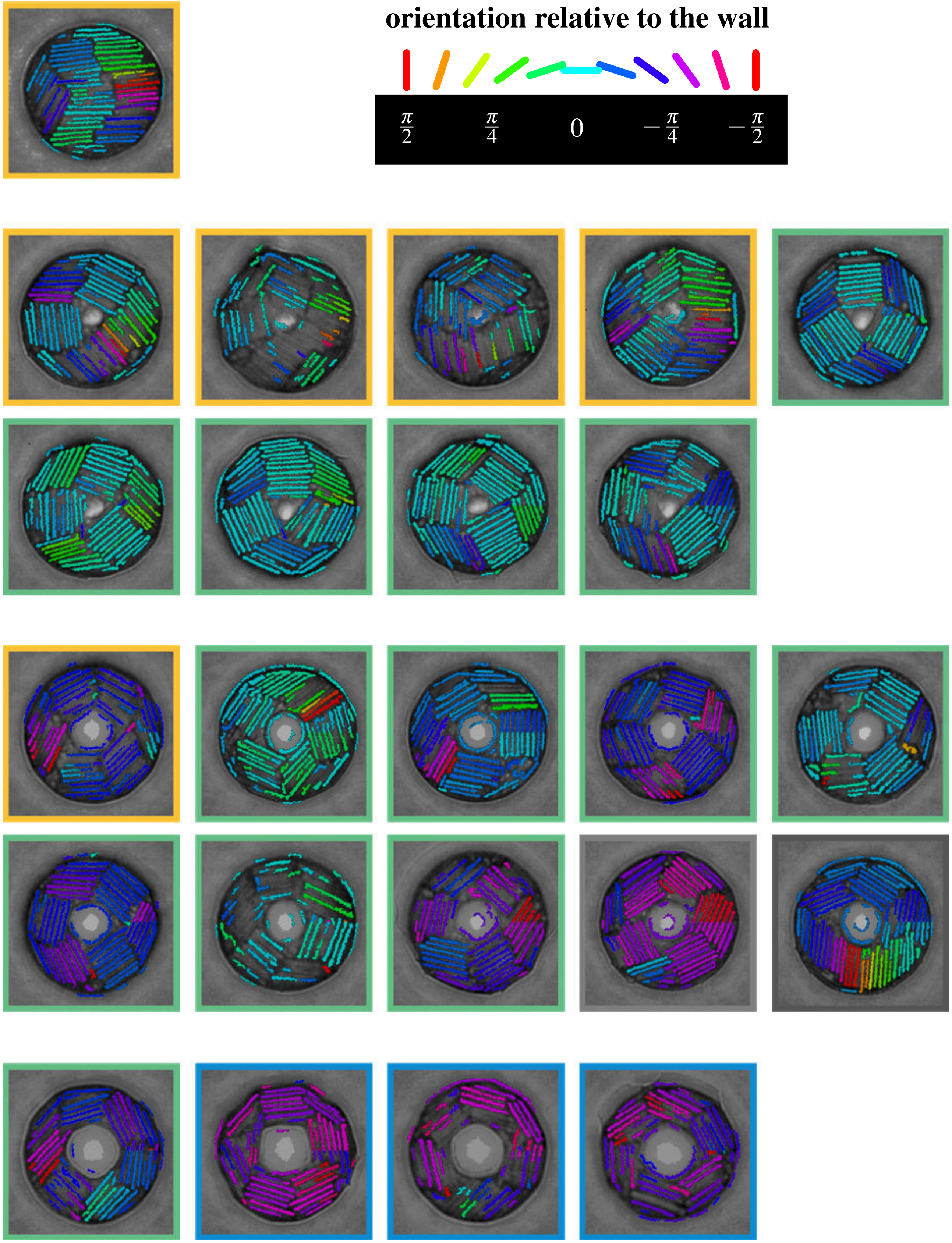

Supplementary Figure 17. As Supplementary Fig. **16** but with additional coloring of single rods indicating their relative orientation to the system boundaries according to the legend.

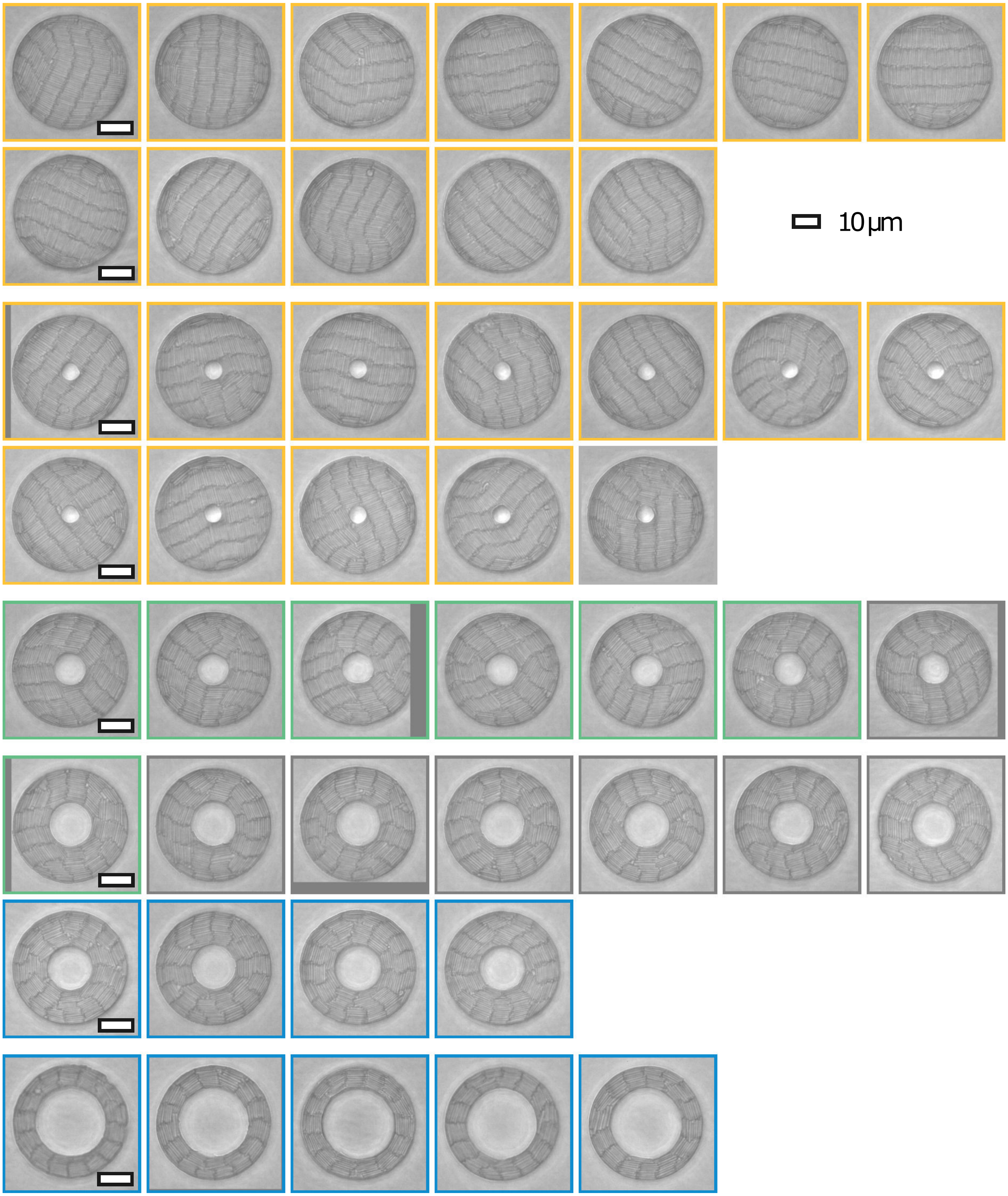

Supplementary Figure 18. Experimental snapshots for $R_{\mathrm{out}} = 3.8L$. The color of the frame indicates the state according to the legend in Supplementary Fig. **16**. Estimated inclusion size ratios from top to bottom: $b = 0$, $b = 0.15$, $b = 0.26$, $b = 0.41$, $b = 0.64$, $b = 0.76$.

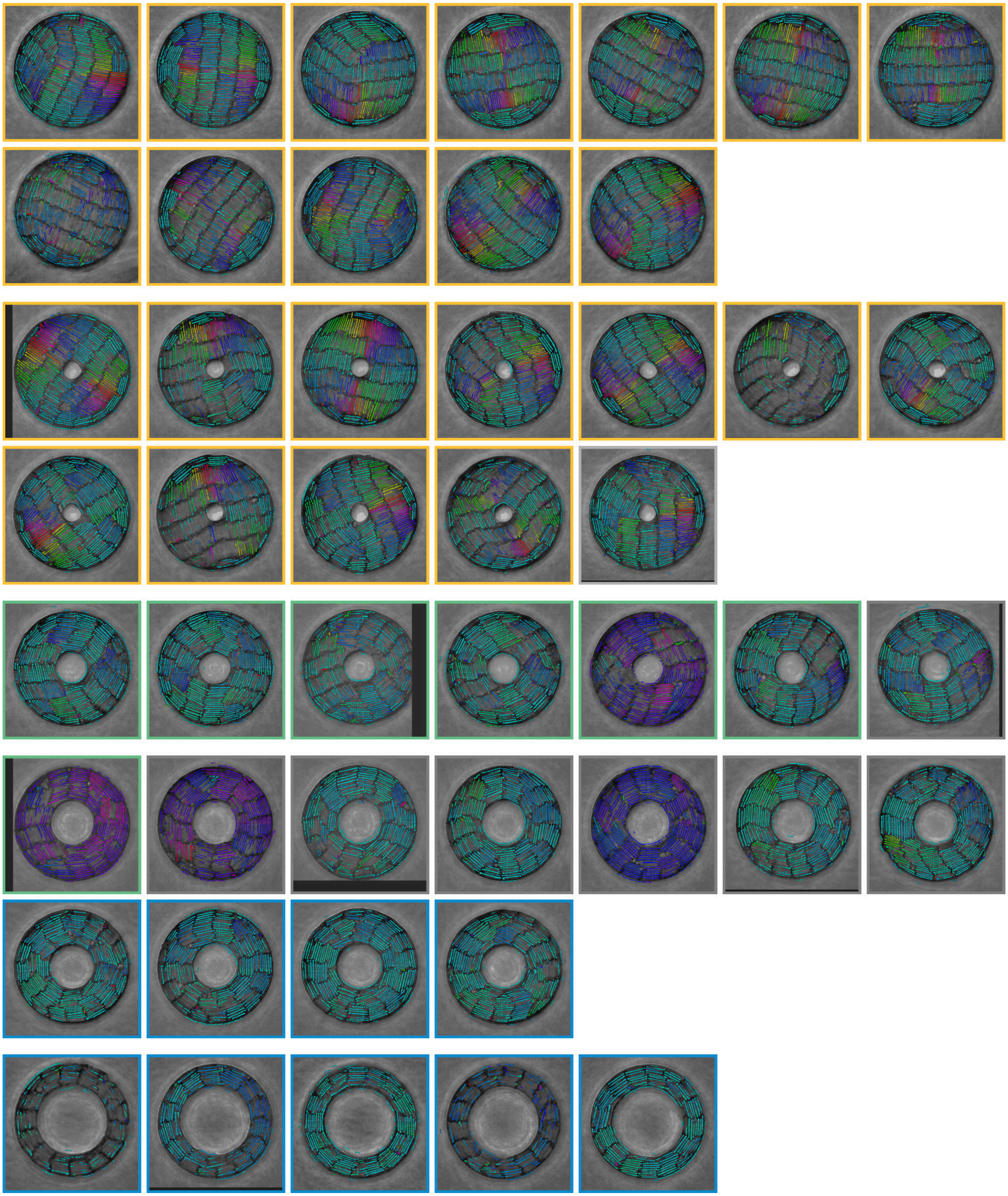

Supplementary Figure 19. As Supplementary Fig. **18** but with additional coloring of single rods indicating their relative orientation to the system boundaries according to the legend in Supplementary Fig. **17**.

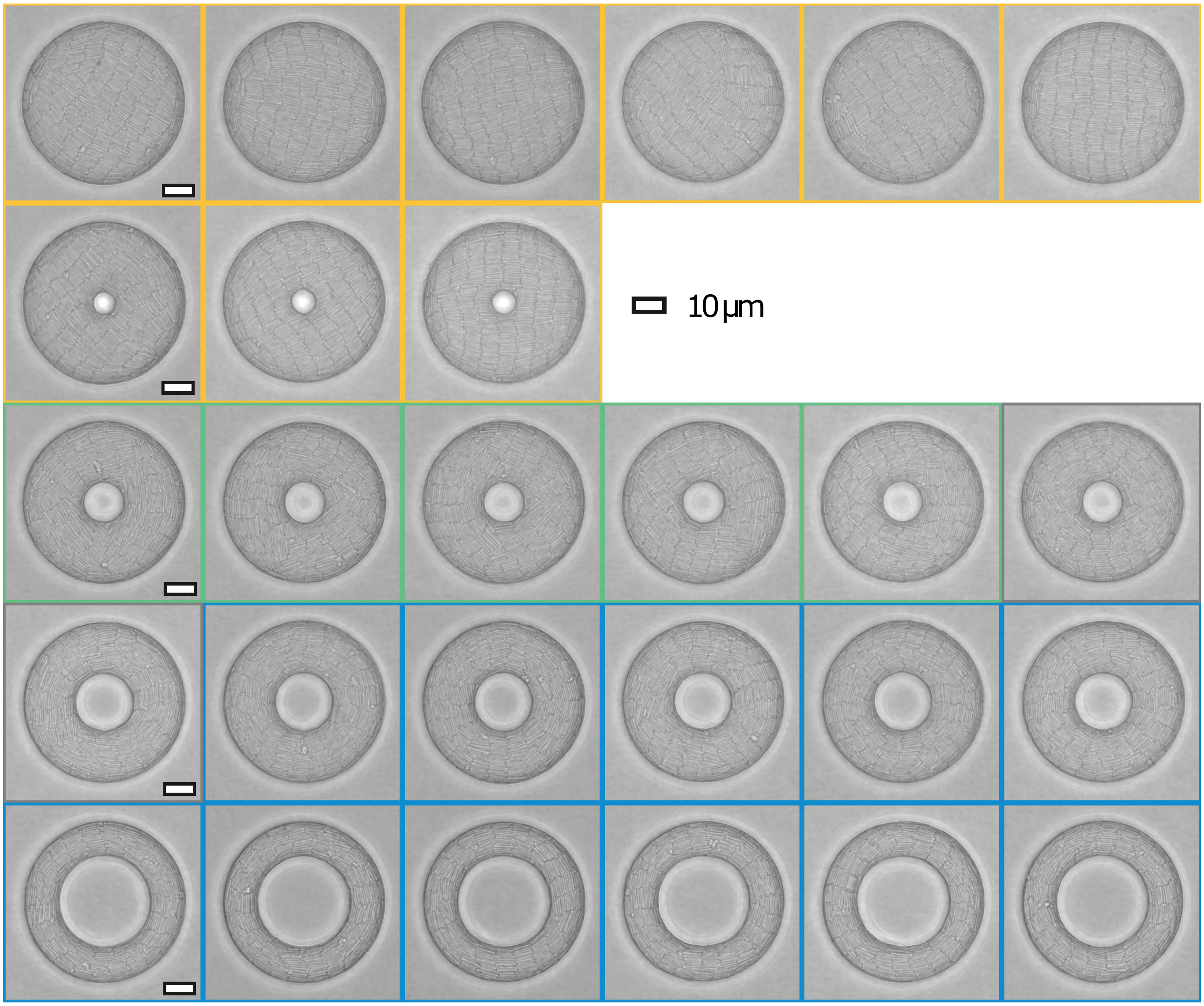

Supplementary Figure 20. Experimental snapshots for $R_{\mathrm{out}} = 5.7L$. The color of the frame indicates the state according to the legend in Supplementary Fig. **16**. Estimated inclusion size ratio from top to bottom: $b = 0$, $b = 0.15$, $b = 0.26$, $b = 0.36$, $b = 0.58$, $b = 0.76$.

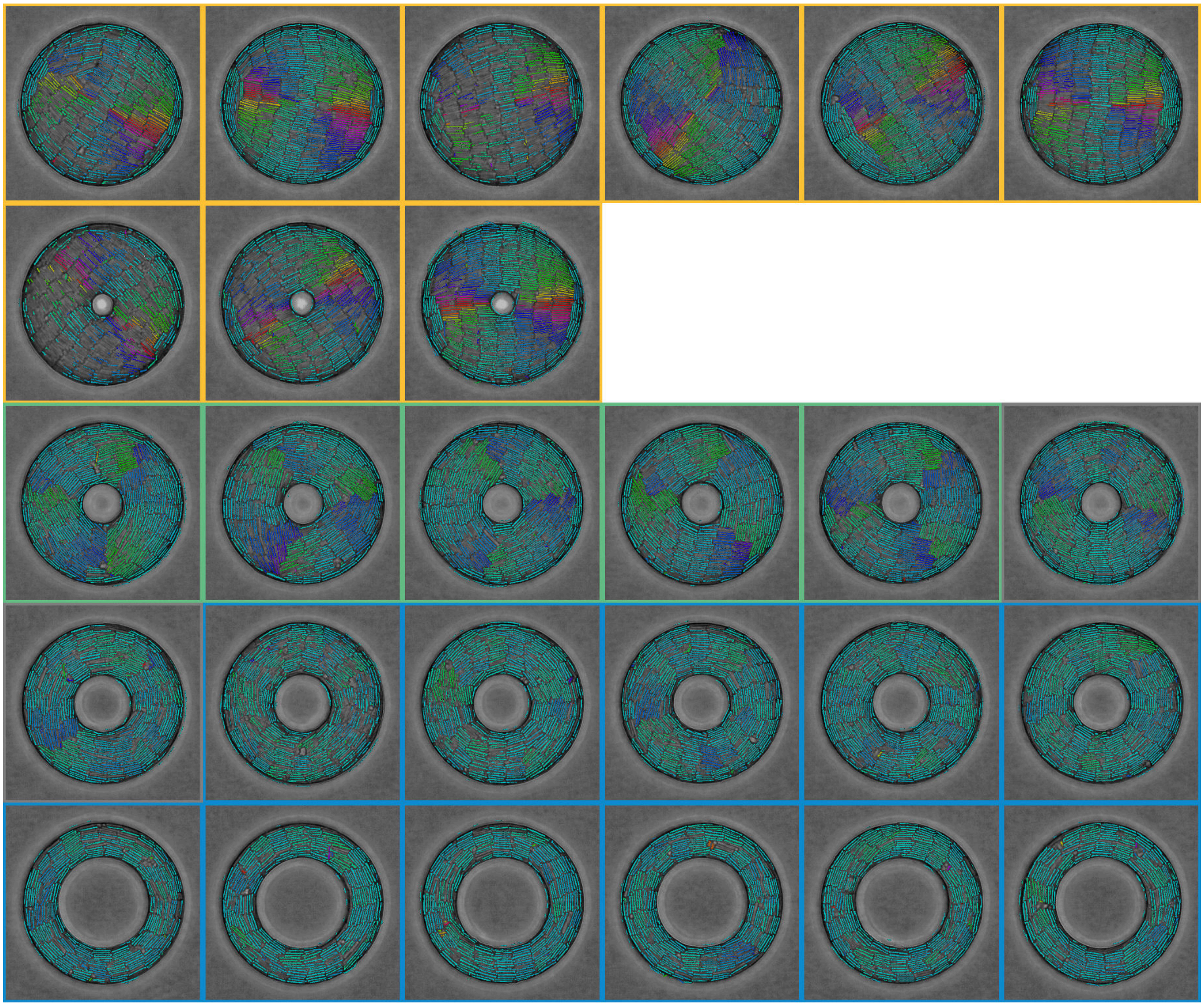

Supplementary Figure 21. As Supplementary Fig. **20** but with additional coloring of single rods indicating their relative orientation to the system boundaries according to the legend in Supplementary Fig. **17**.

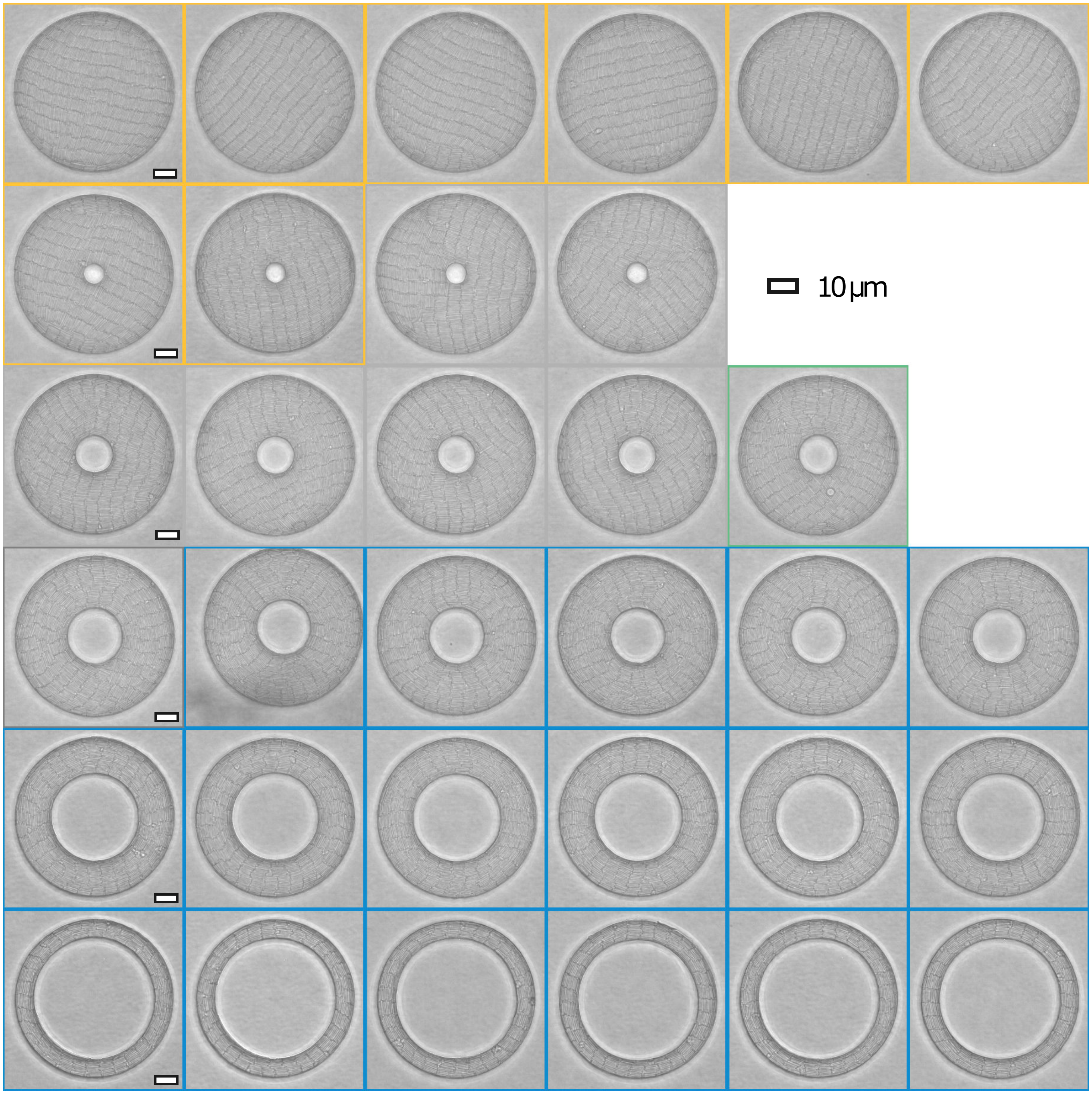

Supplementary Figure 22. Experimental snapshots for $R_{\mathrm{out}} = 7.8L$. The color of the frame indicates the state according to the legend in Supplementary Fig. **16**. Estimated inclusion size ratio from top to bottom: $b = 0$, $b = 0.12$, $b = 0.24$, $b = 0.35$, $b = 0.54$, $b = 0.76$.

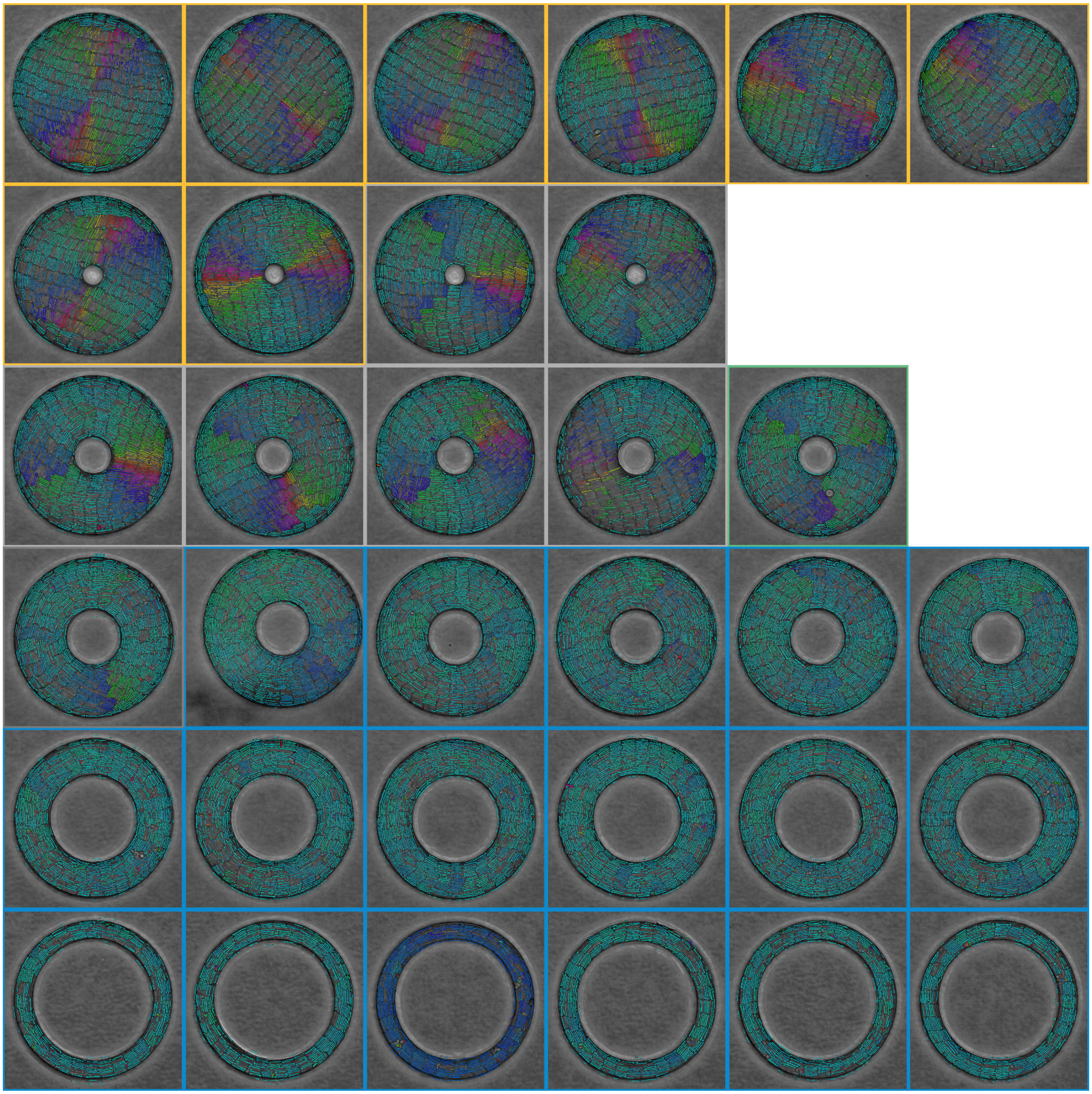

Supplementary Figure 23. As Supplementary Fig. **22** but with additional coloring of single rods indicating their relative orientation to the system boundaries according to the legend in Supplementary Fig. **17**.

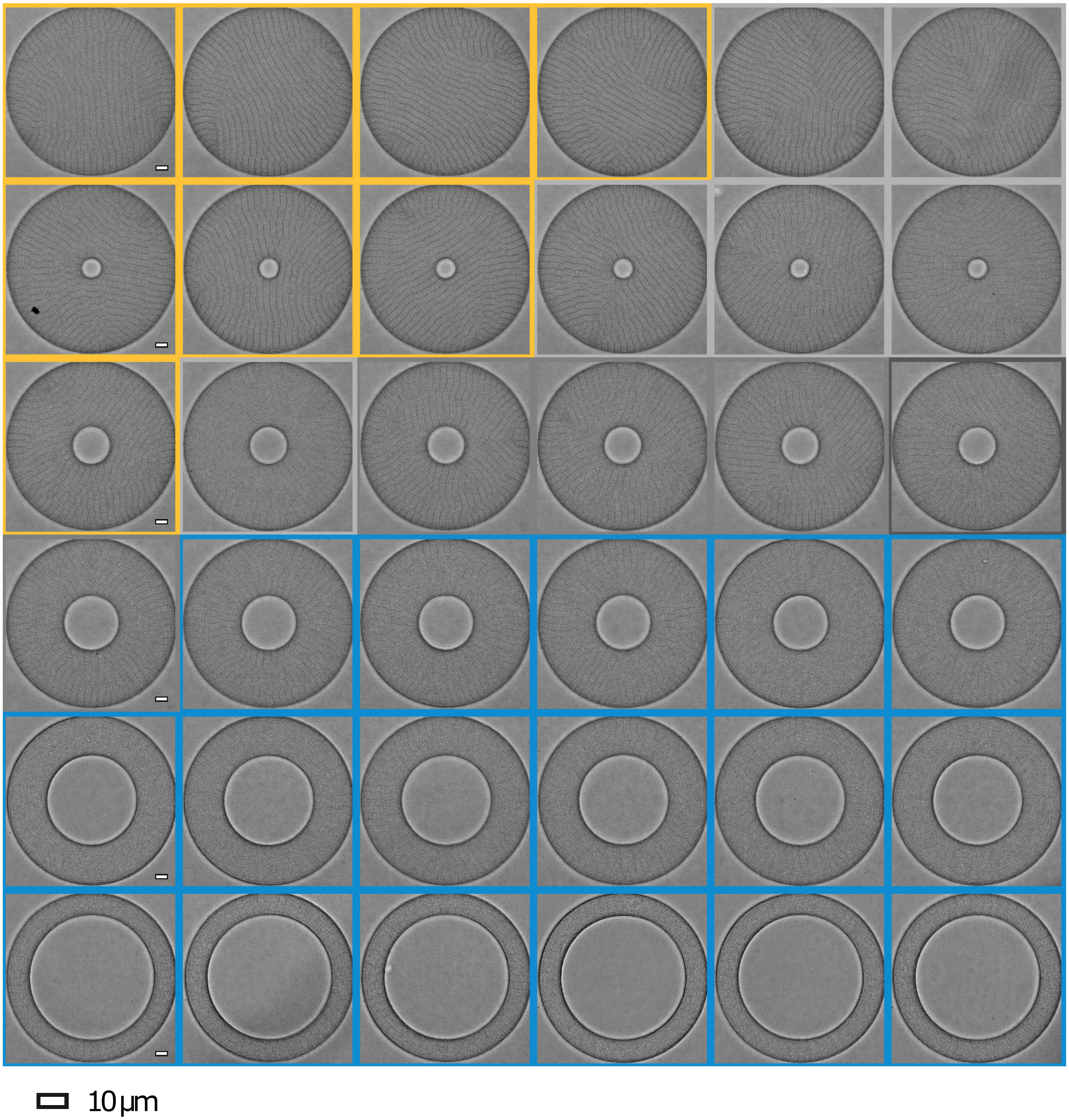

Supplementary Figure 24. Experimental snapshots for $R_{\mathrm{out}} = 15.9L$. The color of the frame indicates the state according to the legend in Supplementary Fig. **16**. Estimated inclusion size ratio from top to bottom: $b = 0$, $b = 0.12$, $b = 0.23$, $b = 0.33$, $b = 0.54$, $b = 0.75$.

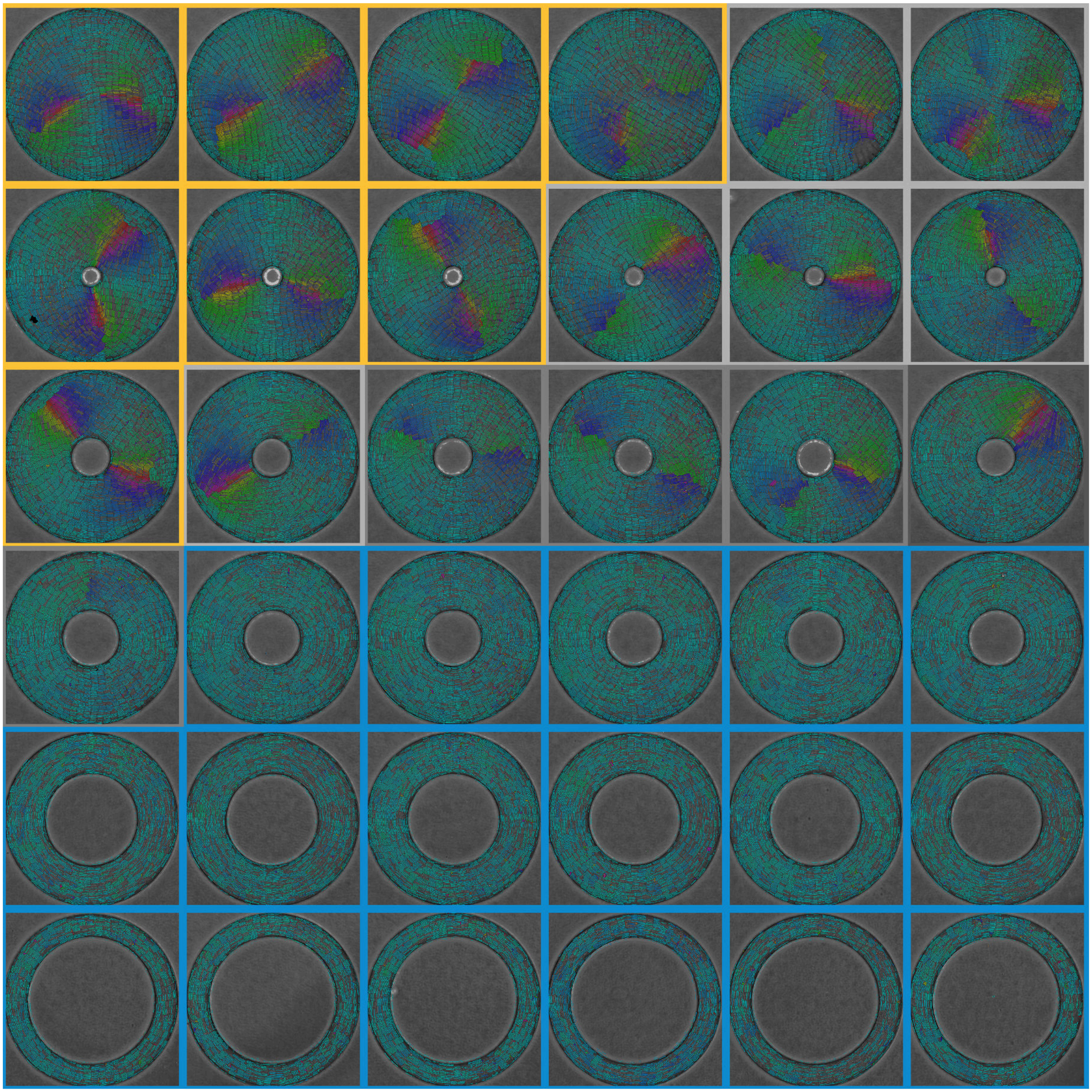

Supplementary Figure 25. As Supplementary Fig. **24** but with additional coloring of single rods indicating their relative orientation to the system boundaries according to the legend in Supplementary Fig. **17**.
