# Supplementary material for: Particle-resolved topological defects of smectic colloidal liquid crystals in extreme confinement
Source: Nat Commun. 2021 Jan 27;12:623. doi: 10.1038/s41467-020-20842-5 (PMC7840983; doi:10.1038/s41467-020-20842-5)
Supplement: Supplementary file 3 — Description of Additional Supplementary Files [file 41467_2020_20842_MOESM3_ESM.pdf]

Description of Additional Supplementary Files

File Name: Supplementary Data 1
Description: Full-size versions of Supplementary Figs. 16 to 25.

File Name: Supplementary Data 2
Description: Collection of raw experimental data and an example for image analysis.

File Name: Supplementary Software 1
Description: Example program to minimize the density functional.

File Name: Supplementary Video 1
Description: Time lapse of the experimental sedimentation process, showing the formation of intermediate isotropic and nematic structures at the bottom of the cavity before the reported smectic states emerge in sedimentation equilibrium.
