# Supplementary material for: Particle-resolved topological defects of smectic colloidal liquid crystals in extreme confinement
Source: Nat Commun. 2021 Jan 27;12:623. doi: 10.1038/s41467-020-20842-5 (PMC7840983; doi:10.1038/s41467-020-20842-5)
Supplement: Supplementary file 4 — Supplementary Data 1 [file 41467_2020_20842_MOESM4_ESM.zip › exp_uncompressed/Fig_16.pdf]

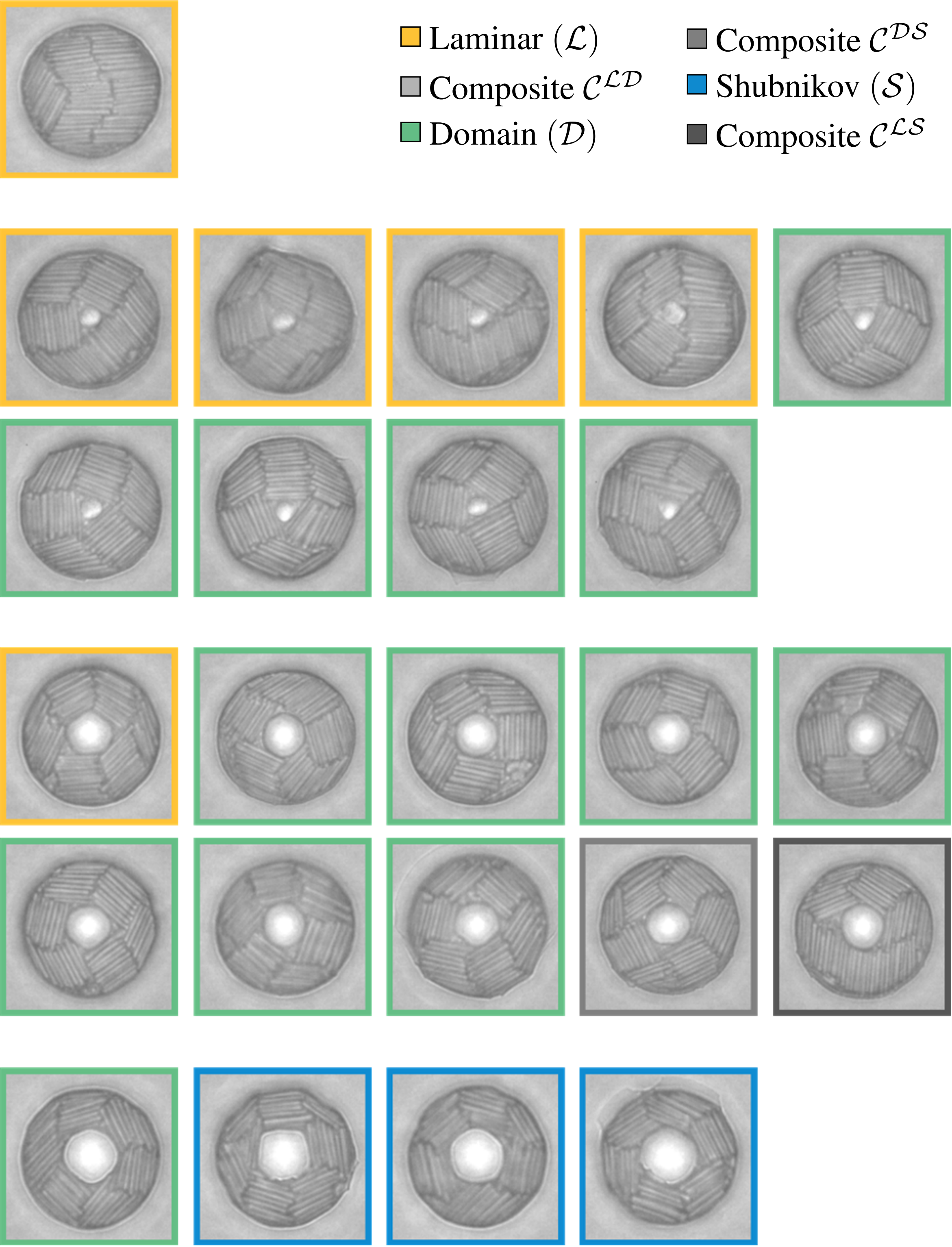

Laminar ($\mathcal{L}$)
Composite $\mathcal{C}^{\mathcal{LD}}$
Domain ($\mathcal{D}$)
Composite $\mathcal{C}^{\mathcal{DS}}$
Shubnikov ($\mathcal{S}$)
Composite $\mathcal{C}^{\mathcal{LS}}$
