# Supplementary figures and images for: Particle-resolved topological defects of smectic colloidal liquid crystals in extreme confinement (part 1 of 2)
Source: Nat Commun. 2021 Jan 27;12:623. doi: 10.1038/s41467-020-20842-5 (PMC7840983; doi:10.1038/s41467-020-20842-5)

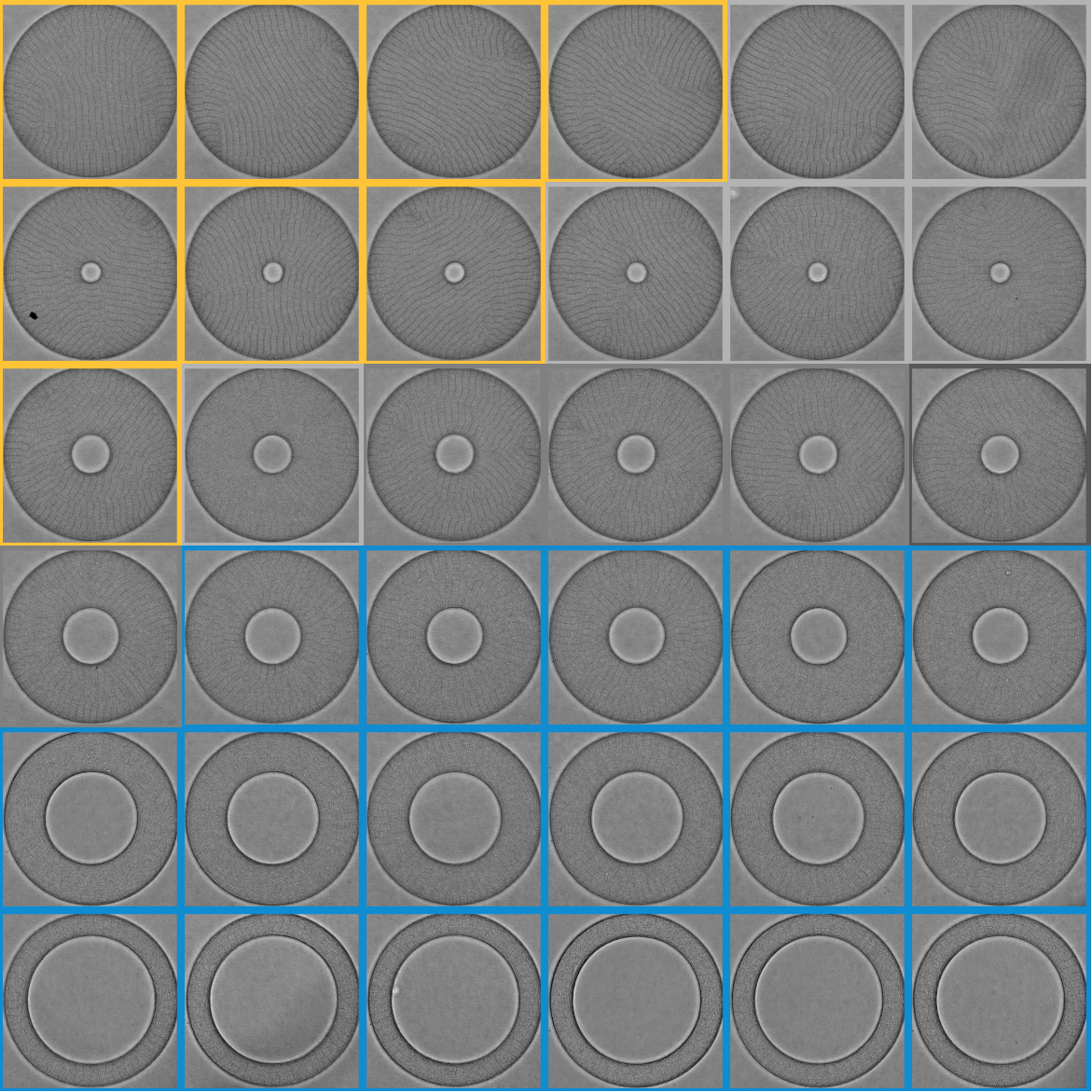

Supplement: Supplementary file 4 — Supplementary Data 1 [file 41467_2020_20842_MOESM4_ESM.zip › exp_uncompressed/Fig_24.pdf]

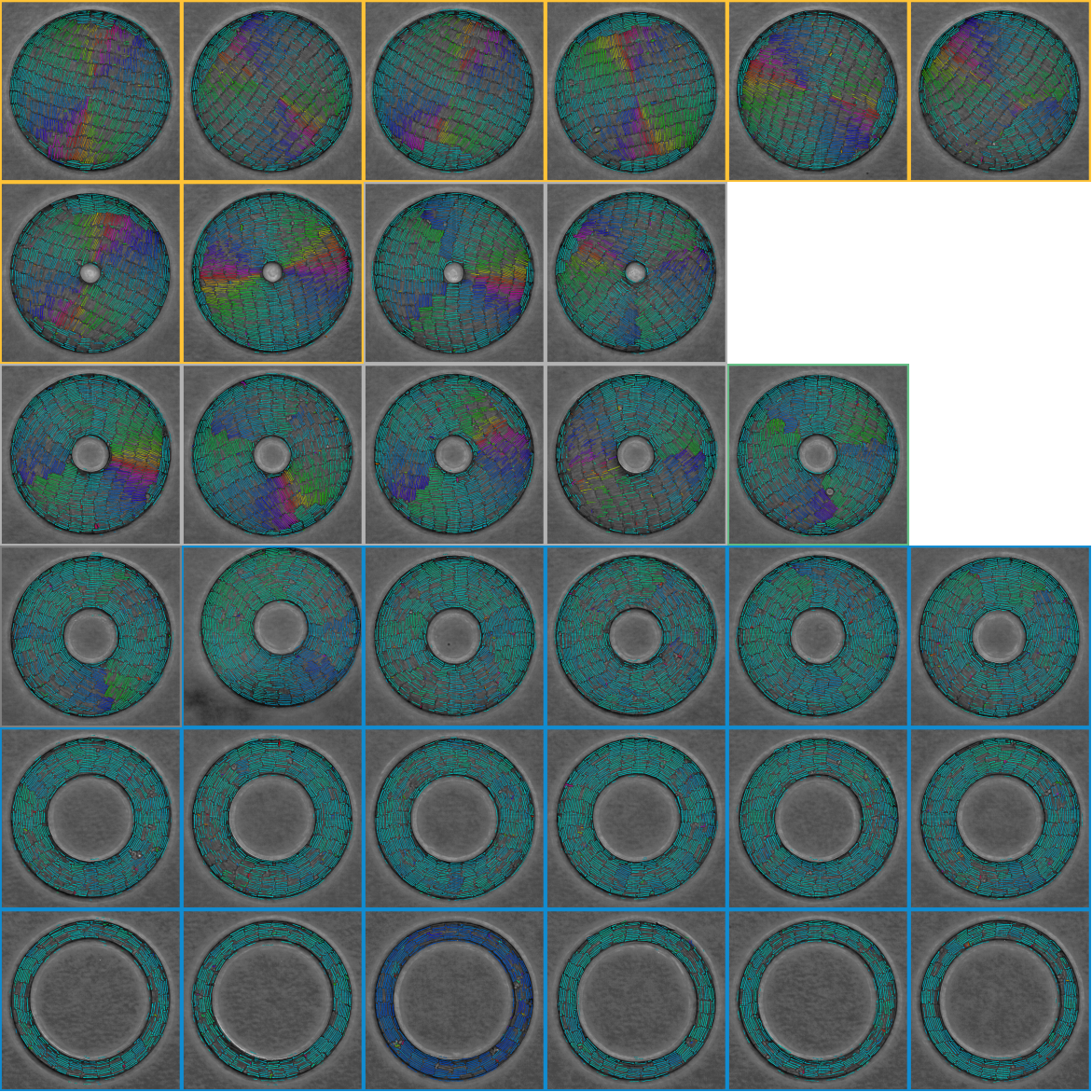

Supplement: Supplementary file 4 — Supplementary Data 1 [file 41467_2020_20842_MOESM4_ESM.zip › exp_uncompressed/Fig_23.pdf]

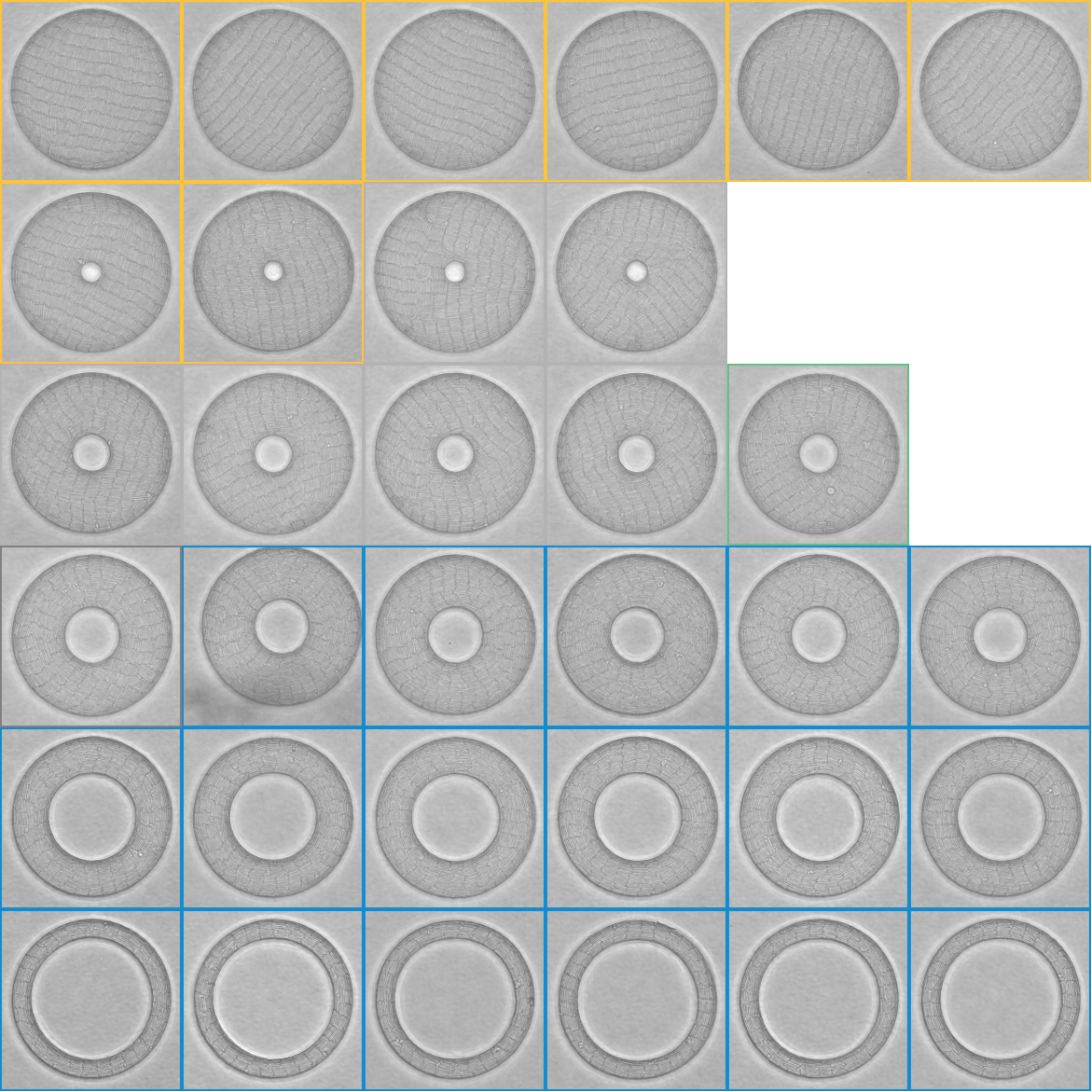

Supplement: Supplementary file 4 — Supplementary Data 1 [file 41467_2020_20842_MOESM4_ESM.zip › exp_uncompressed/Fig_22.pdf]

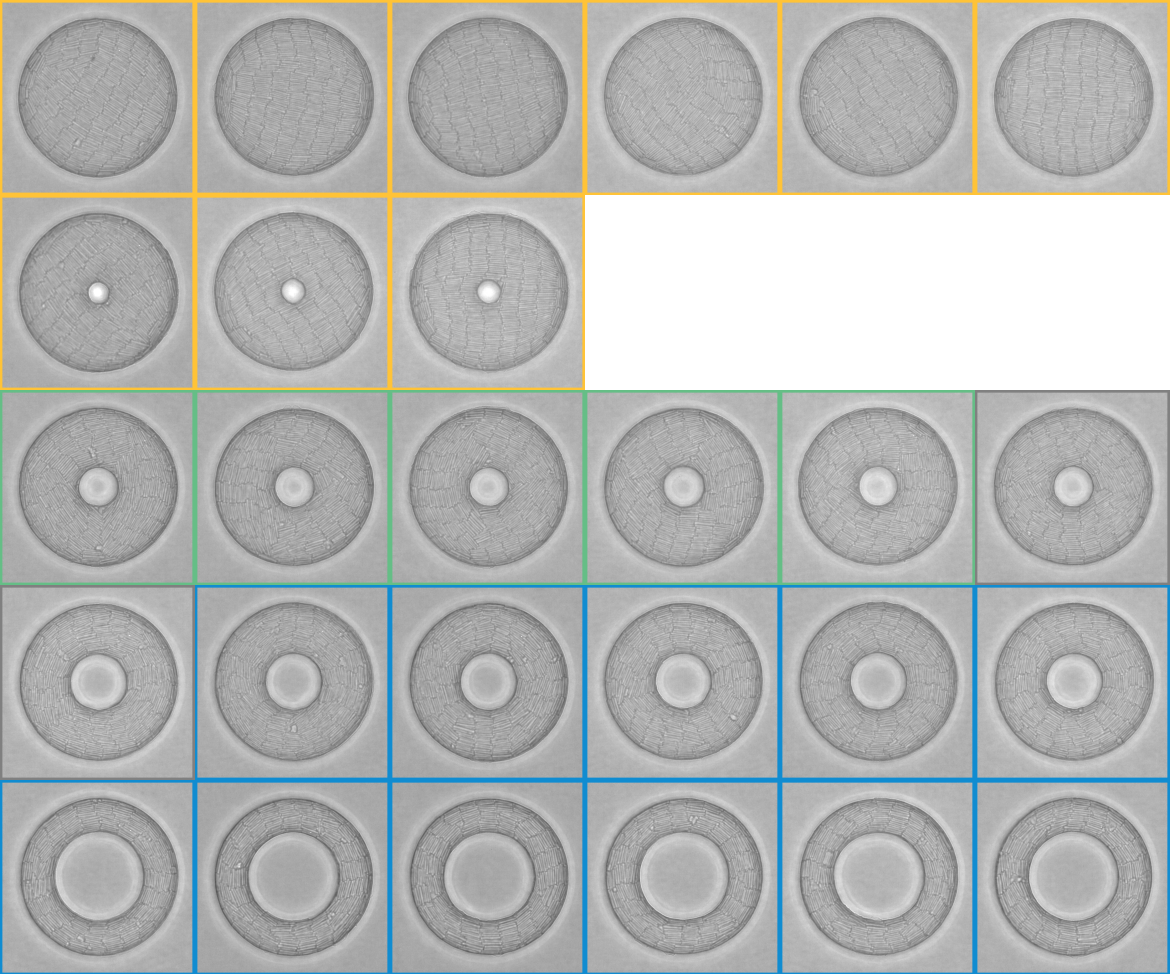

Supplement: Supplementary file 4 — Supplementary Data 1 [file 41467_2020_20842_MOESM4_ESM.zip › exp_uncompressed/Fig_20.pdf]

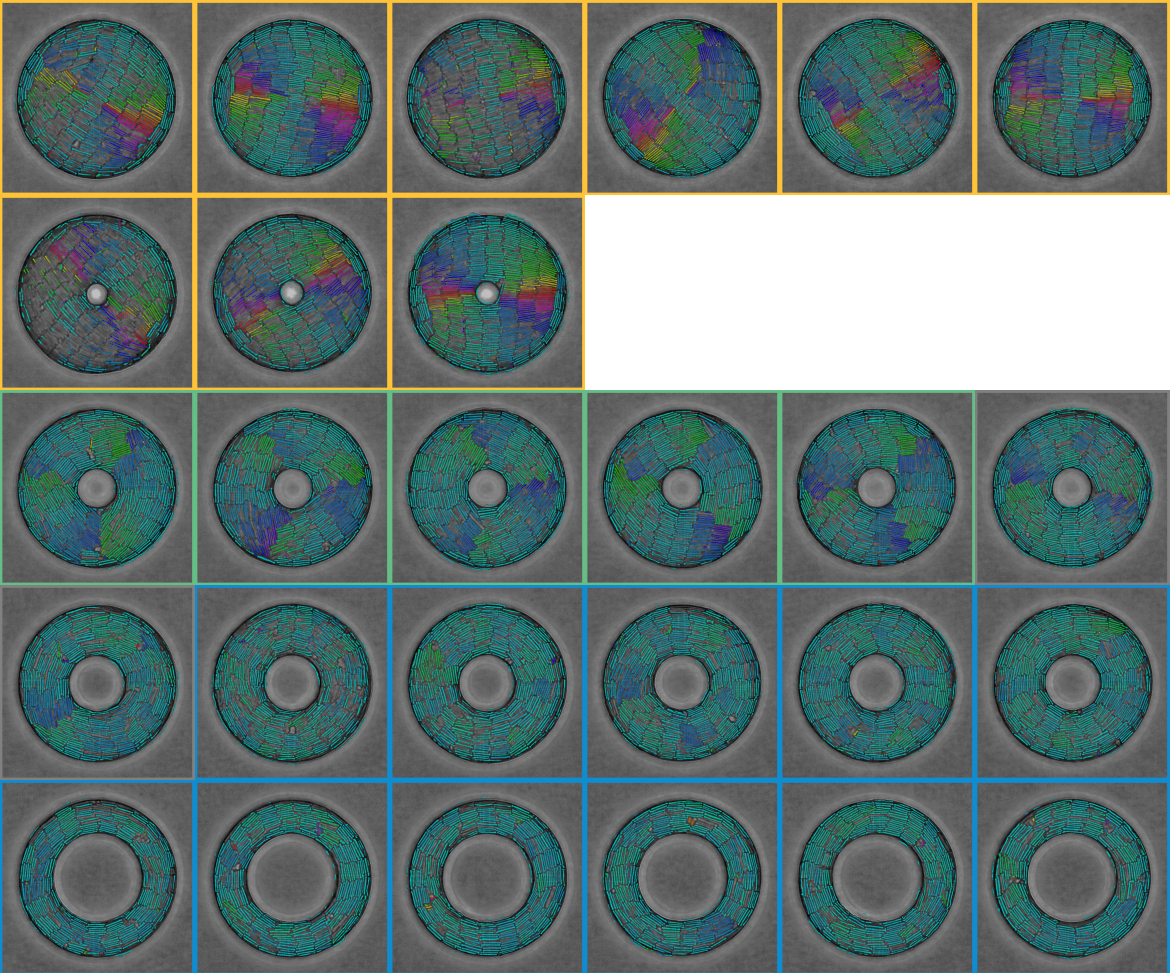

Supplement: Supplementary file 4 — Supplementary Data 1 [file 41467_2020_20842_MOESM4_ESM.zip › exp_uncompressed/Fig_21.pdf]

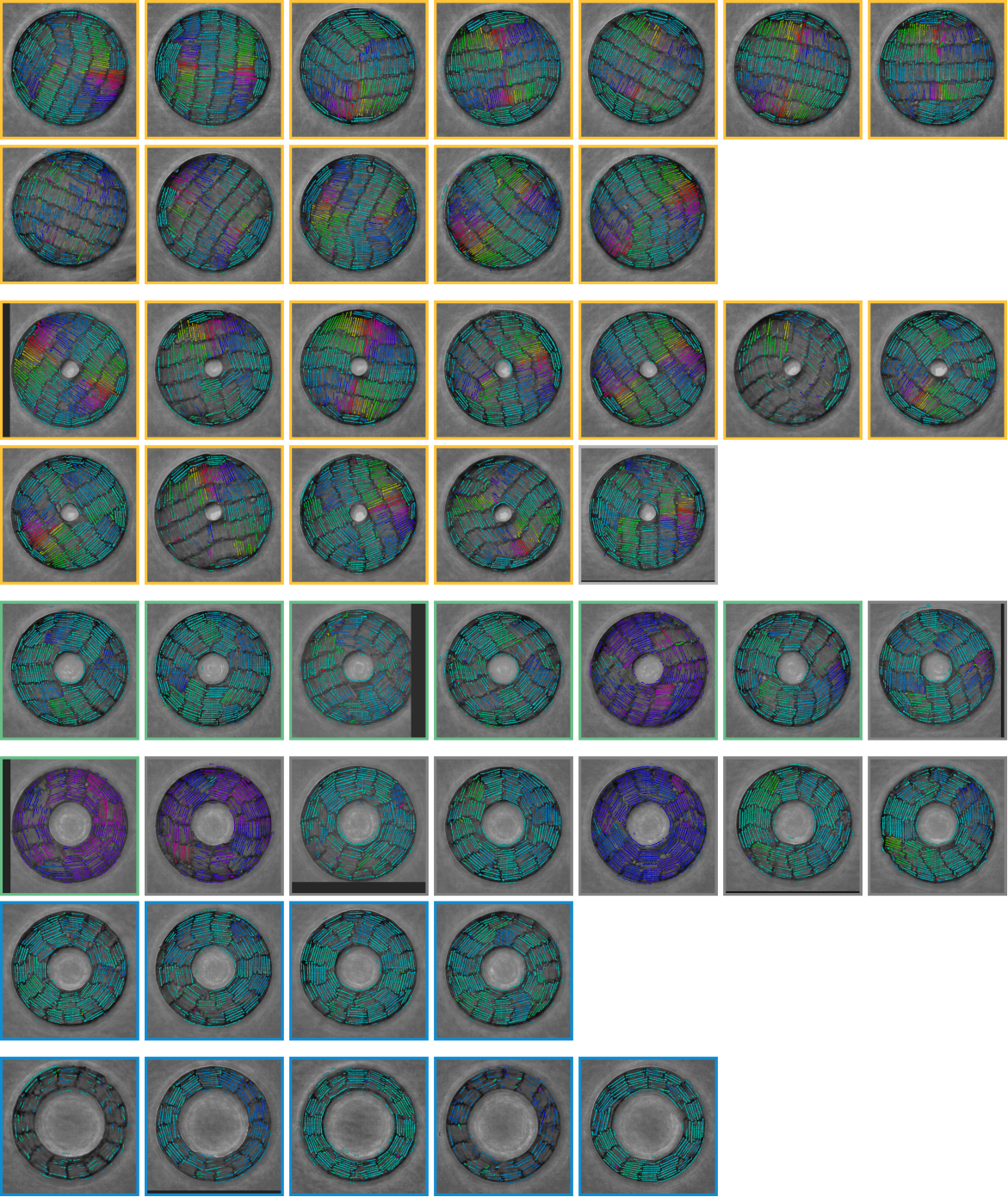

Supplement: Supplementary file 4 — Supplementary Data 1 [file 41467_2020_20842_MOESM4_ESM.zip › exp_uncompressed/Fig_19.pdf]

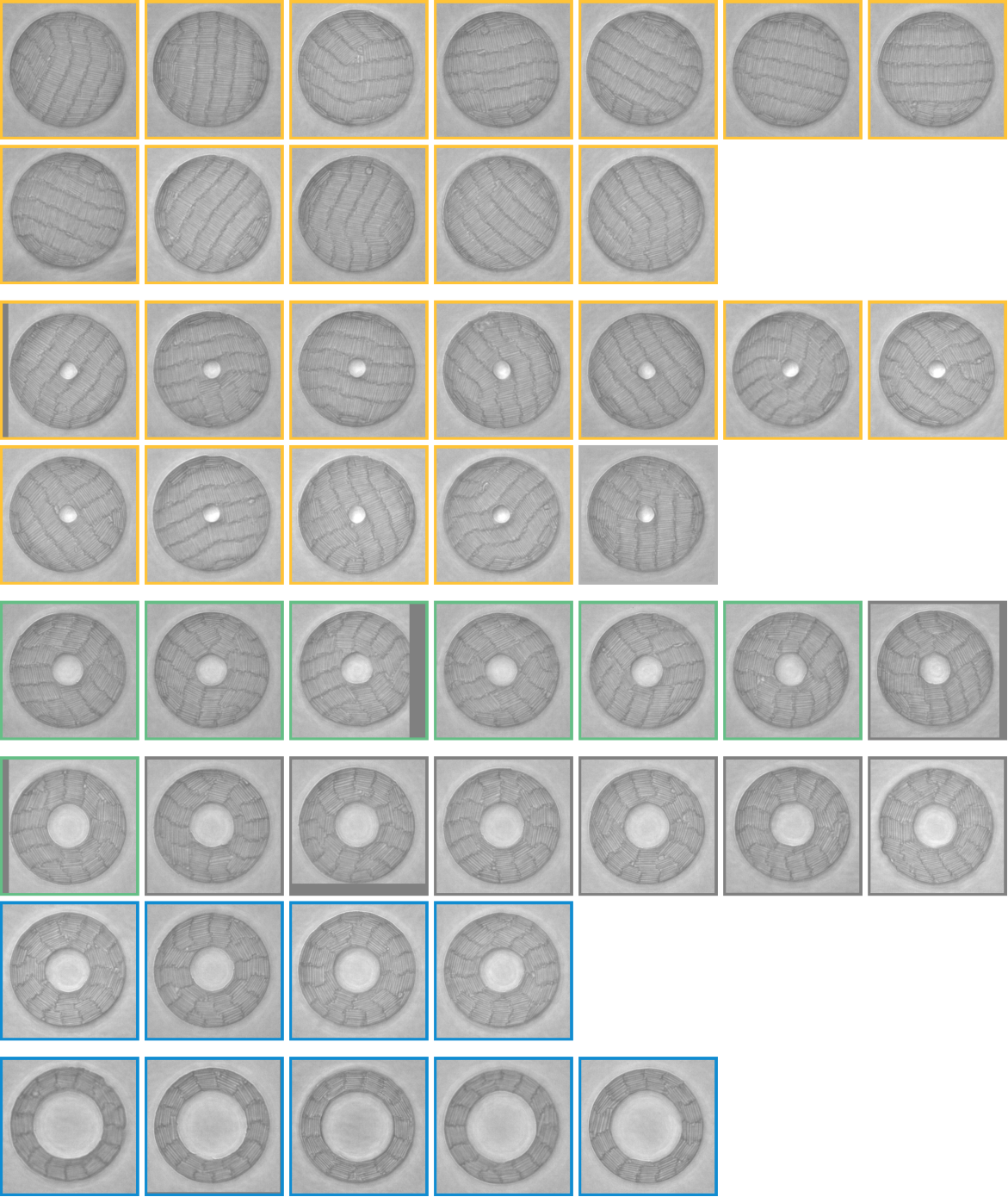

Supplement: Supplementary file 4 — Supplementary Data 1 [file 41467_2020_20842_MOESM4_ESM.zip › exp_uncompressed/Fig_18.pdf]

orientation relative to the wall

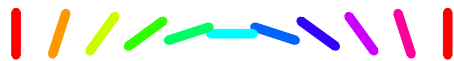

$\frac{\pi}{2}$

$\frac{\pi}{4}$

0

$-\frac{\pi}{4}$

$-\frac{\pi}{2}$

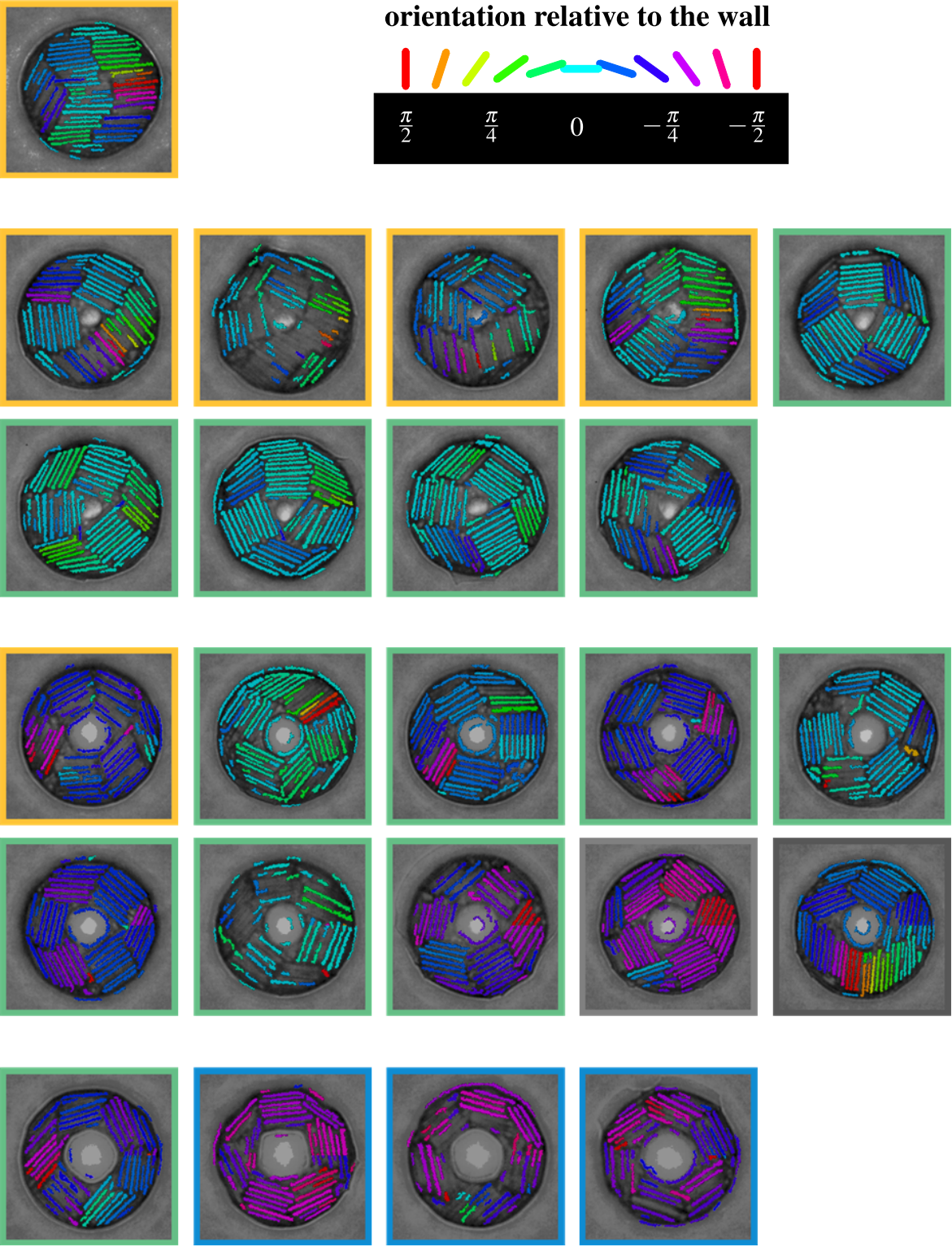

Supplement: Supplementary file 4 — Supplementary Data 1 [file 41467_2020_20842_MOESM4_ESM.zip › exp_uncompressed/Fig_17.pdf]

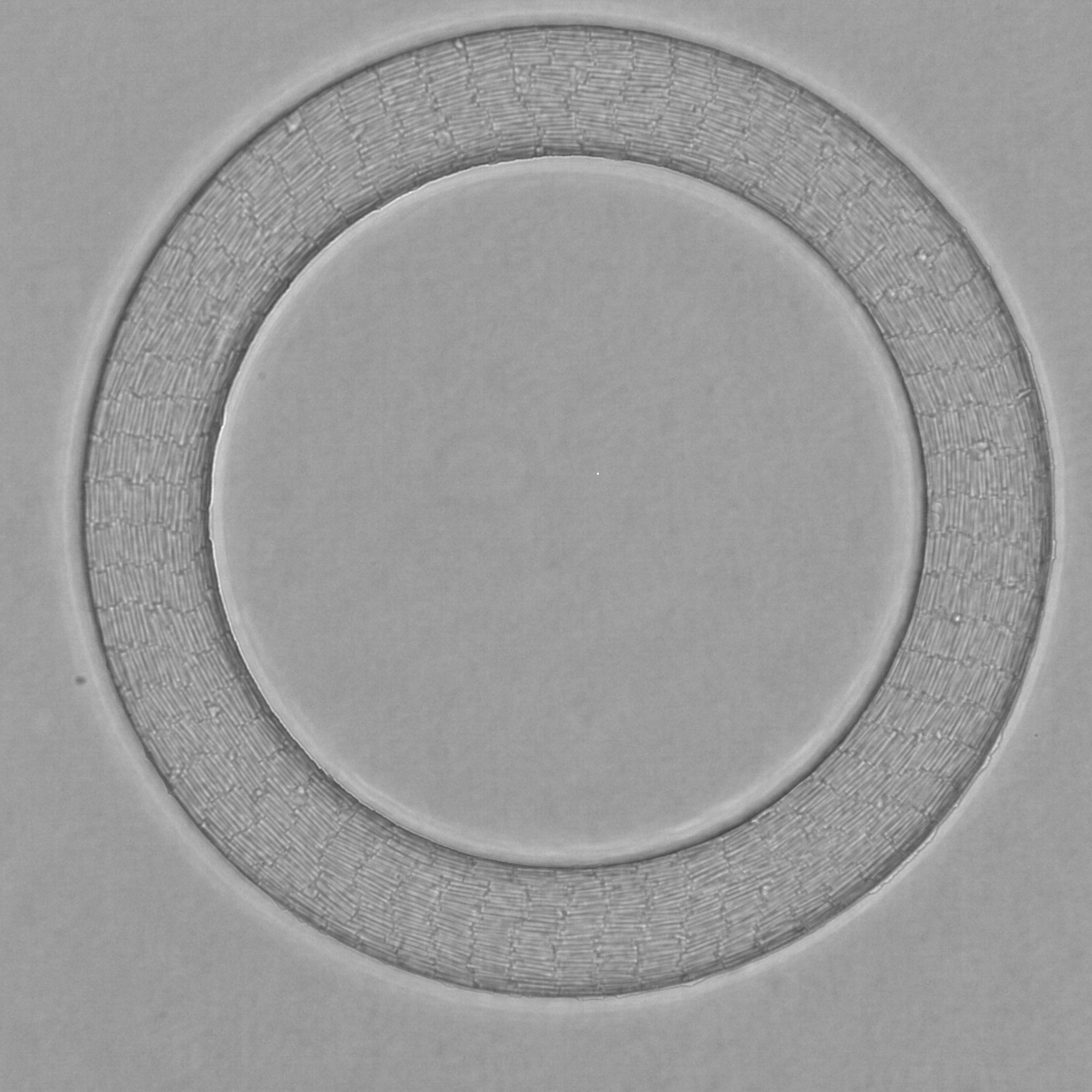

Supplement: Supplementary file 5 — Supplementary Data 2 [file 41467_2020_20842_MOESM5_ESM.zip › rawdata/size6/06_06.tif]

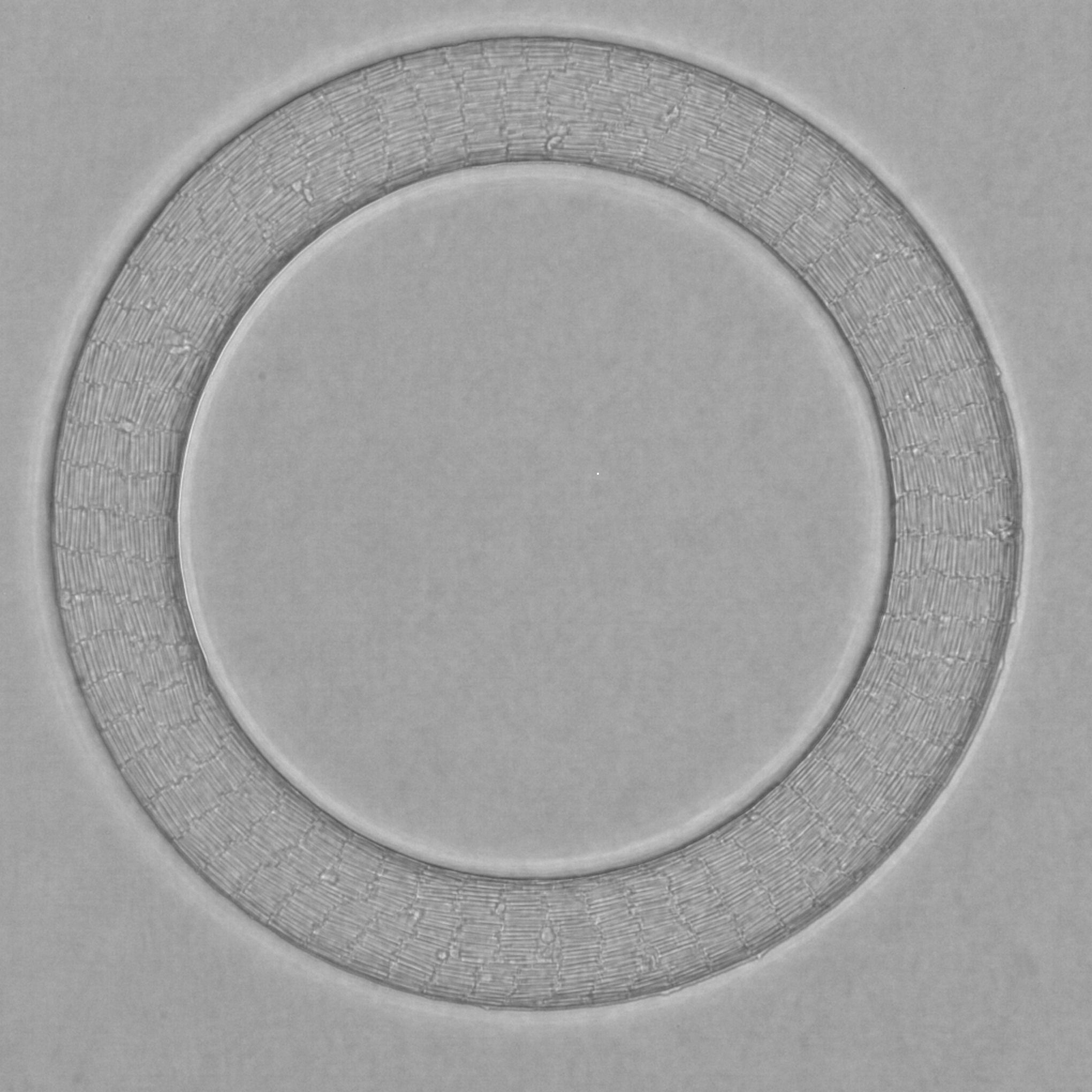

Supplement: Supplementary file 5 — Supplementary Data 2 [file 41467_2020_20842_MOESM5_ESM.zip › rawdata/size6/06_05.tif]

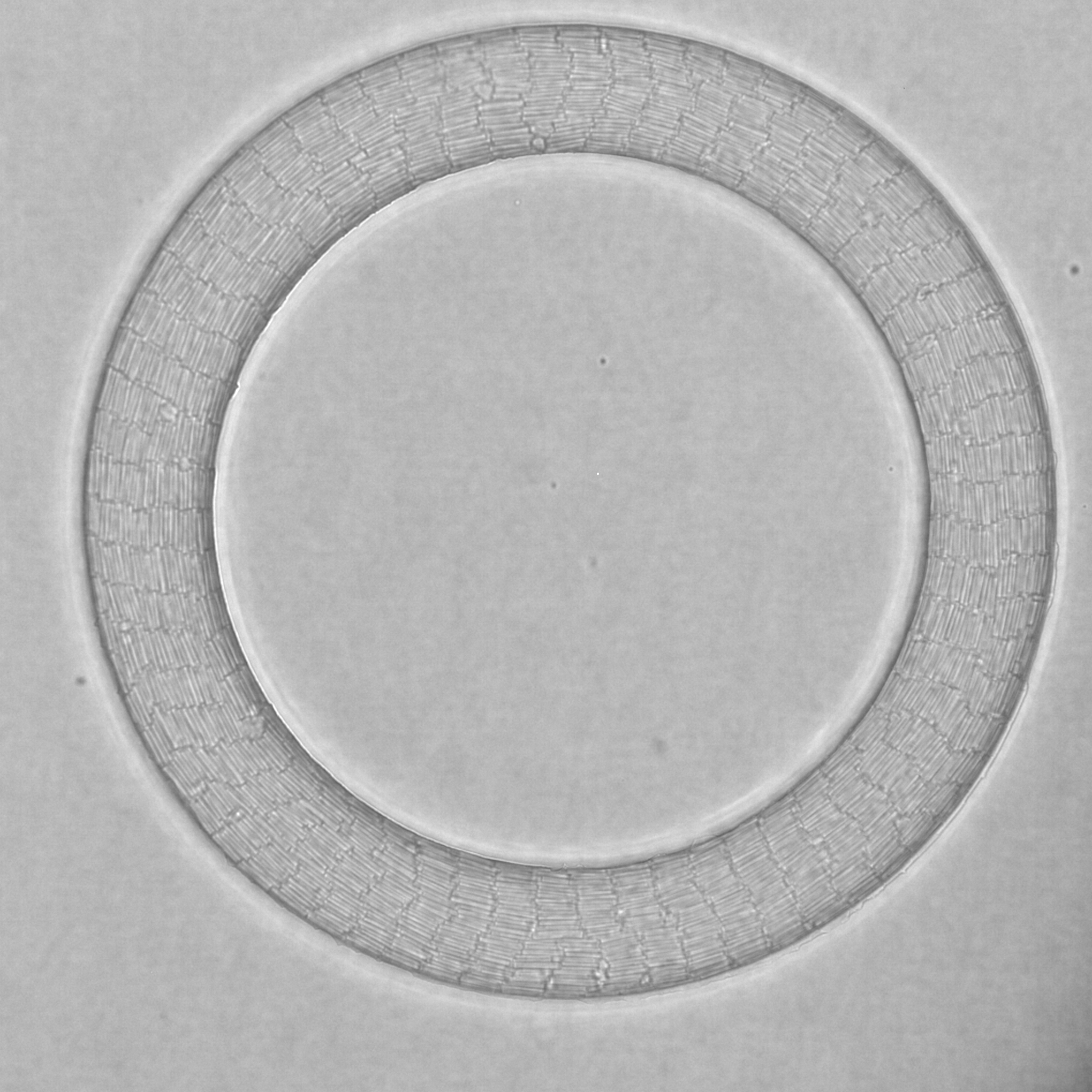

Supplement: Supplementary file 5 — Supplementary Data 2 [file 41467_2020_20842_MOESM5_ESM.zip › rawdata/size6/06_04.tif]

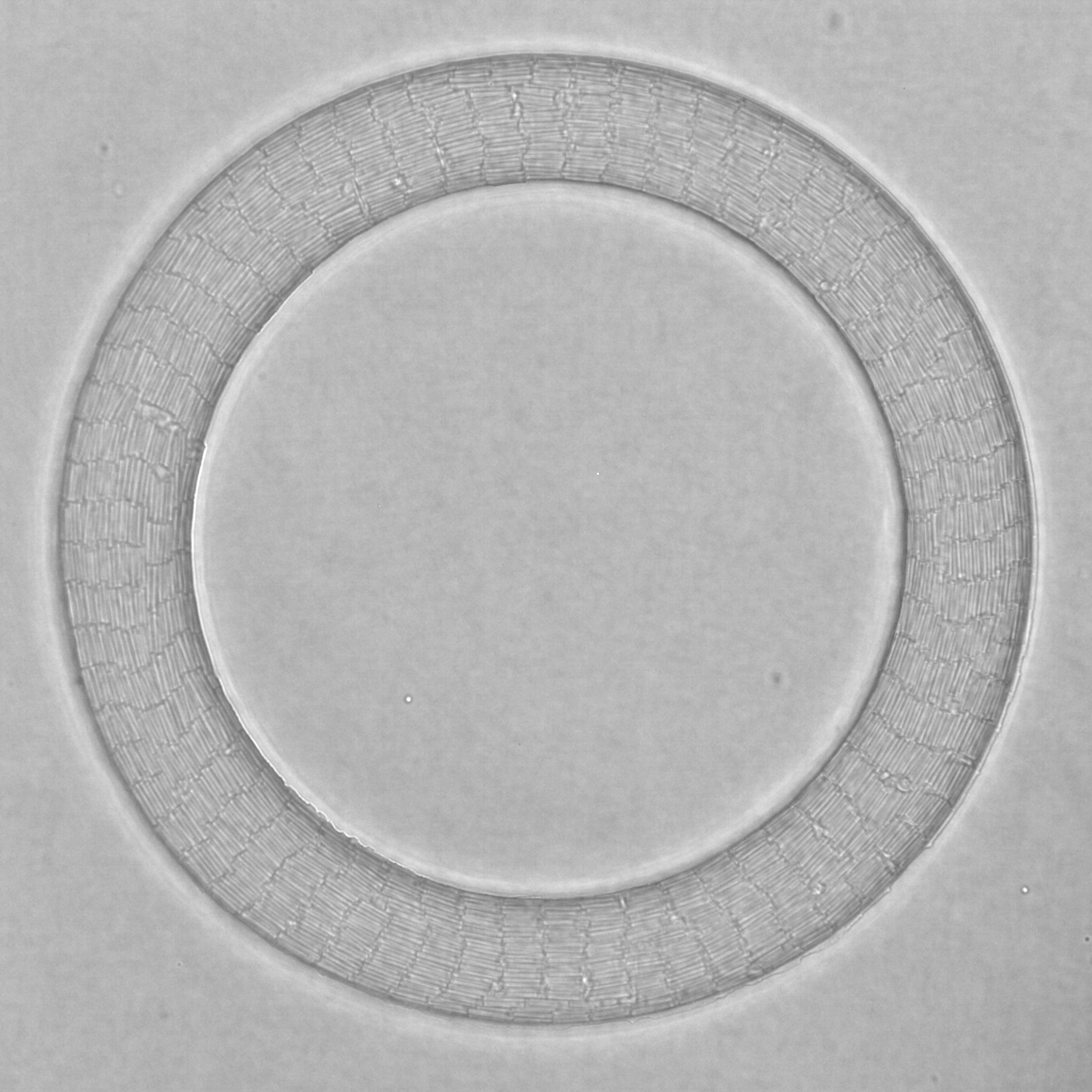

Supplement: Supplementary file 5 — Supplementary Data 2 [file 41467_2020_20842_MOESM5_ESM.zip › rawdata/size6/06_03.tif]

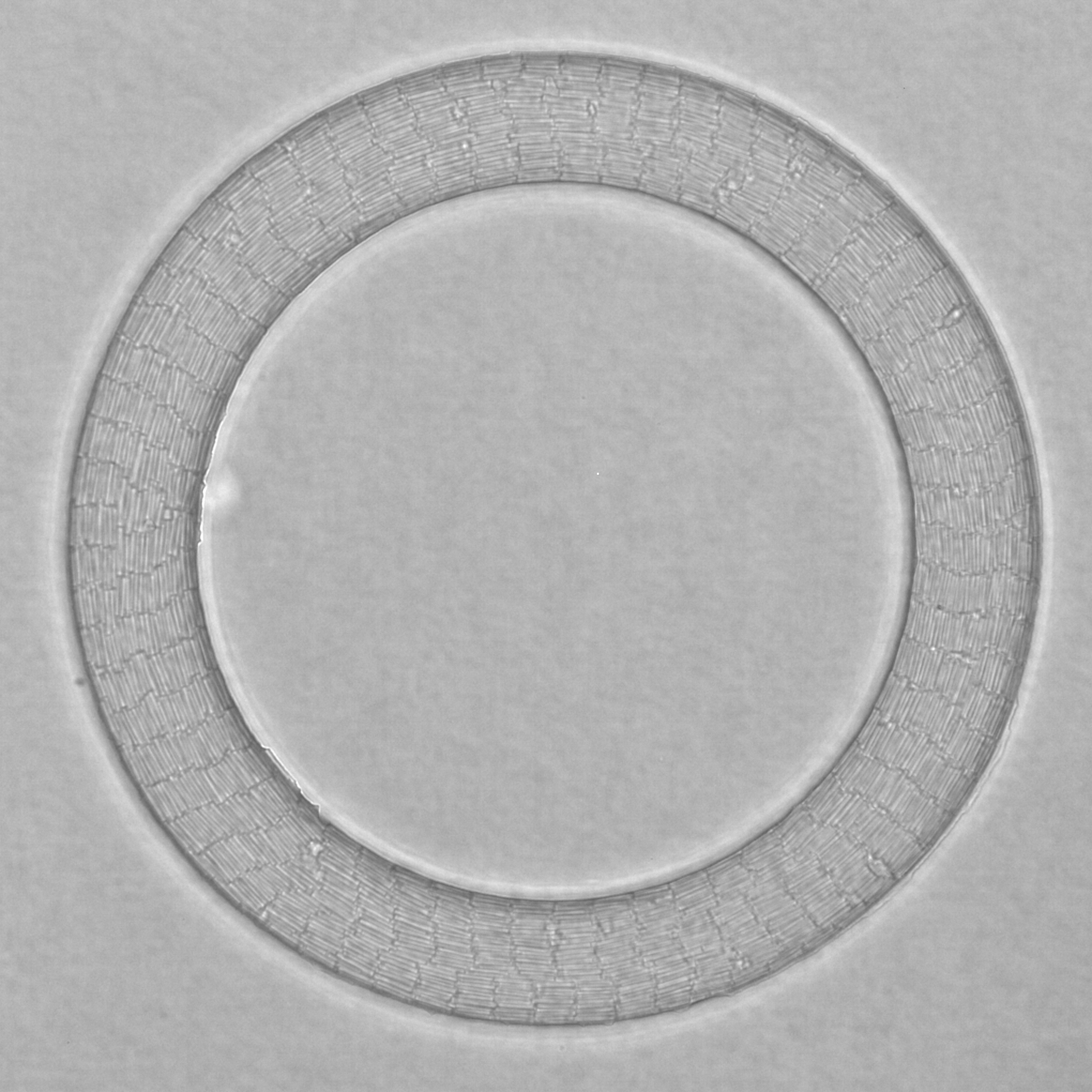

Supplement: Supplementary file 5 — Supplementary Data 2 [file 41467_2020_20842_MOESM5_ESM.zip › rawdata/size6/06_02.tif]

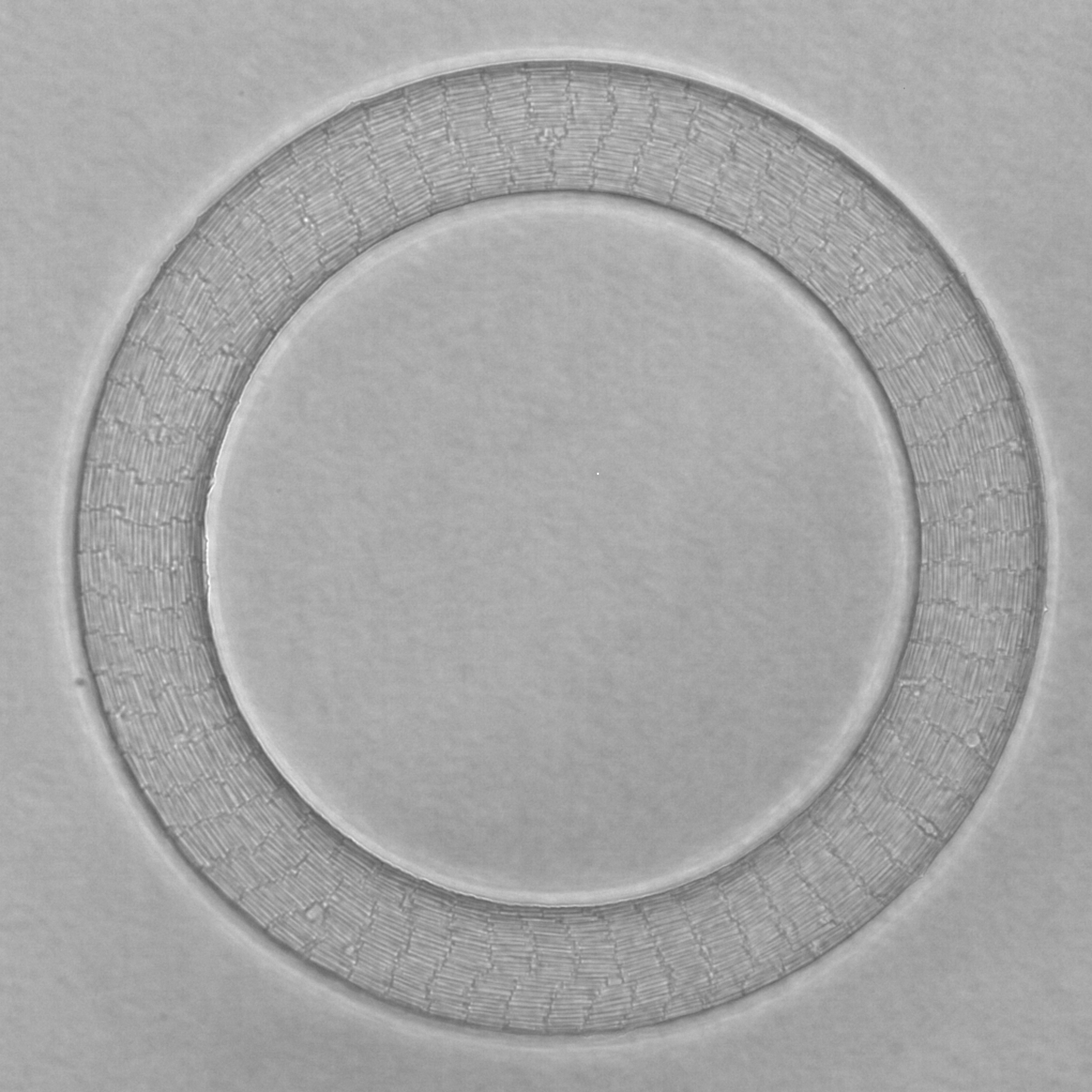

Supplement: Supplementary file 5 — Supplementary Data 2 [file 41467_2020_20842_MOESM5_ESM.zip › rawdata/size6/06_01.tif]

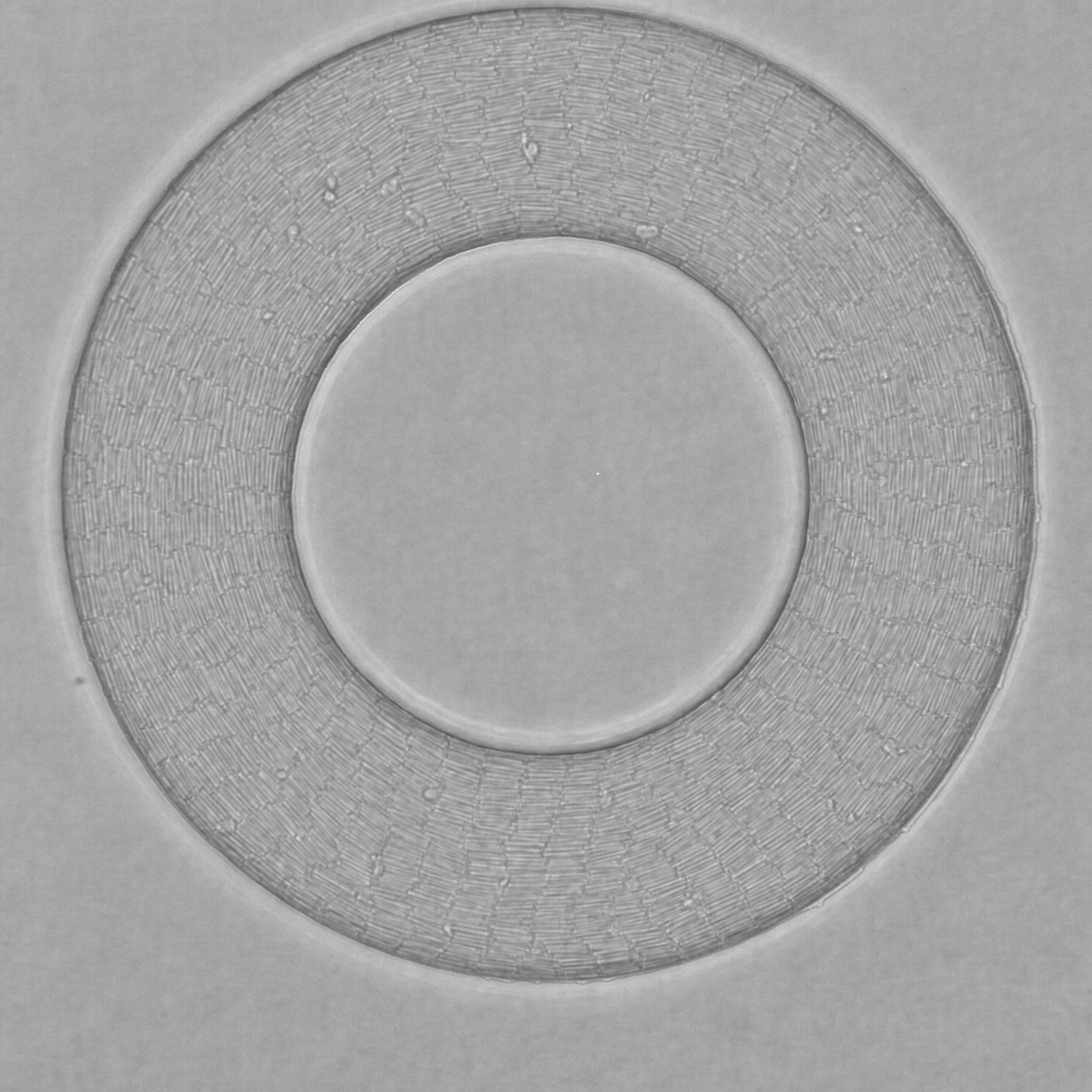

Supplement: Supplementary file 5 — Supplementary Data 2 [file 41467_2020_20842_MOESM5_ESM.zip › rawdata/size6/05_06.tif]

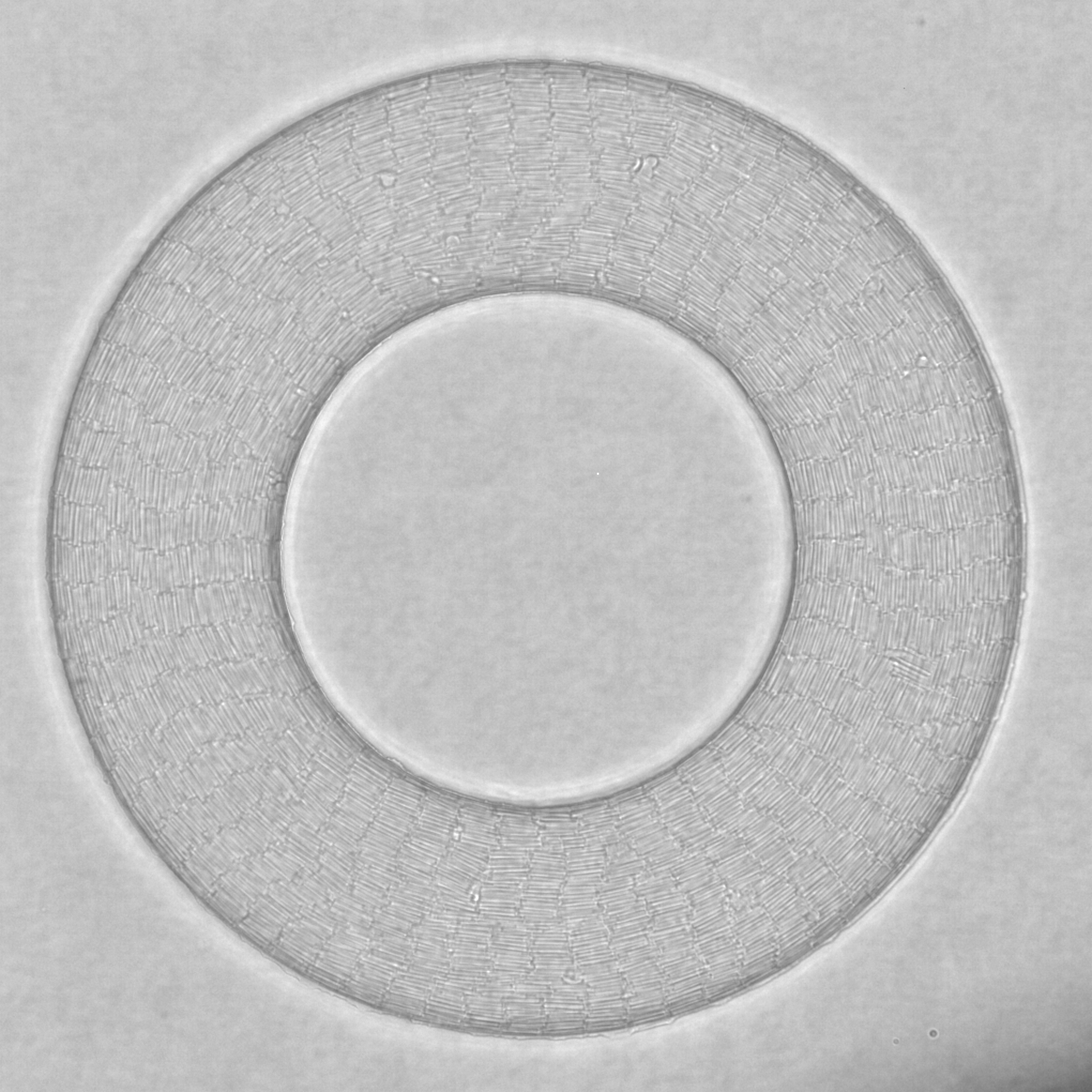

Supplement: Supplementary file 5 — Supplementary Data 2 [file 41467_2020_20842_MOESM5_ESM.zip › rawdata/size6/05_05.tif]

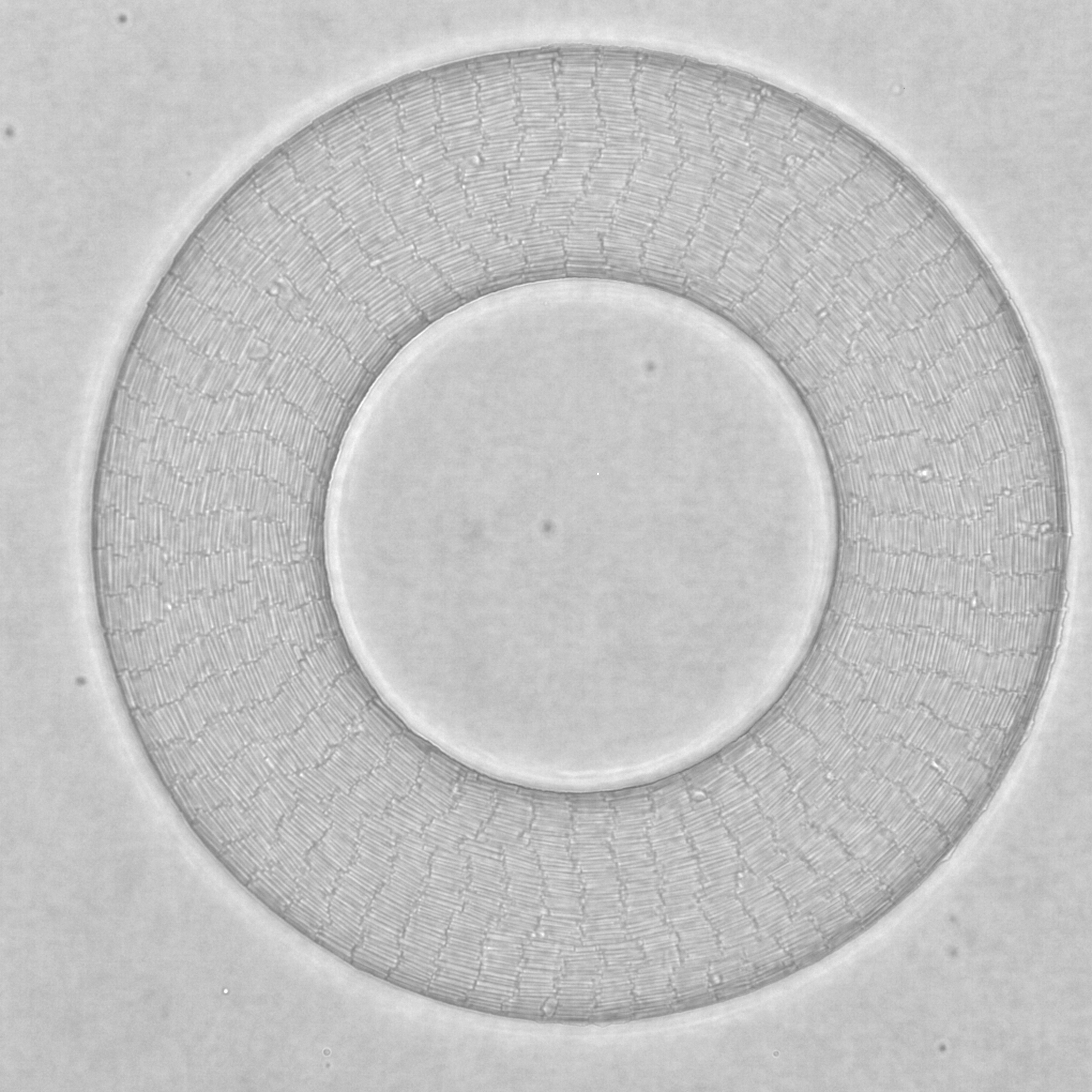

Supplement: Supplementary file 5 — Supplementary Data 2 [file 41467_2020_20842_MOESM5_ESM.zip › rawdata/size6/05_04.tif]

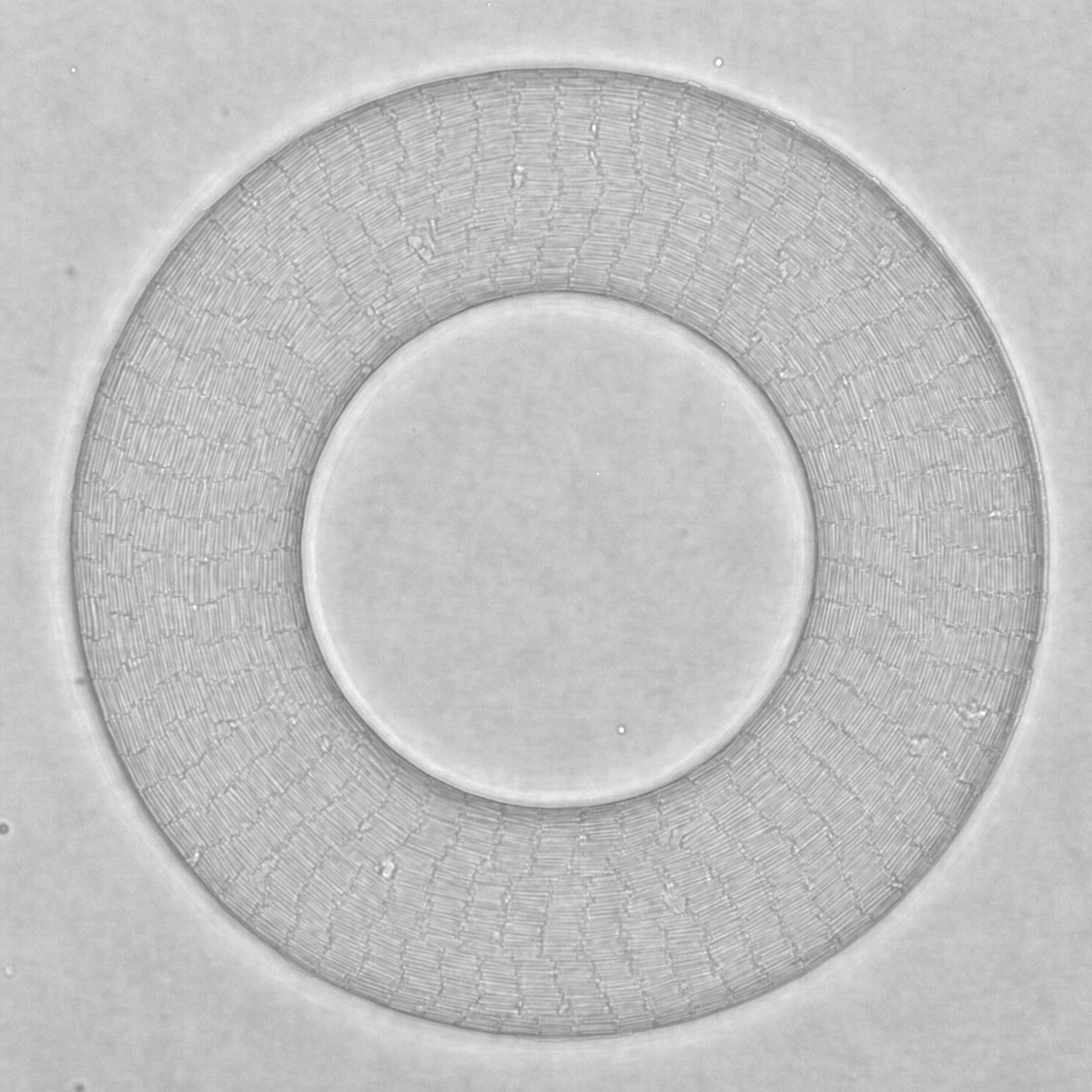

Supplement: Supplementary file 5 — Supplementary Data 2 [file 41467_2020_20842_MOESM5_ESM.zip › rawdata/size6/05_03.tif]

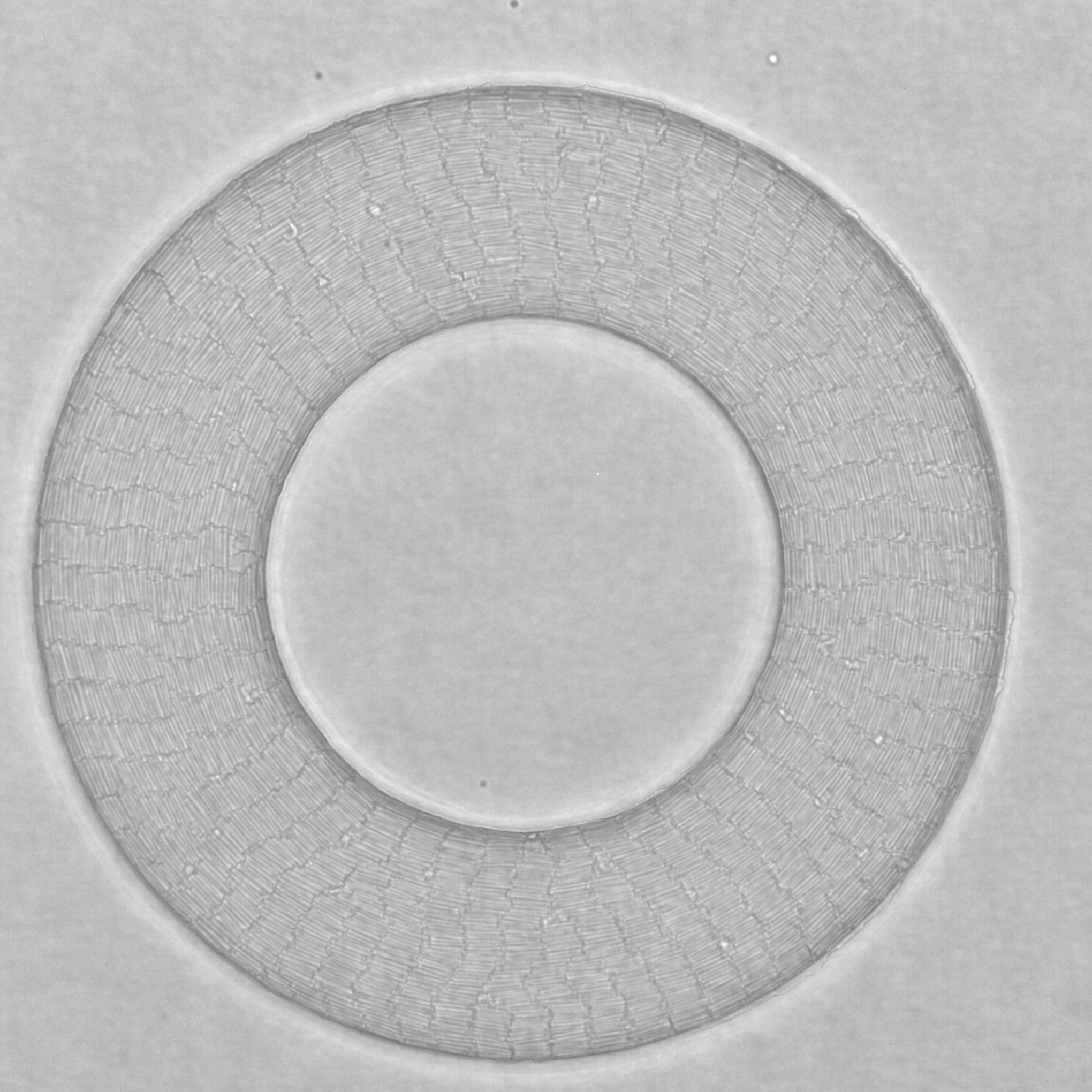

Supplement: Supplementary file 5 — Supplementary Data 2 [file 41467_2020_20842_MOESM5_ESM.zip › rawdata/size6/05_02.tif]

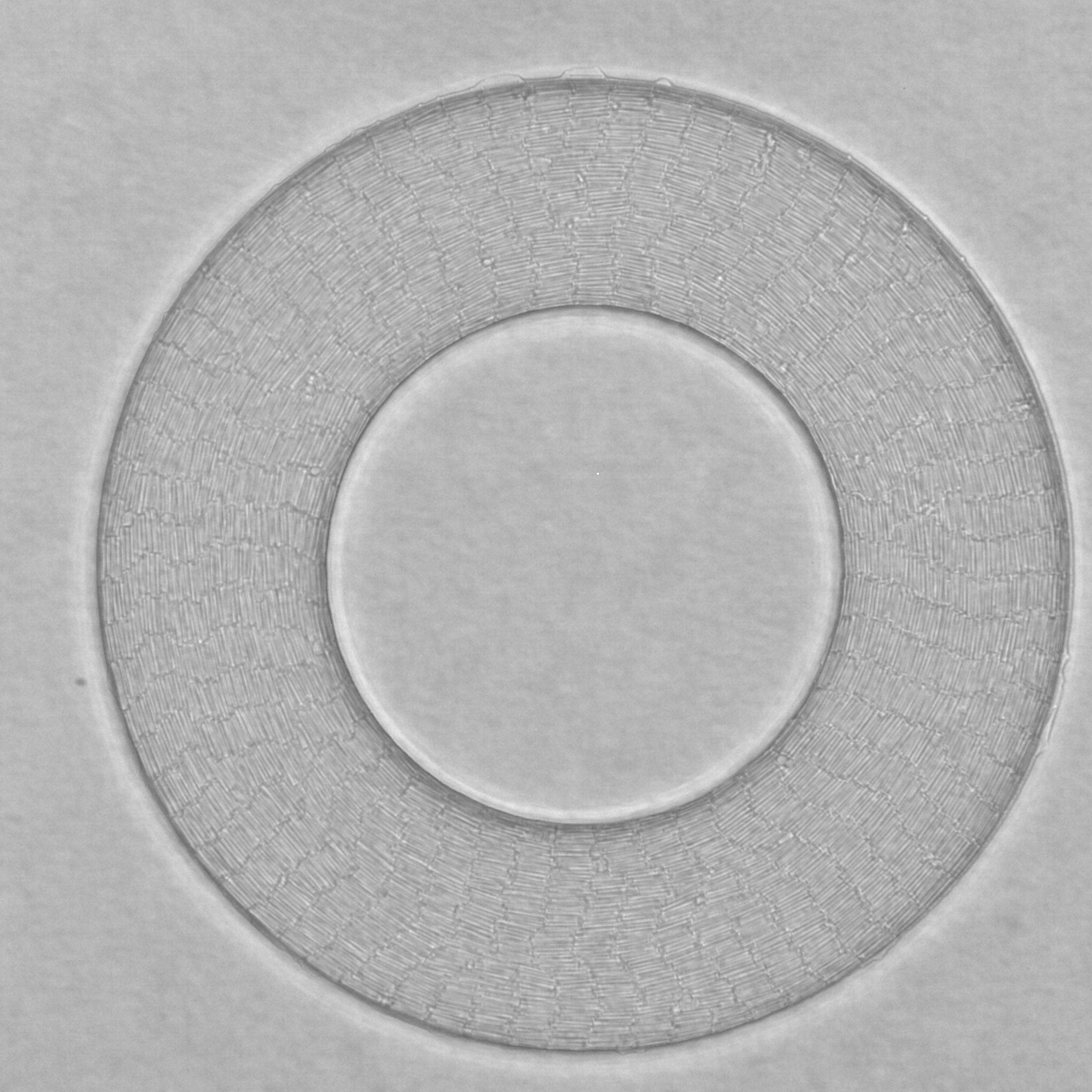

Supplement: Supplementary file 5 — Supplementary Data 2 [file 41467_2020_20842_MOESM5_ESM.zip › rawdata/size6/05_01.tif]

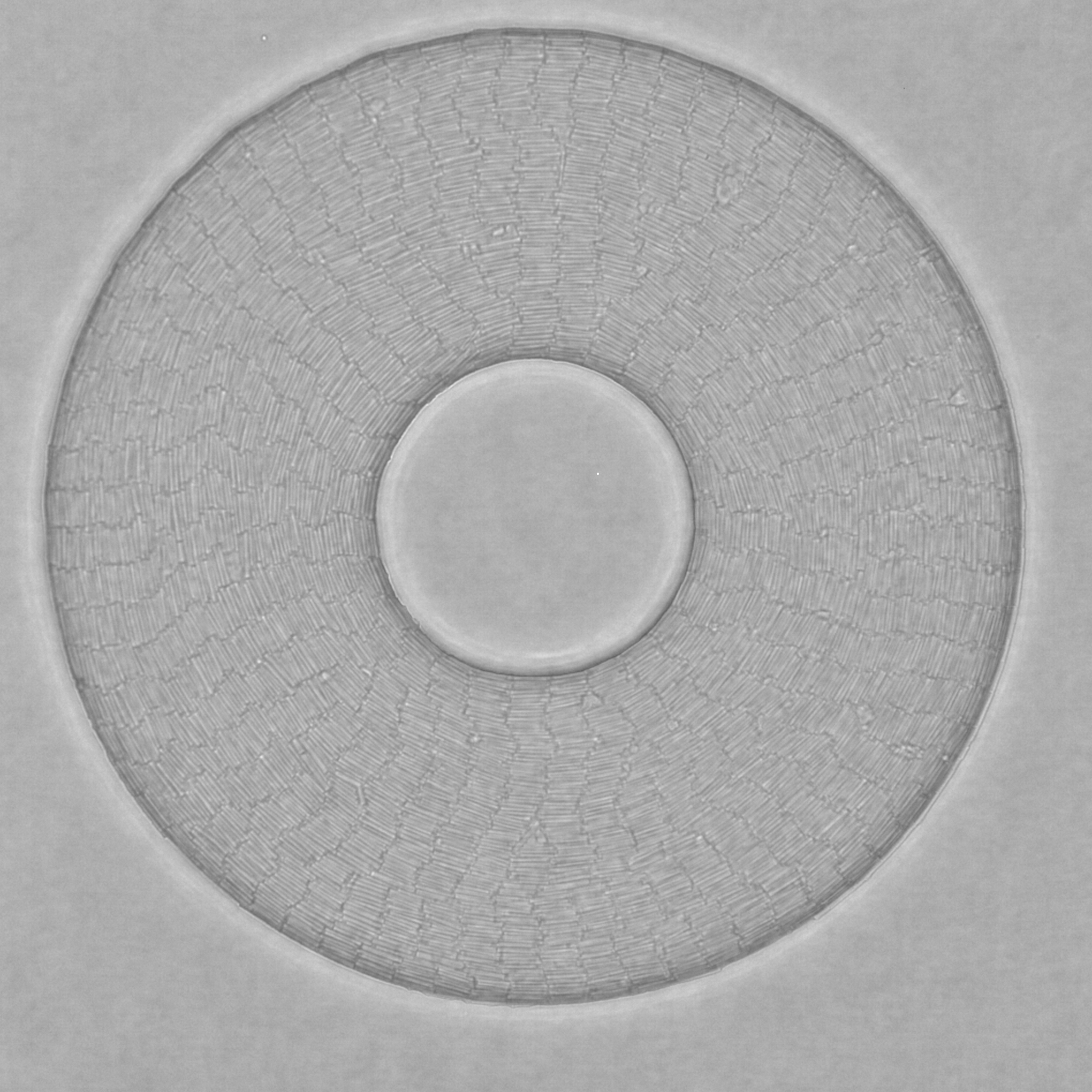

Supplement: Supplementary file 5 — Supplementary Data 2 [file 41467_2020_20842_MOESM5_ESM.zip › rawdata/size6/04_06.tif]

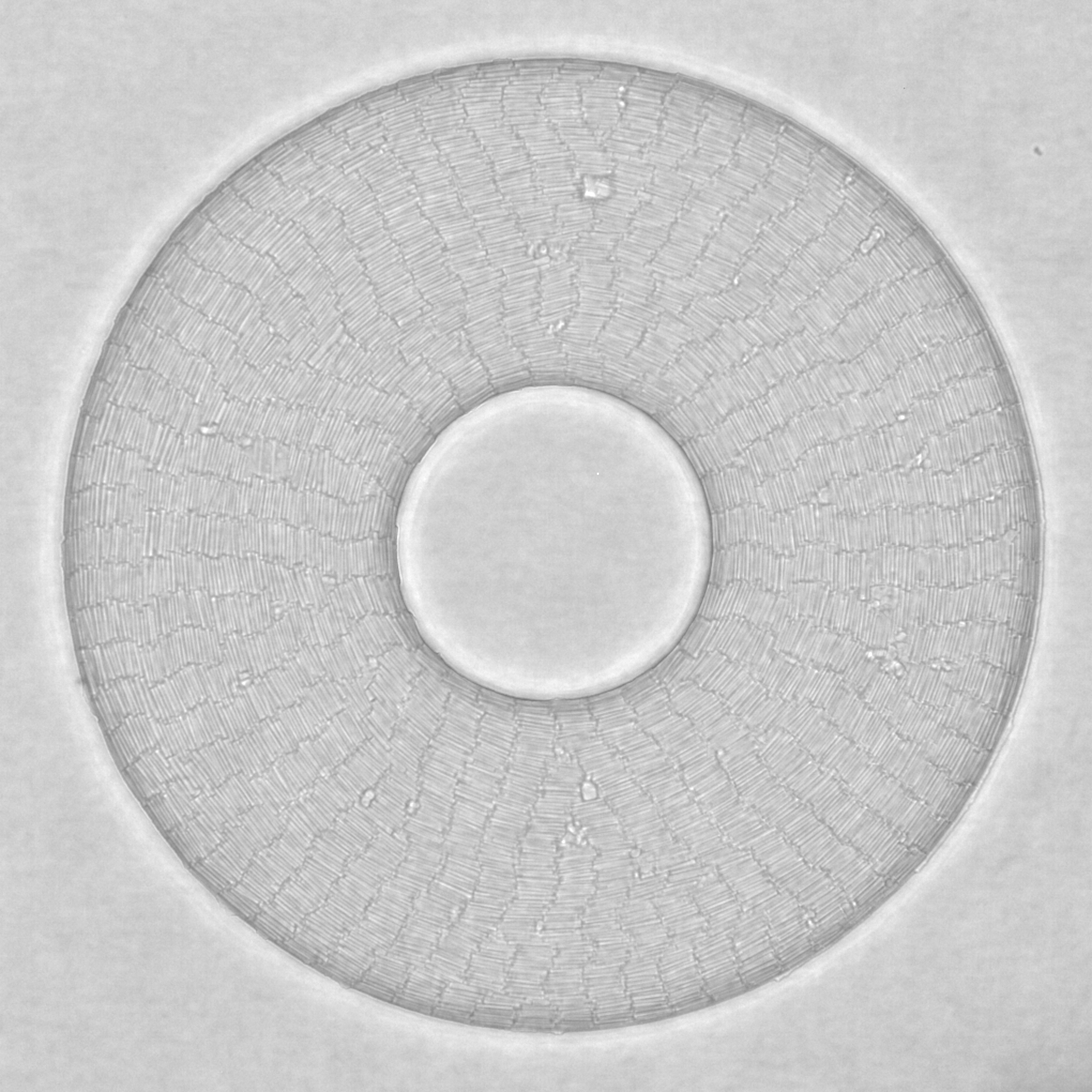

Supplement: Supplementary file 5 — Supplementary Data 2 [file 41467_2020_20842_MOESM5_ESM.zip › rawdata/size6/04_05.tif]

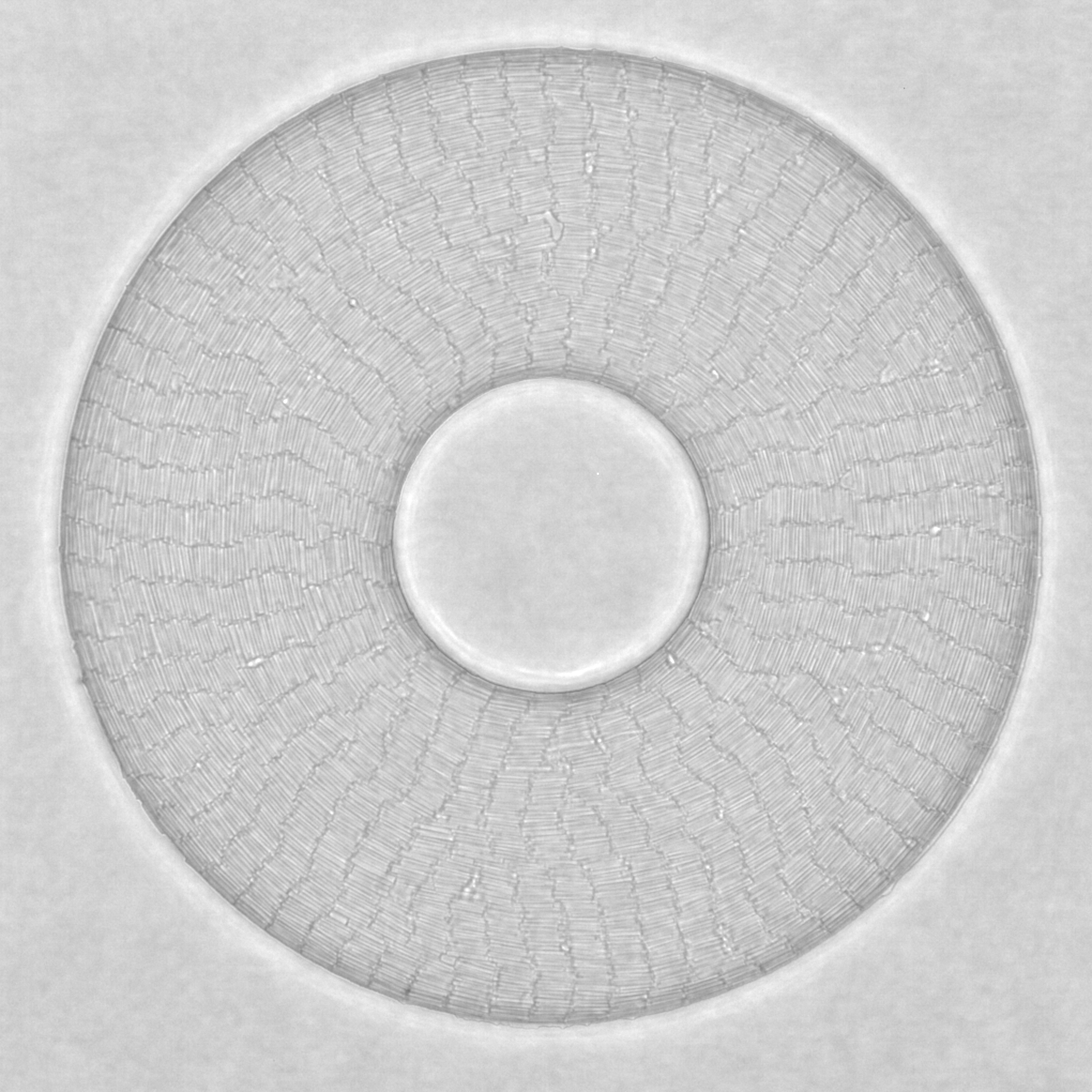

Supplement: Supplementary file 5 — Supplementary Data 2 [file 41467_2020_20842_MOESM5_ESM.zip › rawdata/size6/04_04.tif]

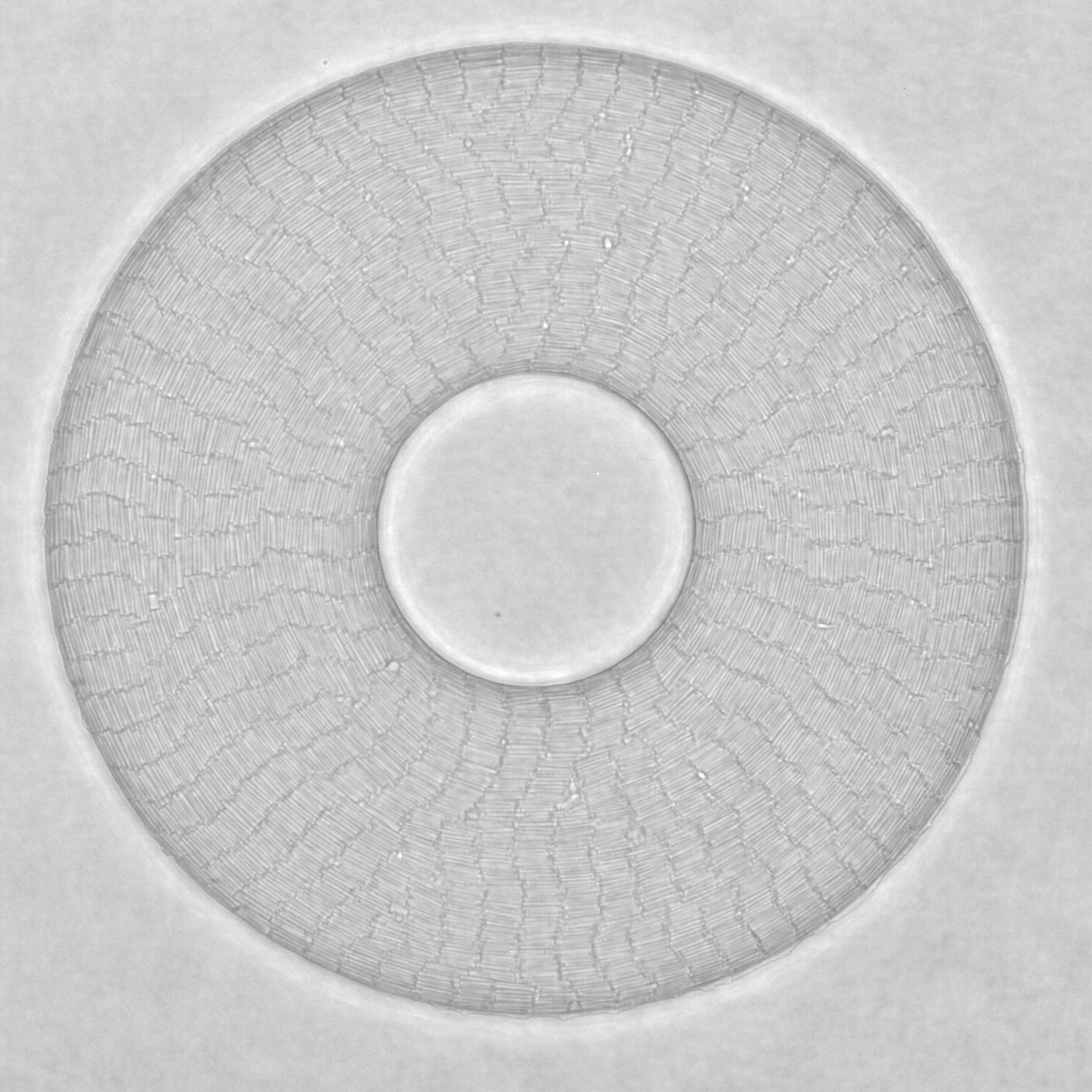

Supplement: Supplementary file 5 — Supplementary Data 2 [file 41467_2020_20842_MOESM5_ESM.zip › rawdata/size6/04_03.tif]

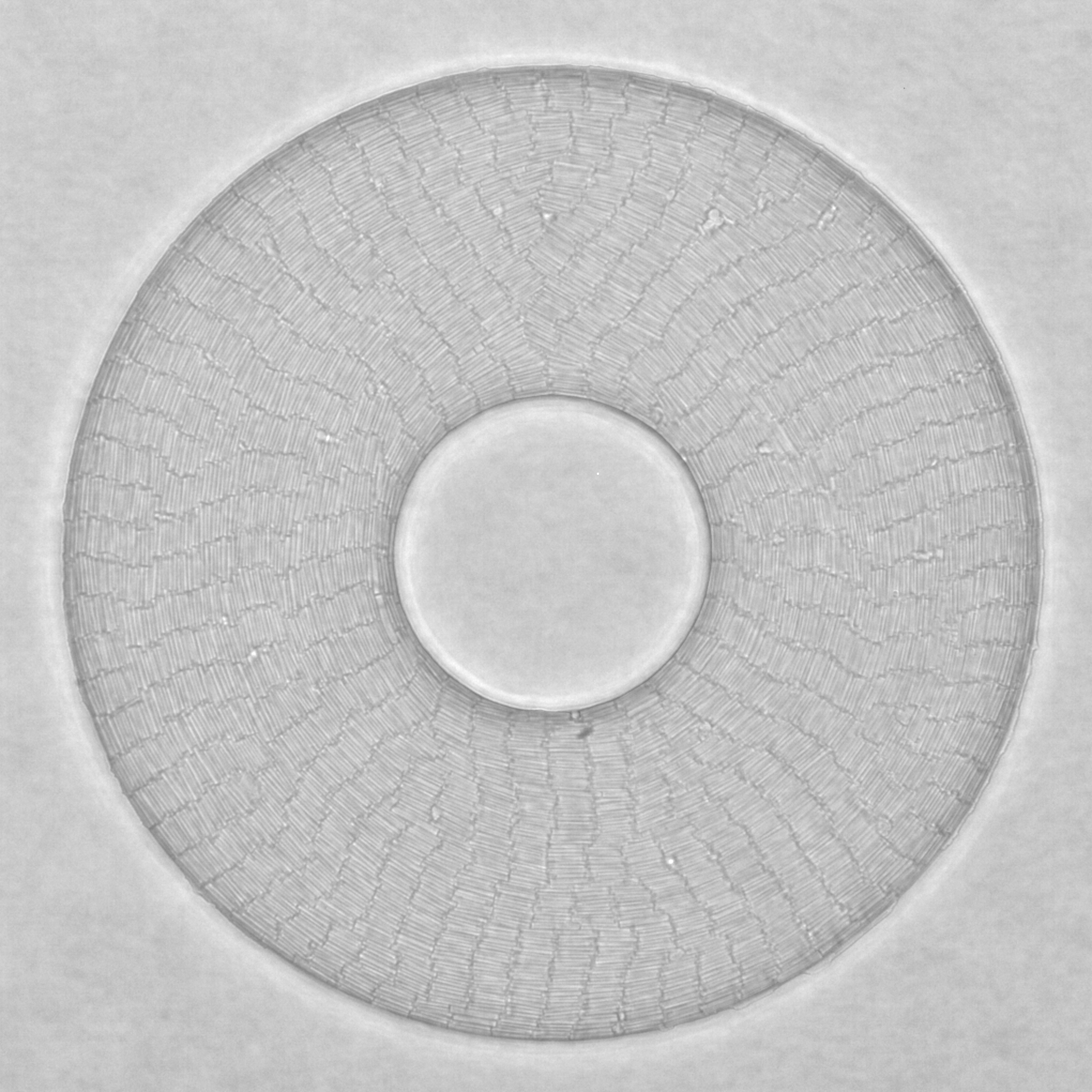

Supplement: Supplementary file 5 — Supplementary Data 2 [file 41467_2020_20842_MOESM5_ESM.zip › rawdata/size6/04_02.tif]

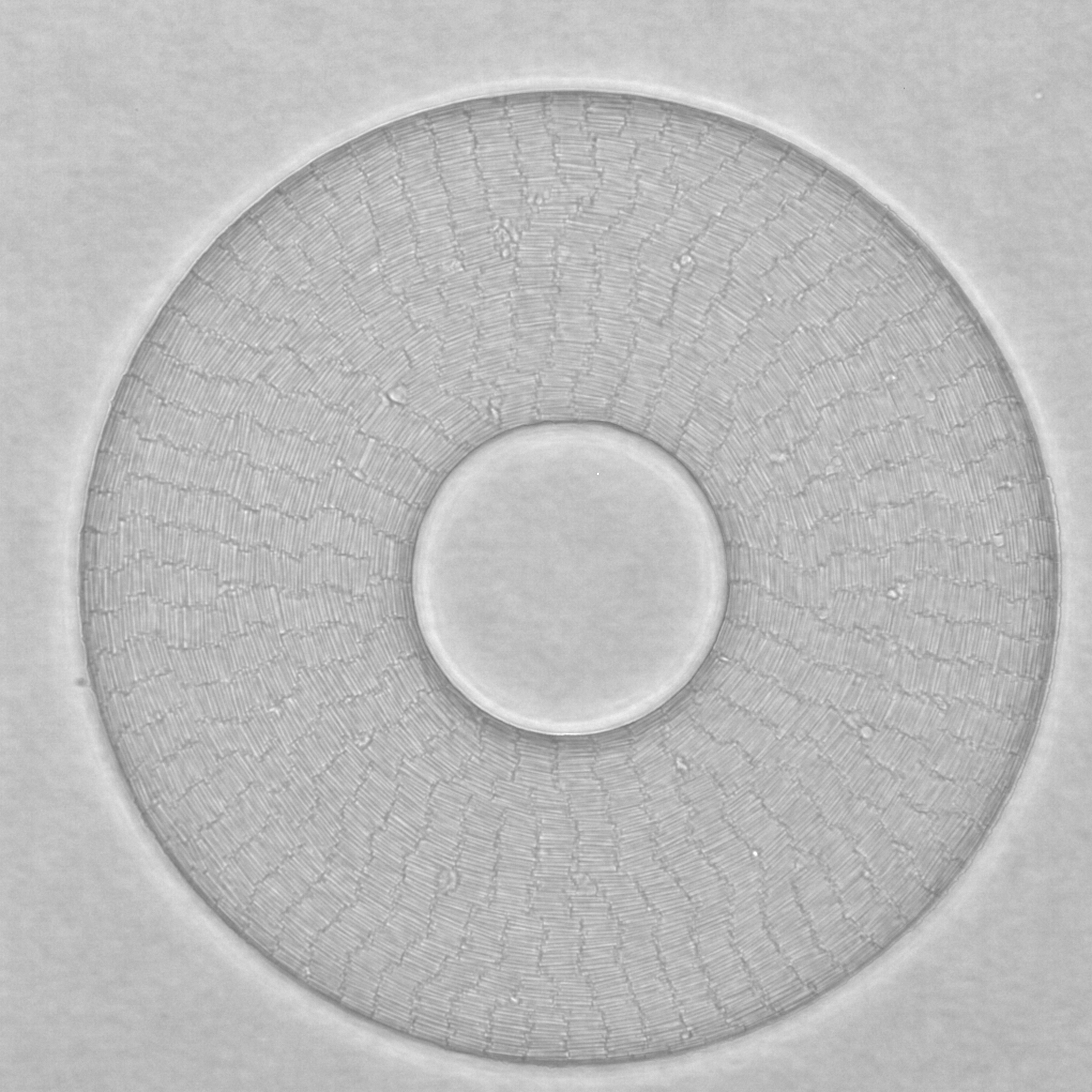

Supplement: Supplementary file 5 — Supplementary Data 2 [file 41467_2020_20842_MOESM5_ESM.zip › rawdata/size6/04_01.tif]

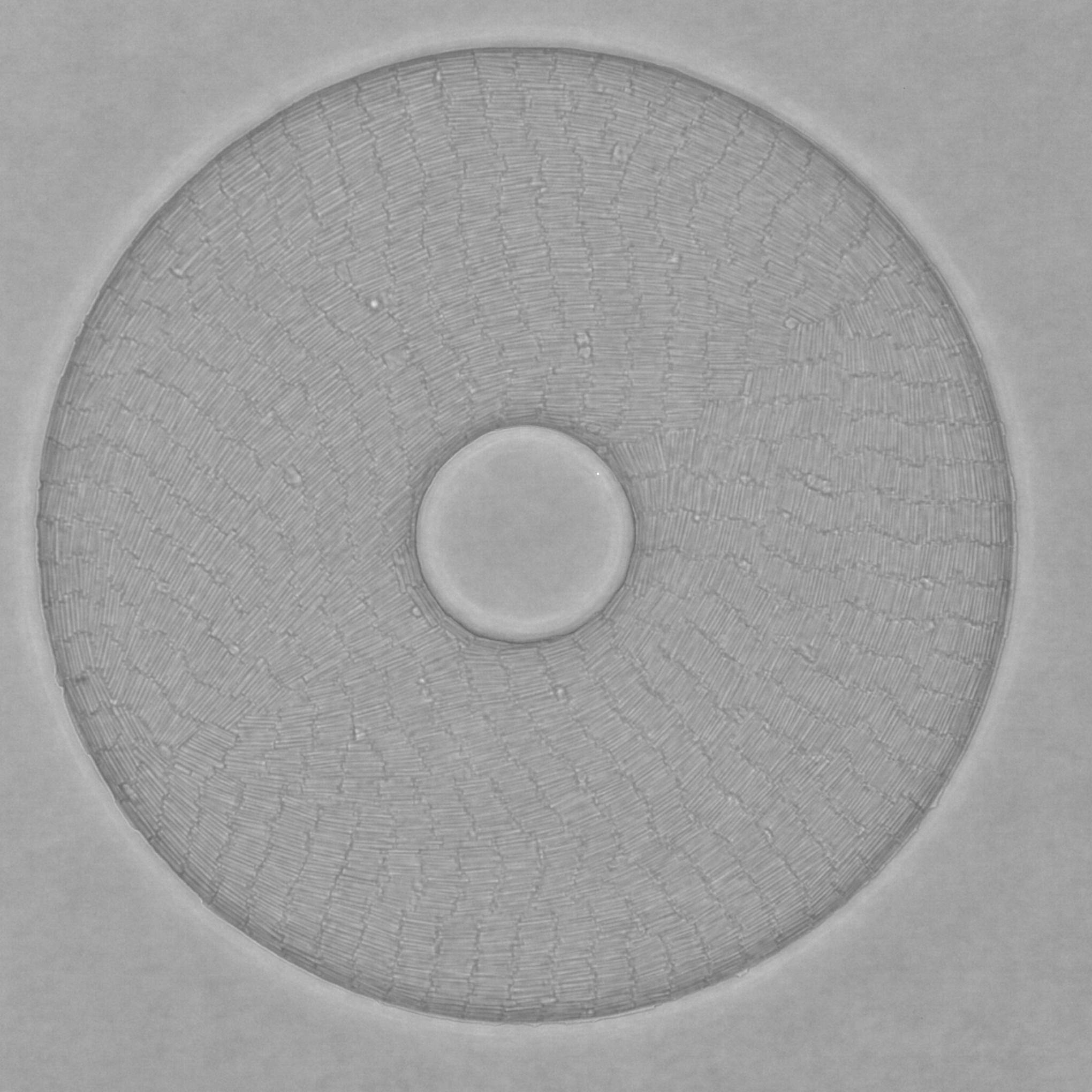

Supplement: Supplementary file 5 — Supplementary Data 2 [file 41467_2020_20842_MOESM5_ESM.zip › rawdata/size6/03_06.tif]

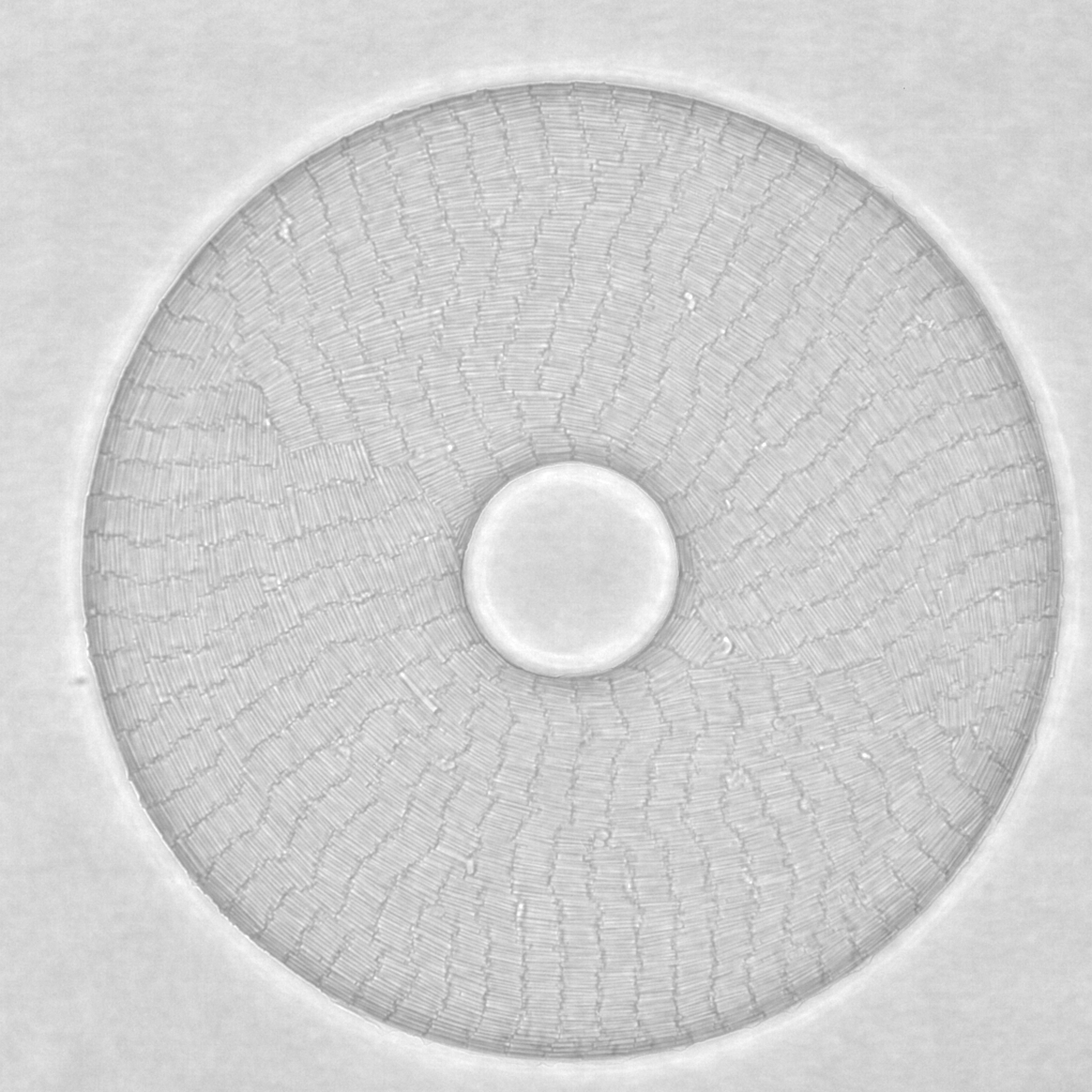

Supplement: Supplementary file 5 — Supplementary Data 2 [file 41467_2020_20842_MOESM5_ESM.zip › rawdata/size6/03_05.tif]

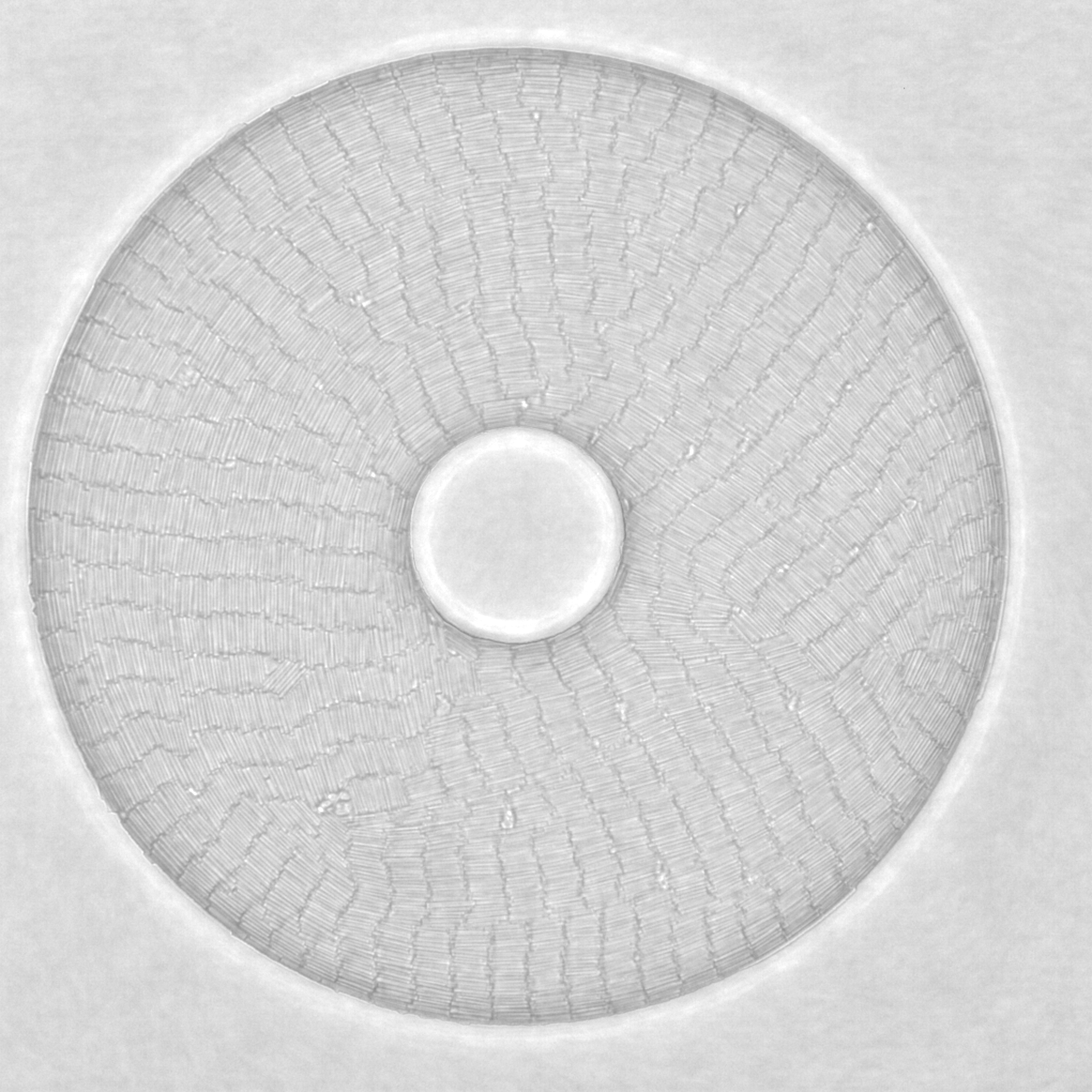

Supplement: Supplementary file 5 — Supplementary Data 2 [file 41467_2020_20842_MOESM5_ESM.zip › rawdata/size6/03_04.tif]

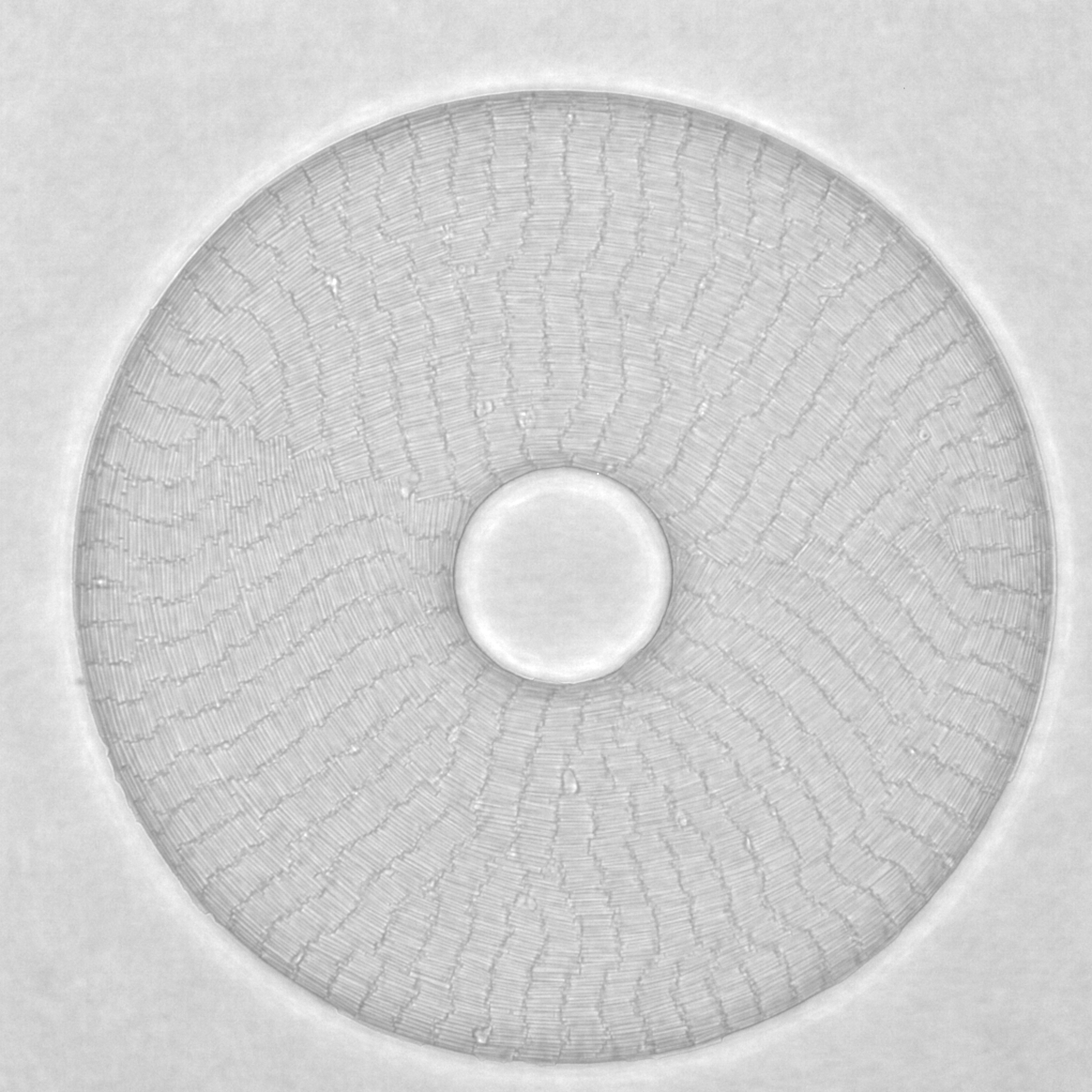

Supplement: Supplementary file 5 — Supplementary Data 2 [file 41467_2020_20842_MOESM5_ESM.zip › rawdata/size6/03_03.tif]

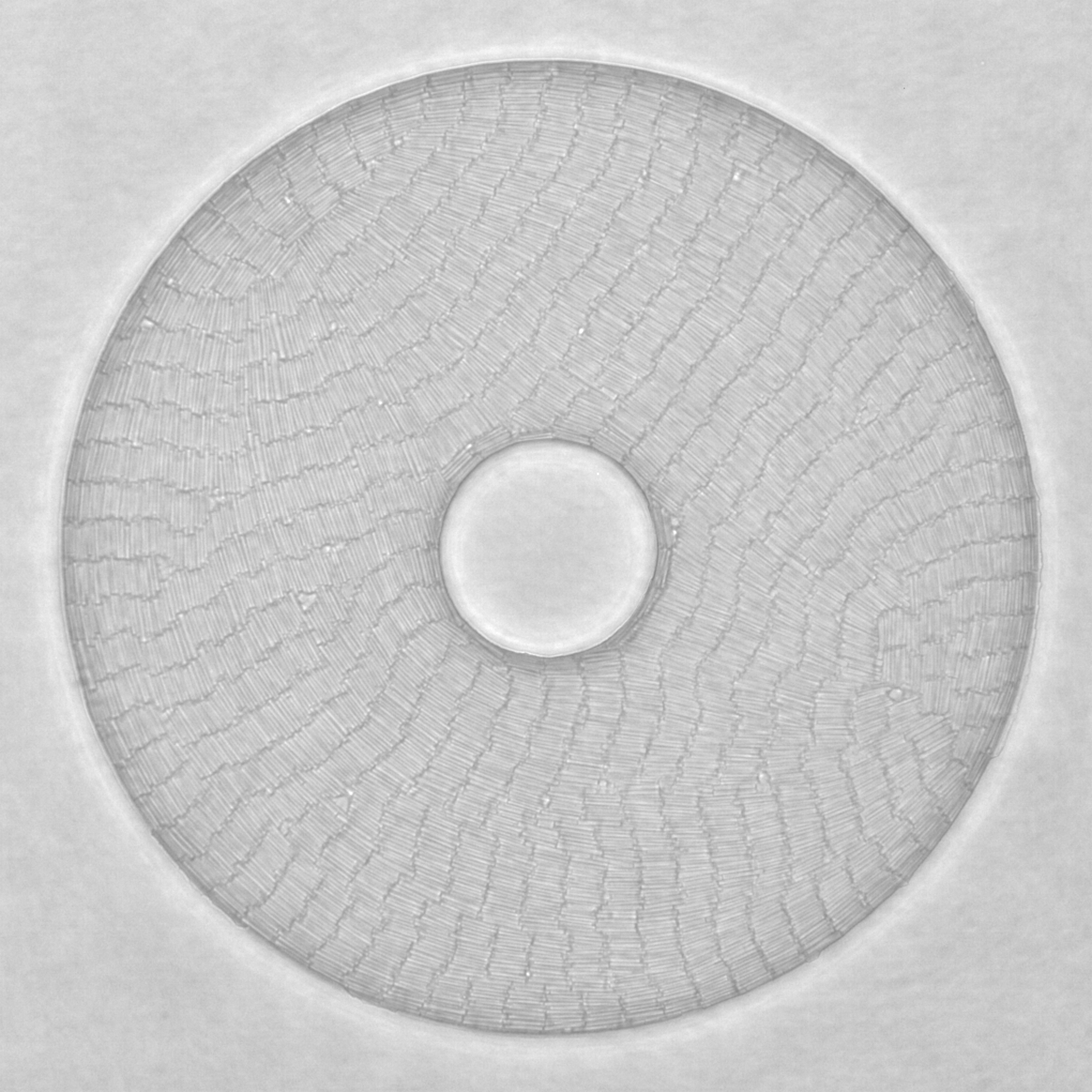

Supplement: Supplementary file 5 — Supplementary Data 2 [file 41467_2020_20842_MOESM5_ESM.zip › rawdata/size6/03_02.tif]

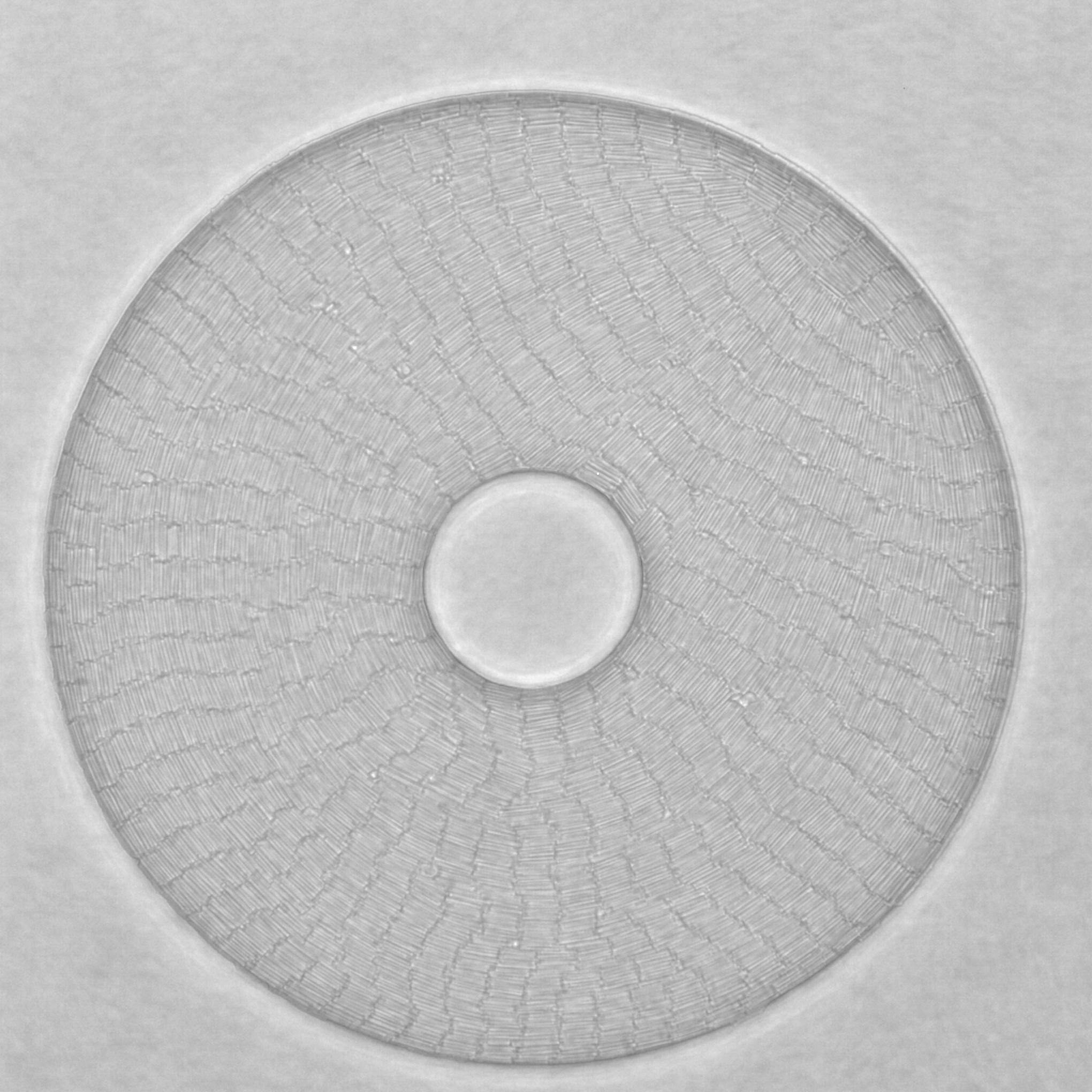

Supplement: Supplementary file 5 — Supplementary Data 2 [file 41467_2020_20842_MOESM5_ESM.zip › rawdata/size6/03_01.tif]

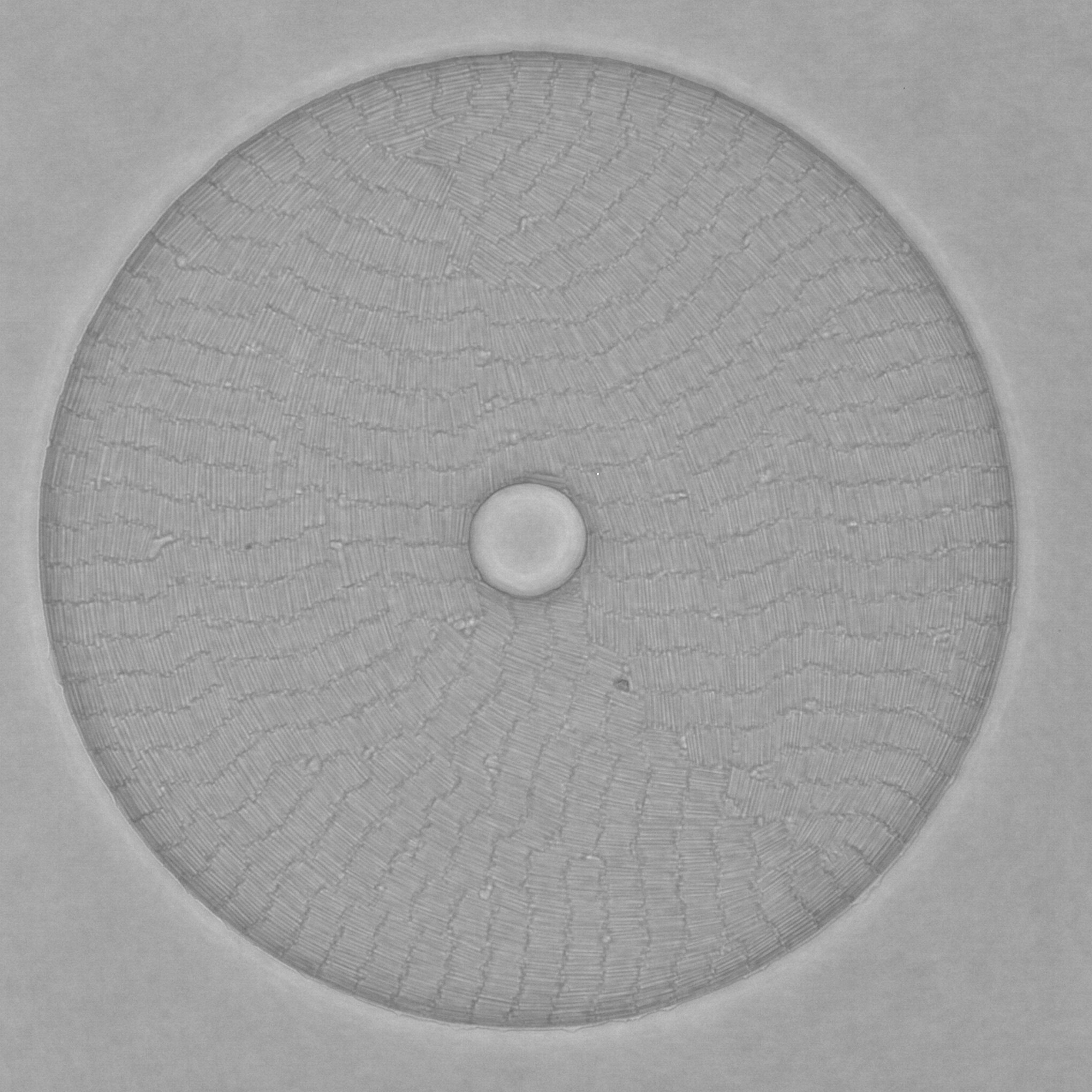

Supplement: Supplementary file 5 — Supplementary Data 2 [file 41467_2020_20842_MOESM5_ESM.zip › rawdata/size6/02_06.tif]

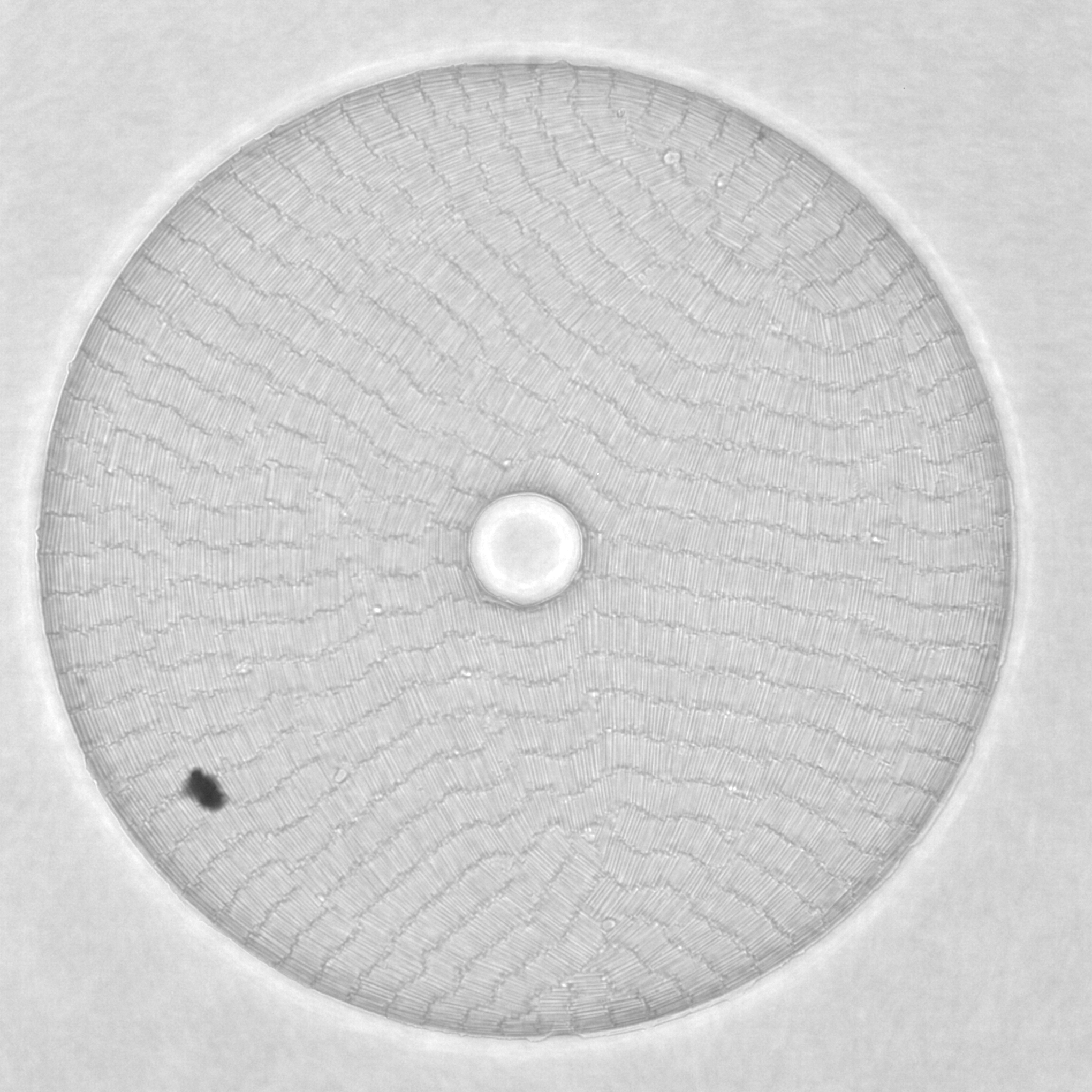

Supplement: Supplementary file 5 — Supplementary Data 2 [file 41467_2020_20842_MOESM5_ESM.zip › rawdata/size6/02_05.tif]

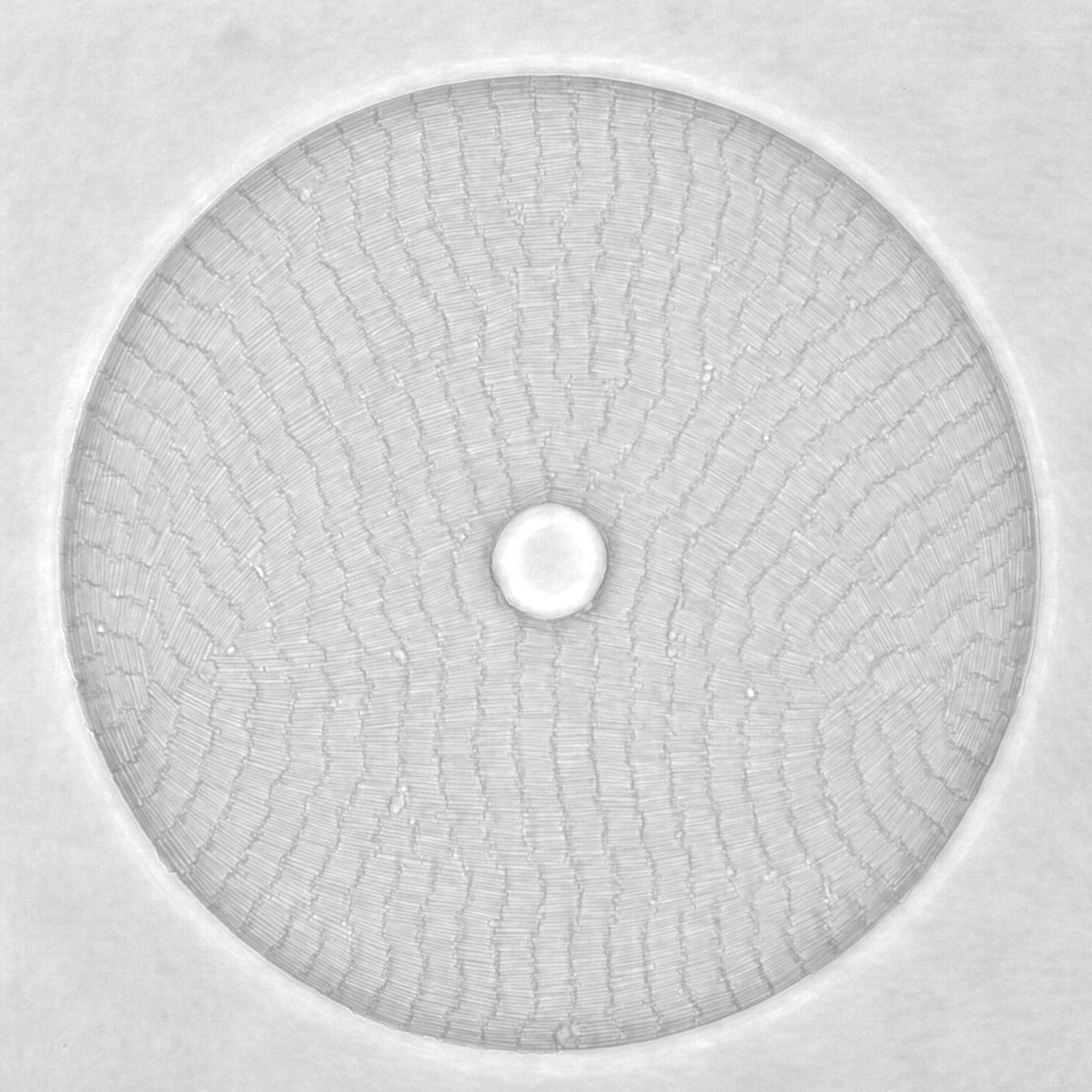

Supplement: Supplementary file 5 — Supplementary Data 2 [file 41467_2020_20842_MOESM5_ESM.zip › rawdata/size6/02_04.tif]

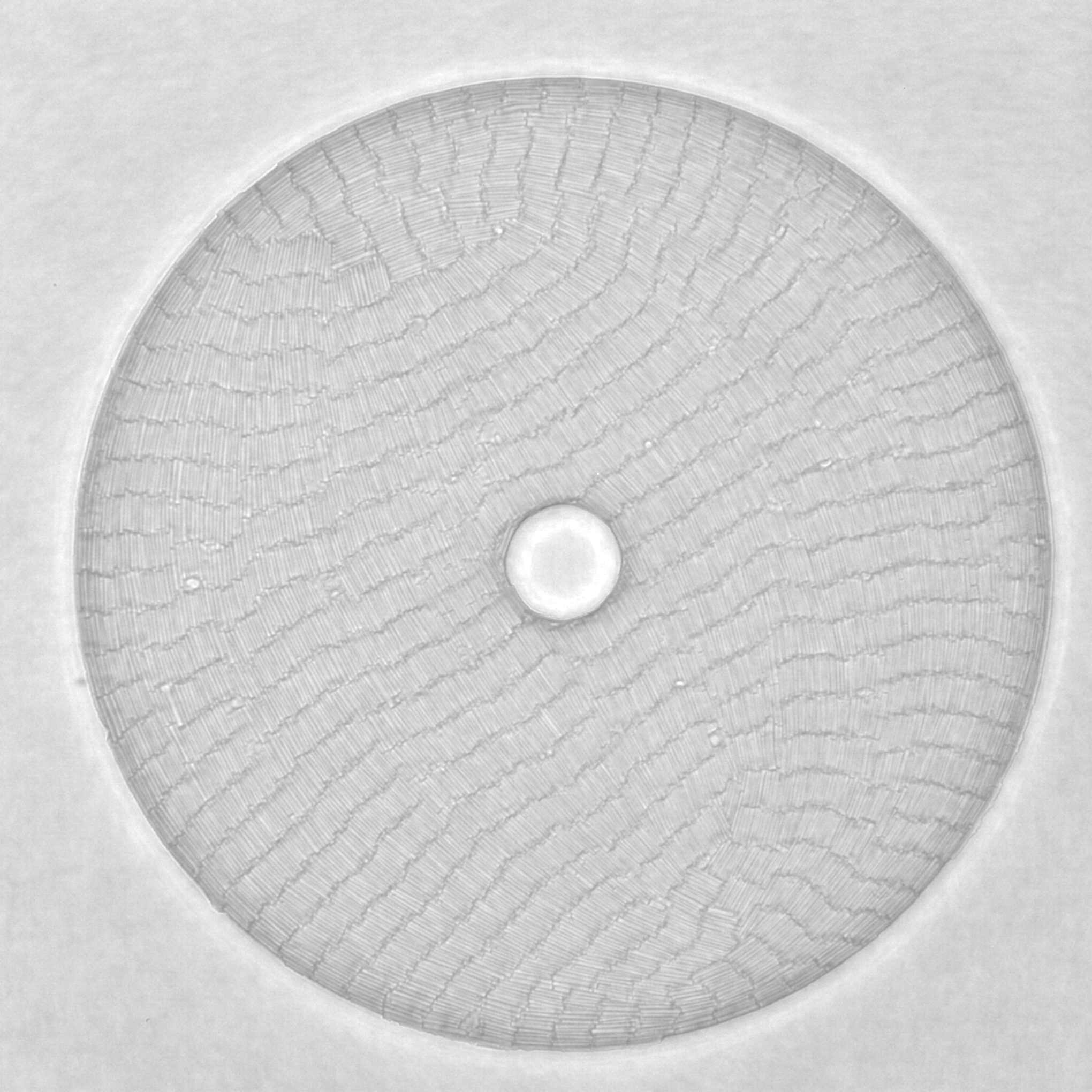

Supplement: Supplementary file 5 — Supplementary Data 2 [file 41467_2020_20842_MOESM5_ESM.zip › rawdata/size6/02_03.tif]

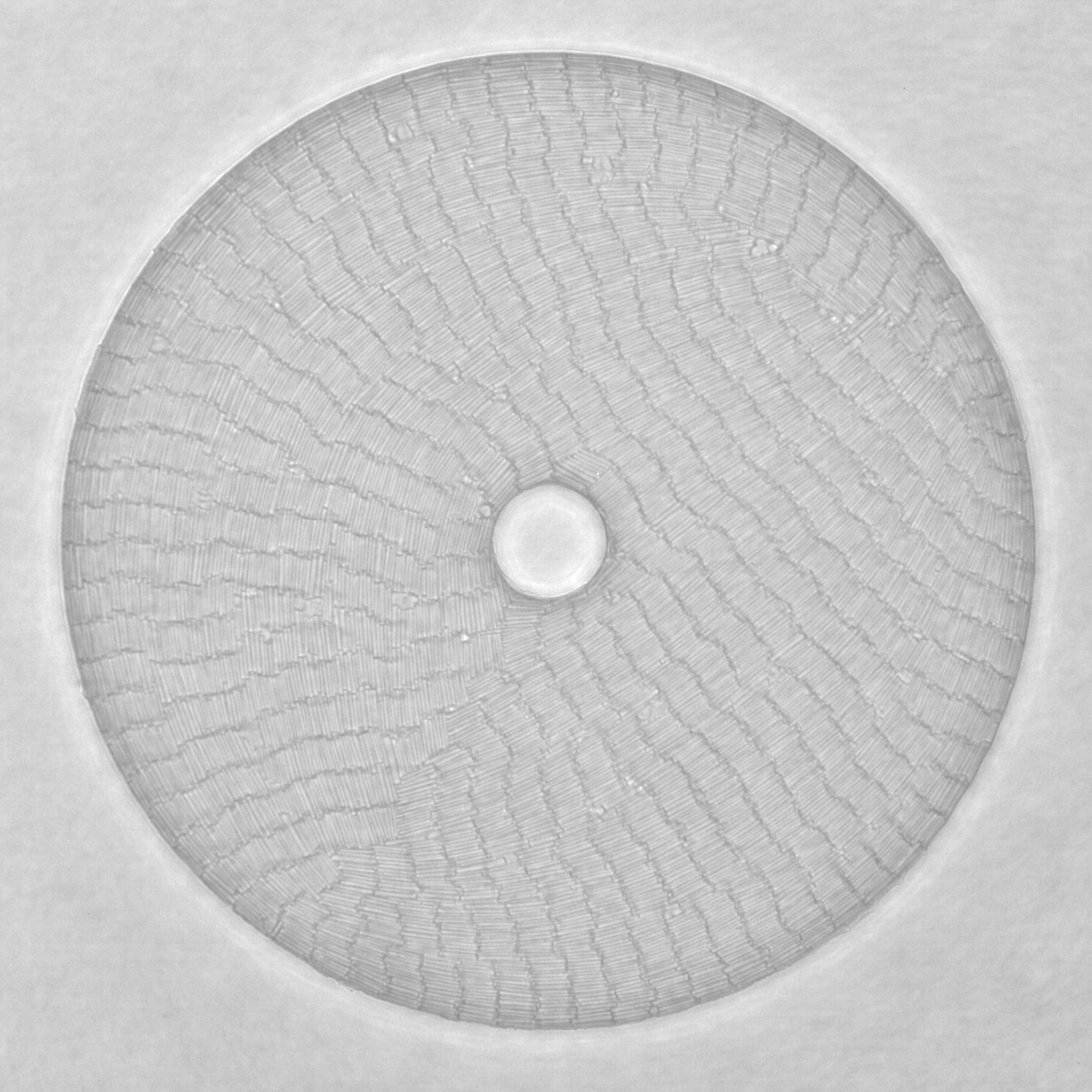

Supplement: Supplementary file 5 — Supplementary Data 2 [file 41467_2020_20842_MOESM5_ESM.zip › rawdata/size6/02_02.tif]

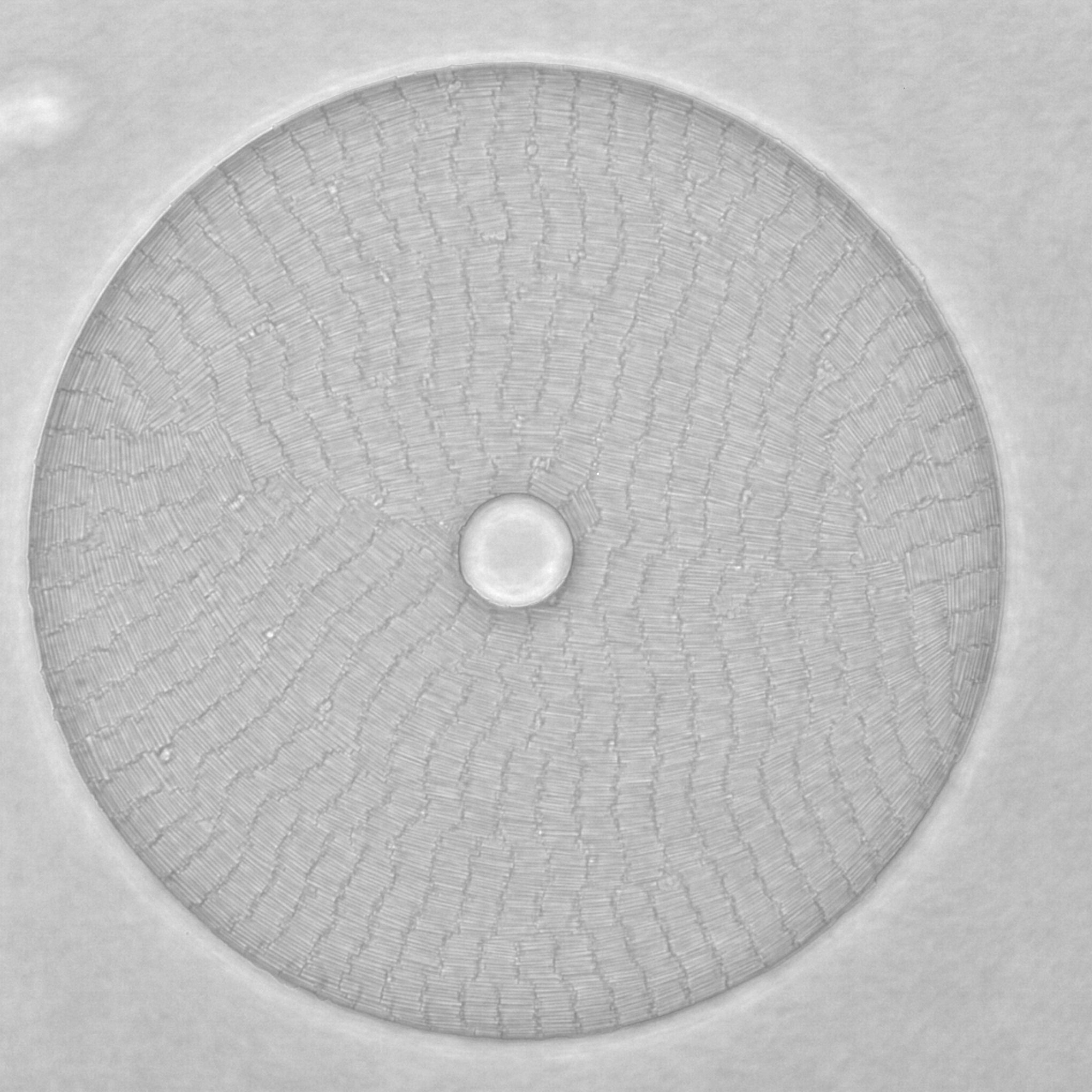

Supplement: Supplementary file 5 — Supplementary Data 2 [file 41467_2020_20842_MOESM5_ESM.zip › rawdata/size6/02_01.tif]

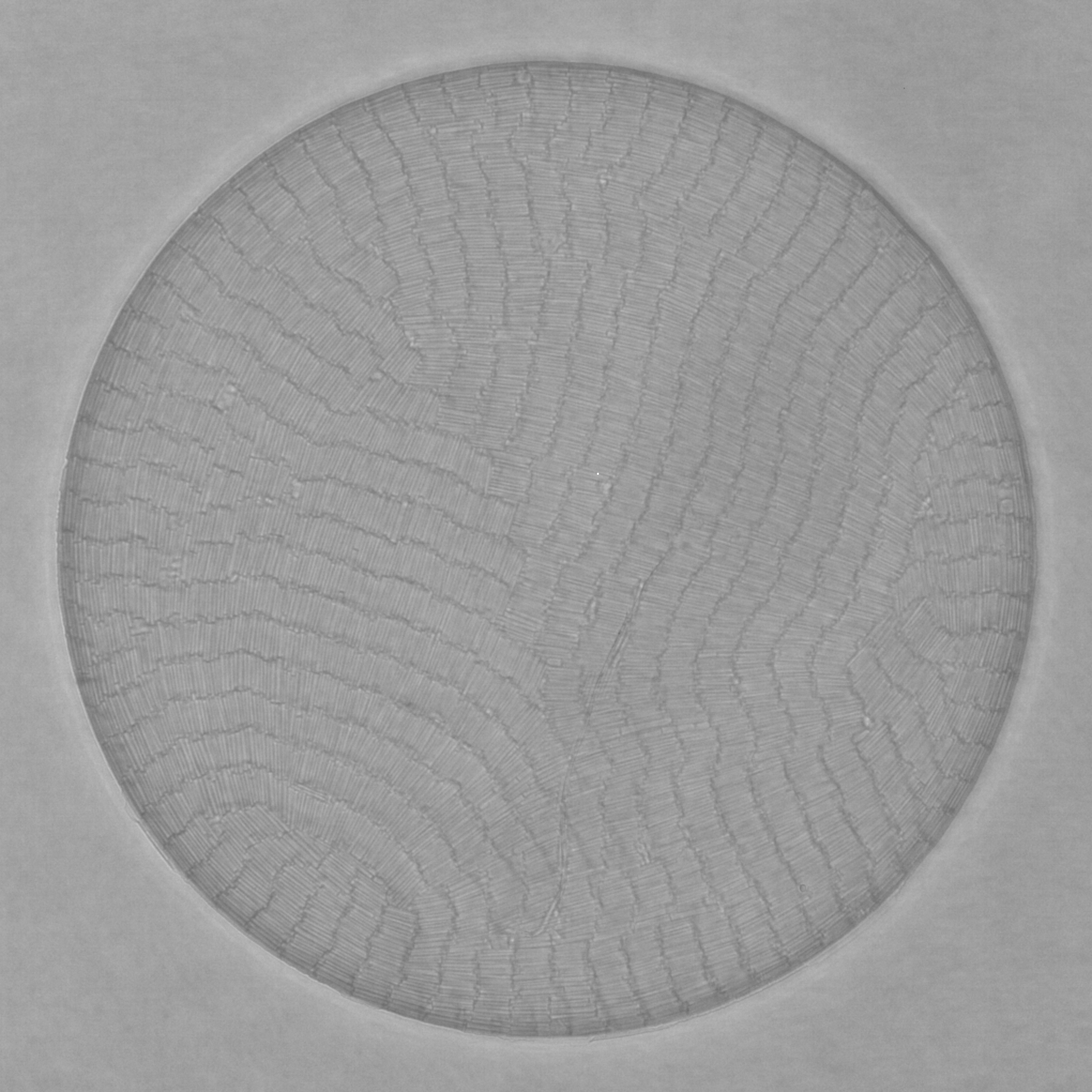

Supplement: Supplementary file 5 — Supplementary Data 2 [file 41467_2020_20842_MOESM5_ESM.zip › rawdata/size6/01_06.tif]

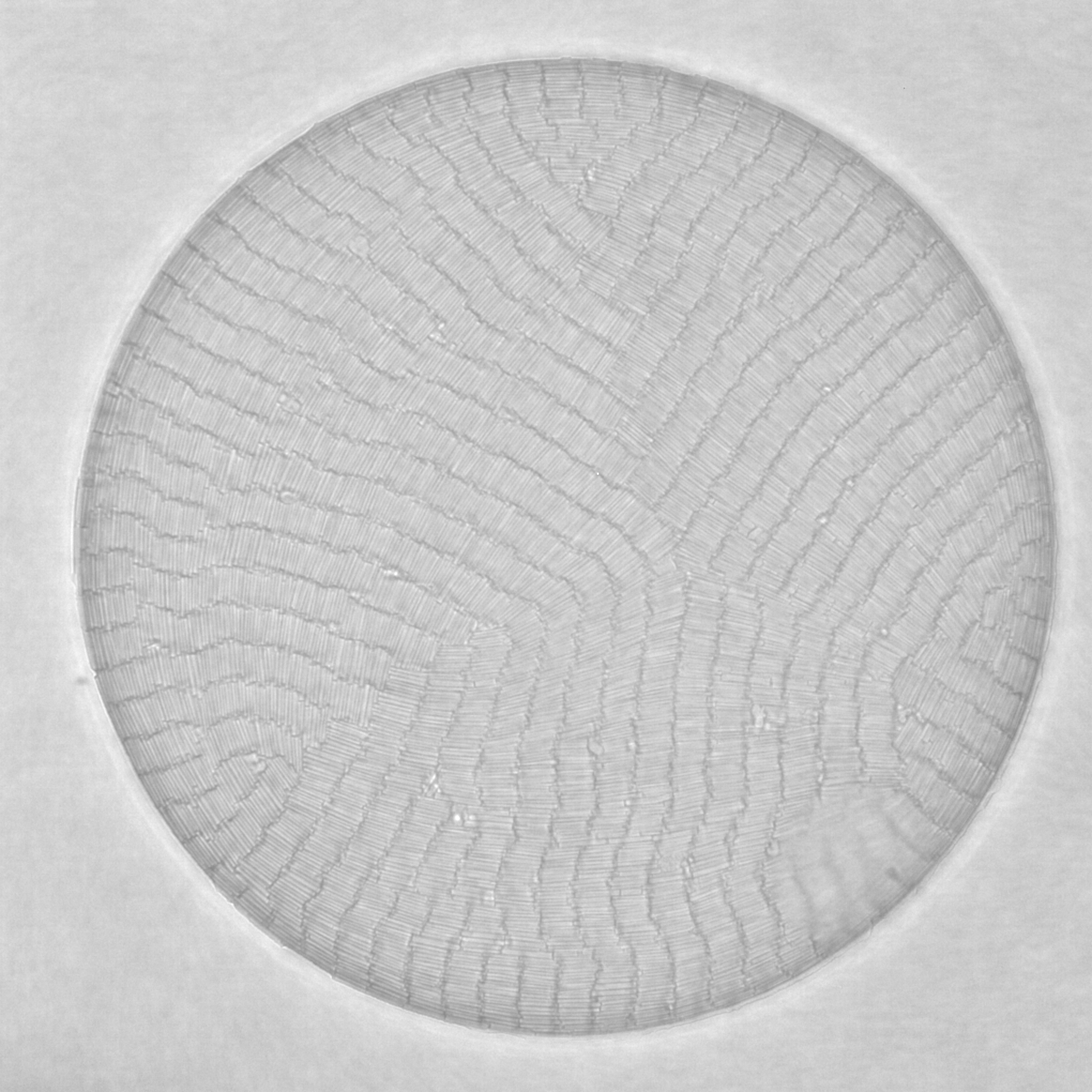

Supplement: Supplementary file 5 — Supplementary Data 2 [file 41467_2020_20842_MOESM5_ESM.zip › rawdata/size6/01_05.tif]

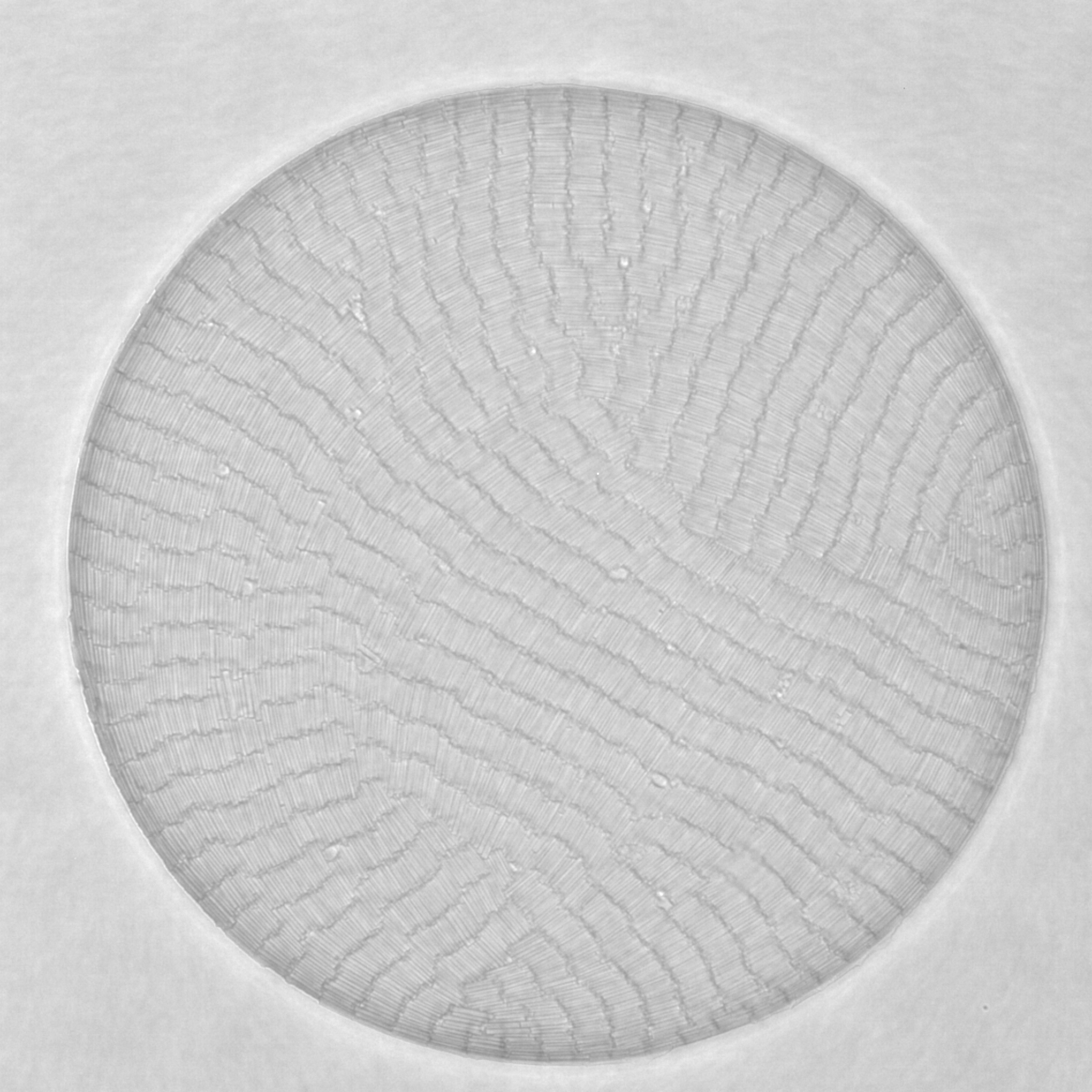

Supplement: Supplementary file 5 — Supplementary Data 2 [file 41467_2020_20842_MOESM5_ESM.zip › rawdata/size6/01_04.tif]

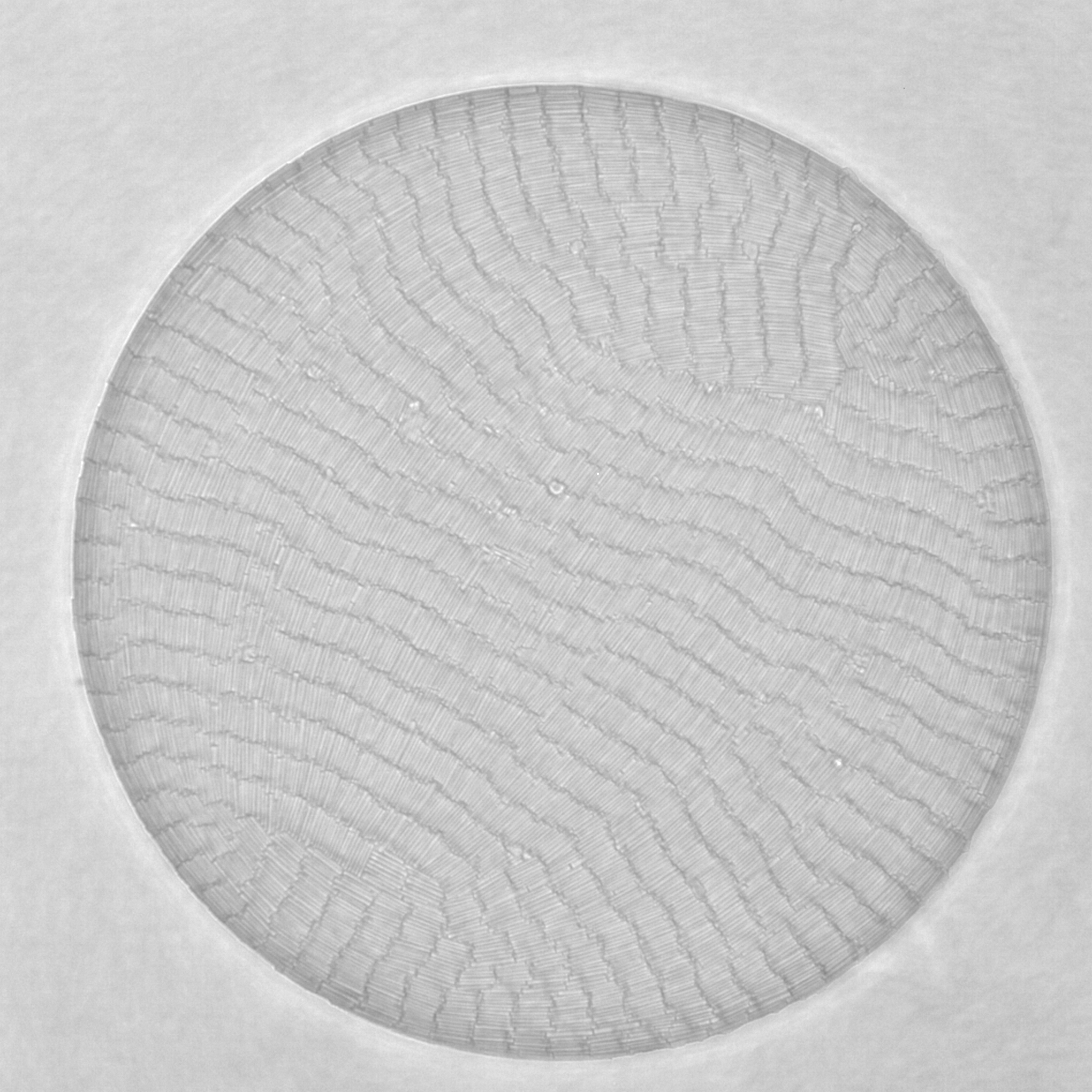

Supplement: Supplementary file 5 — Supplementary Data 2 [file 41467_2020_20842_MOESM5_ESM.zip › rawdata/size6/01_03.tif]

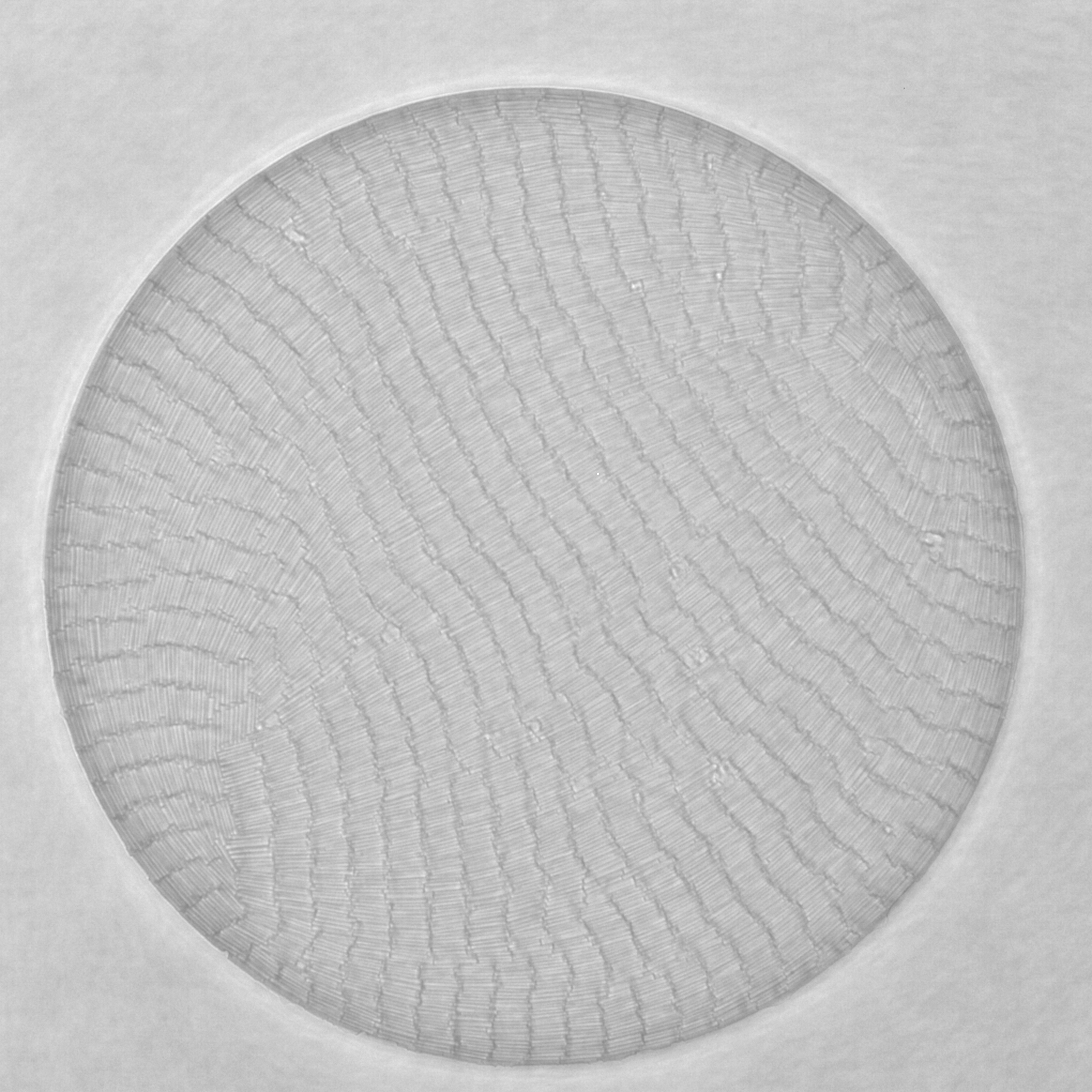

Supplement: Supplementary file 5 — Supplementary Data 2 [file 41467_2020_20842_MOESM5_ESM.zip › rawdata/size6/01_02.tif]

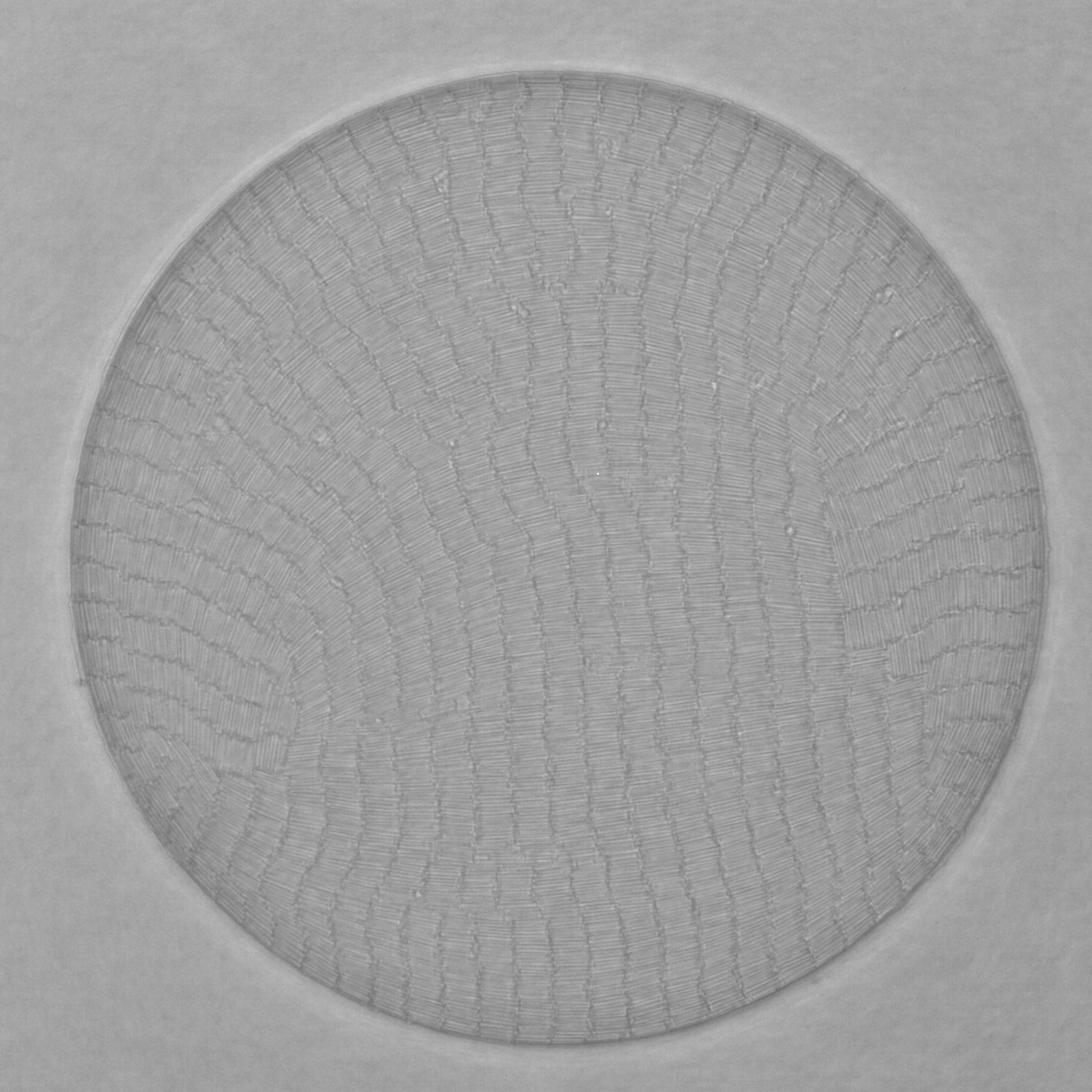

Supplement: Supplementary file 5 — Supplementary Data 2 [file 41467_2020_20842_MOESM5_ESM.zip › rawdata/size6/01_01.tif]

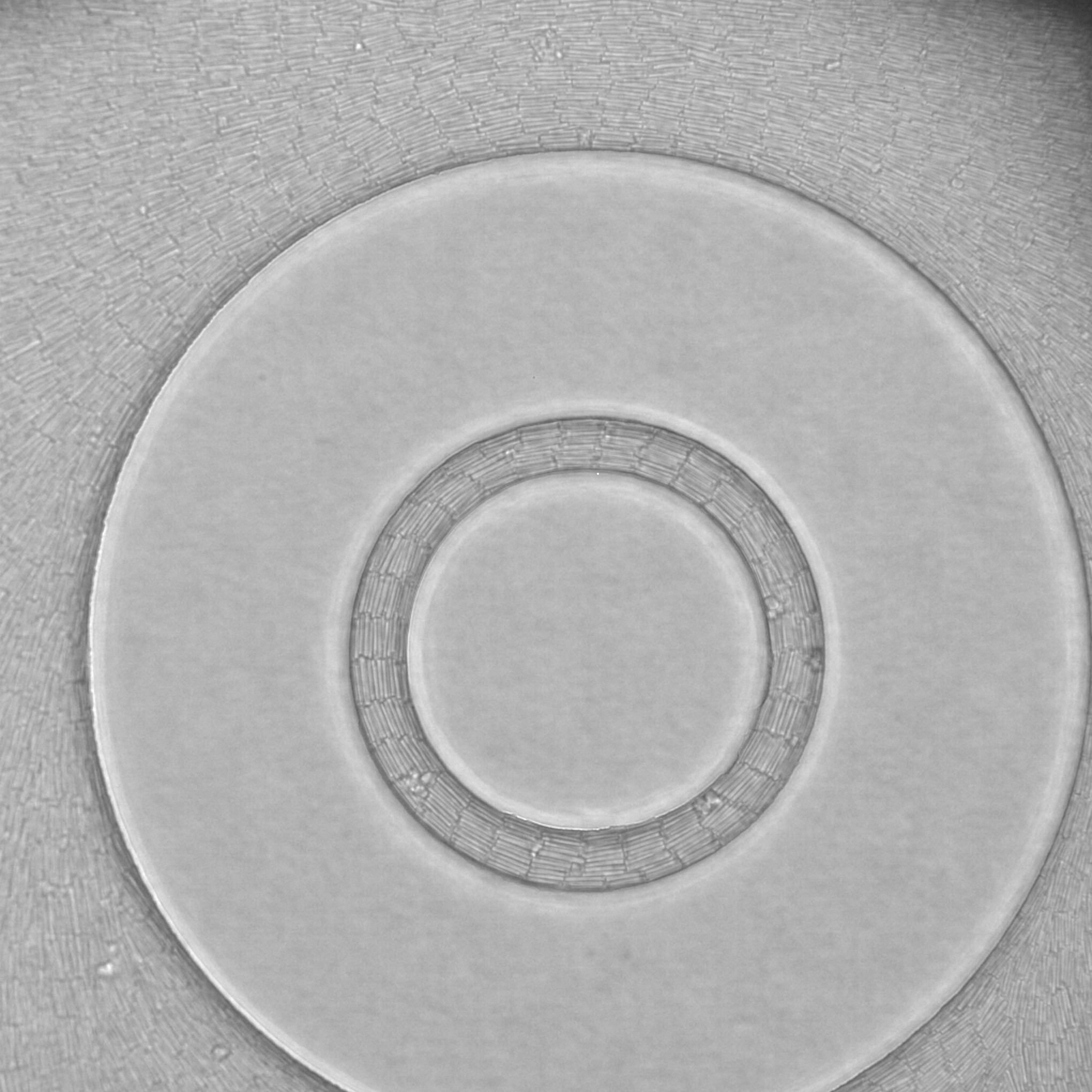

Supplement: Supplementary file 5 — Supplementary Data 2 [file 41467_2020_20842_MOESM5_ESM.zip › rawdata/size5/06_06.tif]

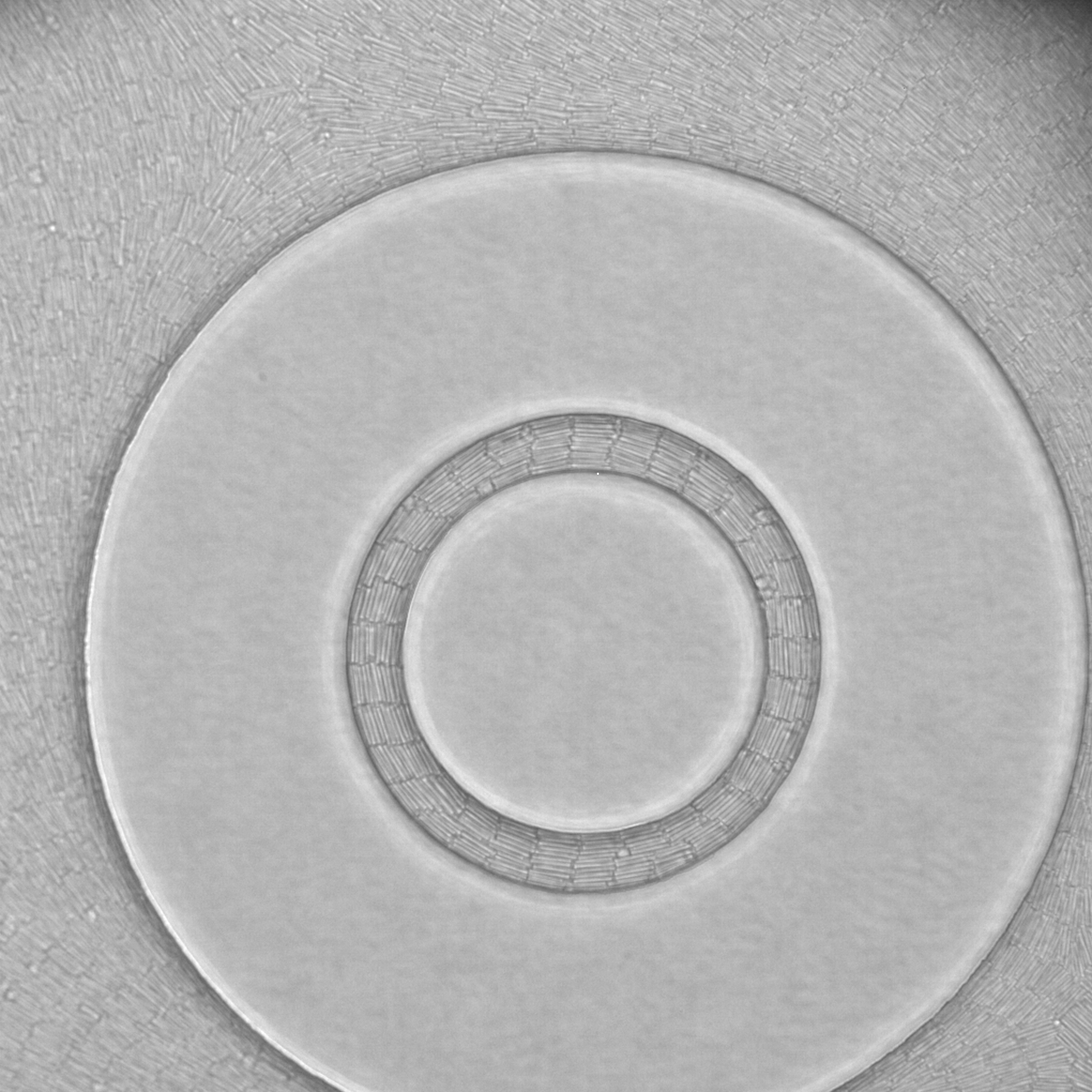

Supplement: Supplementary file 5 — Supplementary Data 2 [file 41467_2020_20842_MOESM5_ESM.zip › rawdata/size5/06_05.tif]

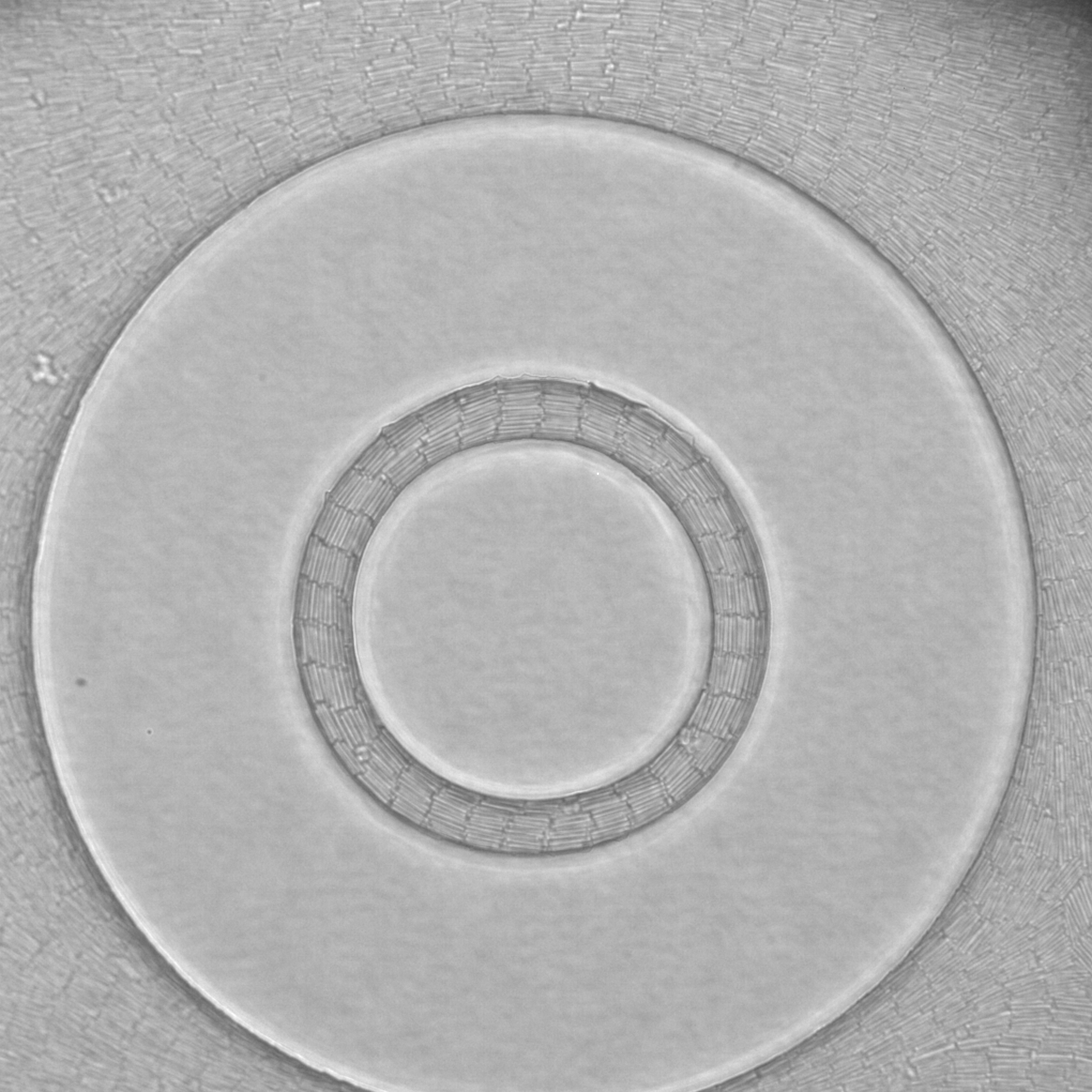

Supplement: Supplementary file 5 — Supplementary Data 2 [file 41467_2020_20842_MOESM5_ESM.zip › rawdata/size5/06_04.tif]

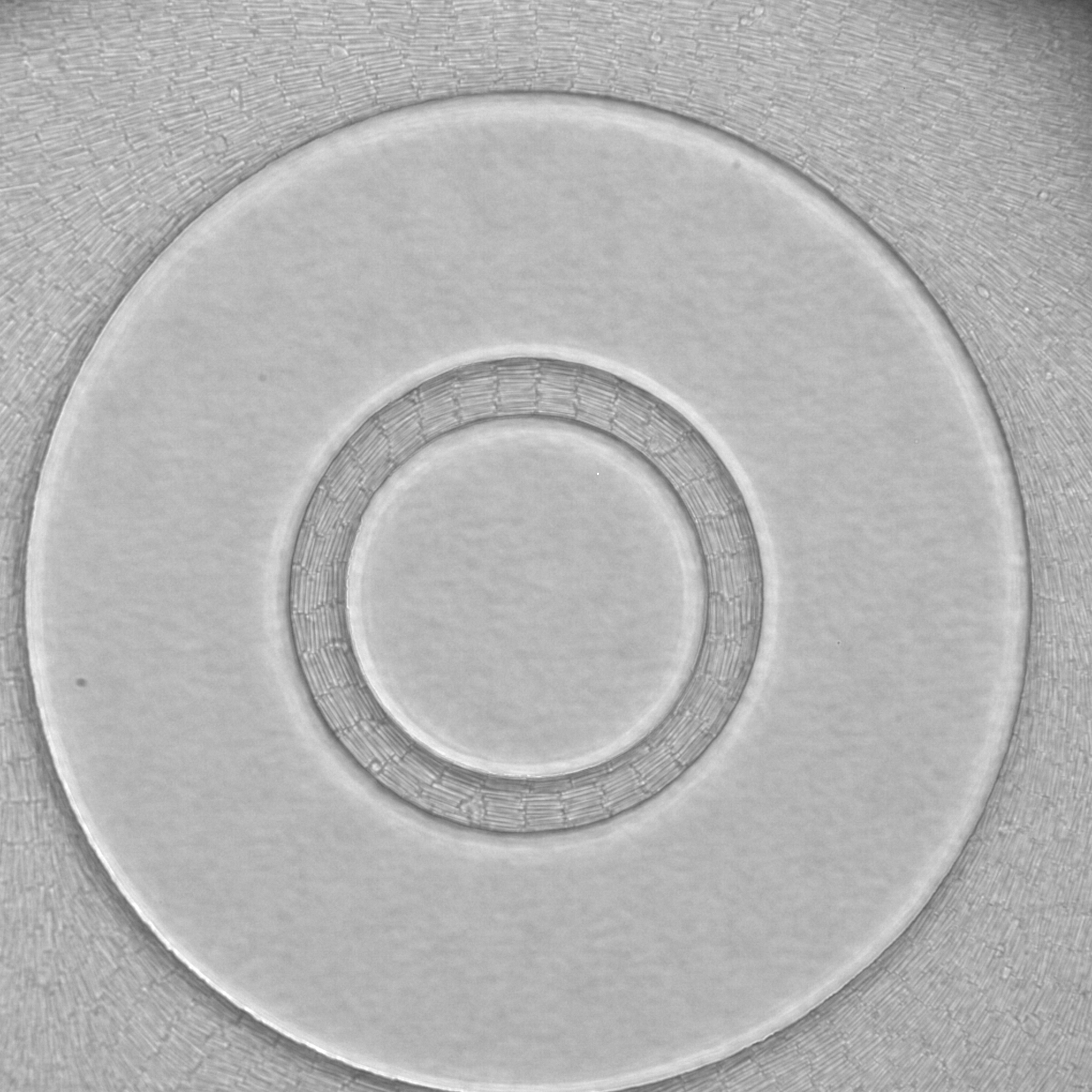

Supplement: Supplementary file 5 — Supplementary Data 2 [file 41467_2020_20842_MOESM5_ESM.zip › rawdata/size5/06_03.tif]

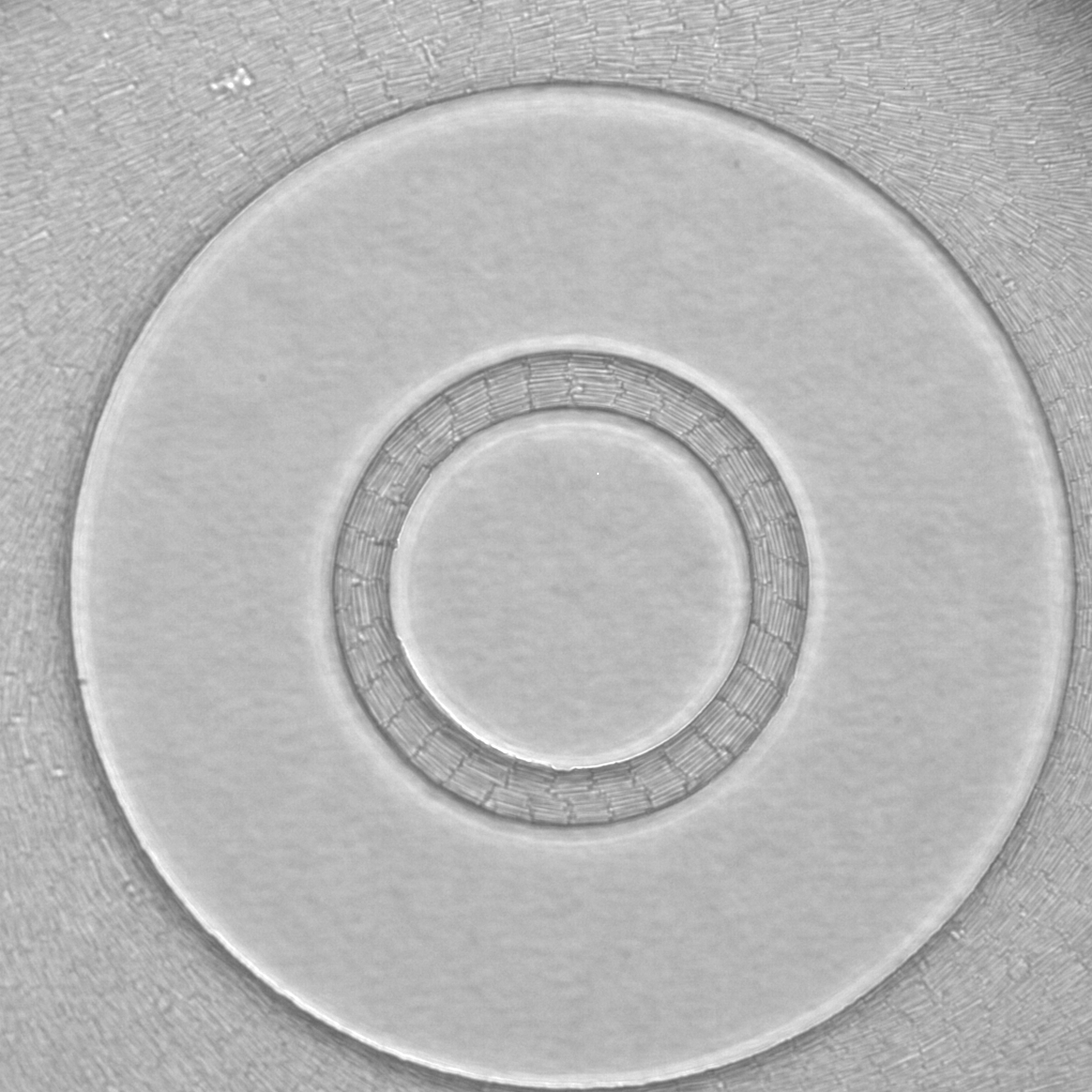

Supplement: Supplementary file 5 — Supplementary Data 2 [file 41467_2020_20842_MOESM5_ESM.zip › rawdata/size5/06_02.tif]

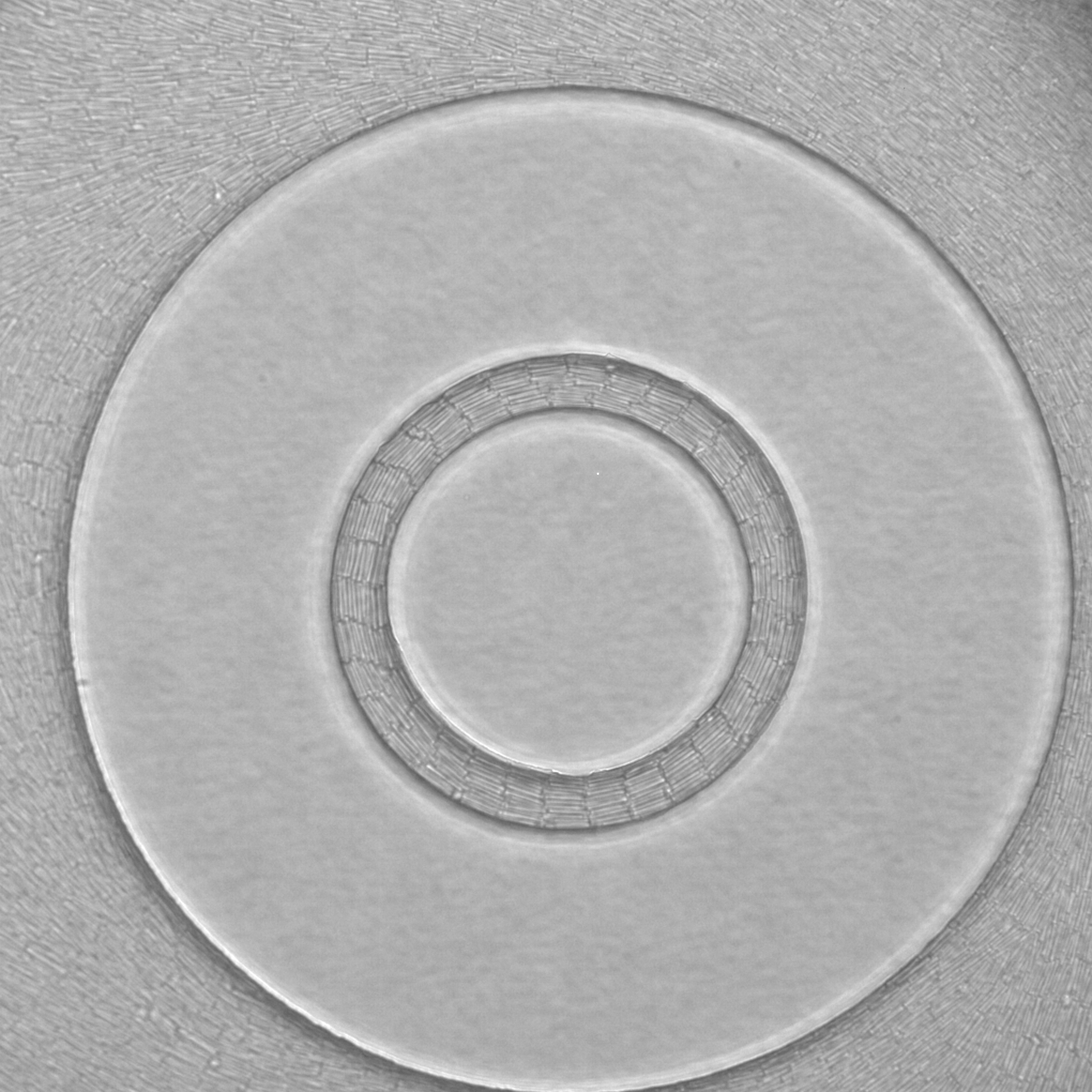

Supplement: Supplementary file 5 — Supplementary Data 2 [file 41467_2020_20842_MOESM5_ESM.zip › rawdata/size5/06_01.tif]

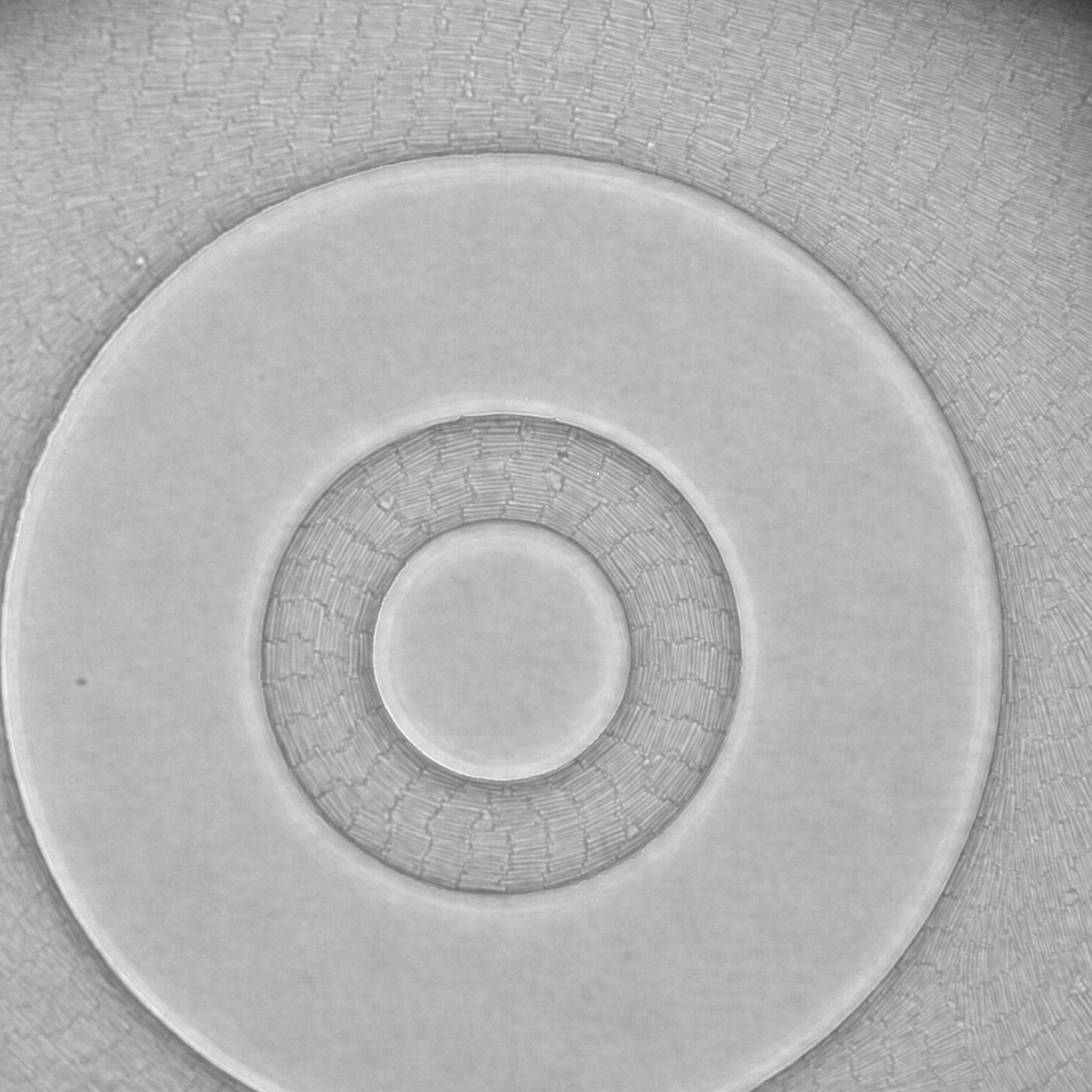

Supplement: Supplementary file 5 — Supplementary Data 2 [file 41467_2020_20842_MOESM5_ESM.zip › rawdata/size5/05_06.tif]

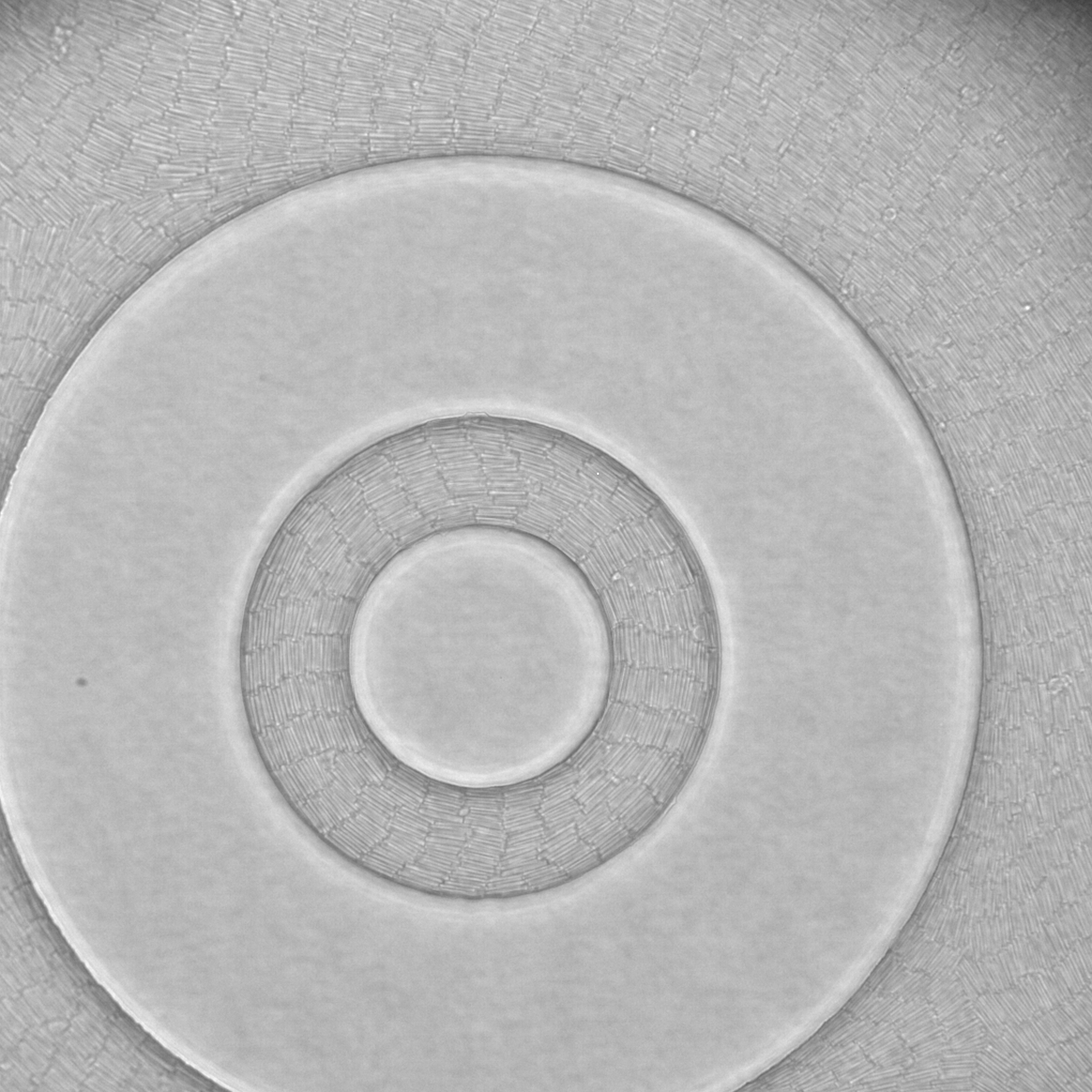

Supplement: Supplementary file 5 — Supplementary Data 2 [file 41467_2020_20842_MOESM5_ESM.zip › rawdata/size5/05_05.tif]

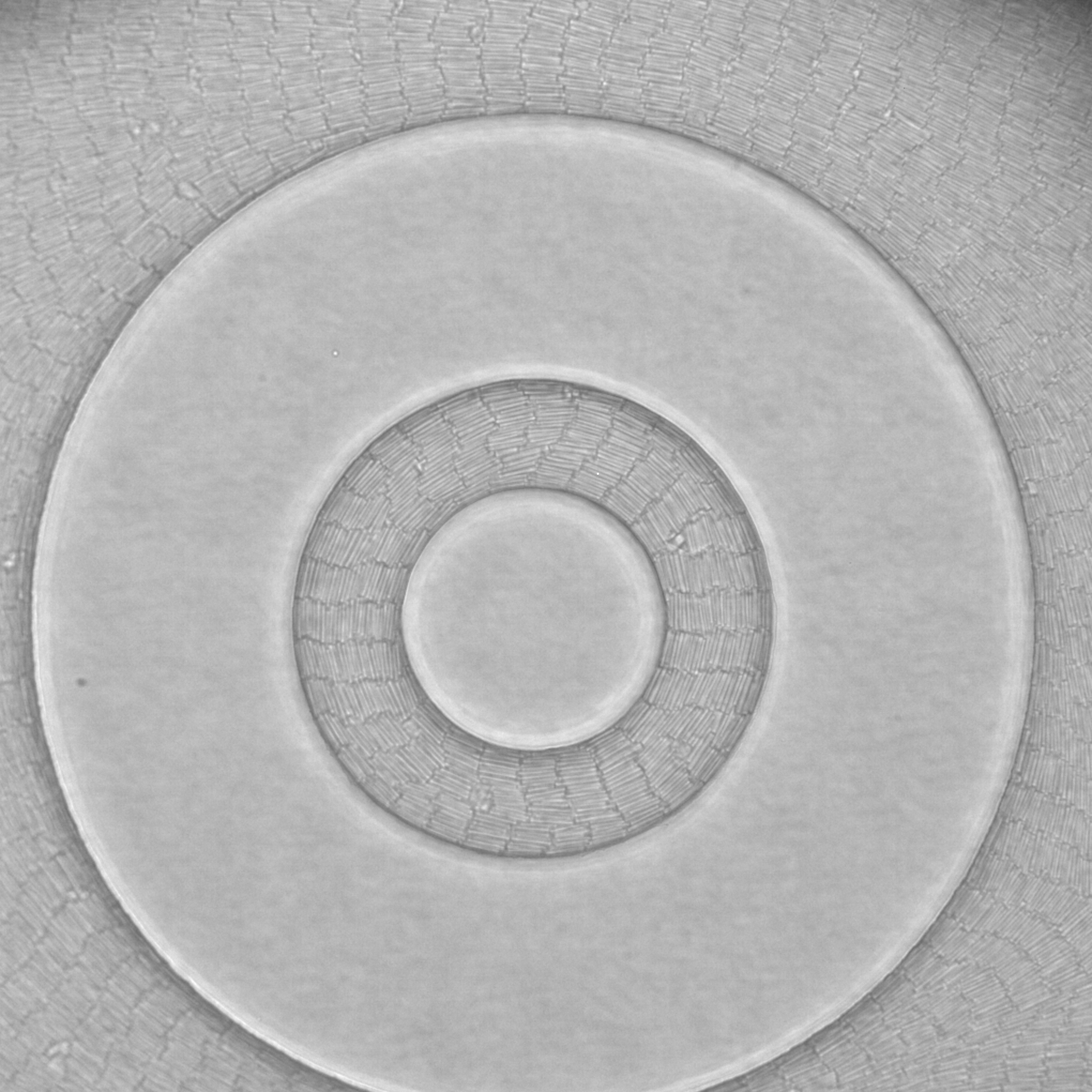

Supplement: Supplementary file 5 — Supplementary Data 2 [file 41467_2020_20842_MOESM5_ESM.zip › rawdata/size5/05_04.tif]

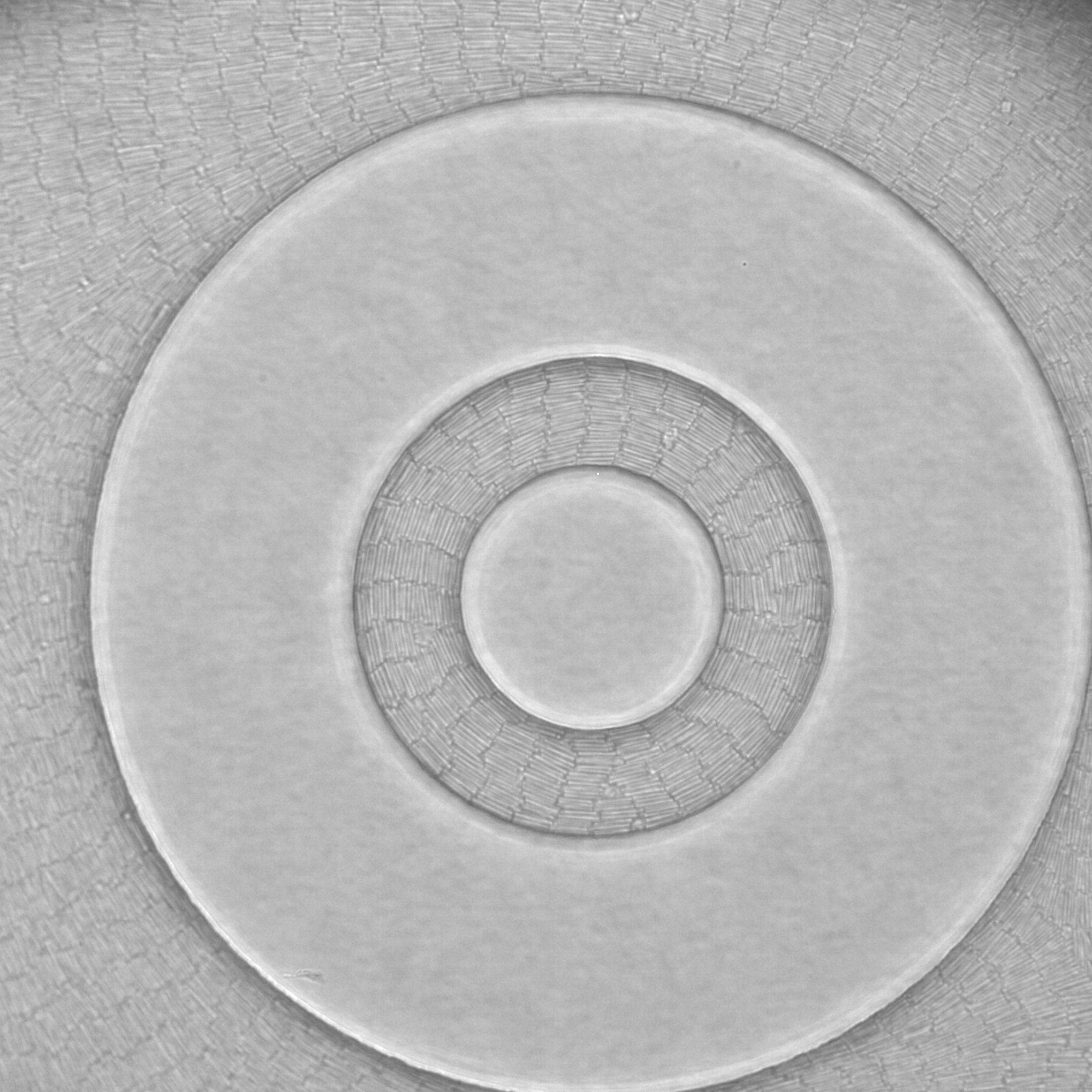

Supplement: Supplementary file 5 — Supplementary Data 2 [file 41467_2020_20842_MOESM5_ESM.zip › rawdata/size5/05_03.tif]

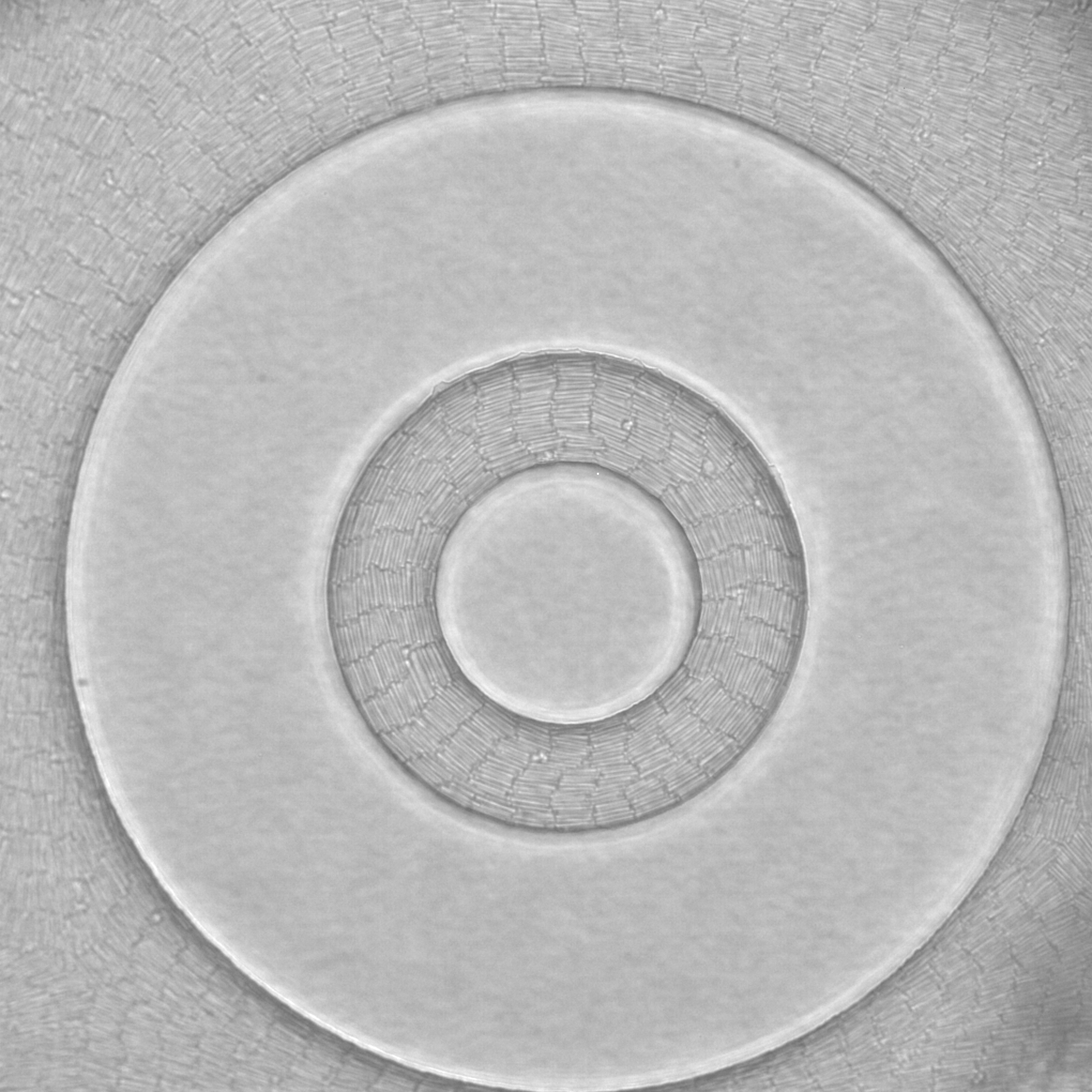

Supplement: Supplementary file 5 — Supplementary Data 2 [file 41467_2020_20842_MOESM5_ESM.zip › rawdata/size5/05_02.tif]

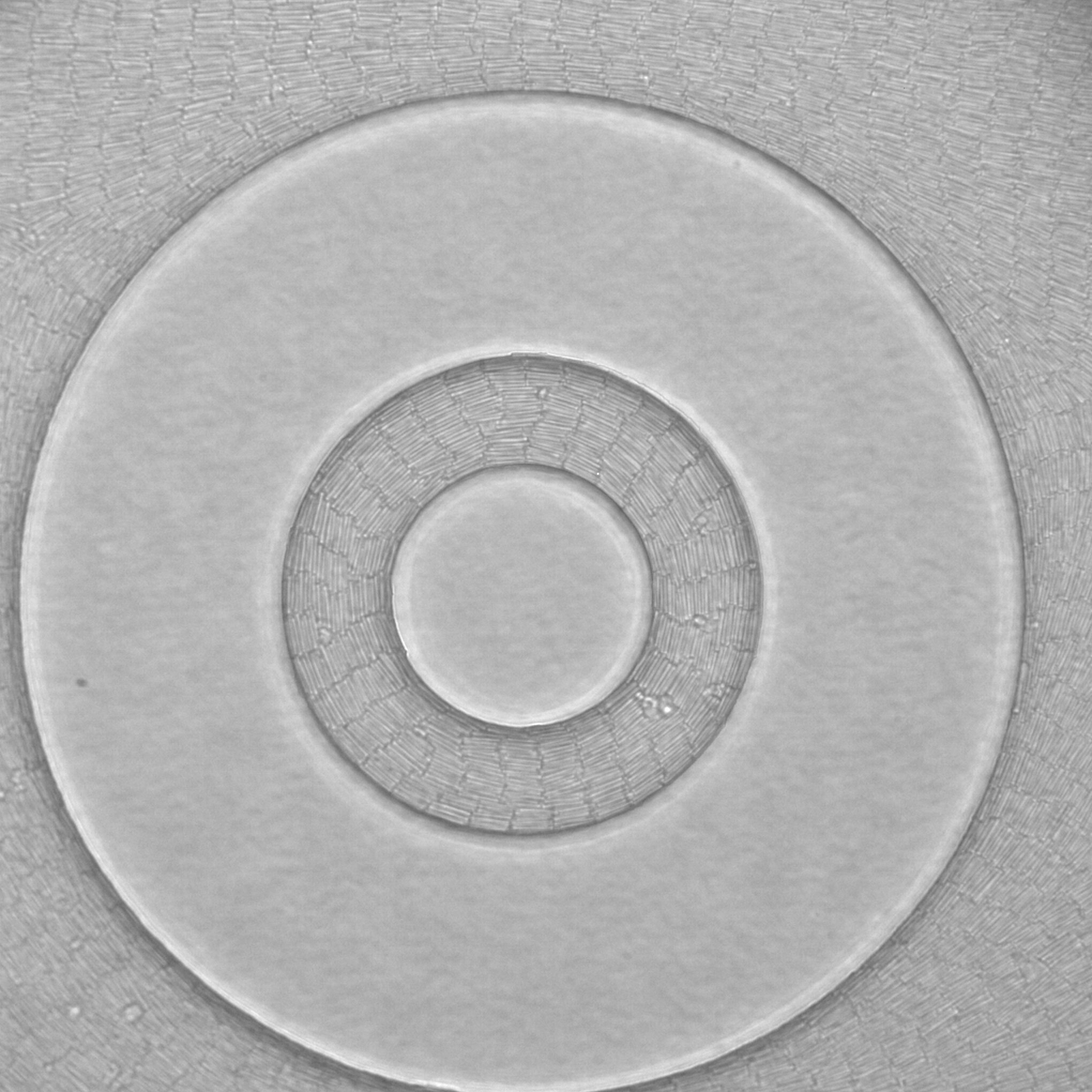

Supplement: Supplementary file 5 — Supplementary Data 2 [file 41467_2020_20842_MOESM5_ESM.zip › rawdata/size5/05_01.tif]

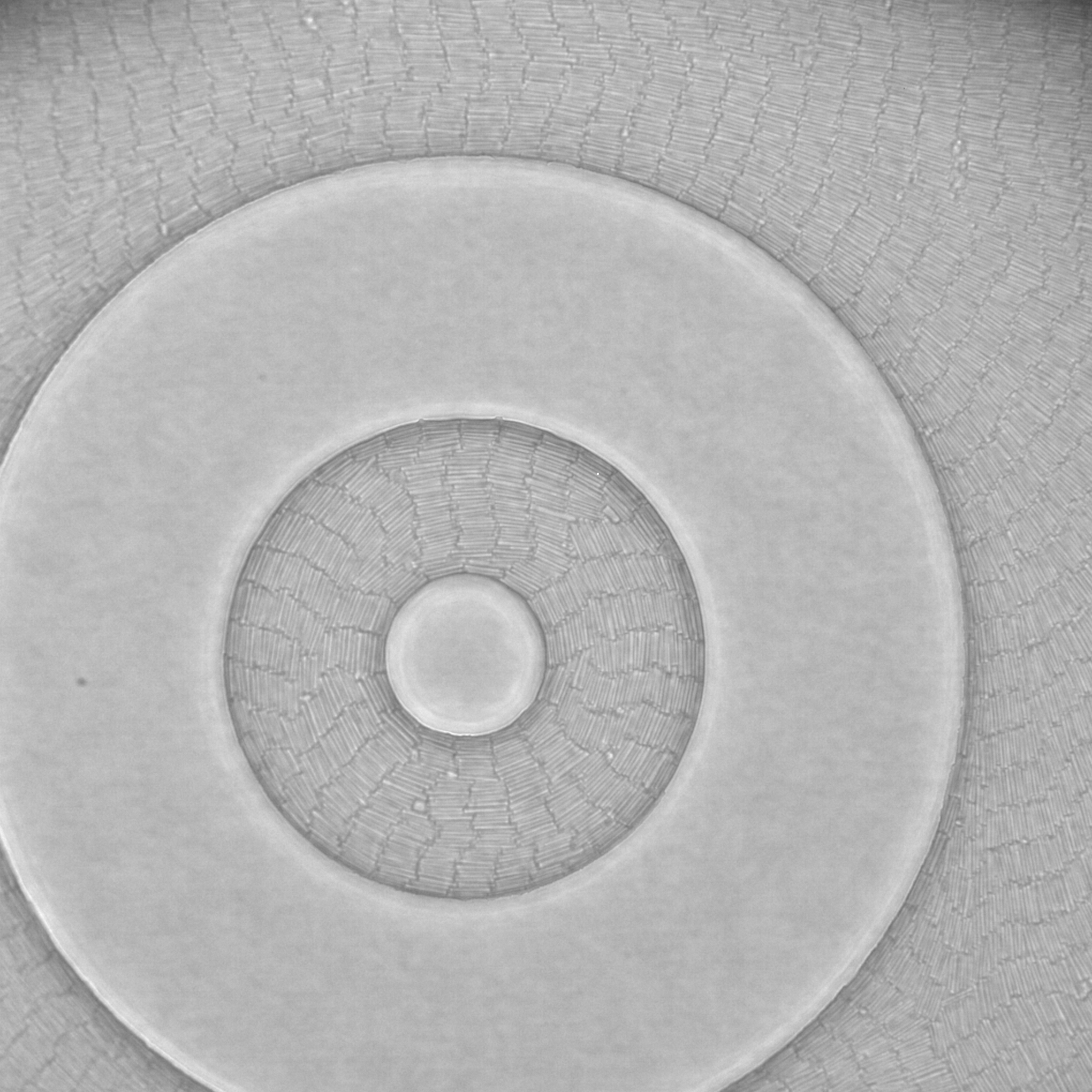

Supplement: Supplementary file 5 — Supplementary Data 2 [file 41467_2020_20842_MOESM5_ESM.zip › rawdata/size5/04_06.tif]

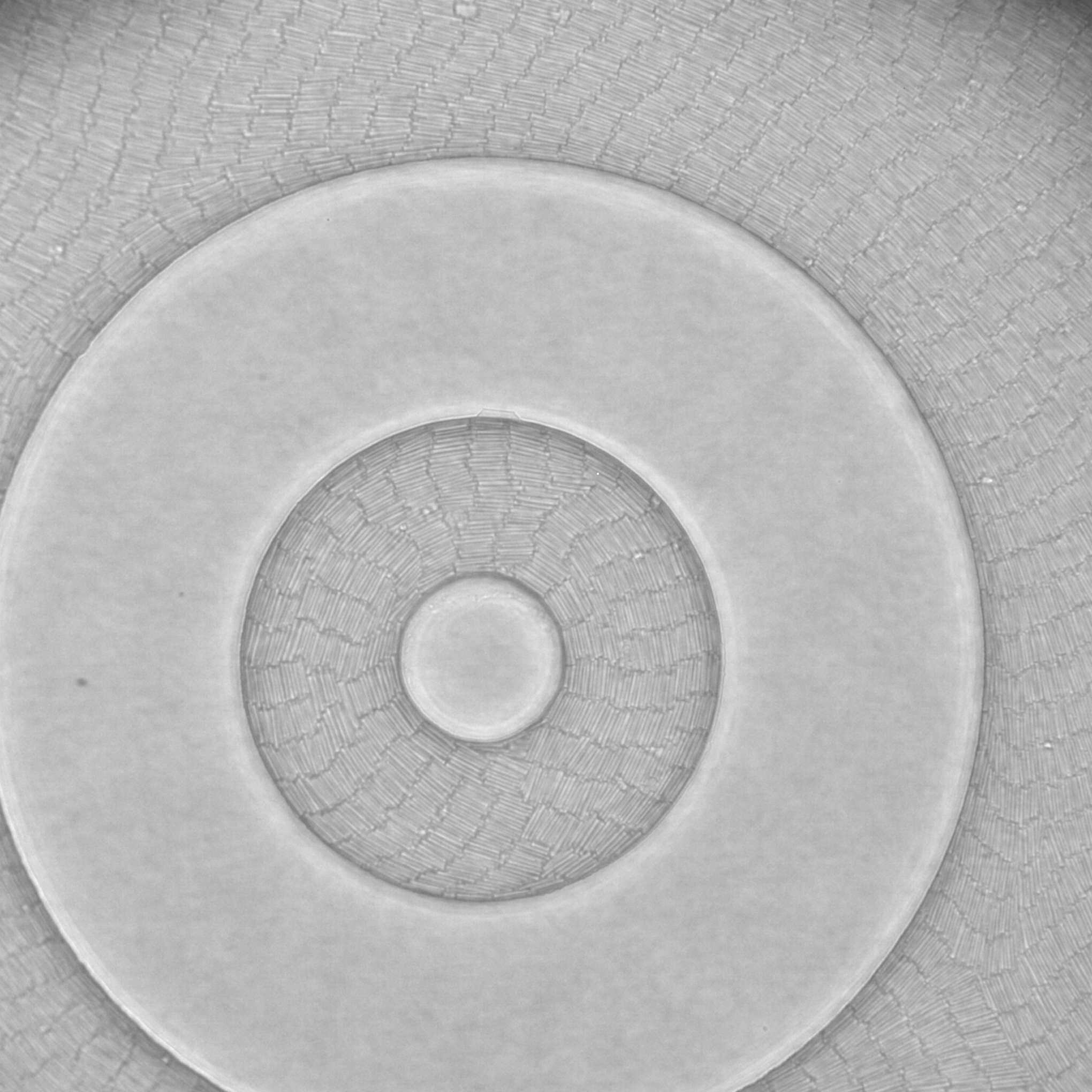

Supplement: Supplementary file 5 — Supplementary Data 2 [file 41467_2020_20842_MOESM5_ESM.zip › rawdata/size5/04_05.tif]

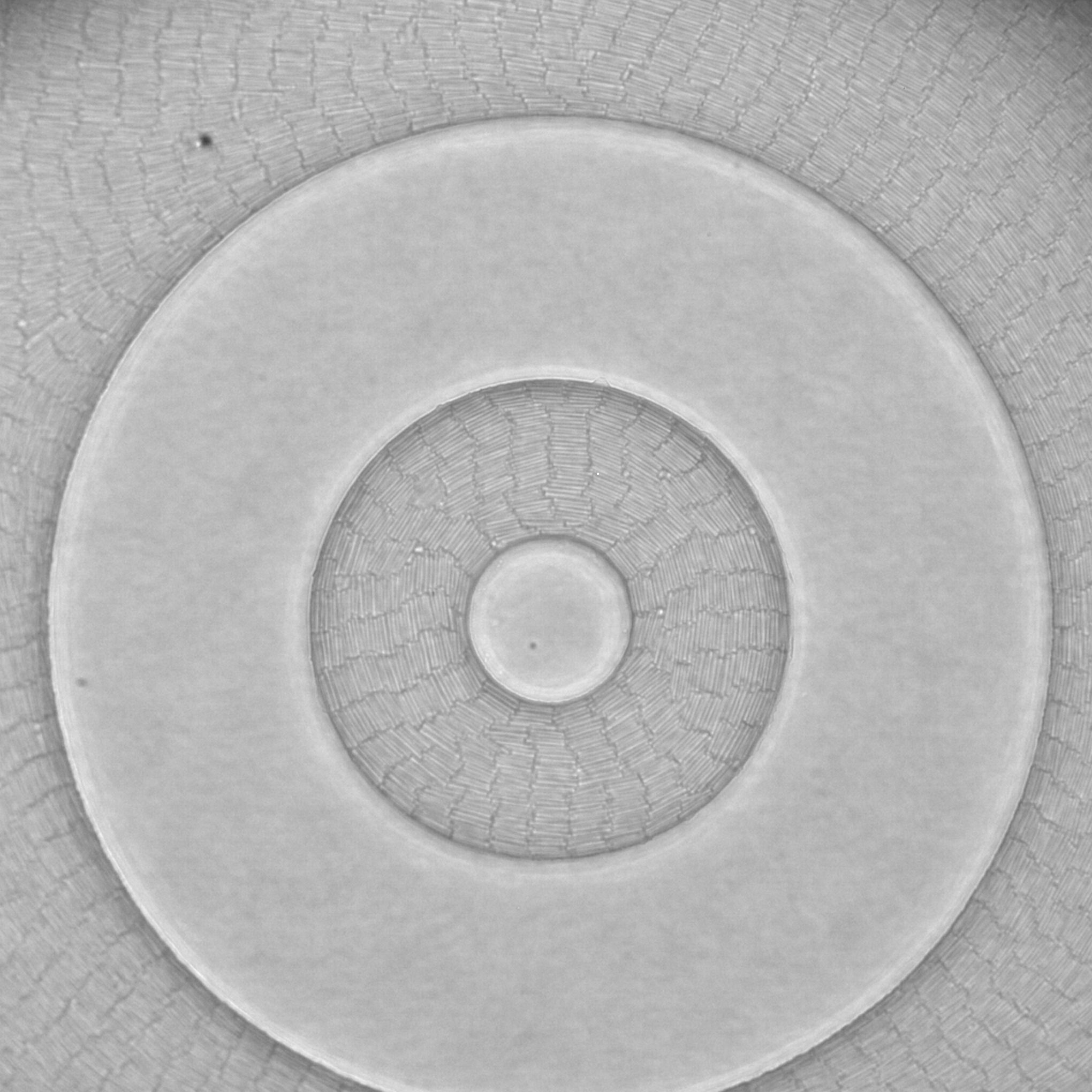

Supplement: Supplementary file 5 — Supplementary Data 2 [file 41467_2020_20842_MOESM5_ESM.zip › rawdata/size5/04_04.tif]

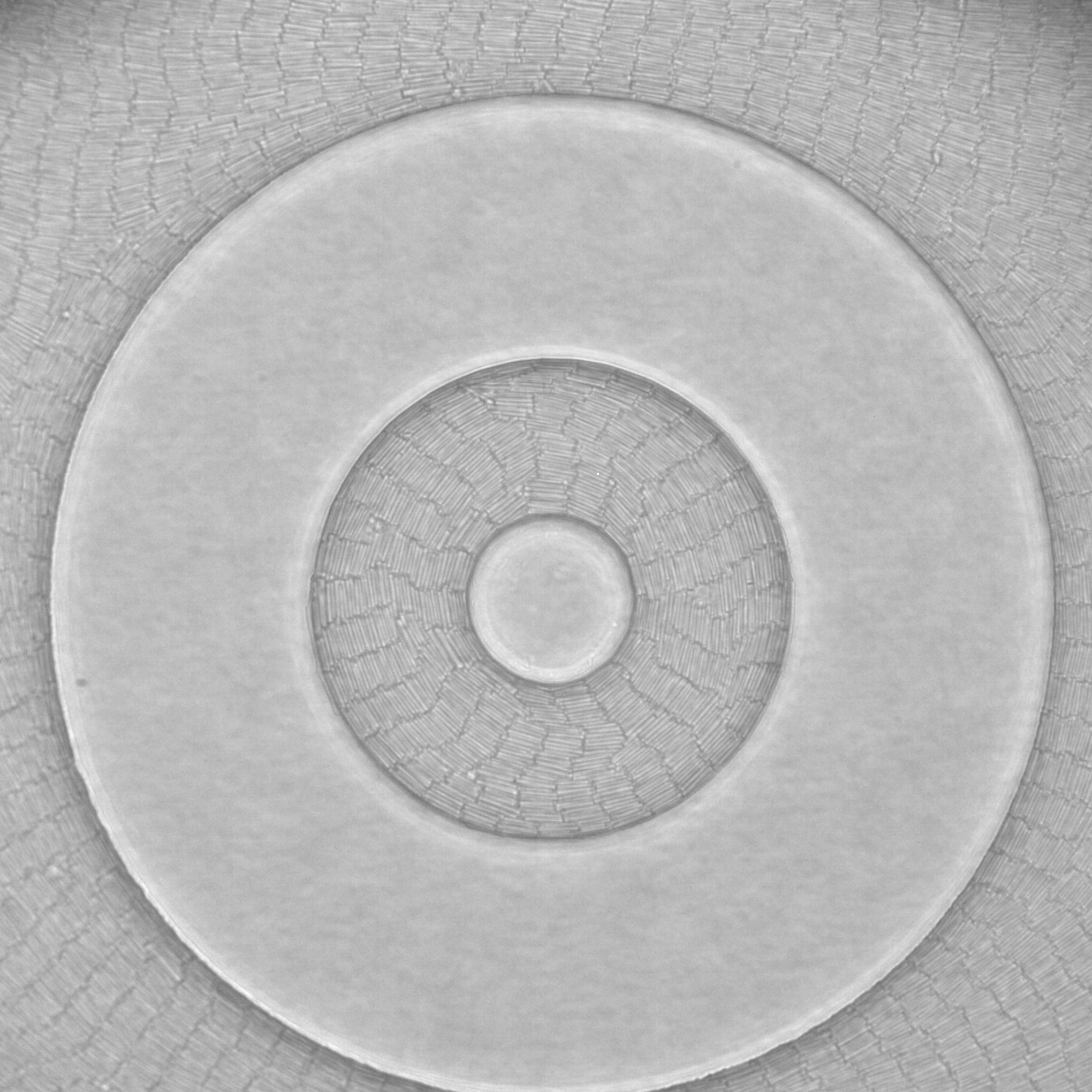

Supplement: Supplementary file 5 — Supplementary Data 2 [file 41467_2020_20842_MOESM5_ESM.zip › rawdata/size5/04_03.tif]

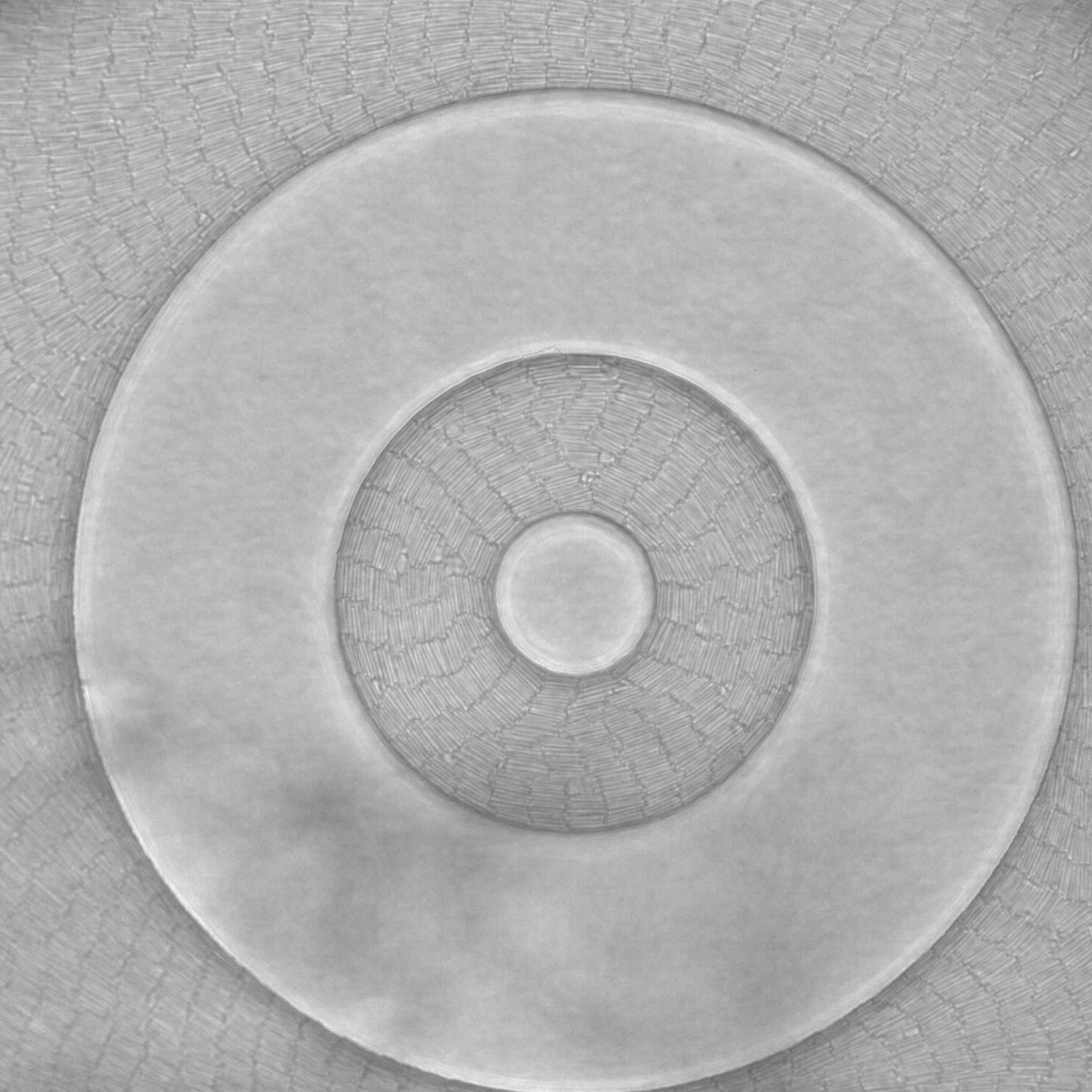

Supplement: Supplementary file 5 — Supplementary Data 2 [file 41467_2020_20842_MOESM5_ESM.zip › rawdata/size5/04_02.tif]

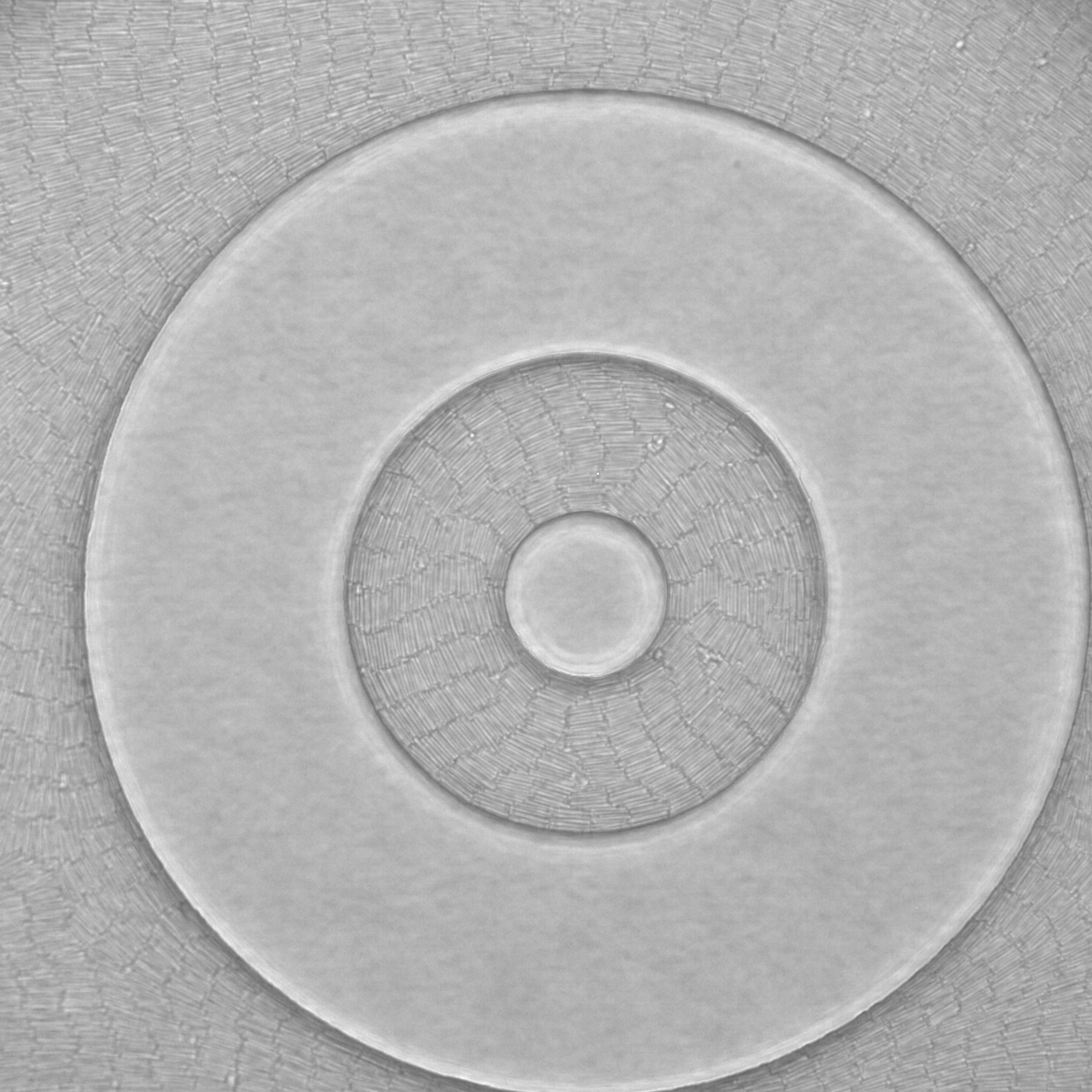

Supplement: Supplementary file 5 — Supplementary Data 2 [file 41467_2020_20842_MOESM5_ESM.zip › rawdata/size5/04_01.tif]

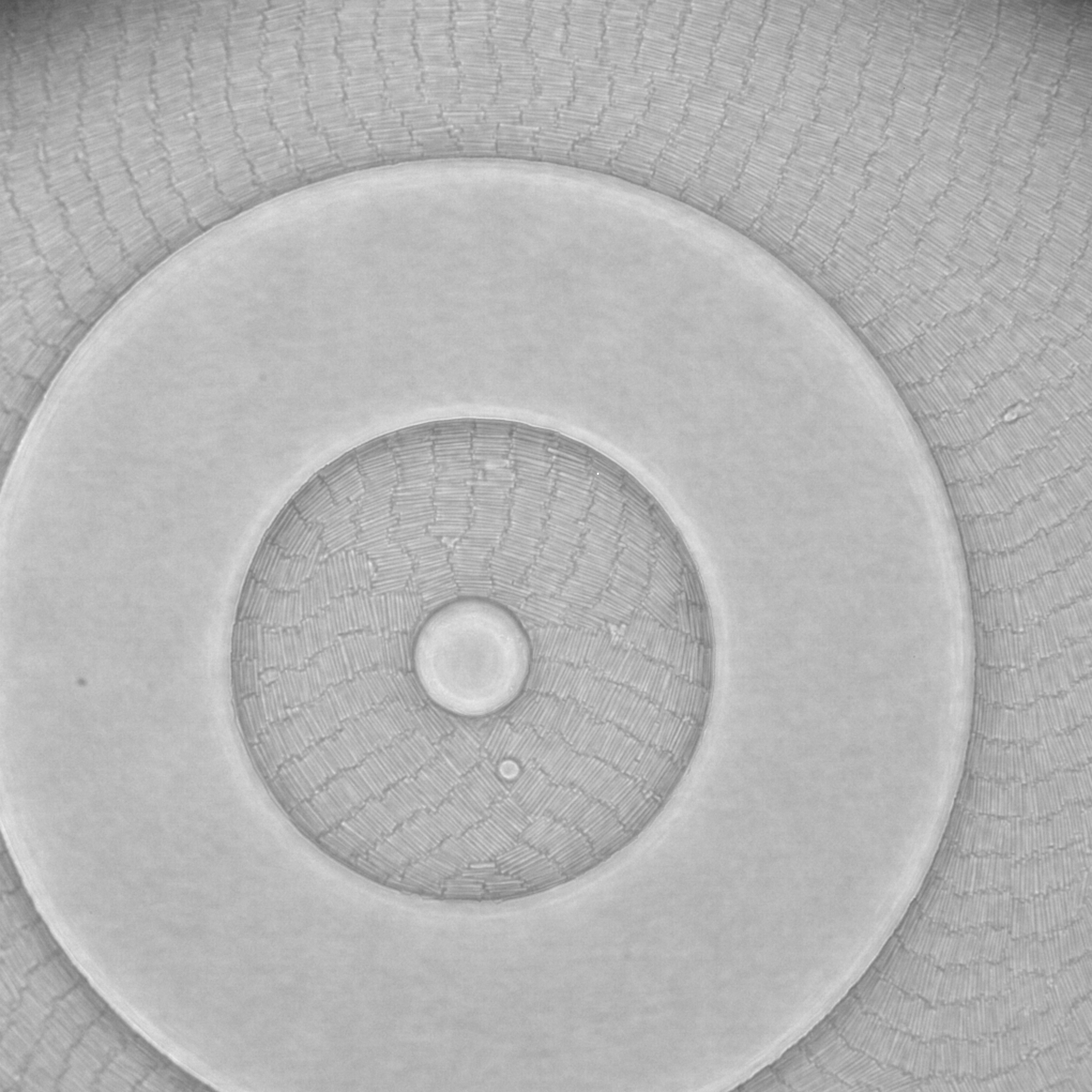

Supplement: Supplementary file 5 — Supplementary Data 2 [file 41467_2020_20842_MOESM5_ESM.zip › rawdata/size5/03_06.tif]

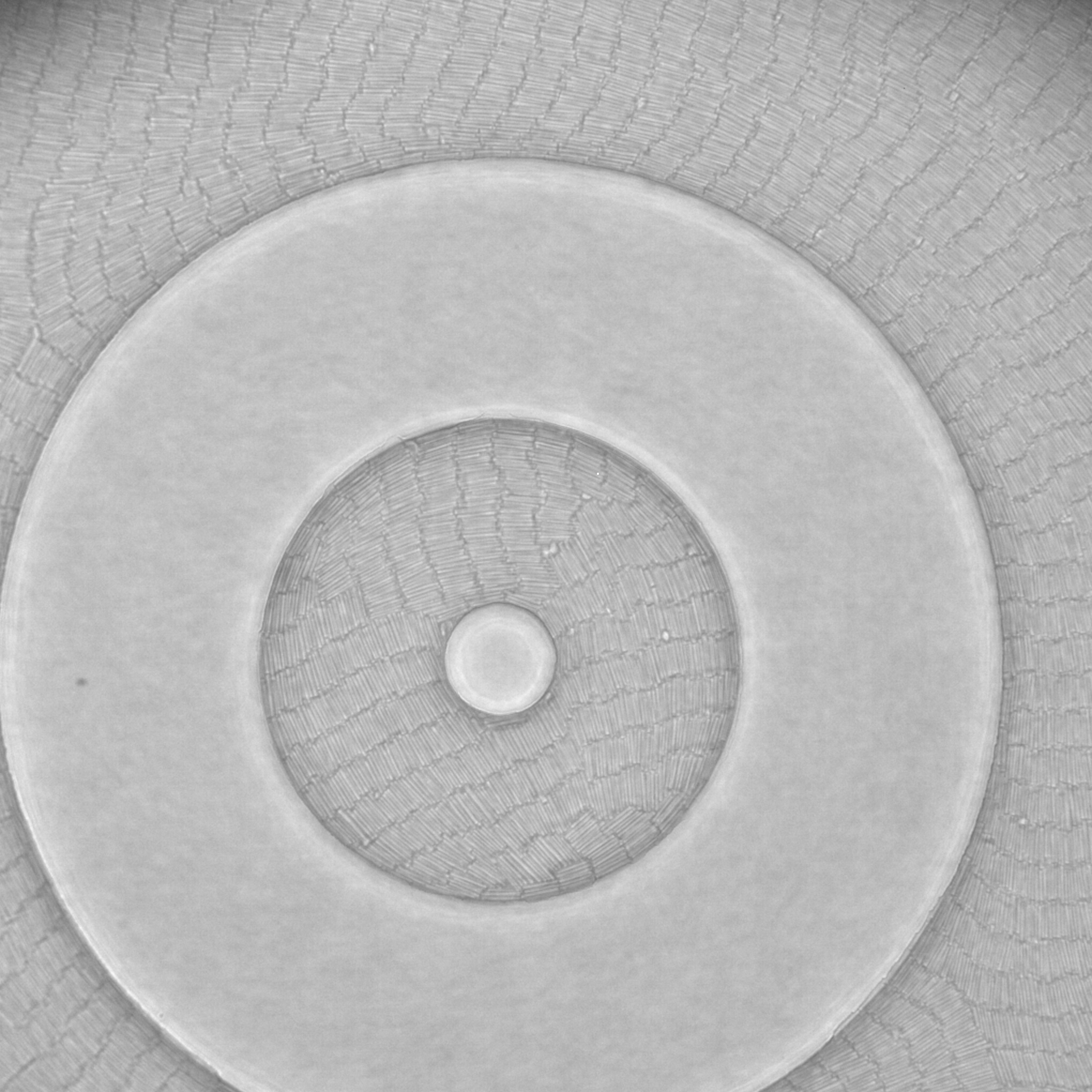

Supplement: Supplementary file 5 — Supplementary Data 2 [file 41467_2020_20842_MOESM5_ESM.zip › rawdata/size5/03_05.tif]

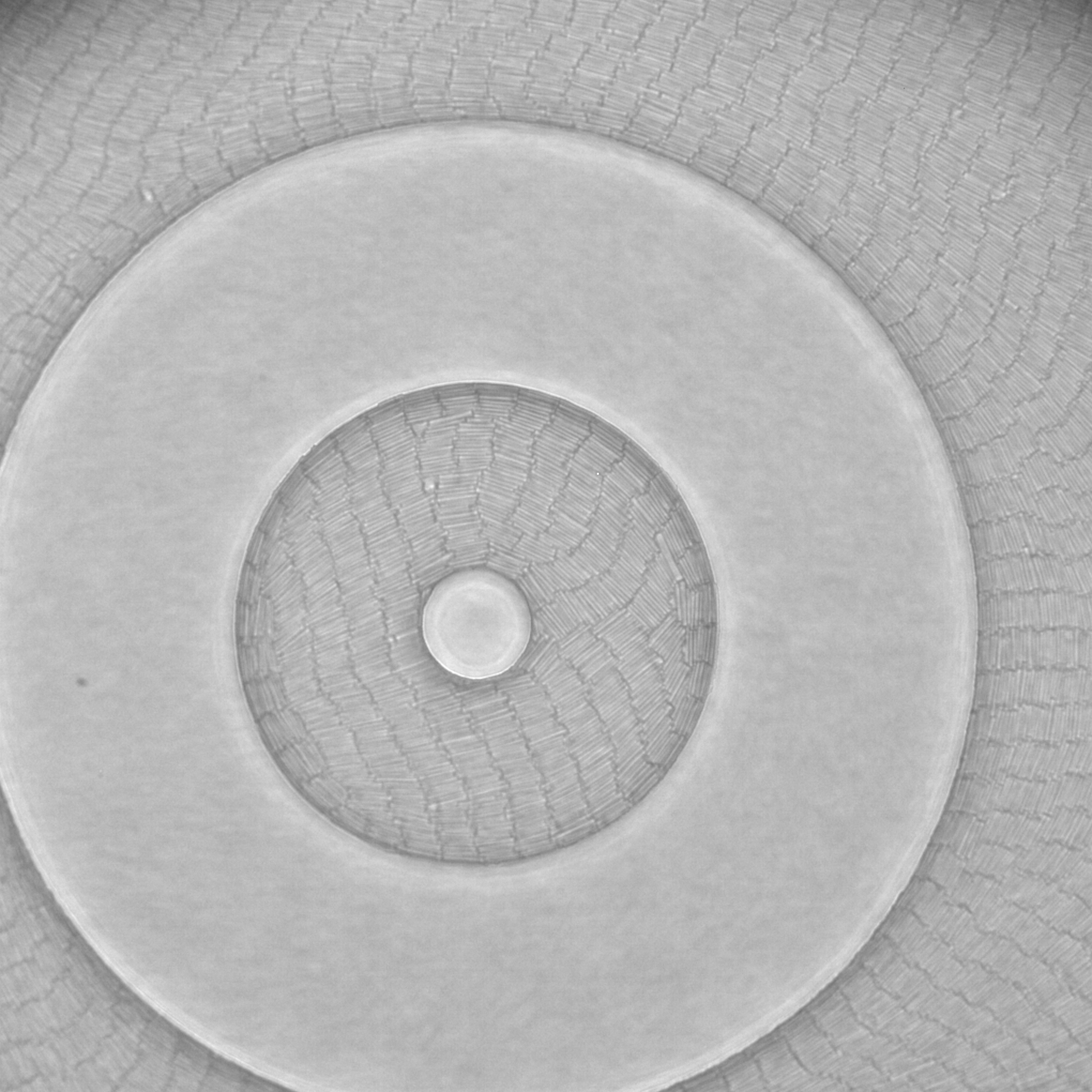

Supplement: Supplementary file 5 — Supplementary Data 2 [file 41467_2020_20842_MOESM5_ESM.zip › rawdata/size5/03_04.tif]

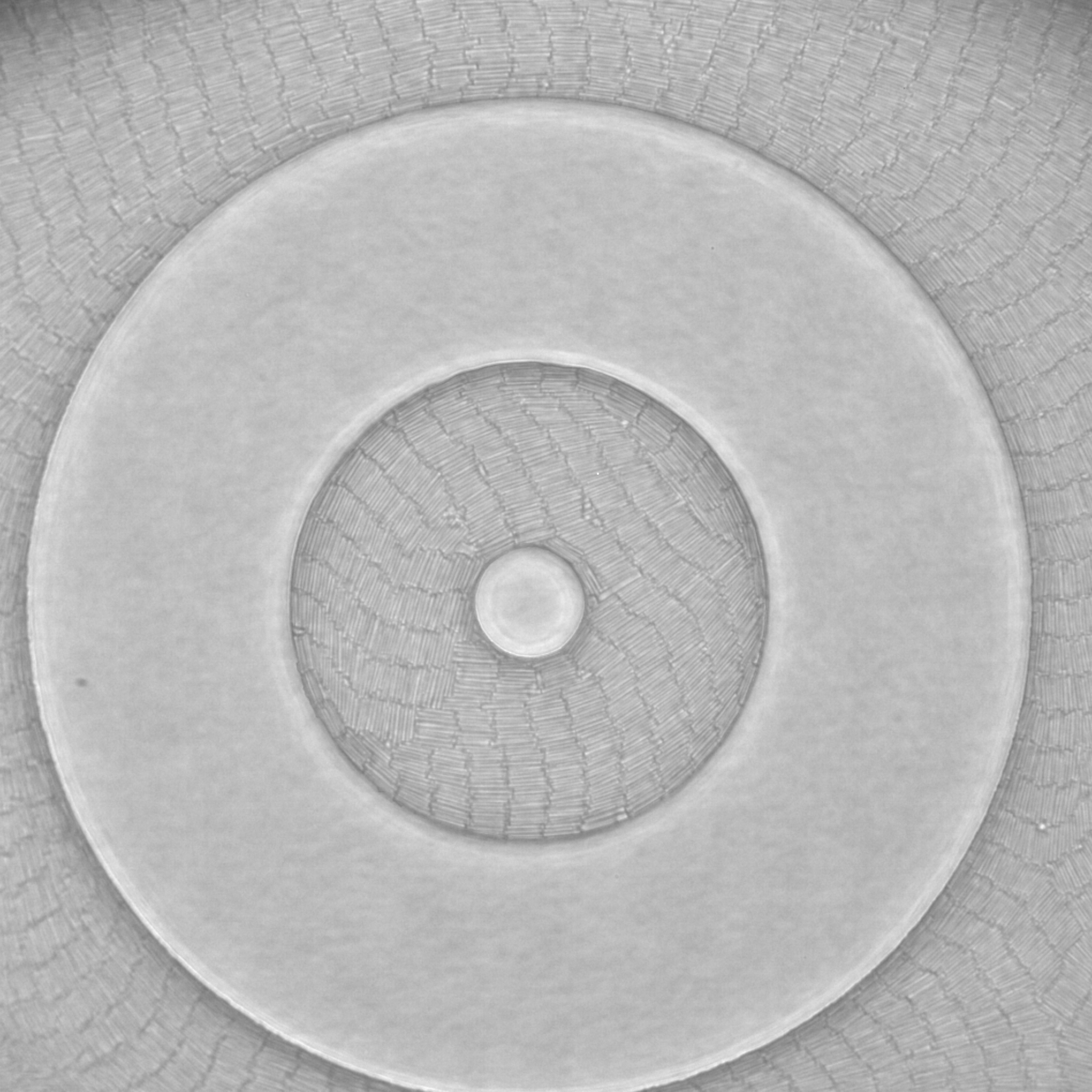

Supplement: Supplementary file 5 — Supplementary Data 2 [file 41467_2020_20842_MOESM5_ESM.zip › rawdata/size5/03_03.tif]

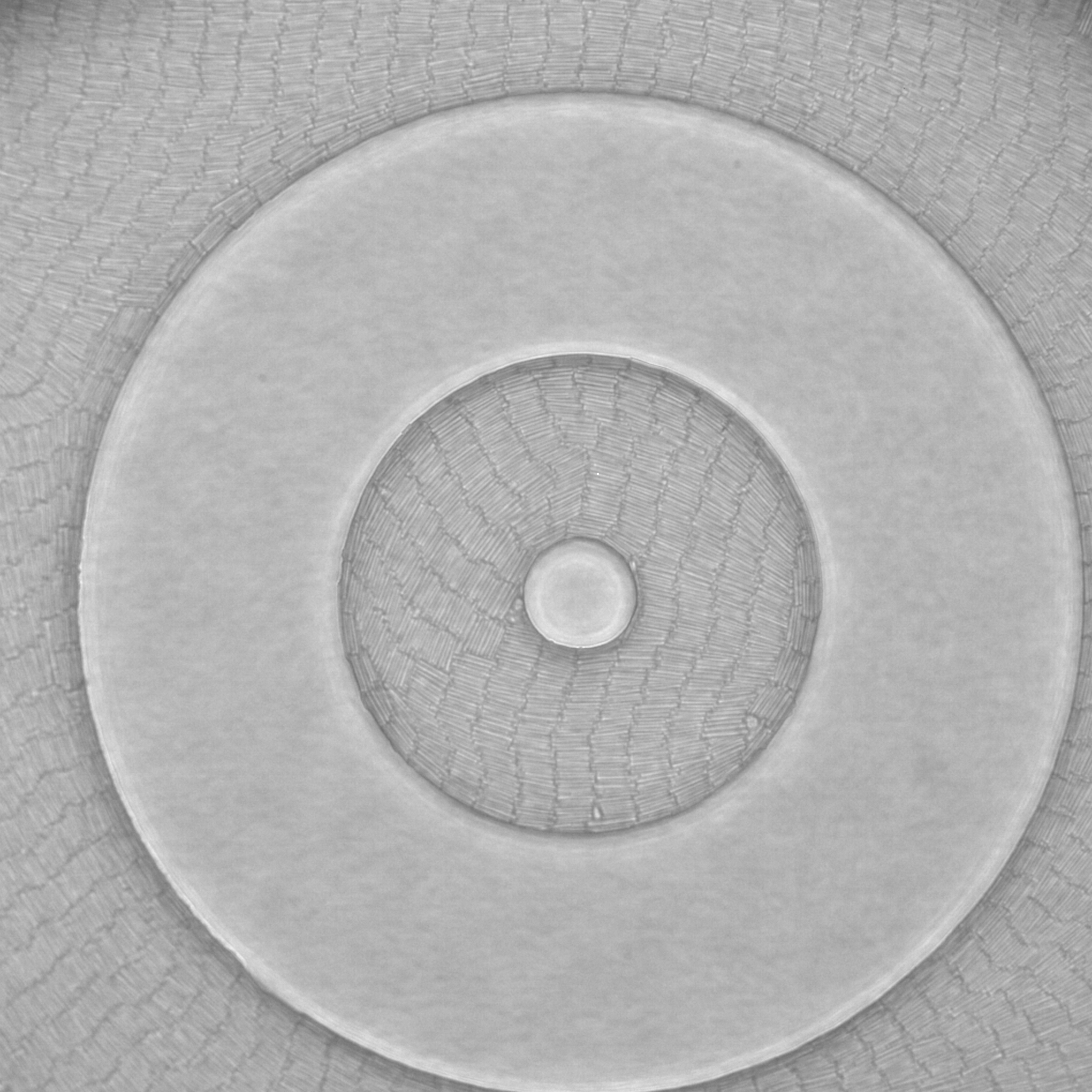

Supplement: Supplementary file 5 — Supplementary Data 2 [file 41467_2020_20842_MOESM5_ESM.zip › rawdata/size5/03_02.tif]

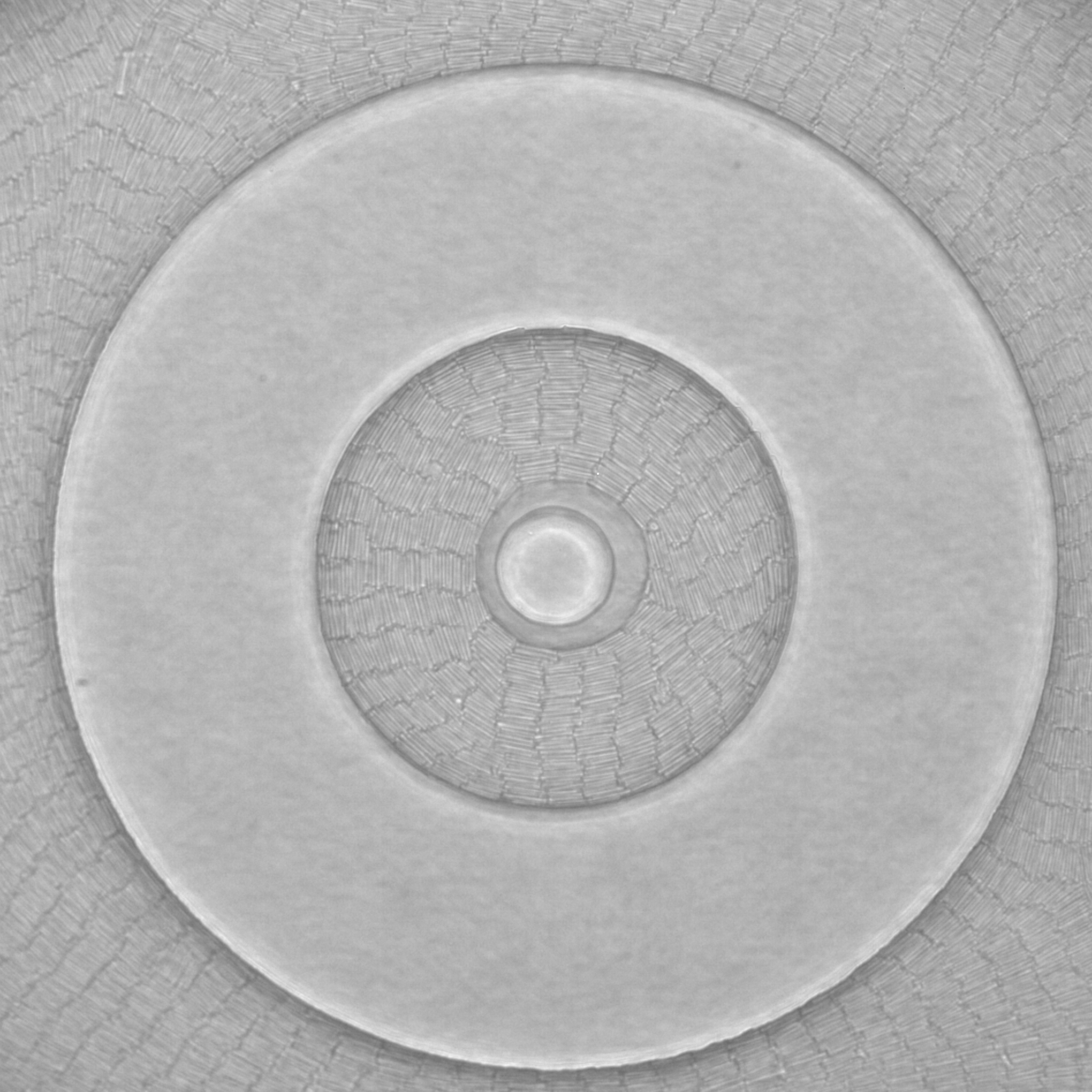

Supplement: Supplementary file 5 — Supplementary Data 2 [file 41467_2020_20842_MOESM5_ESM.zip › rawdata/size5/03_01.tif]

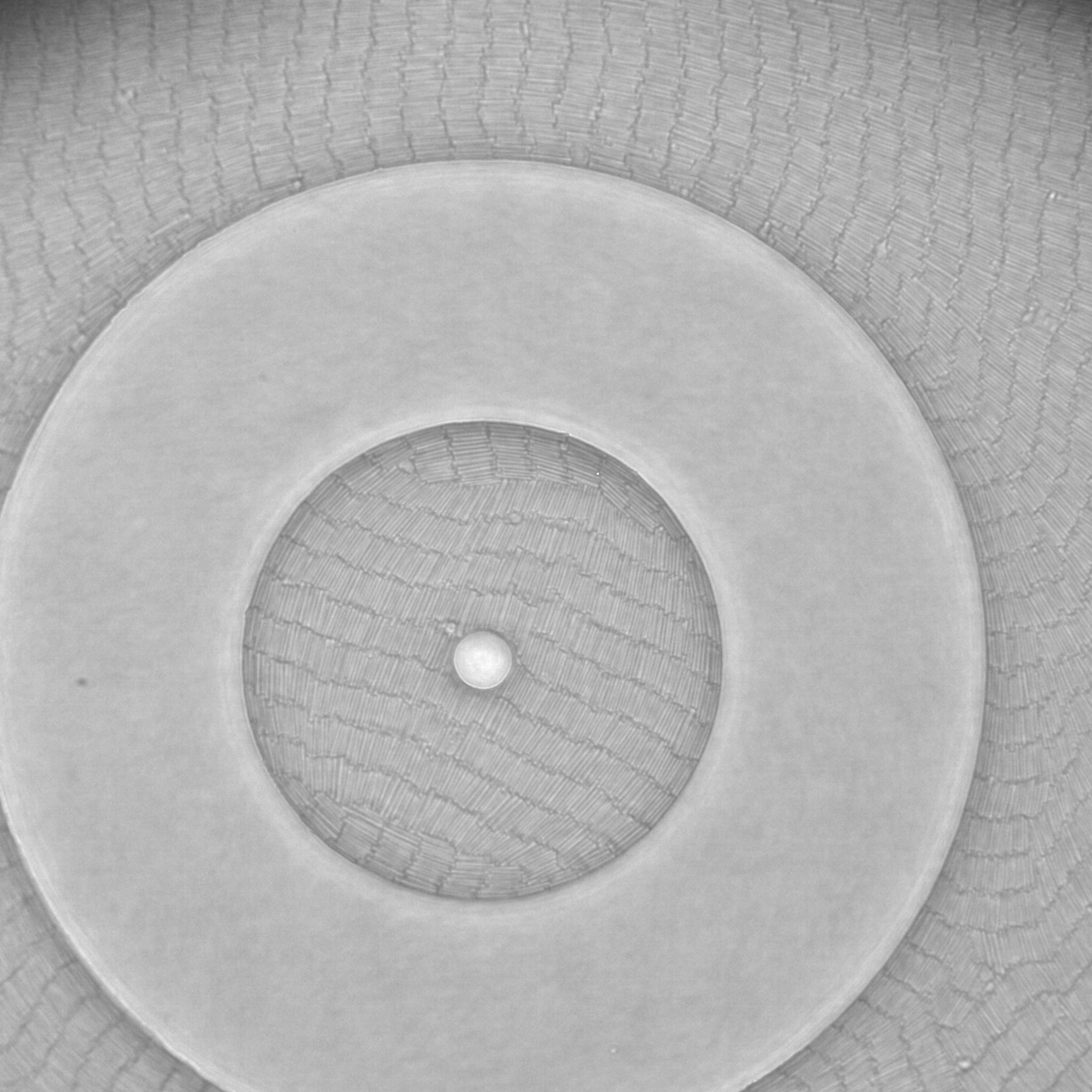

Supplement: Supplementary file 5 — Supplementary Data 2 [file 41467_2020_20842_MOESM5_ESM.zip › rawdata/size5/02_06.tif]

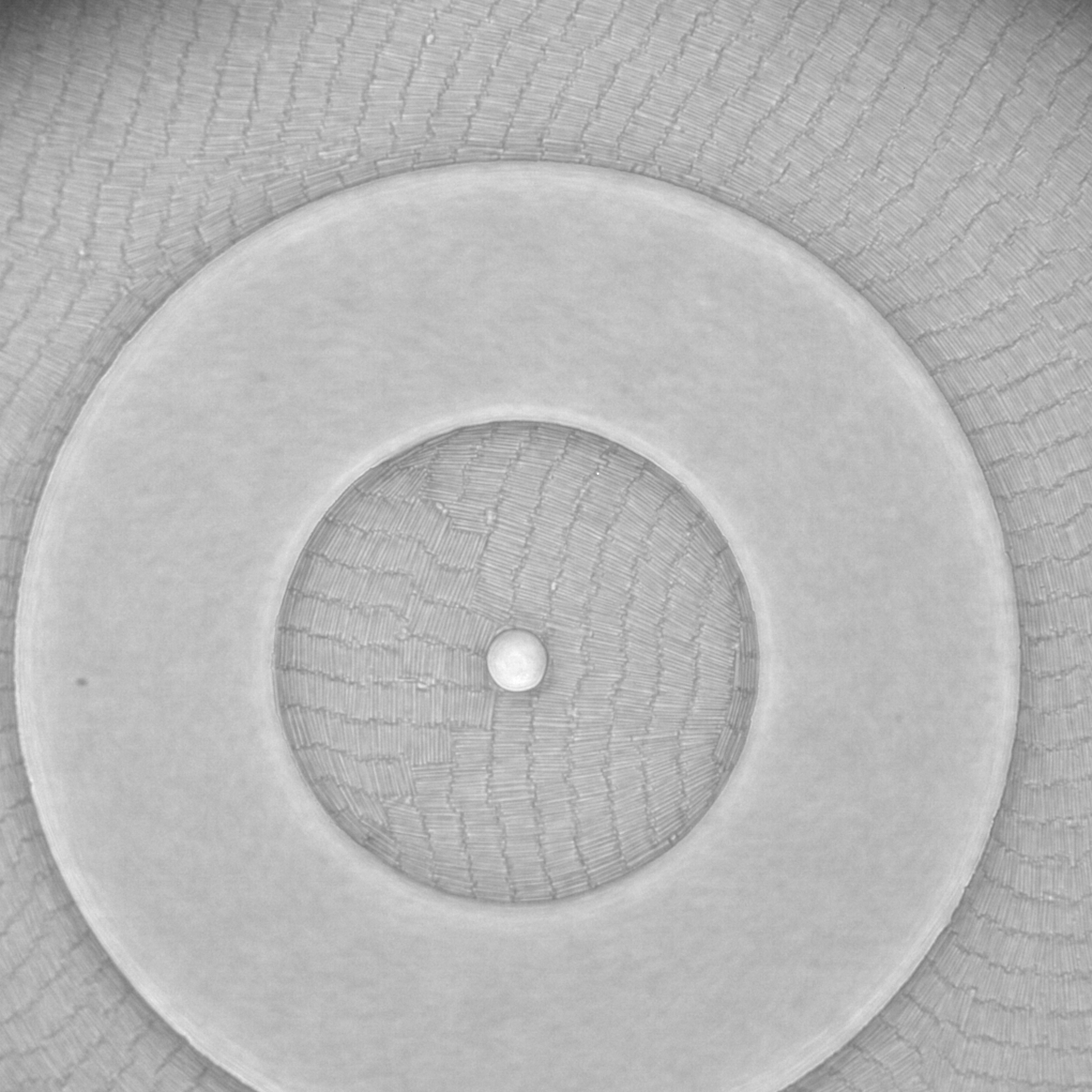

Supplement: Supplementary file 5 — Supplementary Data 2 [file 41467_2020_20842_MOESM5_ESM.zip › rawdata/size5/02_05.tif]

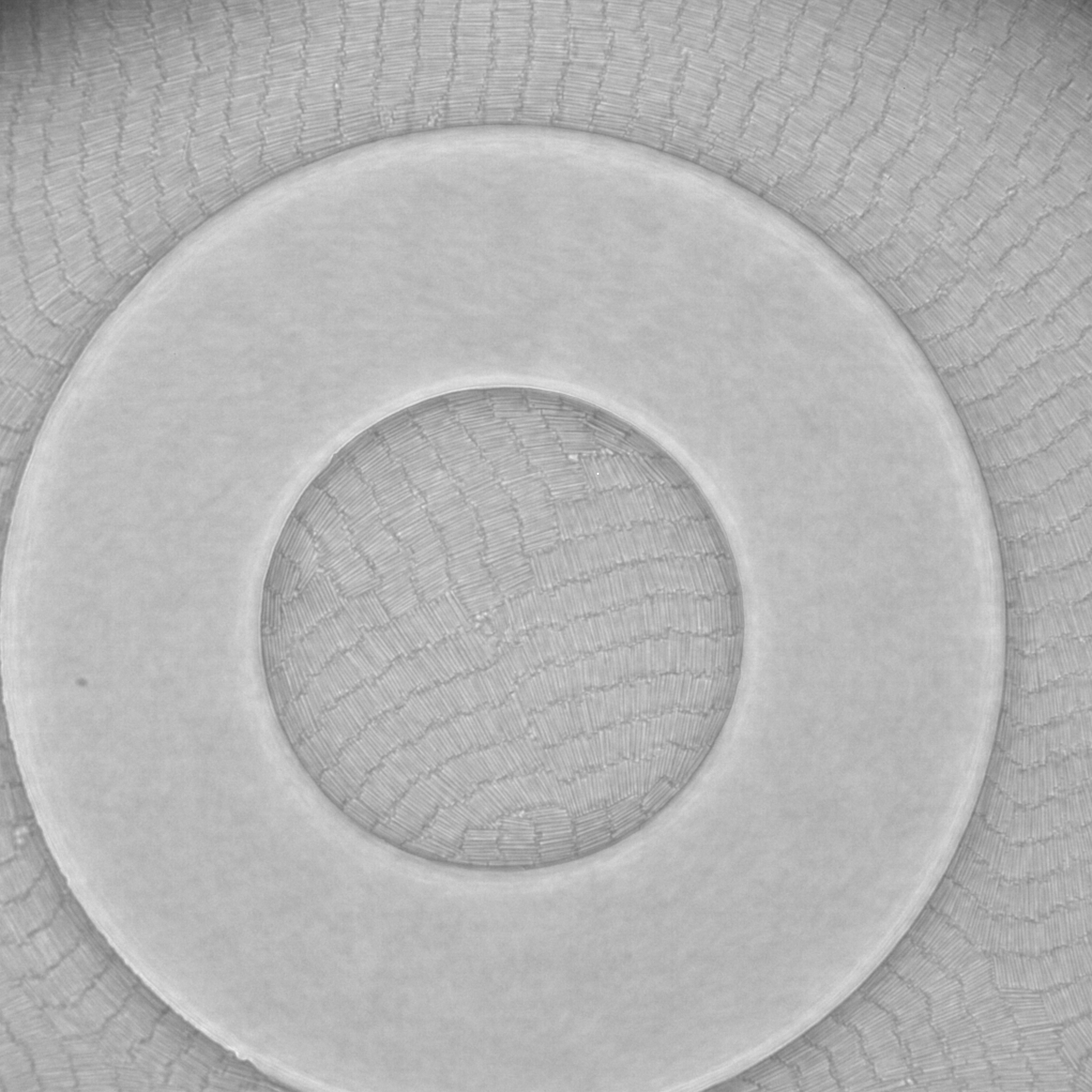

Supplement: Supplementary file 5 — Supplementary Data 2 [file 41467_2020_20842_MOESM5_ESM.zip › rawdata/size5/02_04.tif]

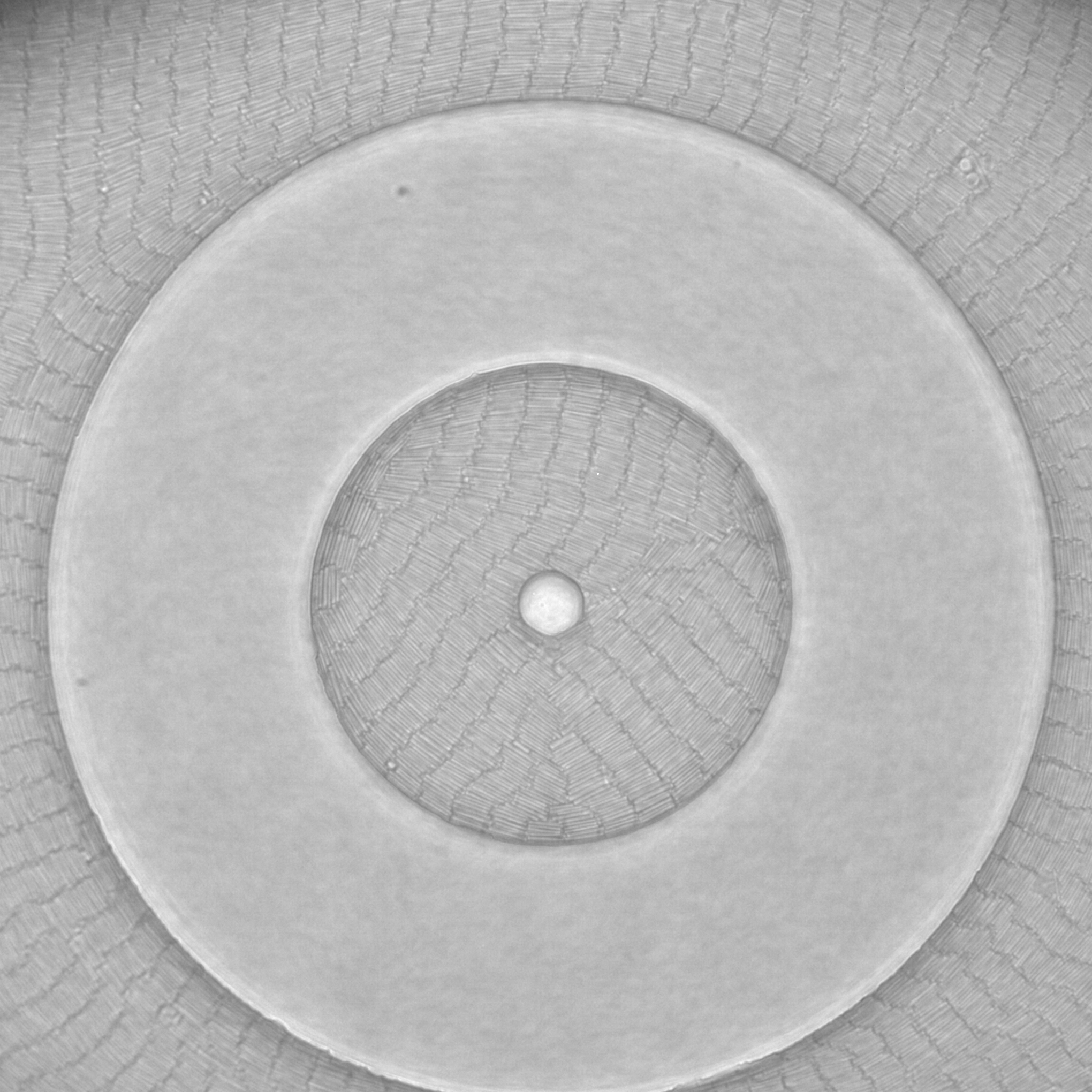

Supplement: Supplementary file 5 — Supplementary Data 2 [file 41467_2020_20842_MOESM5_ESM.zip › rawdata/size5/02_03.tif]

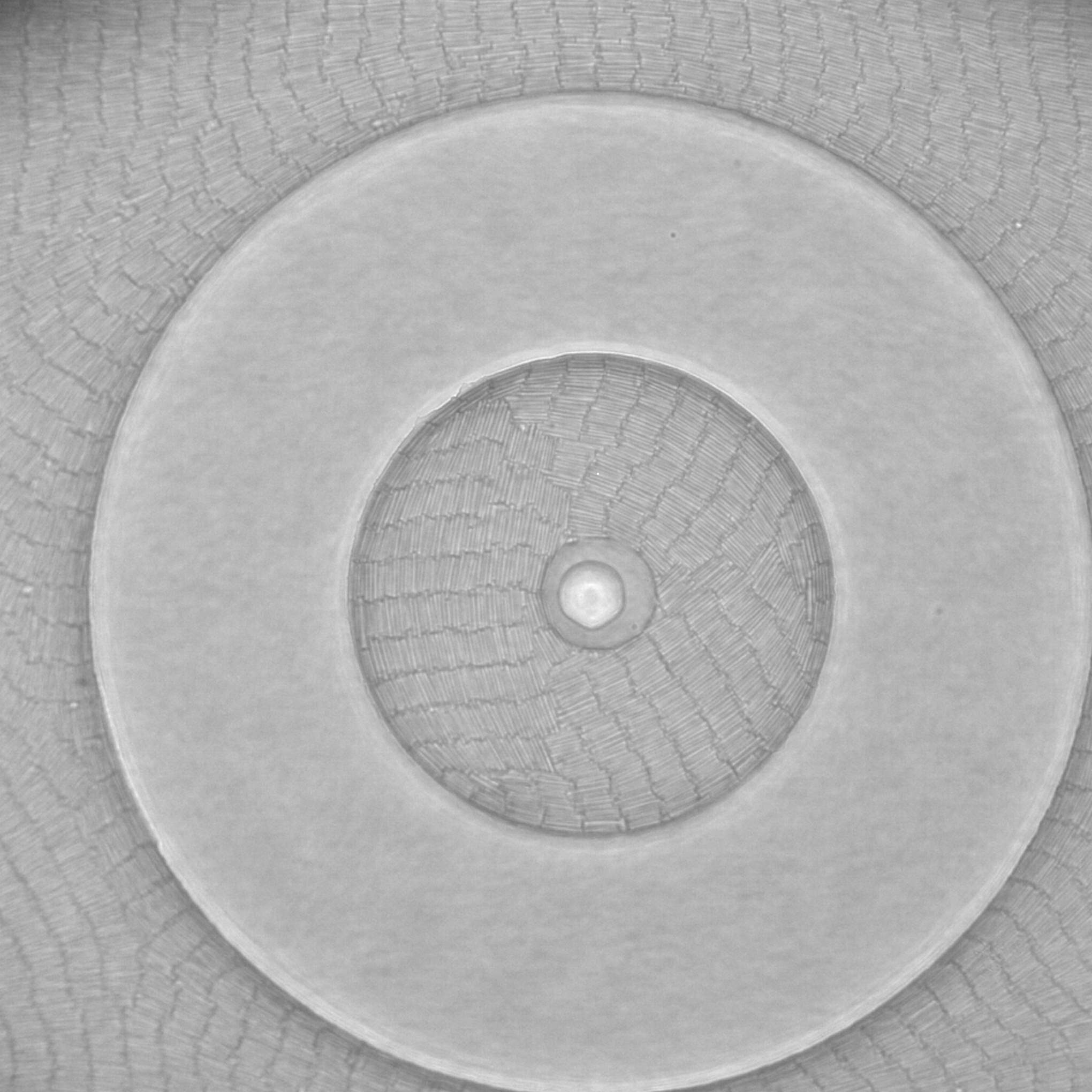

Supplement: Supplementary file 5 — Supplementary Data 2 [file 41467_2020_20842_MOESM5_ESM.zip › rawdata/size5/02_02.tif]

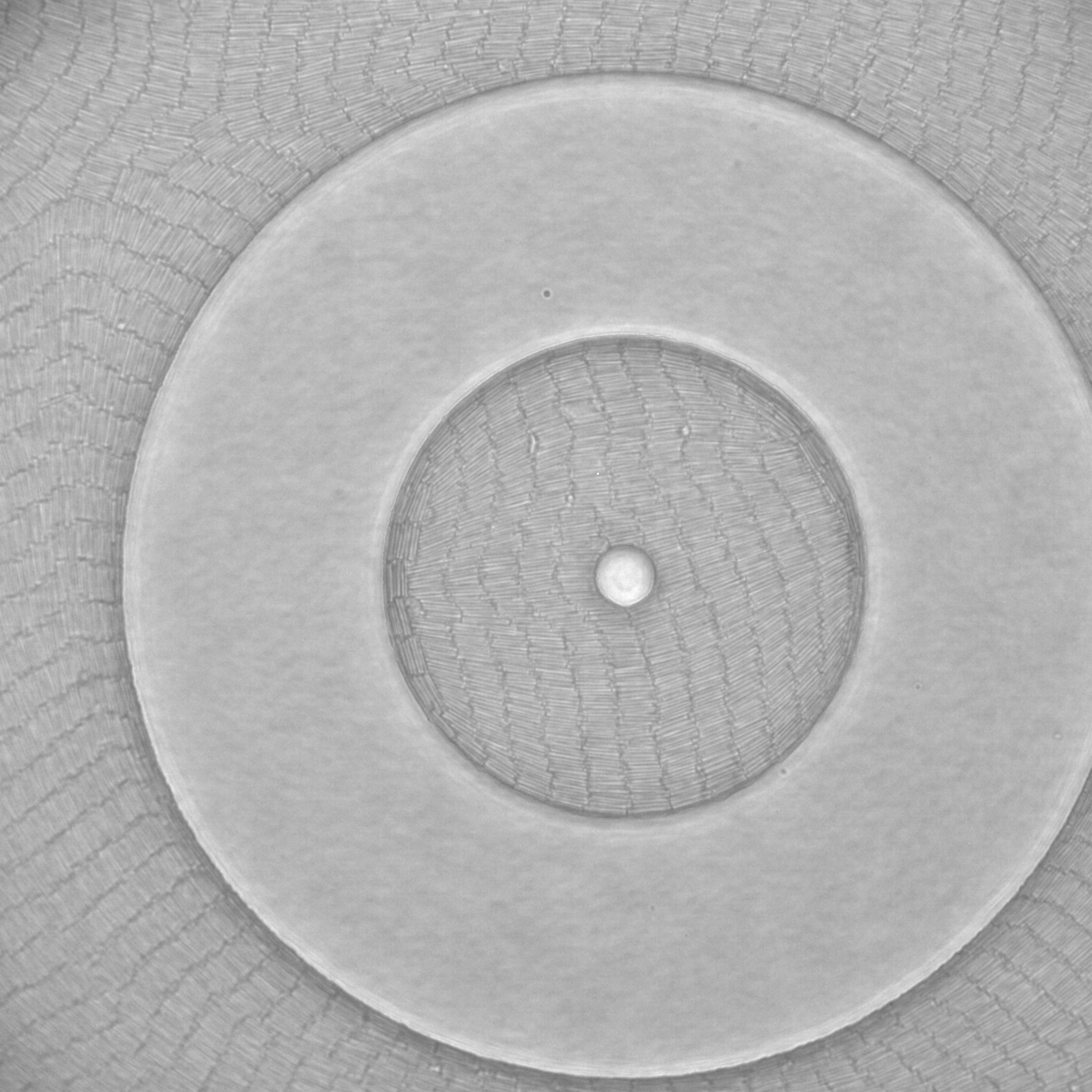

Supplement: Supplementary file 5 — Supplementary Data 2 [file 41467_2020_20842_MOESM5_ESM.zip › rawdata/size5/02_01.tif]

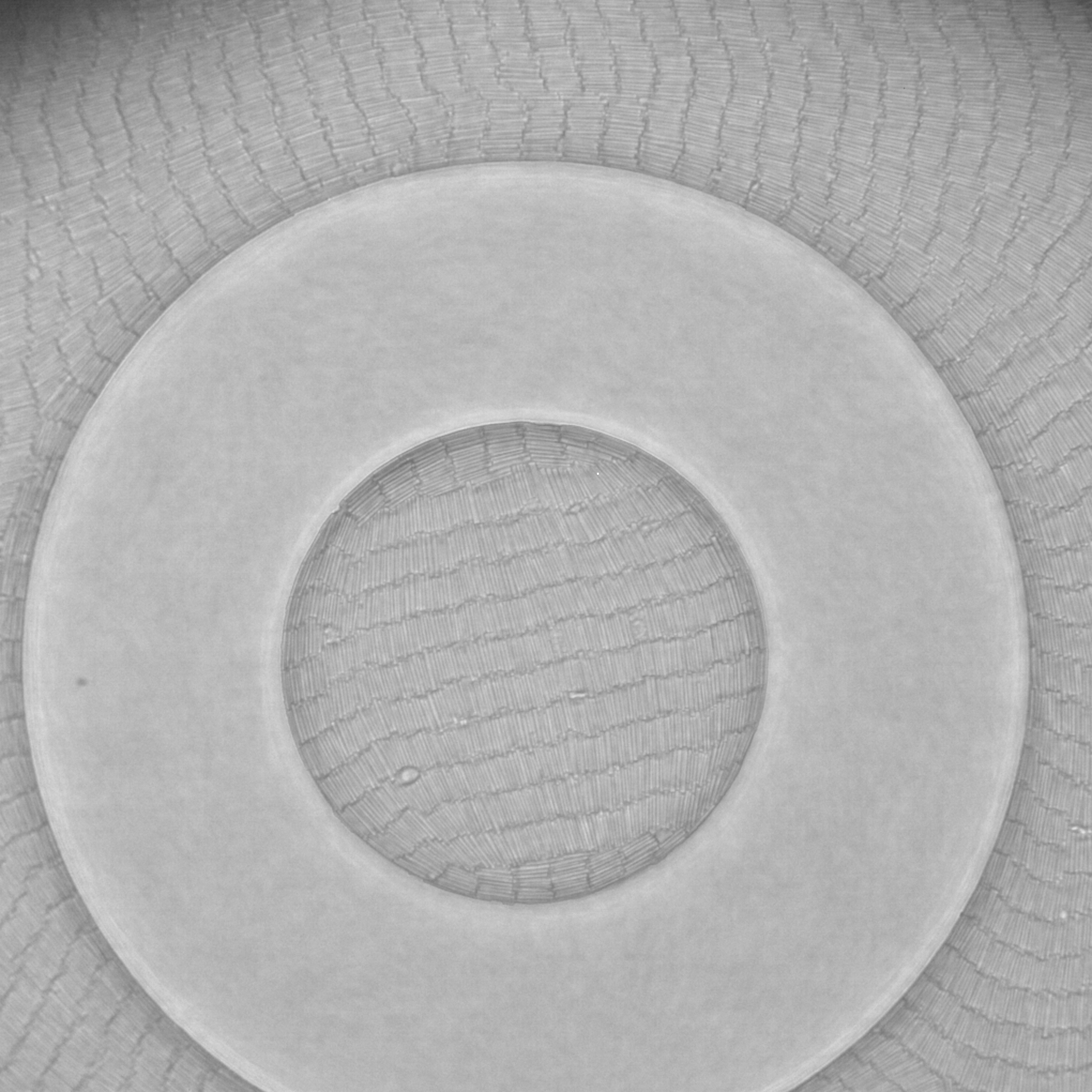

Supplement: Supplementary file 5 — Supplementary Data 2 [file 41467_2020_20842_MOESM5_ESM.zip › rawdata/size5/01_06.tif]

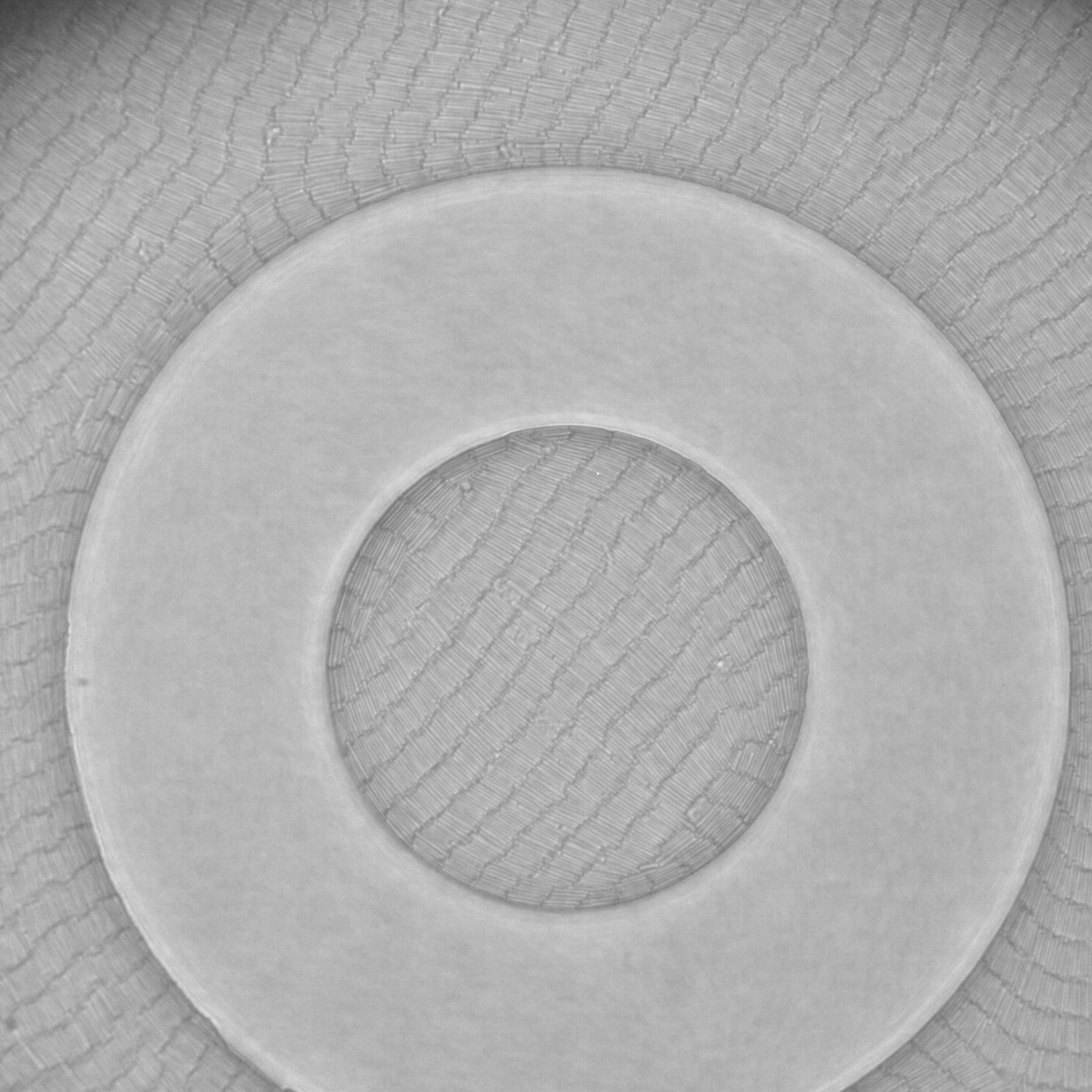

Supplement: Supplementary file 5 — Supplementary Data 2 [file 41467_2020_20842_MOESM5_ESM.zip › rawdata/size5/01_05.tif]

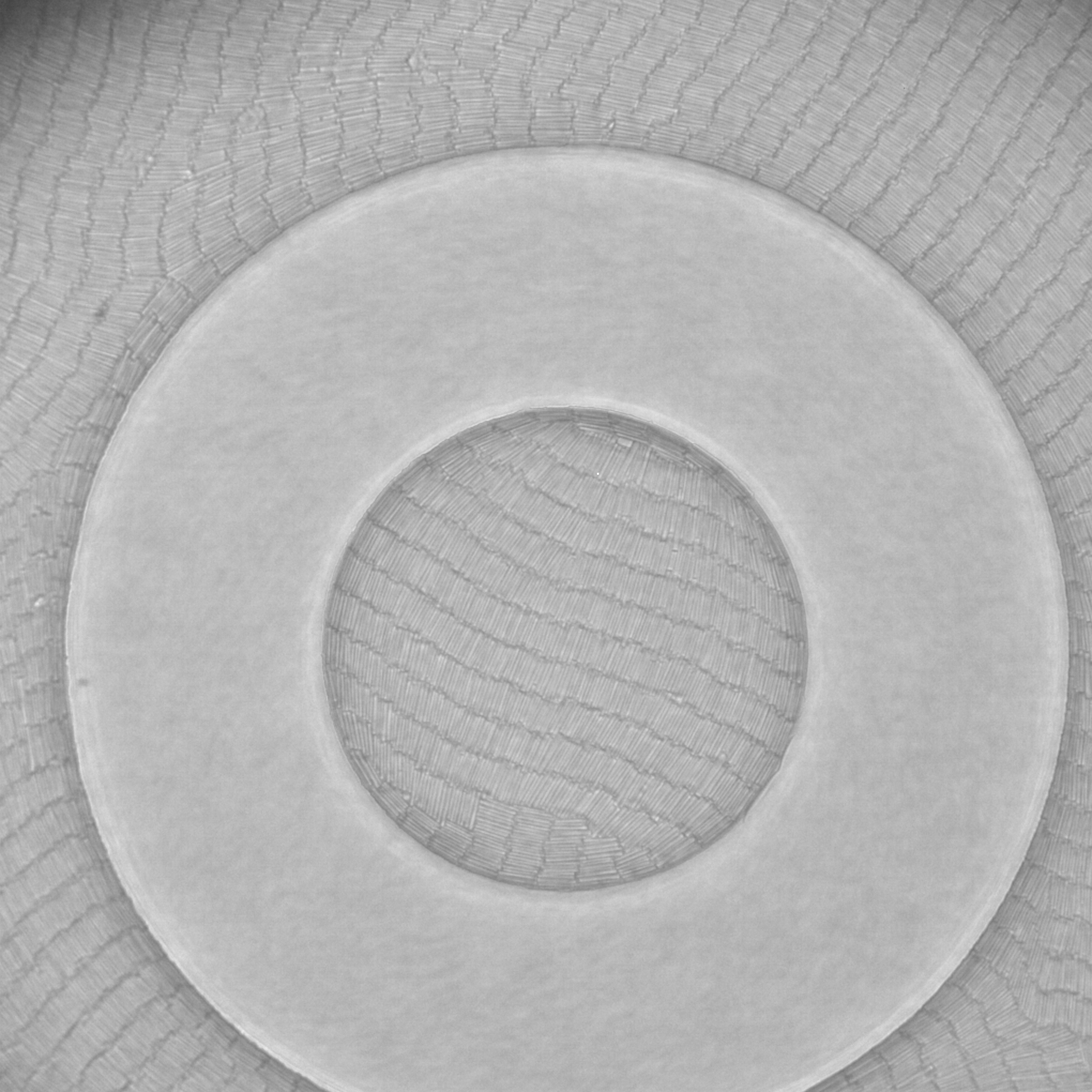

Supplement: Supplementary file 5 — Supplementary Data 2 [file 41467_2020_20842_MOESM5_ESM.zip › rawdata/size5/01_04.tif]

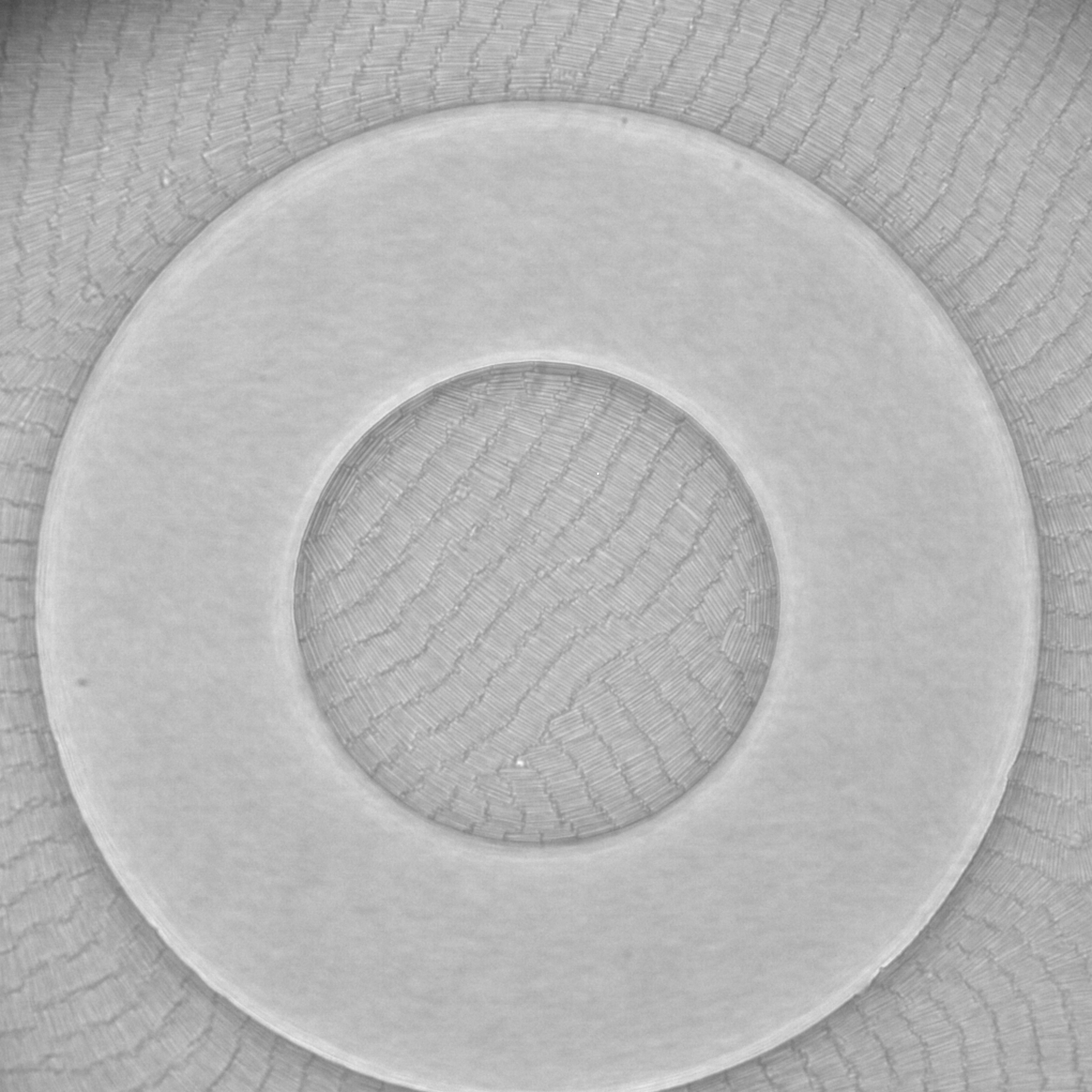

Supplement: Supplementary file 5 — Supplementary Data 2 [file 41467_2020_20842_MOESM5_ESM.zip › rawdata/size5/01_03.tif]

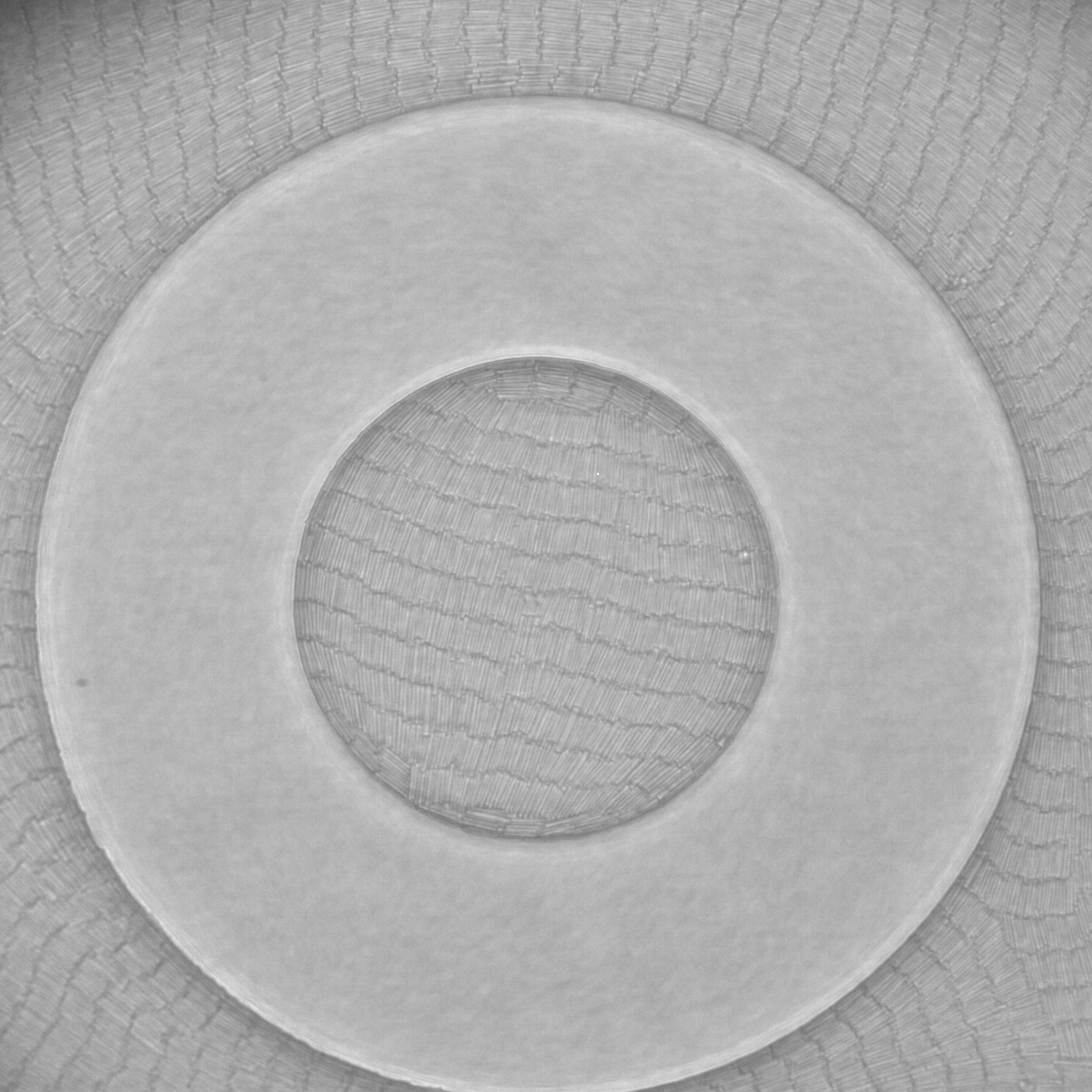

Supplement: Supplementary file 5 — Supplementary Data 2 [file 41467_2020_20842_MOESM5_ESM.zip › rawdata/size5/01_02.tif]

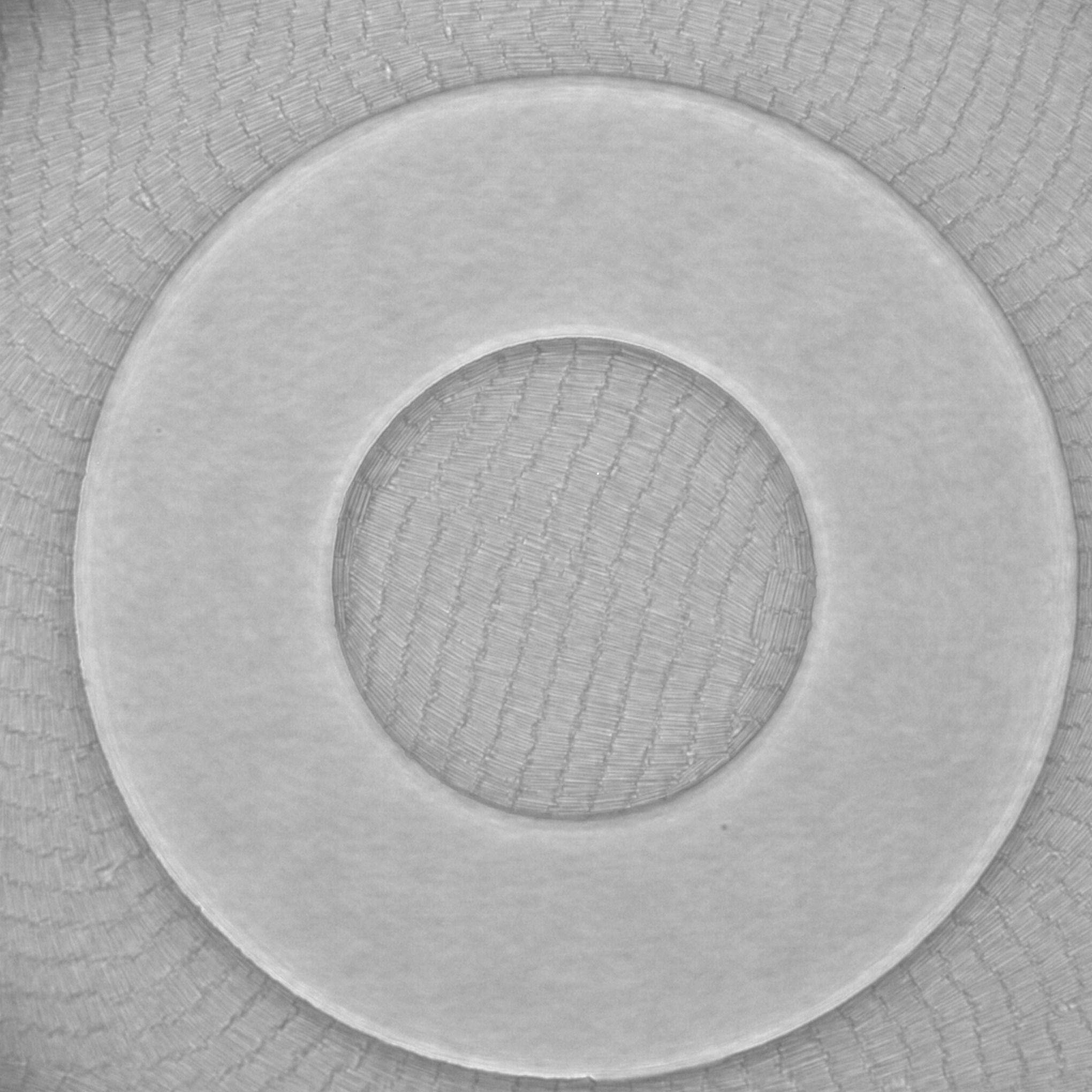

Supplement: Supplementary file 5 — Supplementary Data 2 [file 41467_2020_20842_MOESM5_ESM.zip › rawdata/size5/01_01.tif]

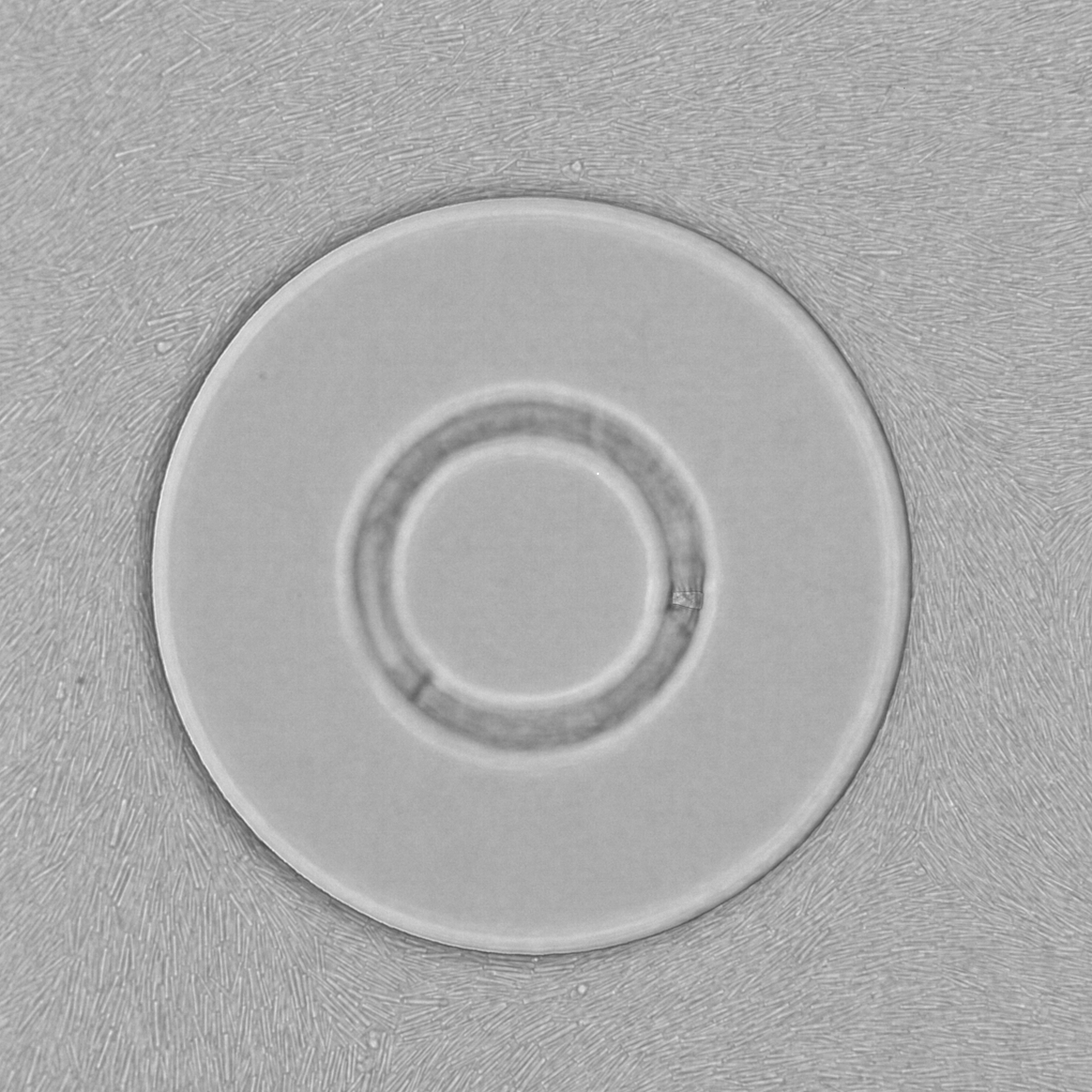

Supplement: Supplementary file 5 — Supplementary Data 2 [file 41467_2020_20842_MOESM5_ESM.zip › rawdata/size4/06_06.tif]

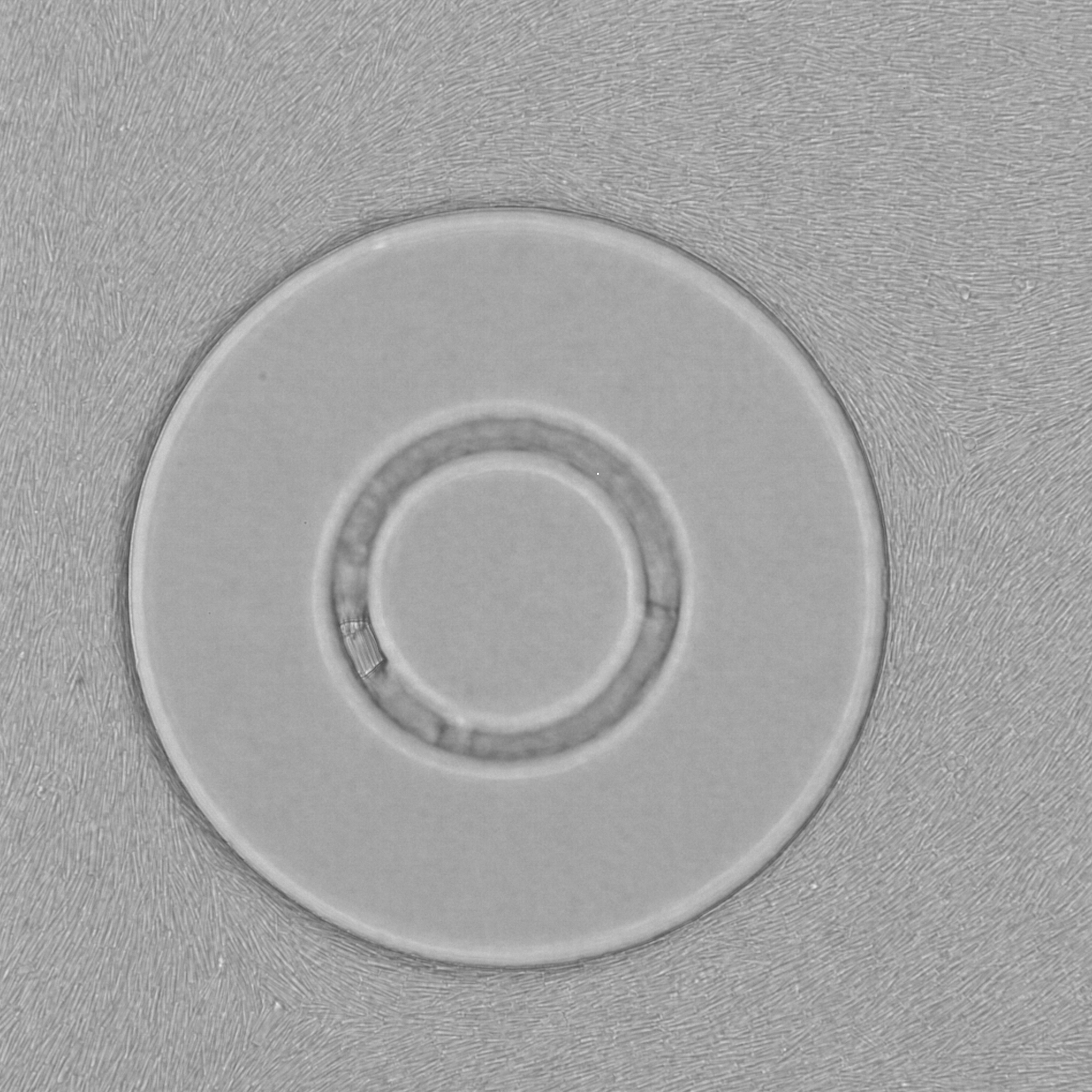

Supplement: Supplementary file 5 — Supplementary Data 2 [file 41467_2020_20842_MOESM5_ESM.zip › rawdata/size4/06_05.tif]

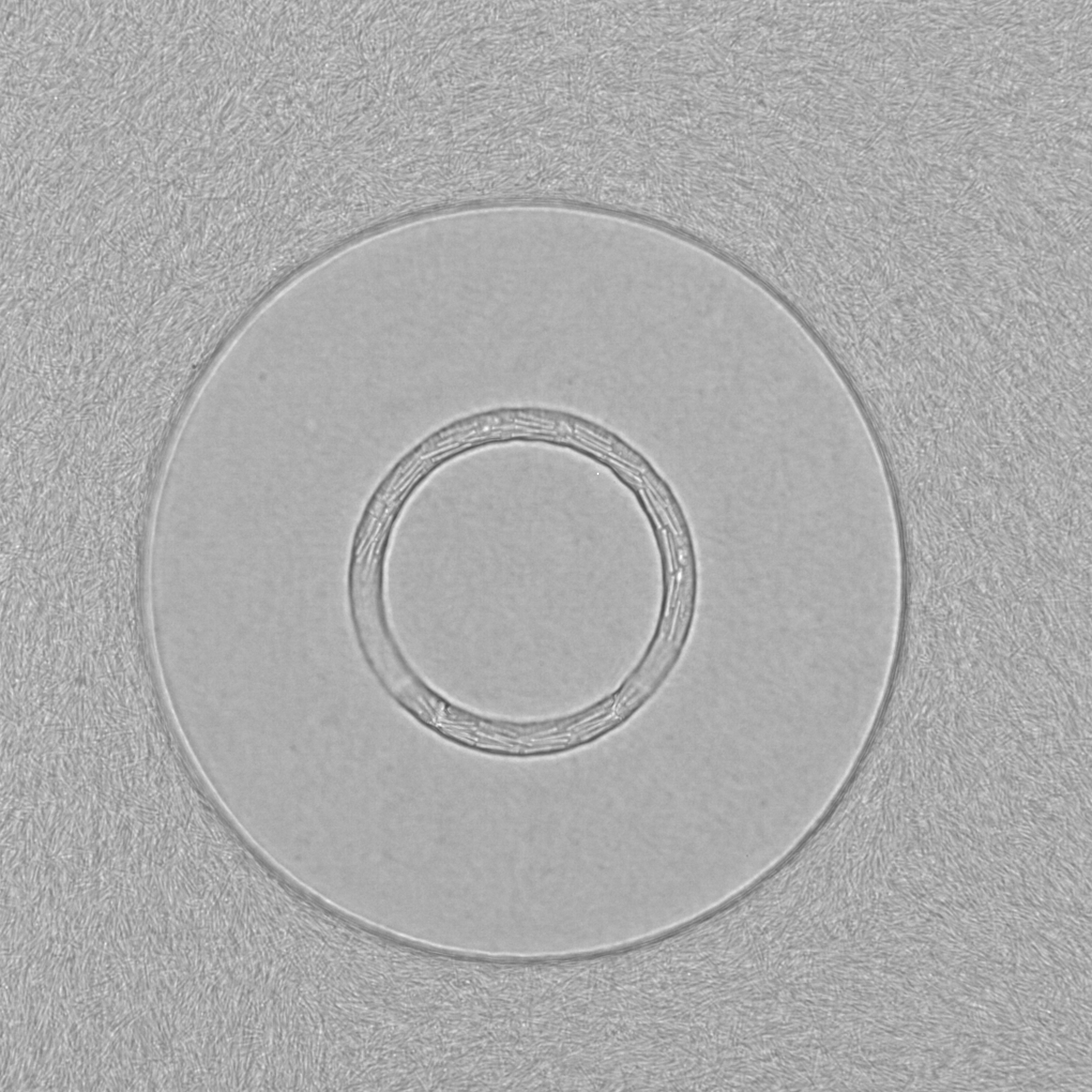

Supplement: Supplementary file 5 — Supplementary Data 2 [file 41467_2020_20842_MOESM5_ESM.zip › rawdata/size4/06_04.tif]

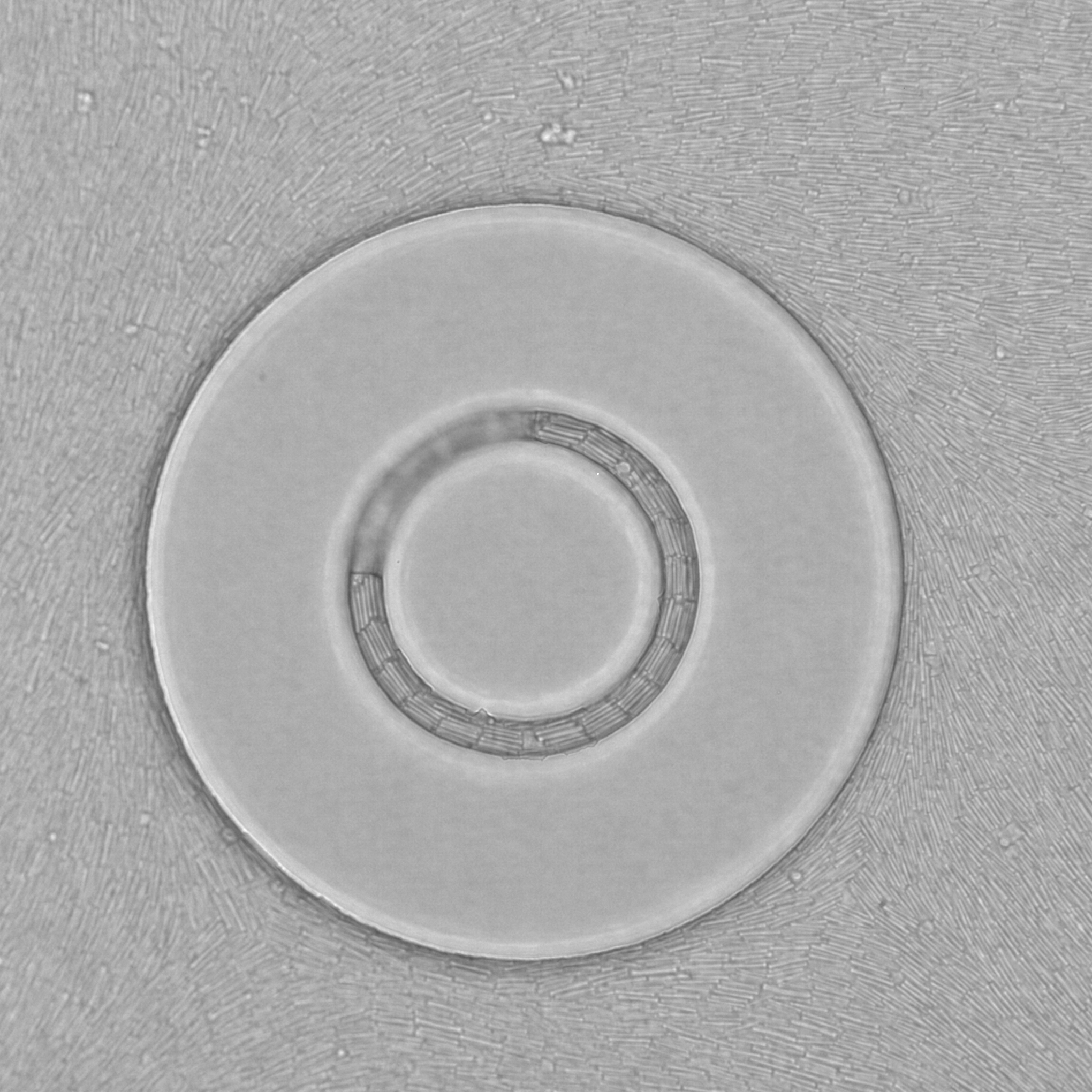

Supplement: Supplementary file 5 — Supplementary Data 2 [file 41467_2020_20842_MOESM5_ESM.zip › rawdata/size4/06_03.tif]

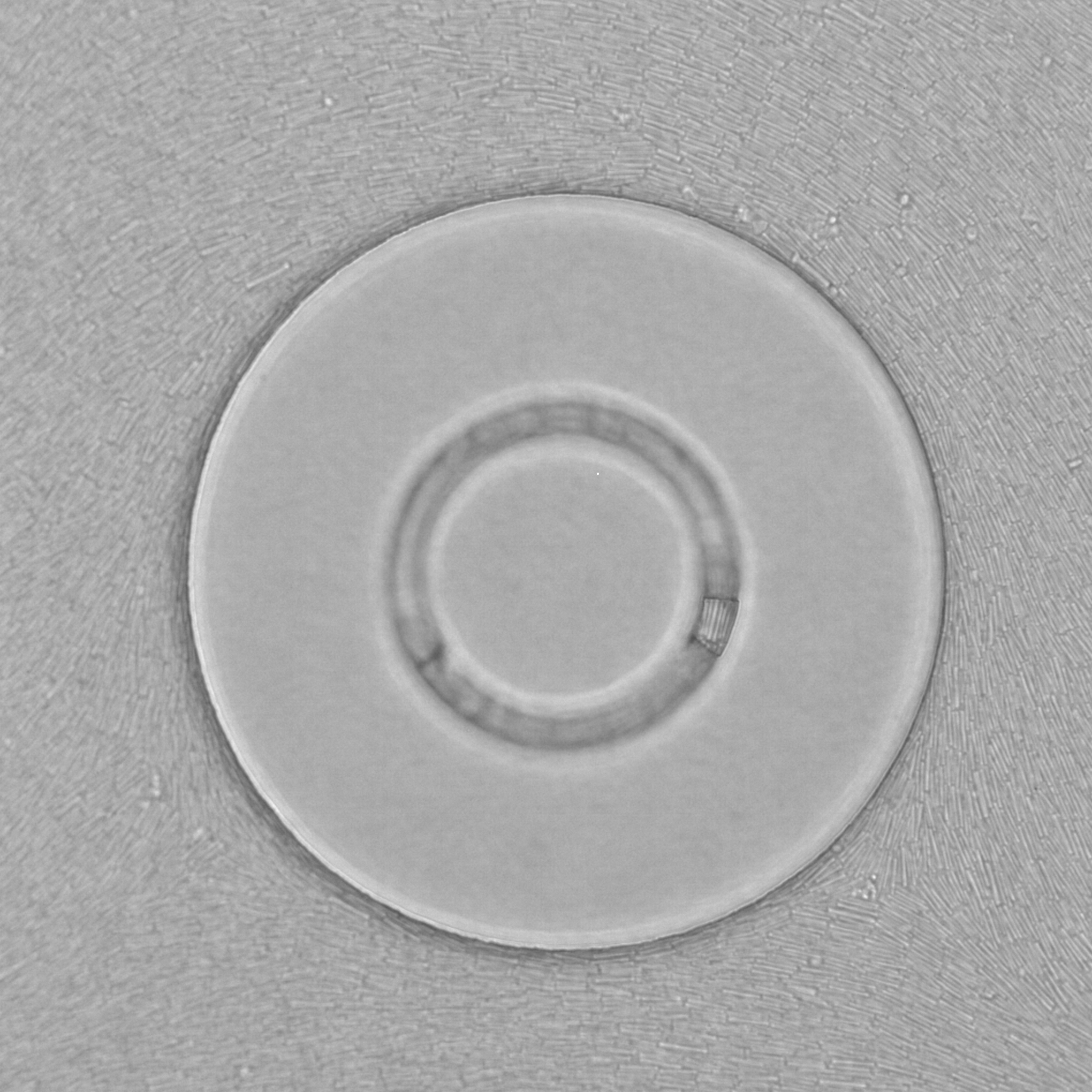

Supplement: Supplementary file 5 — Supplementary Data 2 [file 41467_2020_20842_MOESM5_ESM.zip › rawdata/size4/06_02.tif]

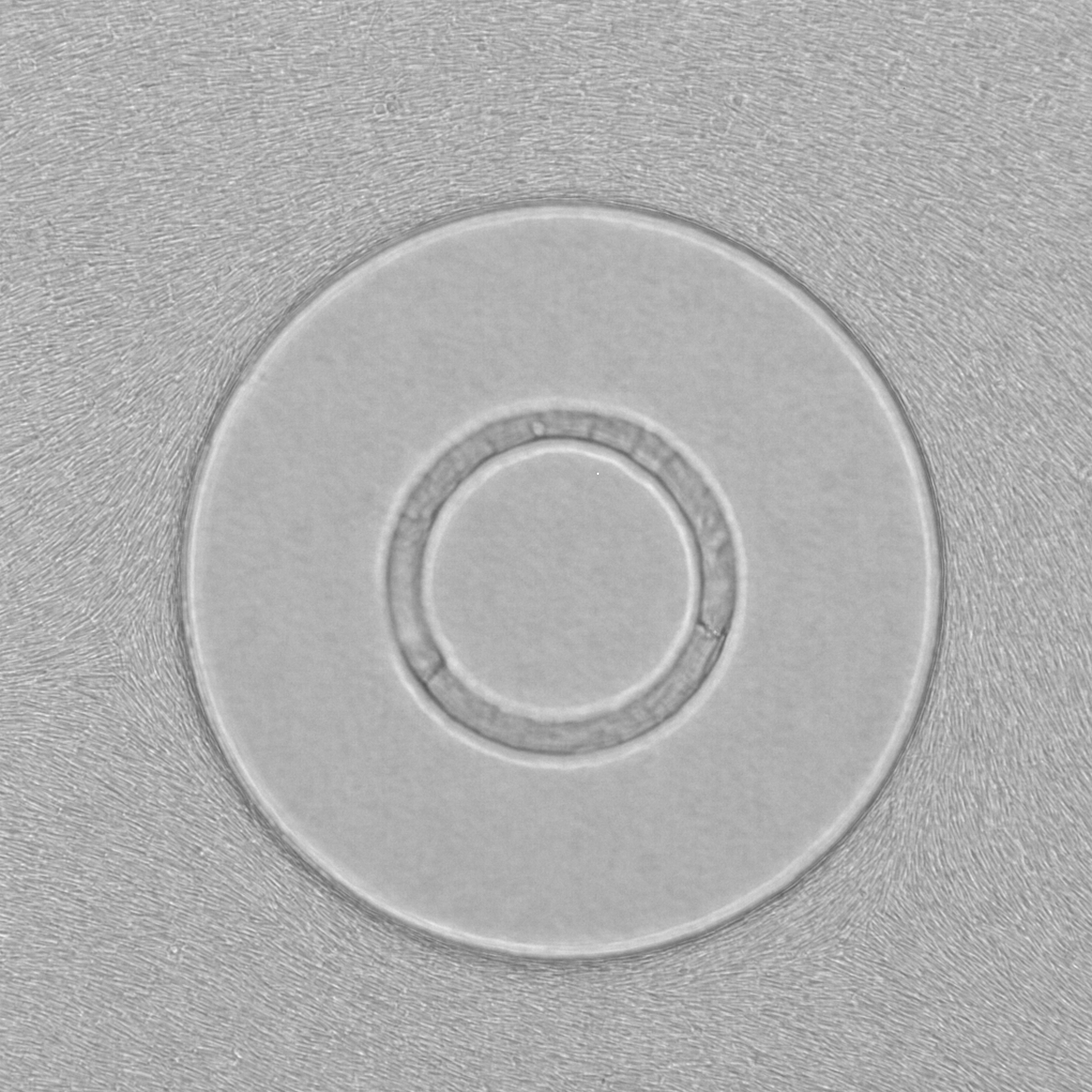

Supplement: Supplementary file 5 — Supplementary Data 2 [file 41467_2020_20842_MOESM5_ESM.zip › rawdata/size4/06_01.tif]

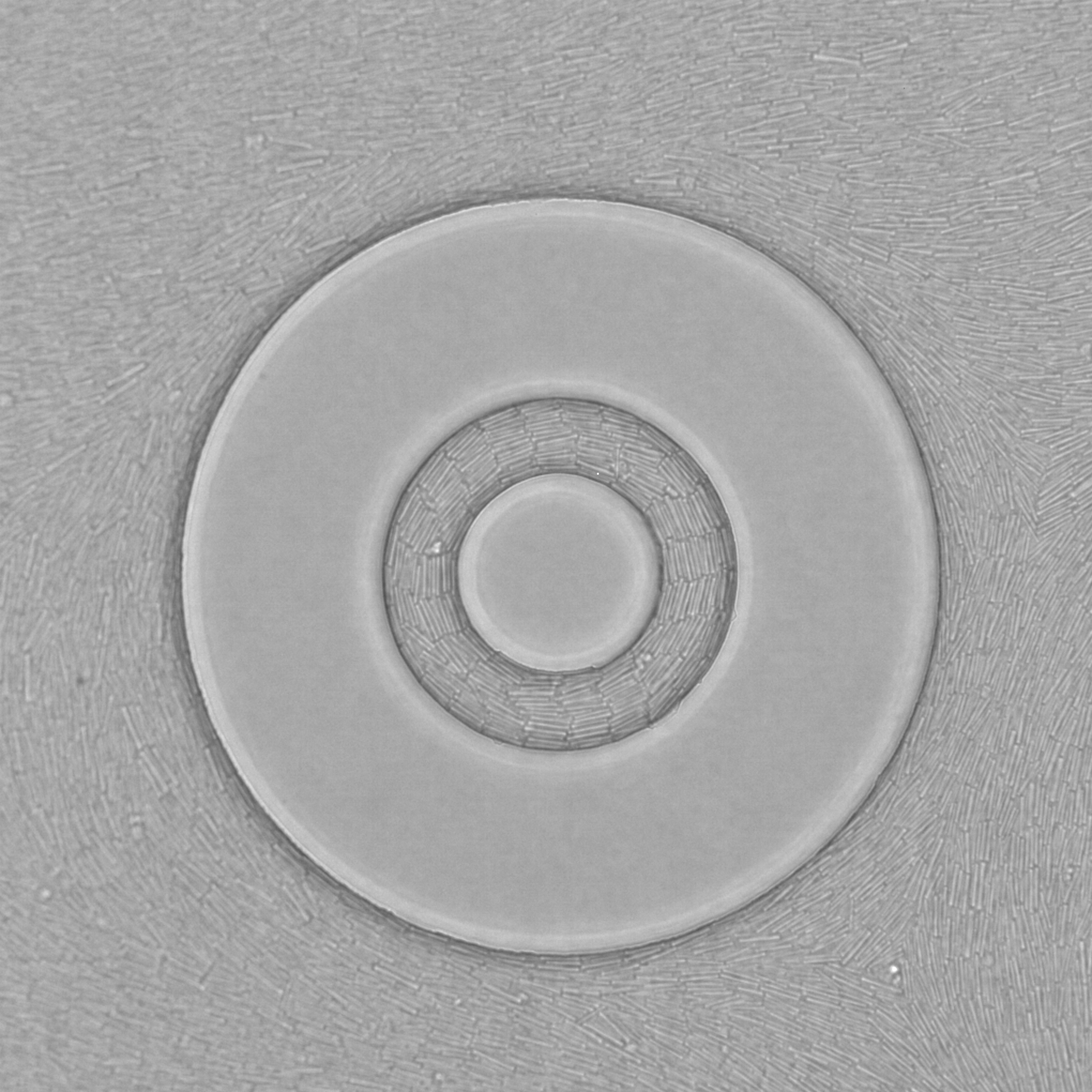

Supplement: Supplementary file 5 — Supplementary Data 2 [file 41467_2020_20842_MOESM5_ESM.zip › rawdata/size4/05_06.tif]

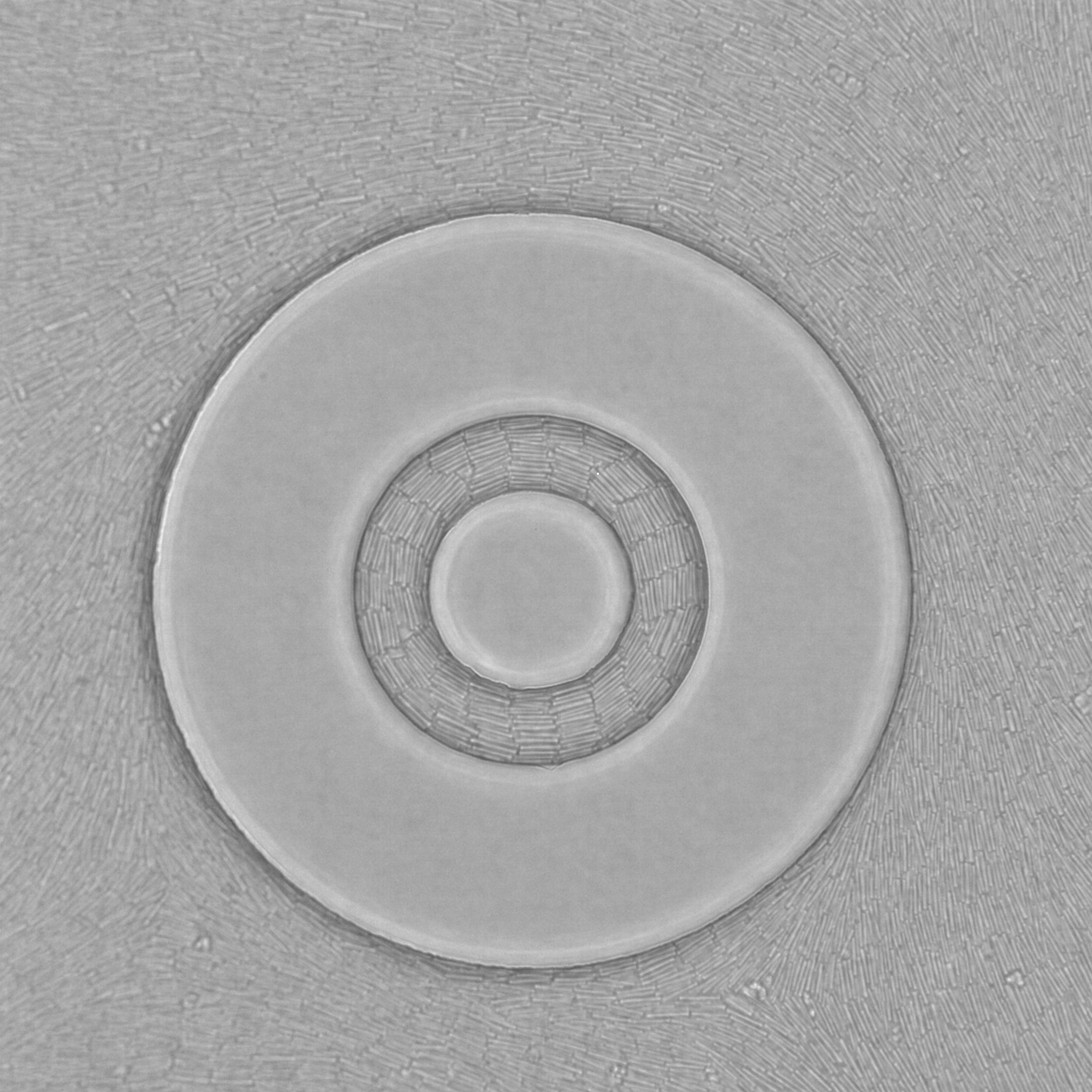

Supplement: Supplementary file 5 — Supplementary Data 2 [file 41467_2020_20842_MOESM5_ESM.zip › rawdata/size4/05_05.tif]

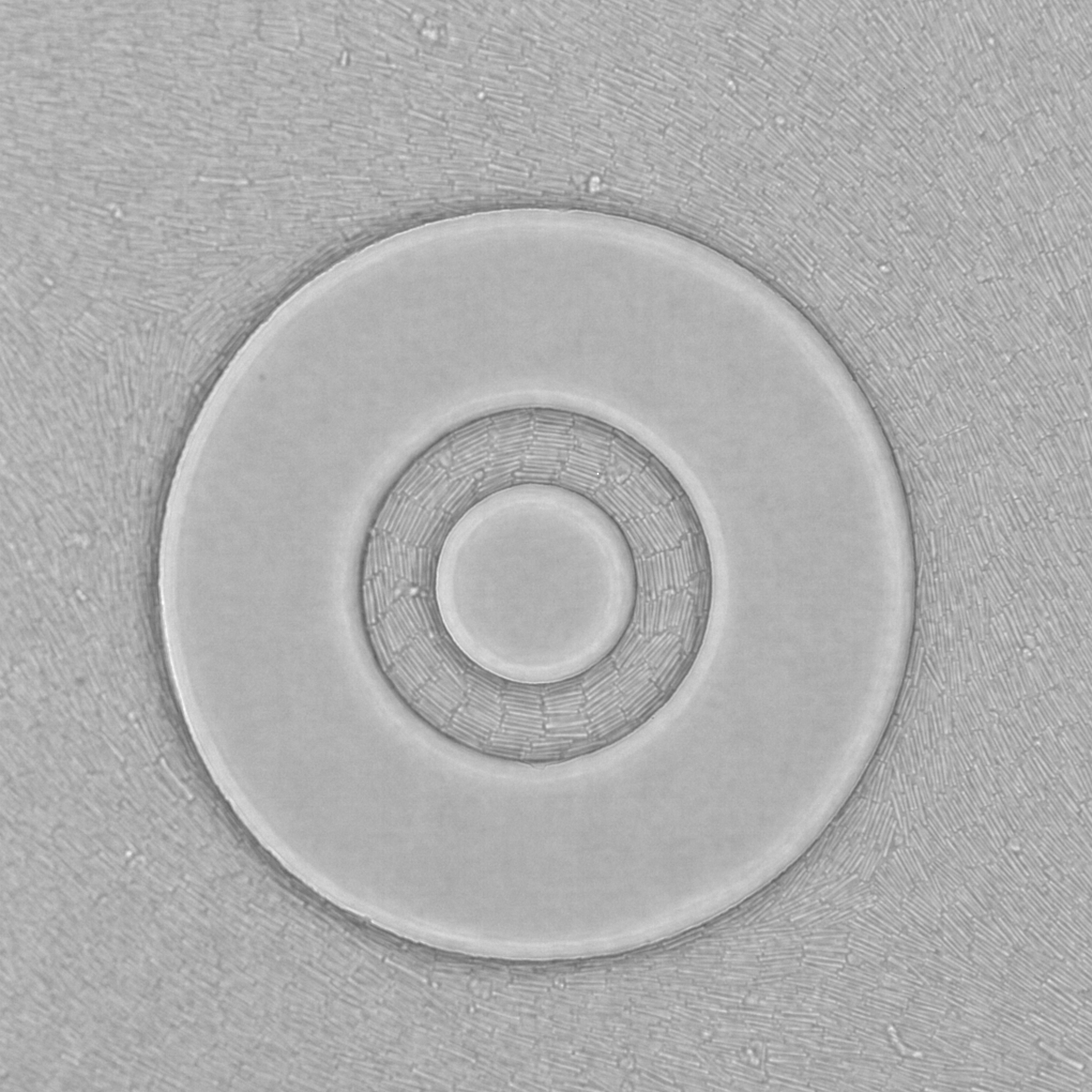

Supplement: Supplementary file 5 — Supplementary Data 2 [file 41467_2020_20842_MOESM5_ESM.zip › rawdata/size4/05_04.tif]

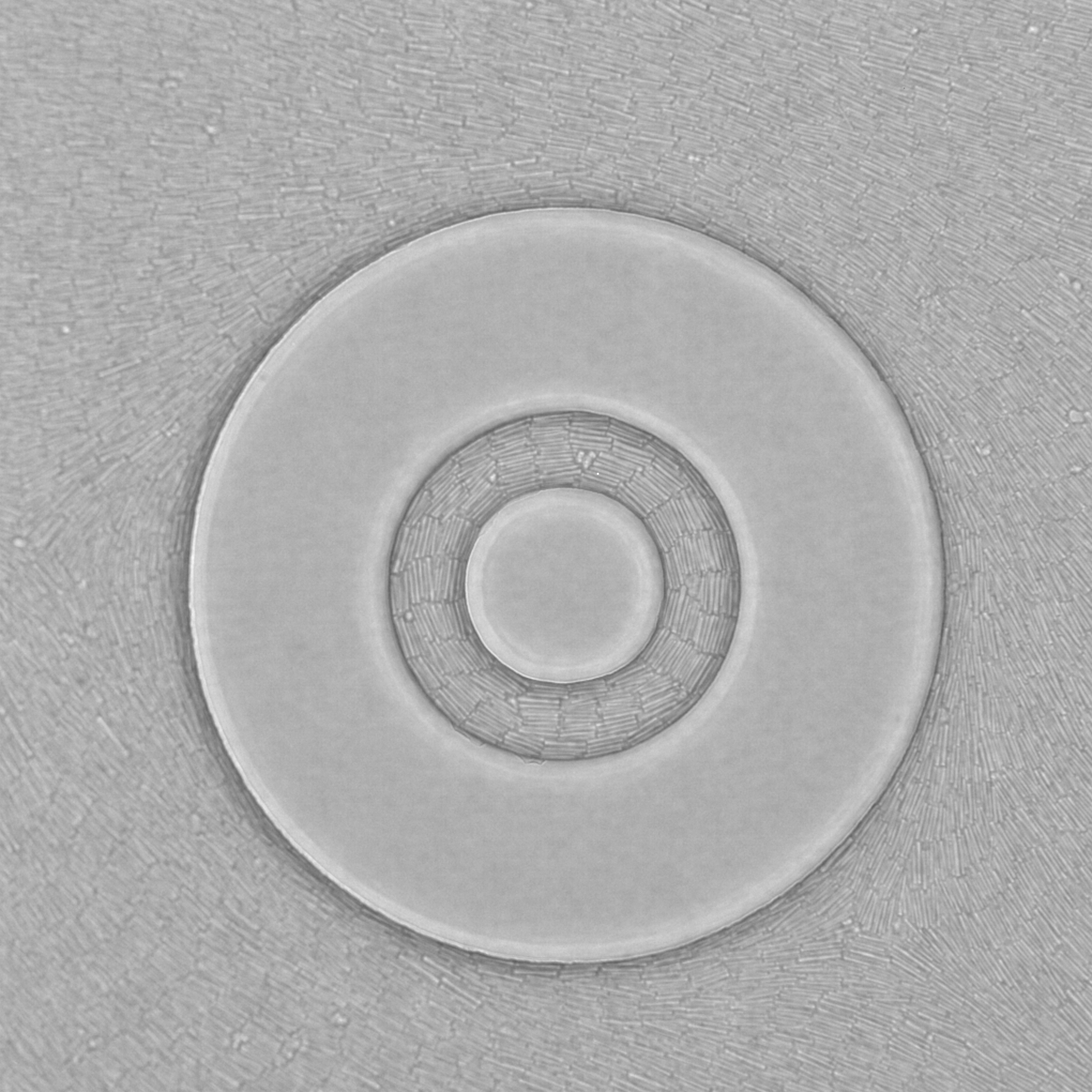

Supplement: Supplementary file 5 — Supplementary Data 2 [file 41467_2020_20842_MOESM5_ESM.zip › rawdata/size4/05_03.tif]

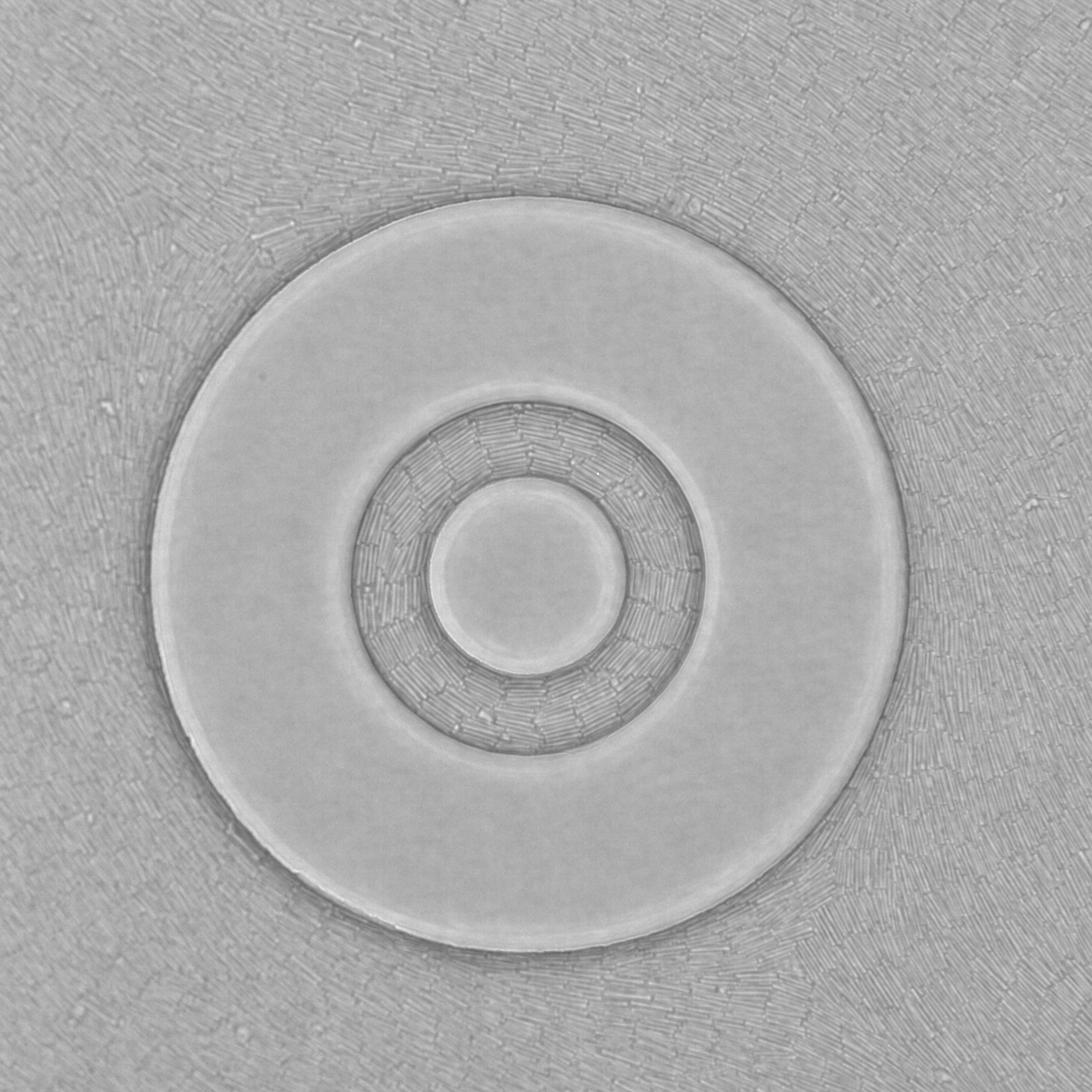

Supplement: Supplementary file 5 — Supplementary Data 2 [file 41467_2020_20842_MOESM5_ESM.zip › rawdata/size4/05_02.tif]

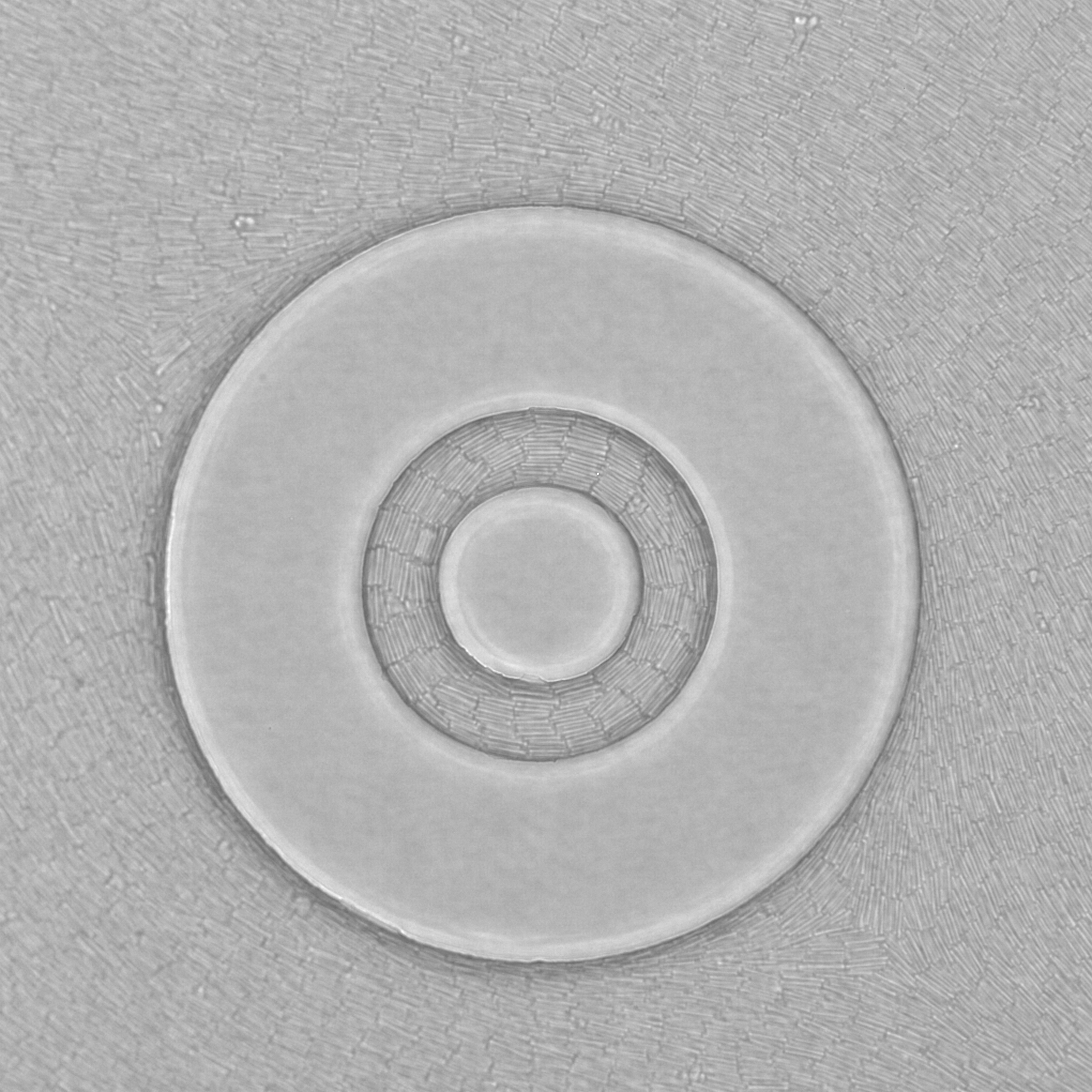

Supplement: Supplementary file 5 — Supplementary Data 2 [file 41467_2020_20842_MOESM5_ESM.zip › rawdata/size4/05_01.tif]

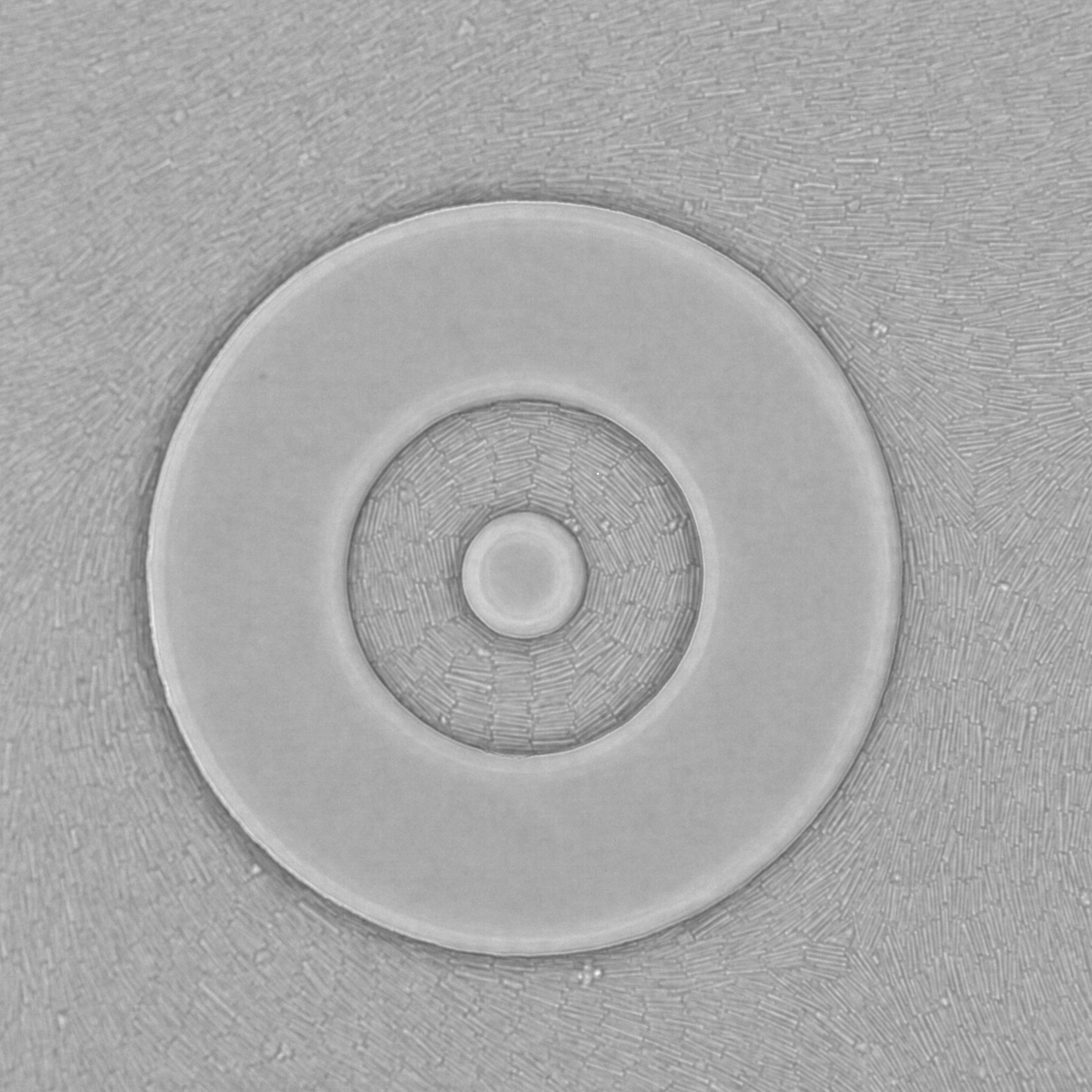

Supplement: Supplementary file 5 — Supplementary Data 2 [file 41467_2020_20842_MOESM5_ESM.zip › rawdata/size4/04_06.tif]

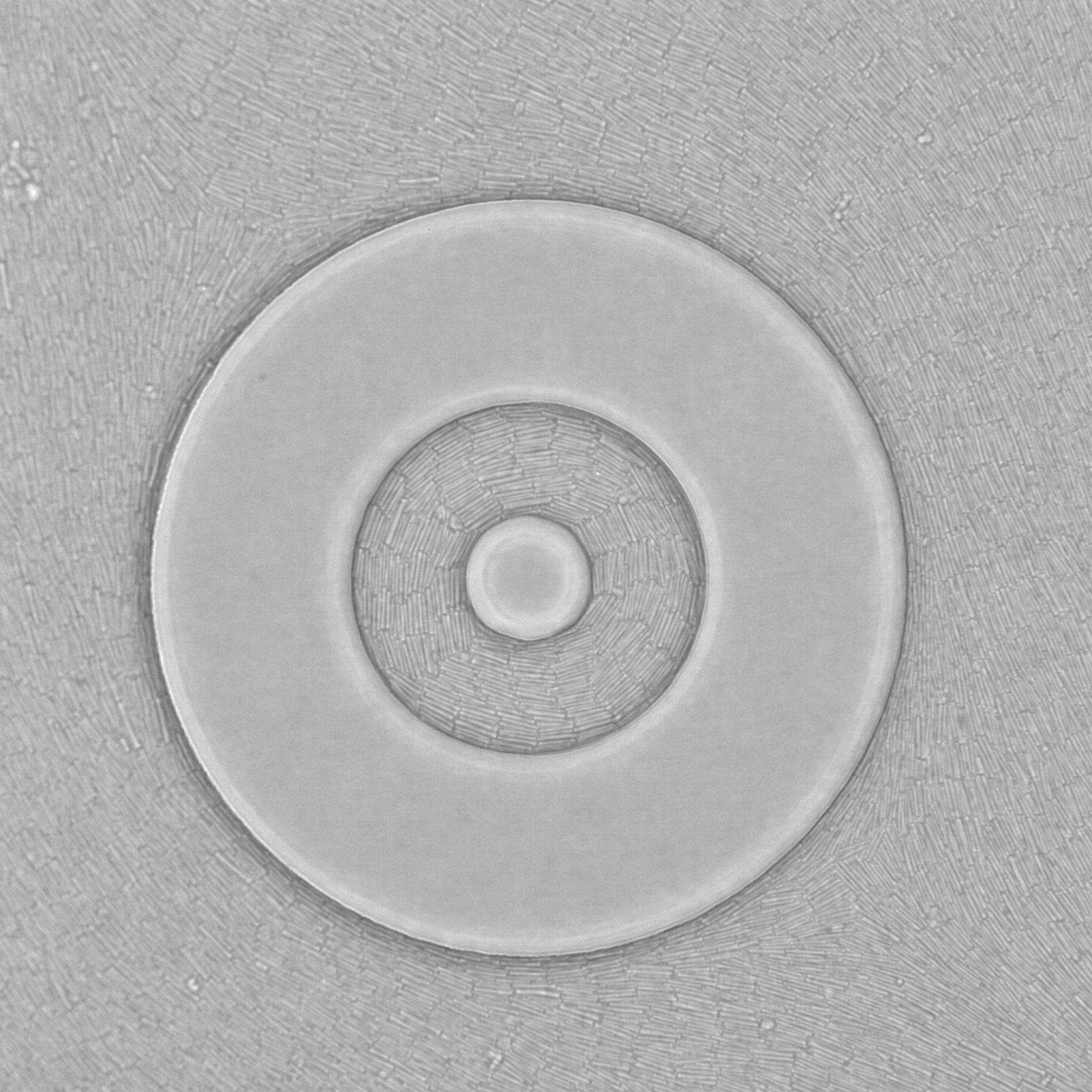

Supplement: Supplementary file 5 — Supplementary Data 2 [file 41467_2020_20842_MOESM5_ESM.zip › rawdata/size4/04_05.tif]

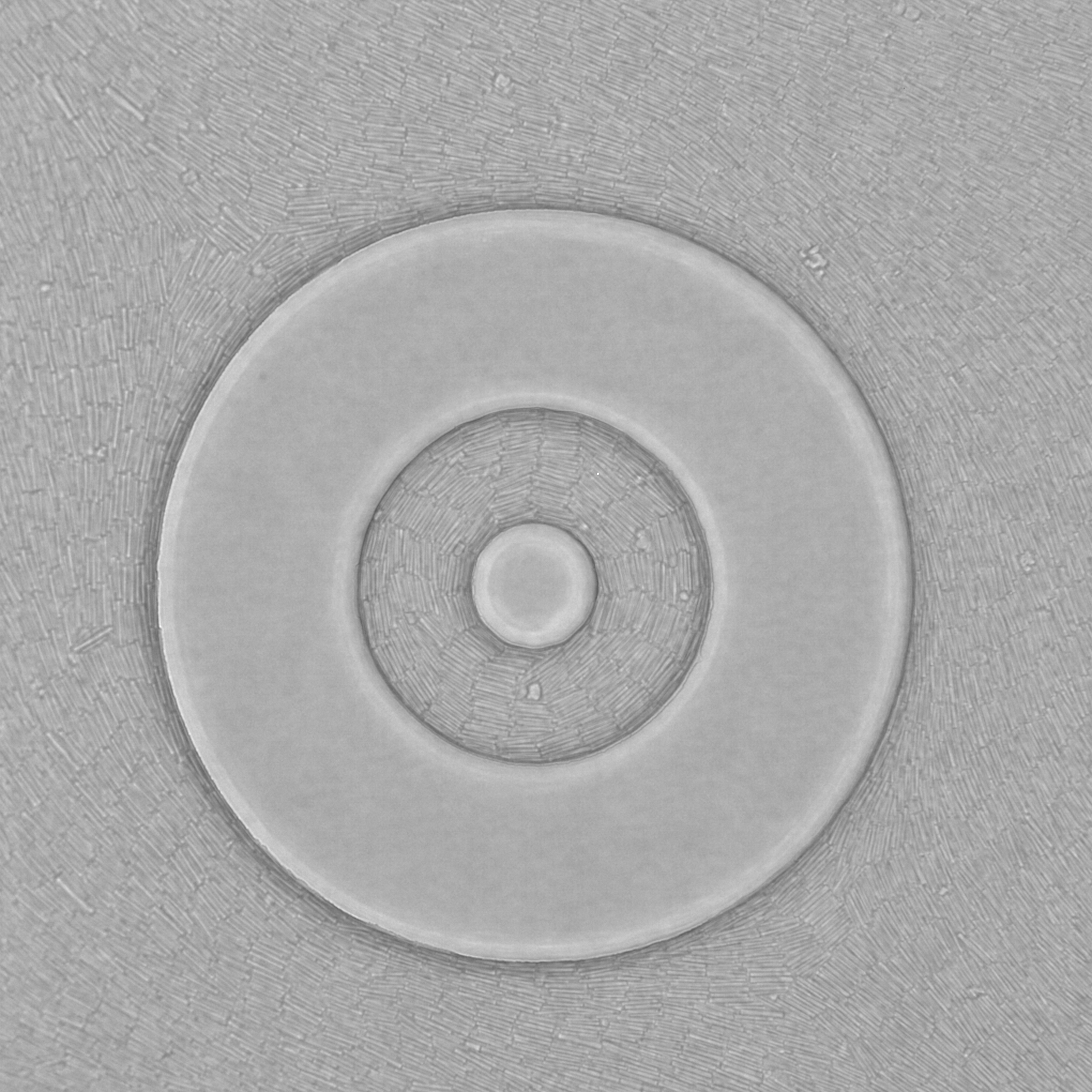

Supplement: Supplementary file 5 — Supplementary Data 2 [file 41467_2020_20842_MOESM5_ESM.zip › rawdata/size4/04_04.tif]

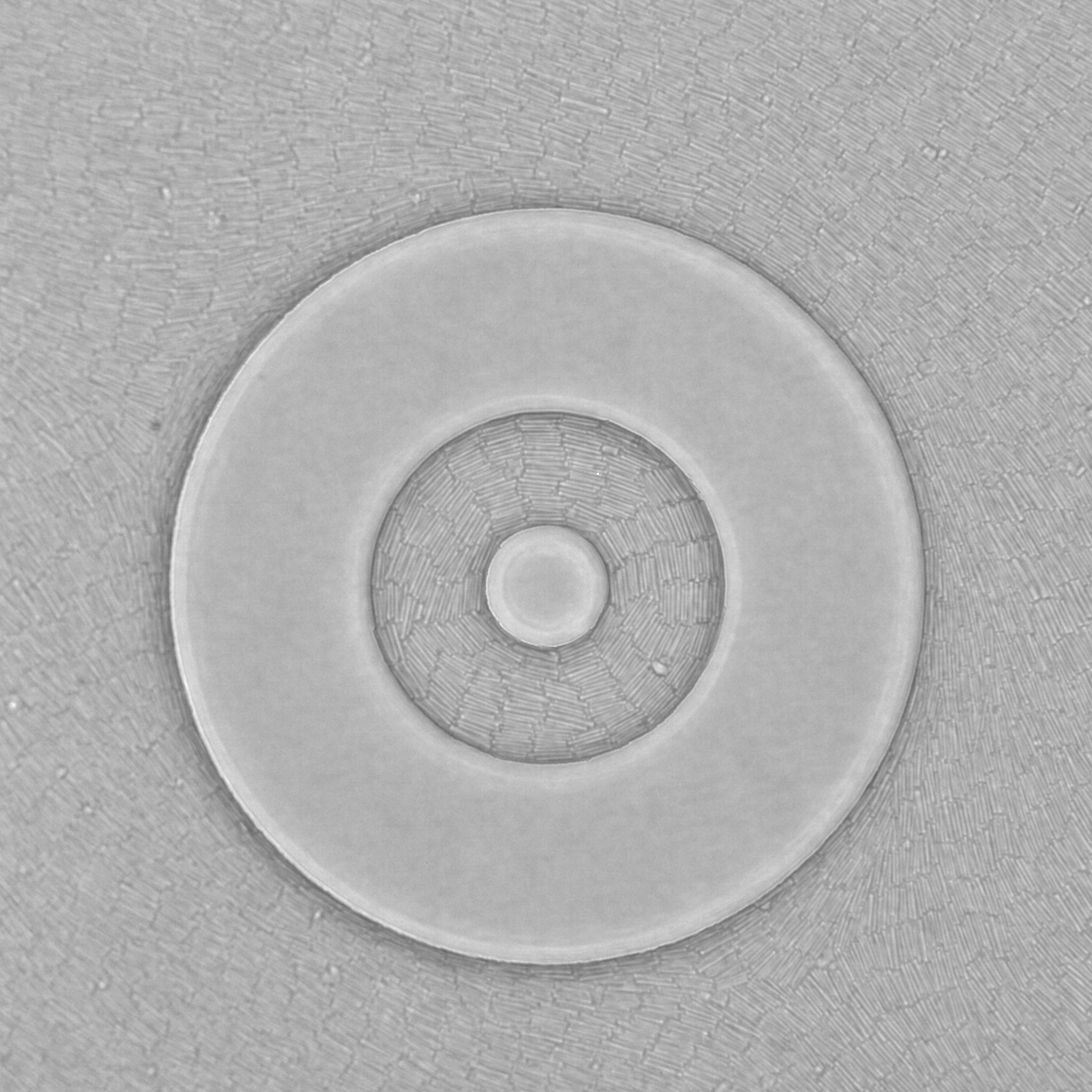

Supplement: Supplementary file 5 — Supplementary Data 2 [file 41467_2020_20842_MOESM5_ESM.zip › rawdata/size4/04_03.tif]

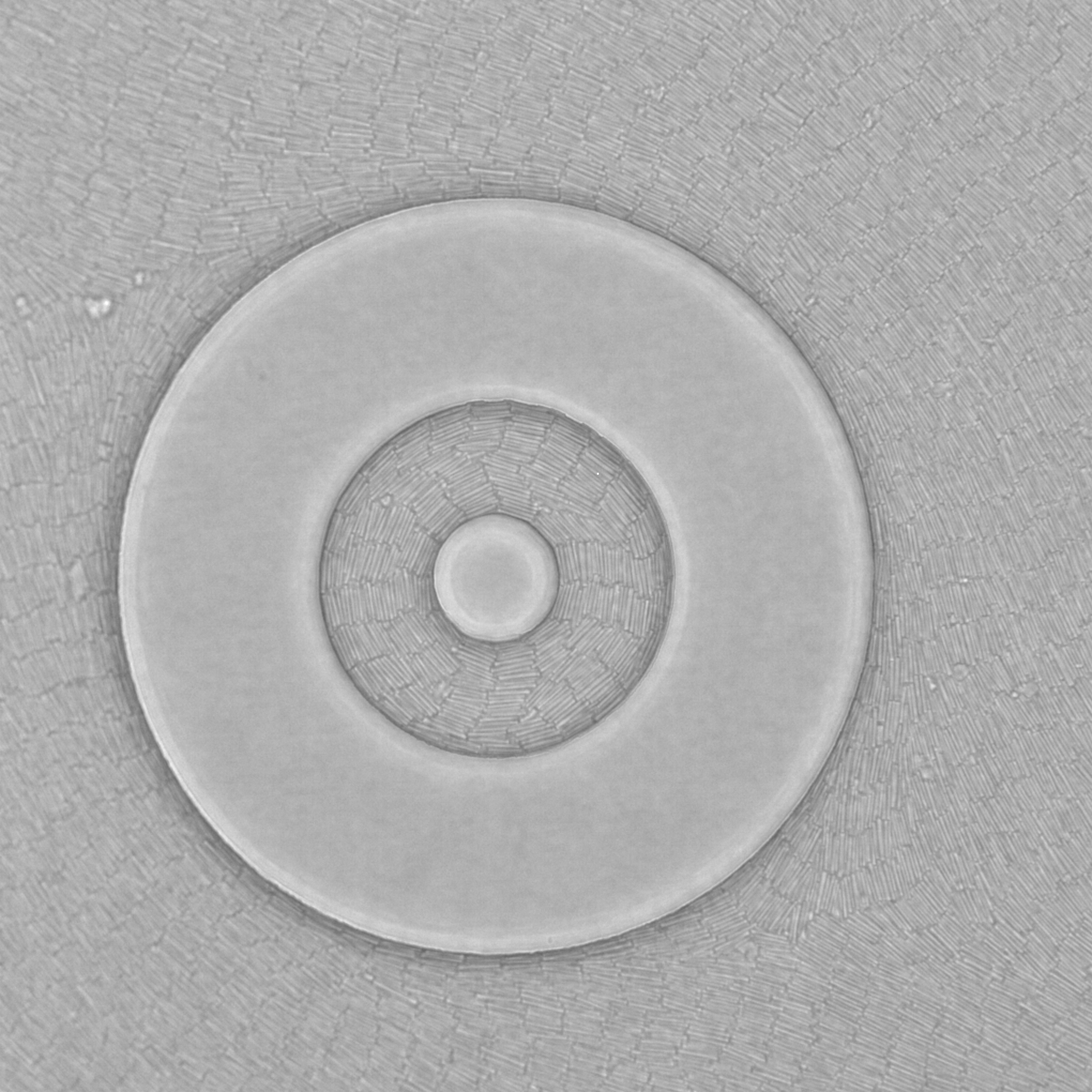

Supplement: Supplementary file 5 — Supplementary Data 2 [file 41467_2020_20842_MOESM5_ESM.zip › rawdata/size4/04_02.tif]

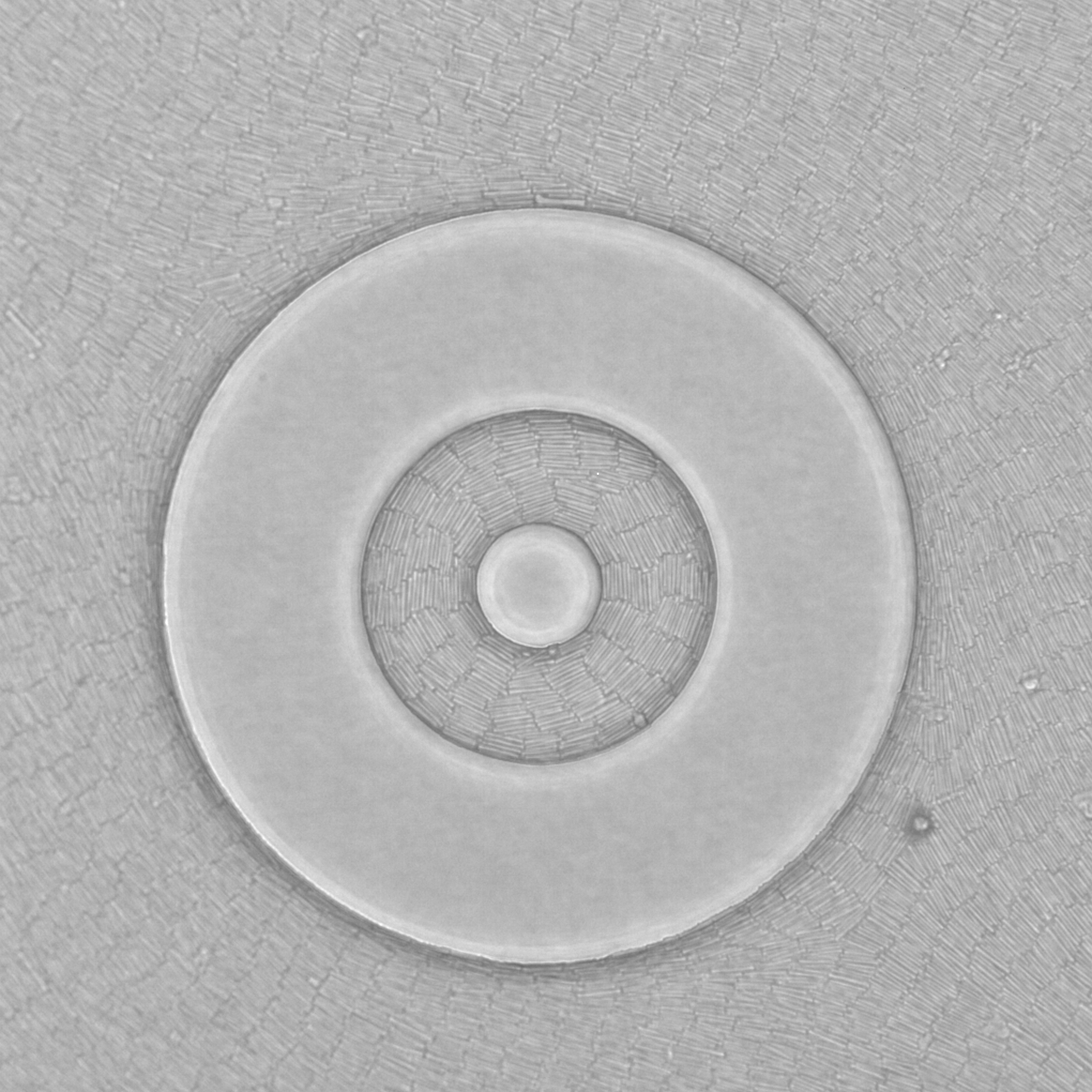

Supplement: Supplementary file 5 — Supplementary Data 2 [file 41467_2020_20842_MOESM5_ESM.zip › rawdata/size4/04_01.tif]

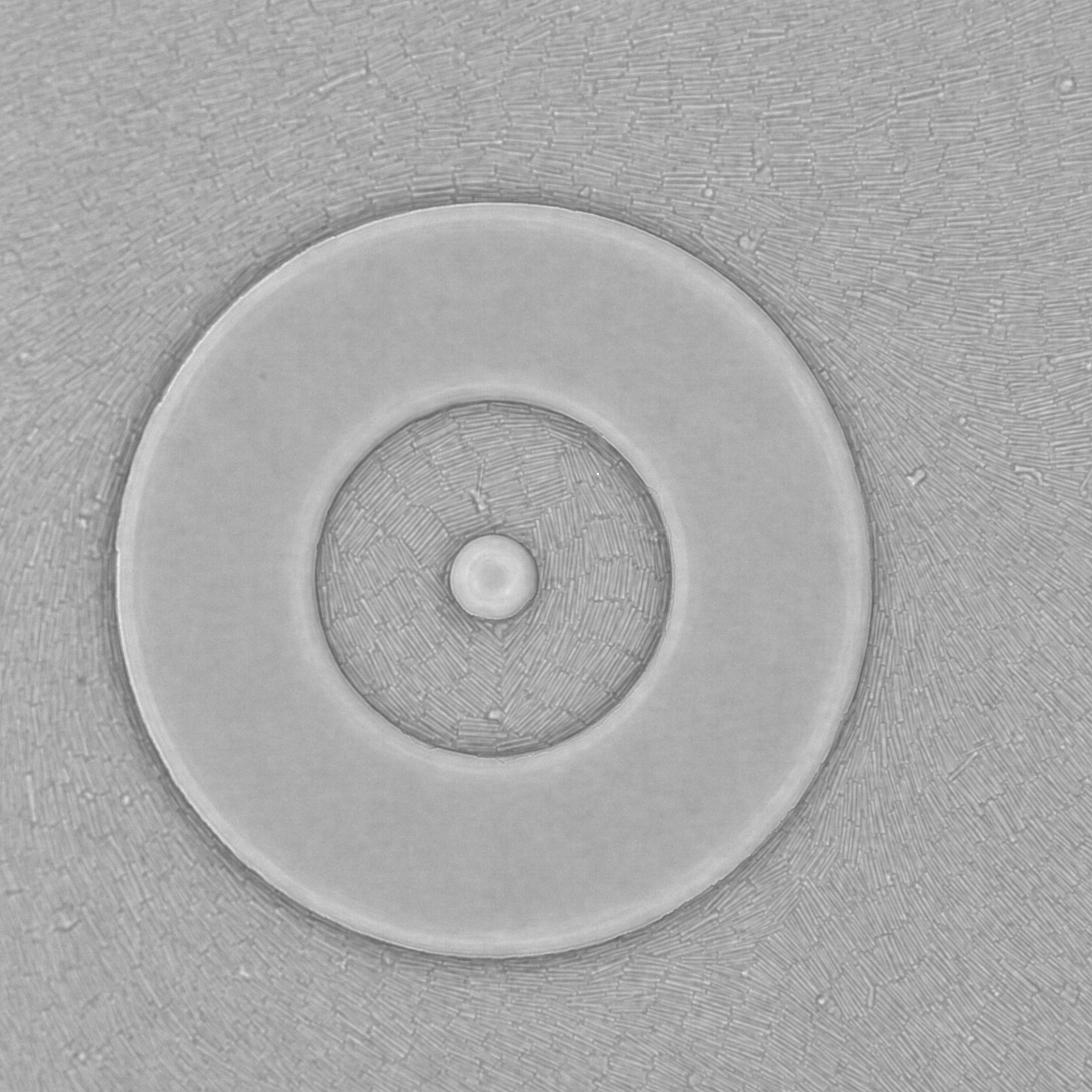

Supplement: Supplementary file 5 — Supplementary Data 2 [file 41467_2020_20842_MOESM5_ESM.zip › rawdata/size4/03_06.tif]

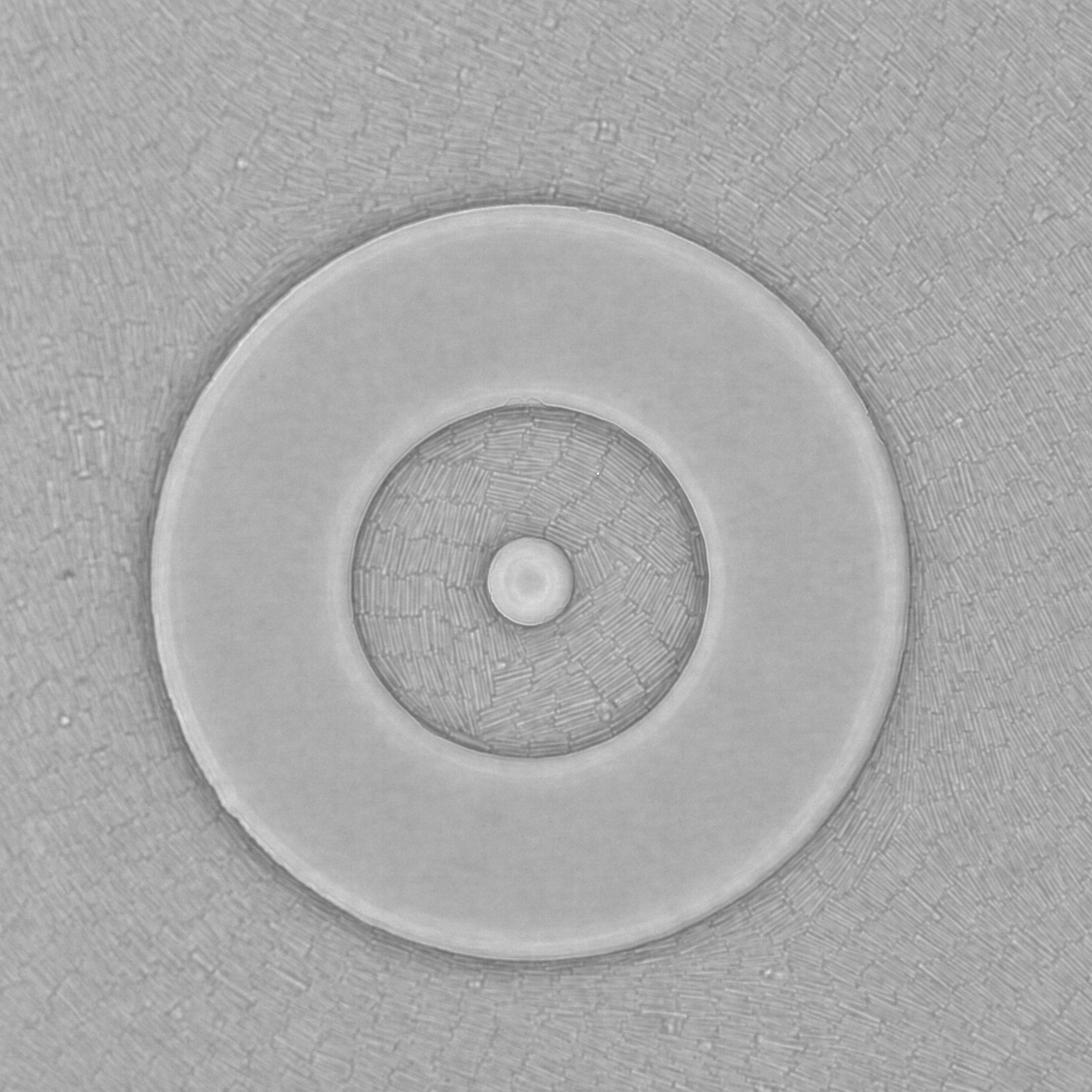

Supplement: Supplementary file 5 — Supplementary Data 2 [file 41467_2020_20842_MOESM5_ESM.zip › rawdata/size4/03_05.tif]
