# Supplementary material for: Particle-resolved topological defects of smectic colloidal liquid crystals in extreme confinement
Source: Nat Commun. 2021 Jan 27;12:623. doi: 10.1038/s41467-020-20842-5 (PMC7840983; doi:10.1038/s41467-020-20842-5)

Rod detection and colouring

Import Image

In[55]:=
```
image = Import[NotebookDirectory[] <> "\\01_01.tif"]
```

Out[55]=

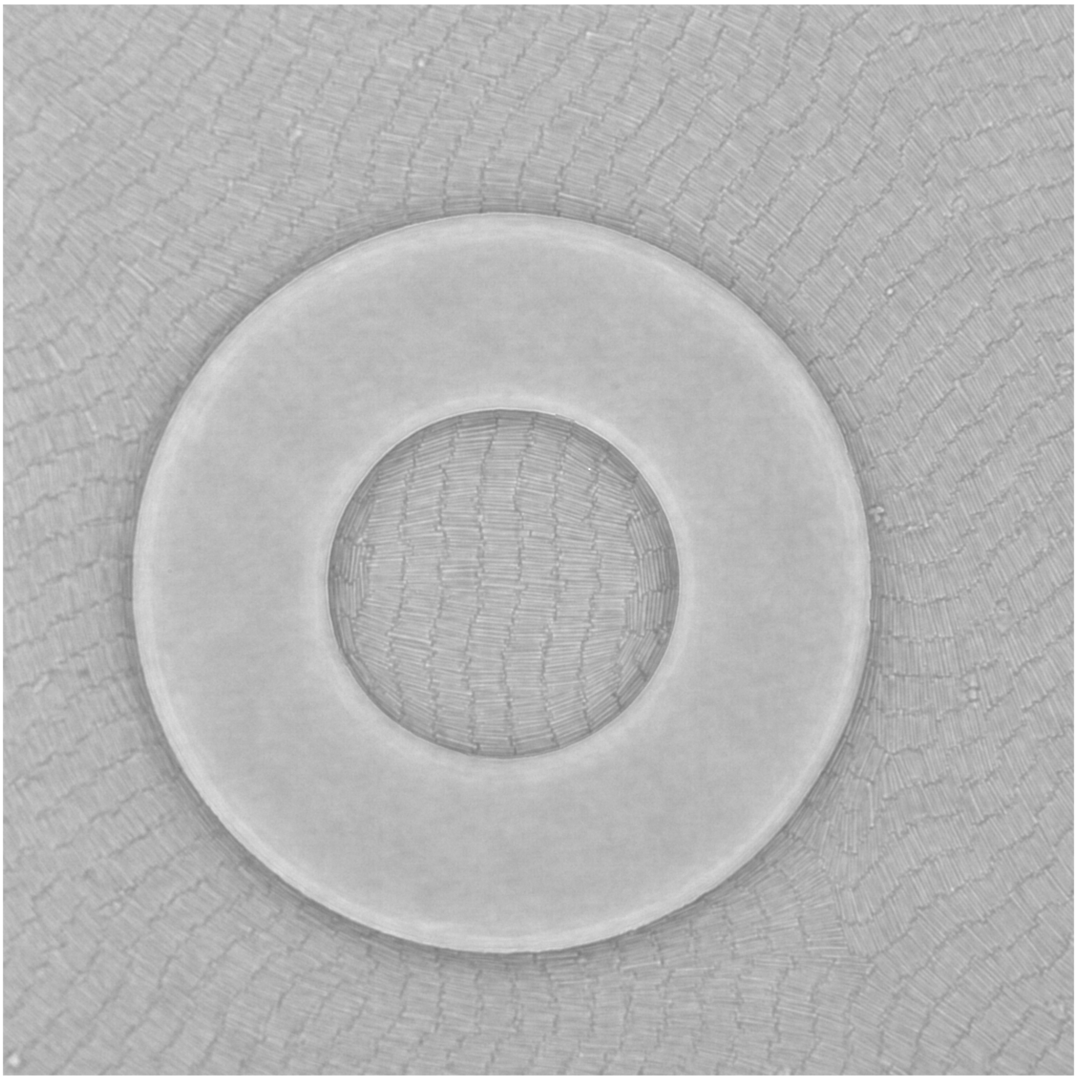

Crop image

In[56]:=
```
imageCrop = ImageTrim[image, {{600, 580}, {600 + 360 * 2, 580 + 360 * 2}}]
```

Out[56]=

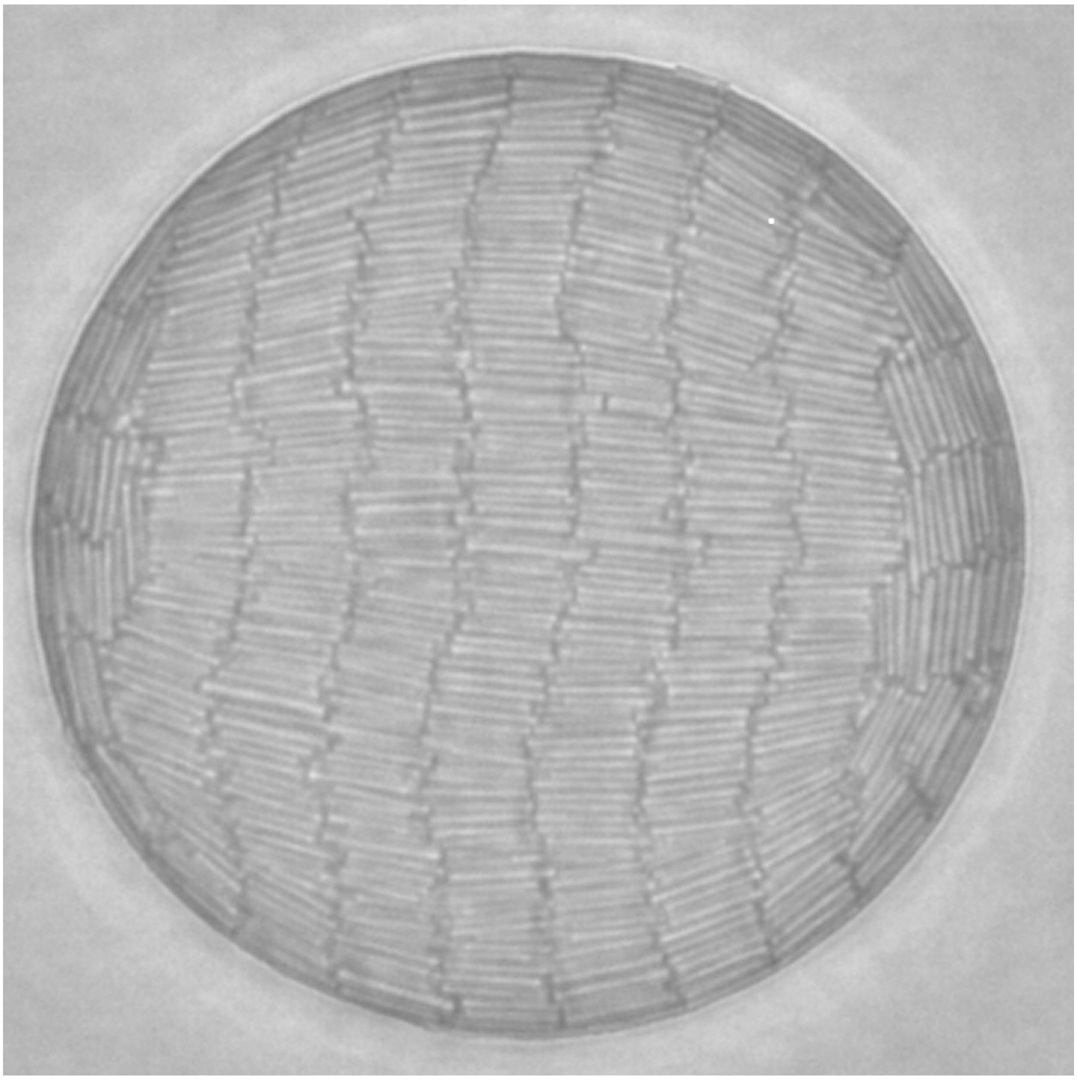

Apply filter

In[57]:=
```
imageFilter = FourierDCTFilter[imageCrop, 0.1]
```

Out[57]=

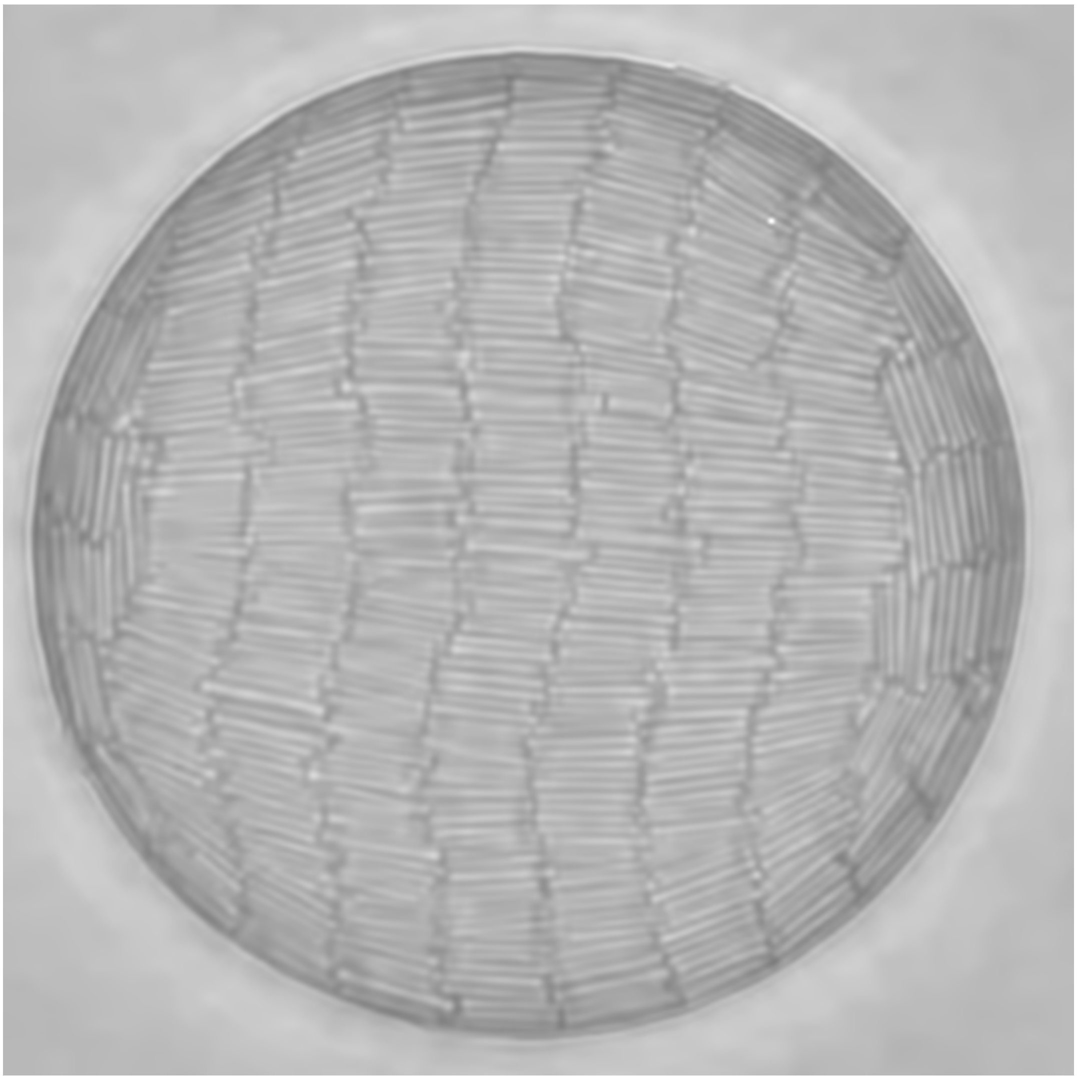

Binarize

In[58]:=
```
localadr = 4;
localadp = {1.02, 0, 0};
```

In[60]:=
```
im1 = LocalAdaptiveBinarize[imageFilter, localadr, localadp]
```

Out[60]=

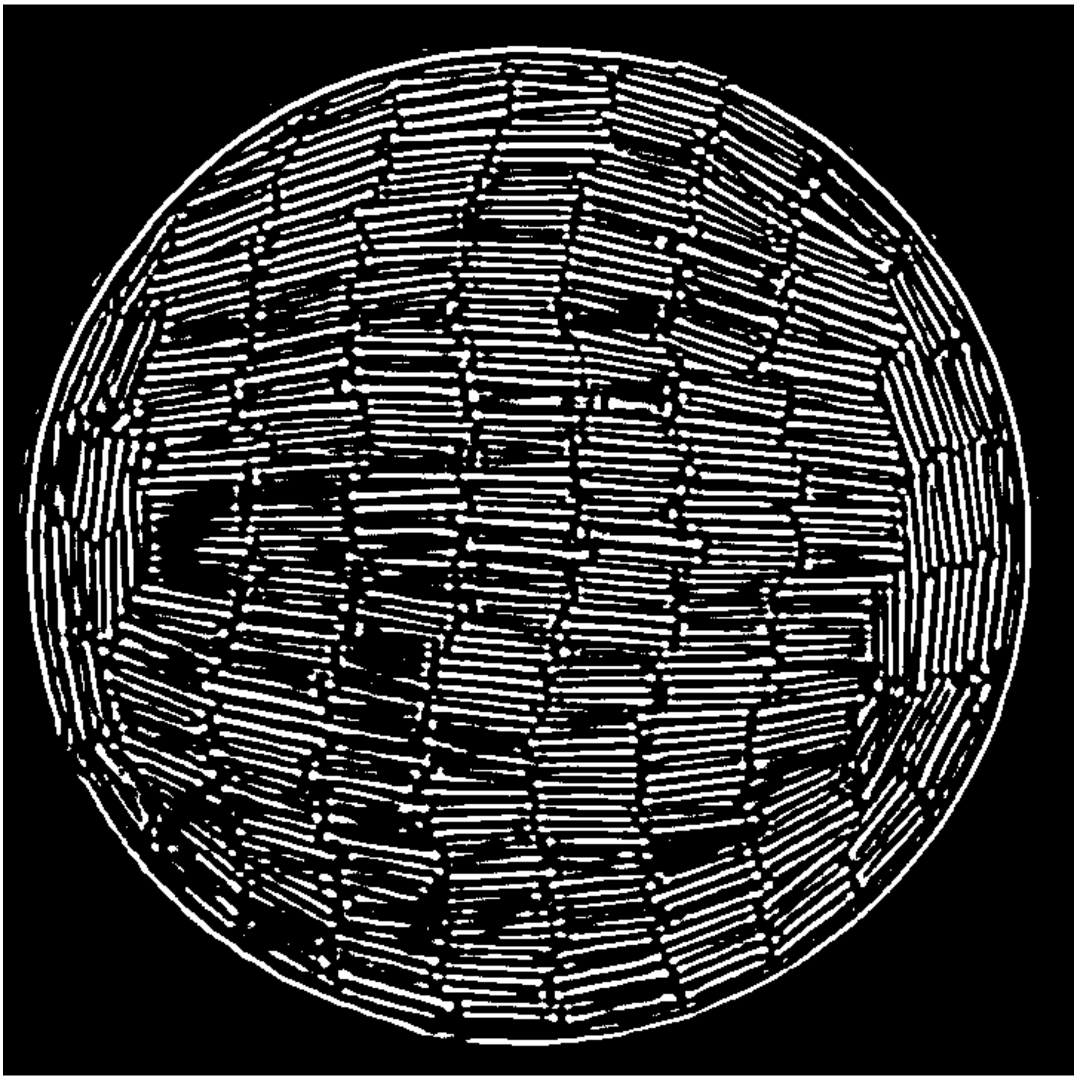

Select component by area and elongation

In[61]:=
```
sel1 = SelectComponents[im1, 30 < #Area < 500 && #Elongation > 0.87 &]
```

Out[61]=

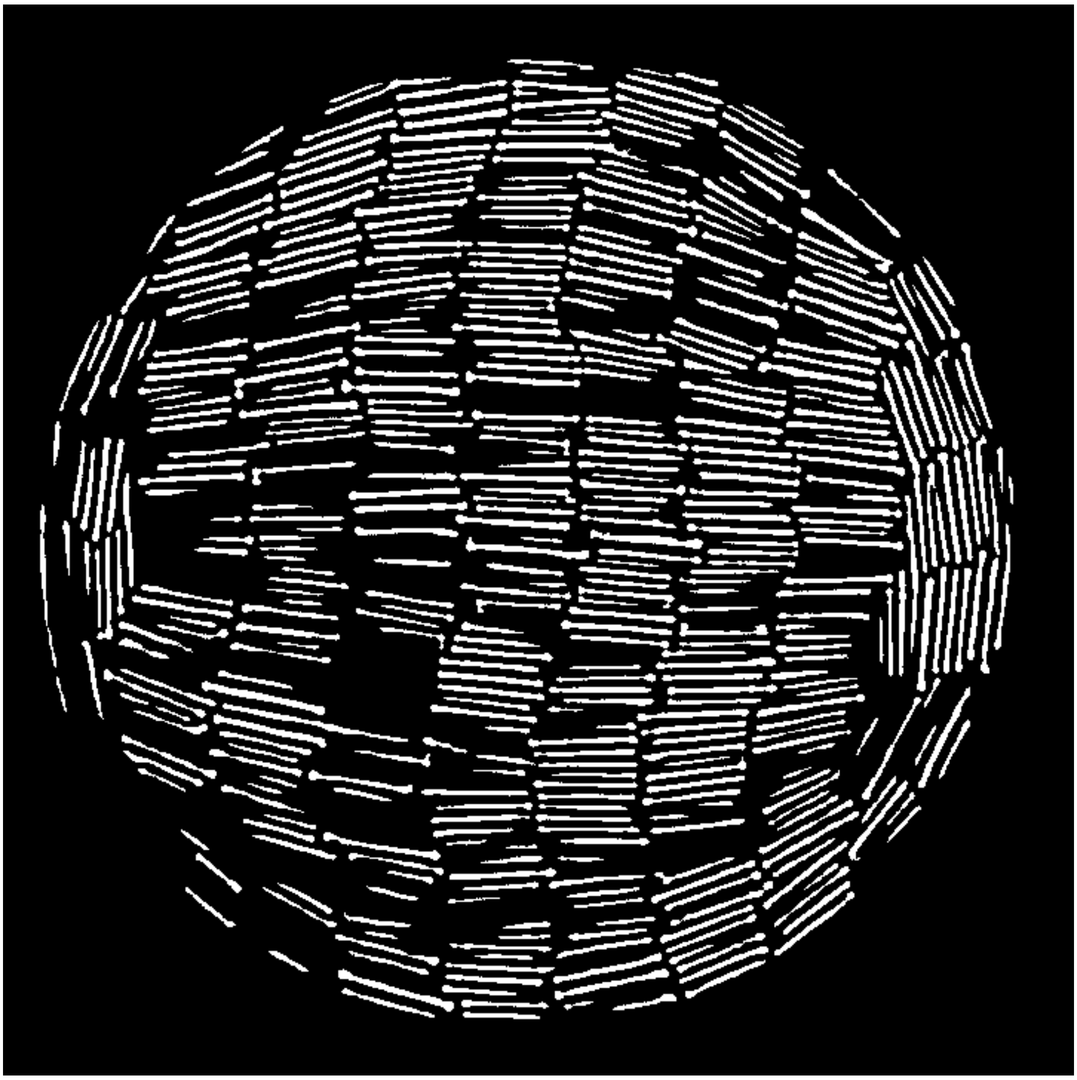

Polar transformation

In[62]:=
```
ImgPolarT[img_] :=
 ({center, radius} = {ImageDimensions[img] / 2, ImageDimensions[img][[1]] / 2};
  polar = ImageTransformation[img, center + {Cos[#[[1]]], Sin[#[[1]]]} * #[[2]] &,
    {2 Pi radius, radius}, DataRange → Full, PlotRange → {{0, 360 °}, {1, radius}}])
```

In[63]:=
```
flat = ImgPolarT[sel1]
```

Out[63]=

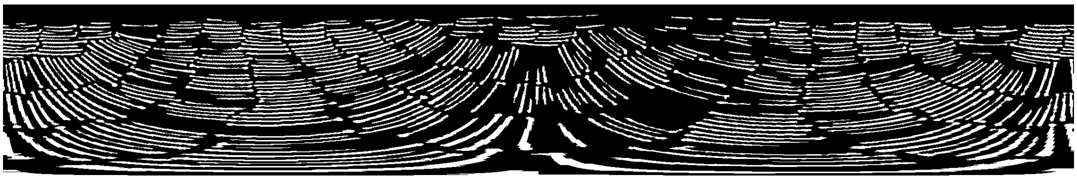

Measure orientation

In[64]:=
```
mes = ComponentMeasurements[flat, "Orientation"]; (* Find layer orientation *)
(* Transform orientation between {-π,π} to {-π/2,π/2} *)
mes[[All, 2]] = If[# < 0, # + π, #] & /@ mes[[All, 2]];
mes[[All, 2]] = If[# > π / 2, # - π, #] & /@ mes[[All, 2]];
mes[[All, 2]] = ((mes[[All, 2]] + π / 2) / π);
(* Transform orientation between {-π/2,π/2} to {0,1} *)
mes[[All, 2]] = If[# == 0, 0.00001, #] & /@ # & /@ mes[[All, 2]];
(* Replace 0 values by 0.0001, 0 will be use for the background *)
PrependTo[mes, 0 → 0]; (* Used to make the background black *)
Dlaymod =
  Sequence @@ # & /@ # & /@ ((Nearest[mes, #]) & /@ MorphologicalComponents[flat]);
 (* Replace the morphological component values by the
 measured orientation at the location *)
col = Colorize[Image[Dlaymod],
  ColorFunction -> Function[{x}, Hue[x]], ColorRules → {0 → Black}]
```

Out[71]=
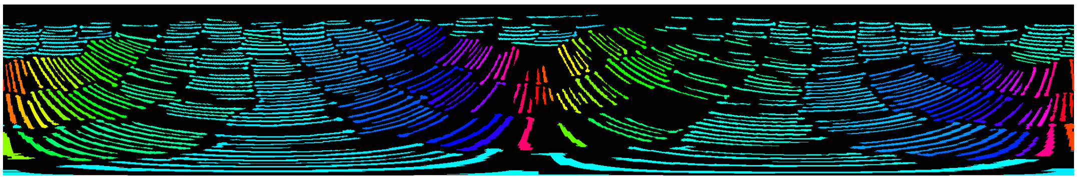

Transform back

In[72]:=
```
reversetransformation[img_] := ImageTransformation[img,
  {ArcTan @@ (radius - #), Norm[# - radius]} &, {2 radius, 2 radius},
  DataRange → {{-180 °, 180 °}, {1, radius}}, PlotRange → {{0, 2 radius}, {0, 2 radius}}]
```

In[73]:=
```
col1 = reversetransformation[col]
```

Out[73]=

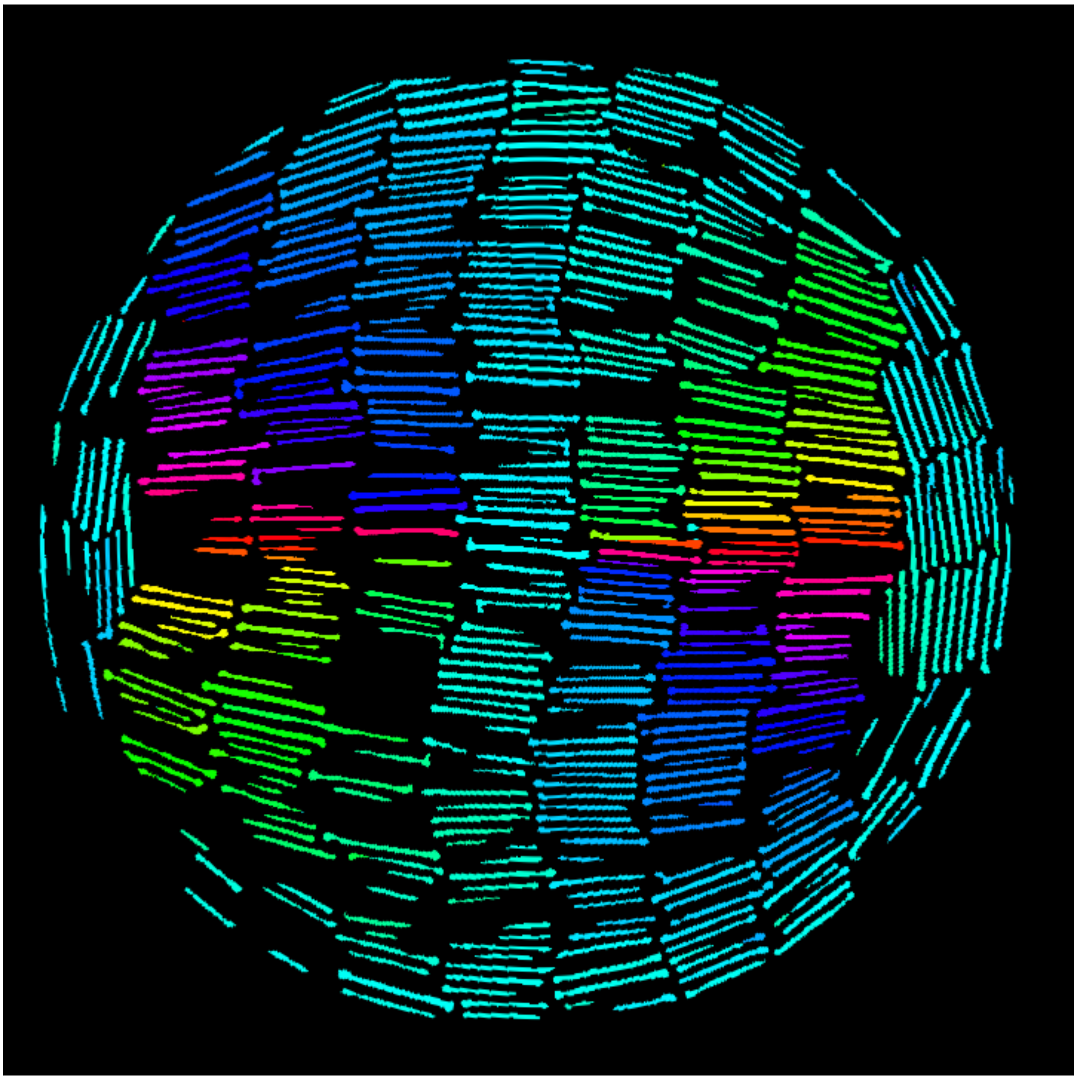

Assemble with original image

In[74]:=
```
(* Used to homogenize brightness between images*)
refhistogram = ImageAdjust[imageCrop];
```

In[75]:=
```
imdim = ImageDimensions[imageCrop];
col1 = ImageTake[col1, {1, imdim[[2]]}, {1, imdim[[1]]}];
3/4 * col1 +
 3/4 ColorNegate[Binarize[col1, 0.1]] * HistogramTransform[imageCrop, refhistogram]
```

Out[77]=

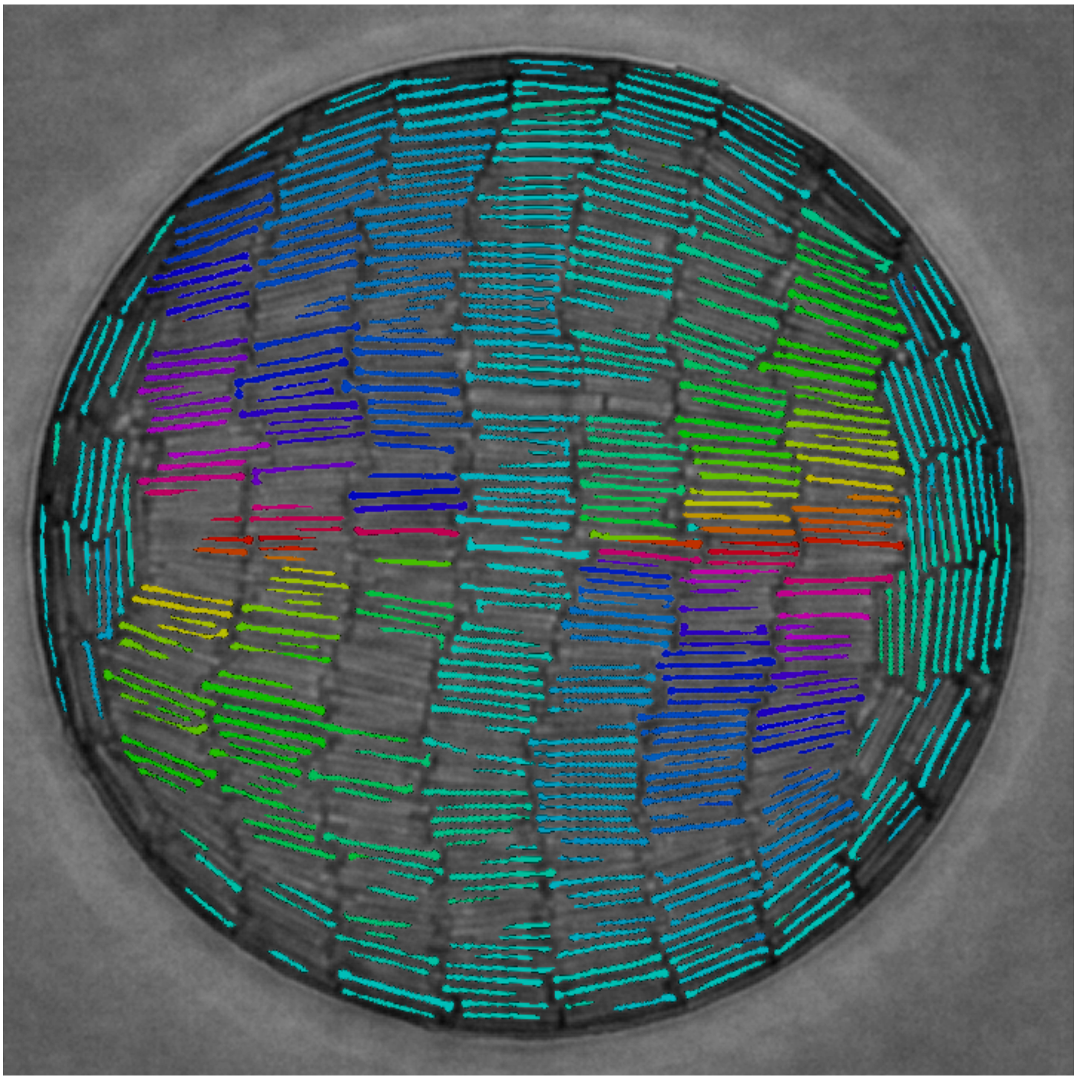

Supplement: Supplementary file 5 — Supplementary Data 2 [file 41467_2020_20842_MOESM5_ESM.zip › rawdata/ImageAnalysis/SingleImageAnalysis_CodeAndResults.pdf]
