# Supplementary figures and images for: Particle-resolved topological defects of smectic colloidal liquid crystals in extreme confinement (part 2 of 2)
Source: Nat Commun. 2021 Jan 27;12:623. doi: 10.1038/s41467-020-20842-5 (PMC7840983; doi:10.1038/s41467-020-20842-5)

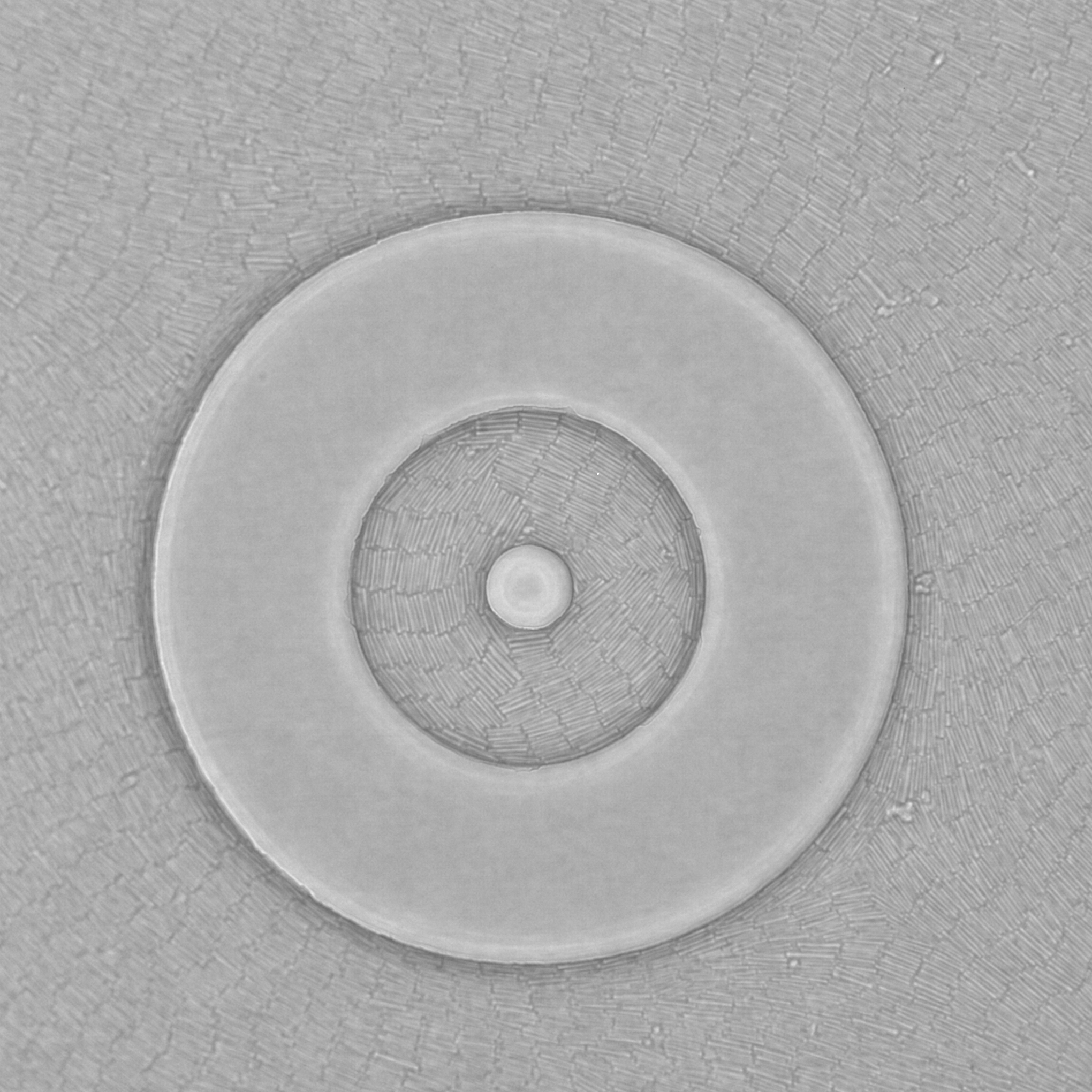

Supplement: Supplementary file 5 — Supplementary Data 2 [file 41467_2020_20842_MOESM5_ESM.zip › rawdata/size4/03_04.tif]

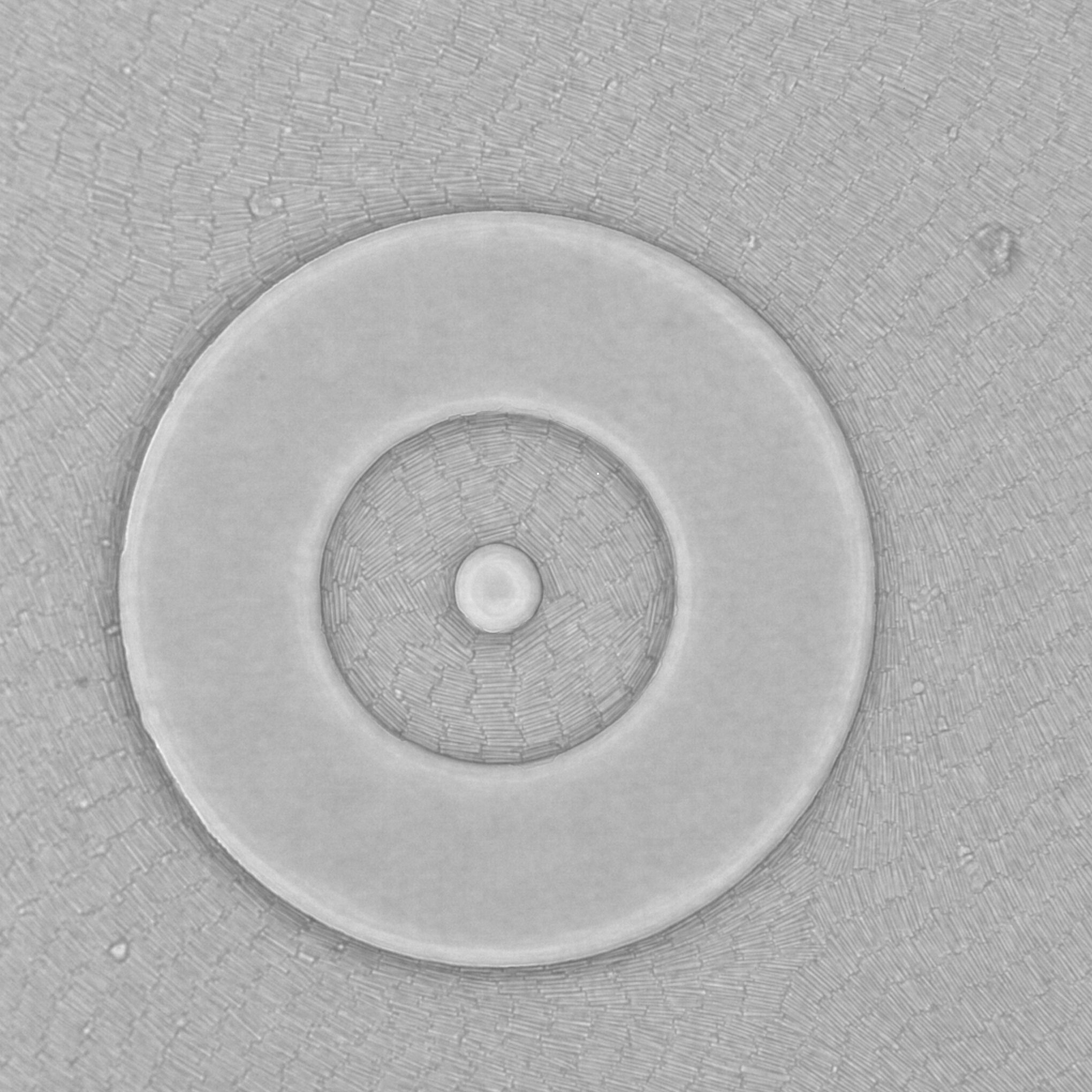

Supplement: Supplementary file 5 — Supplementary Data 2 [file 41467_2020_20842_MOESM5_ESM.zip › rawdata/size4/03_03.tif]

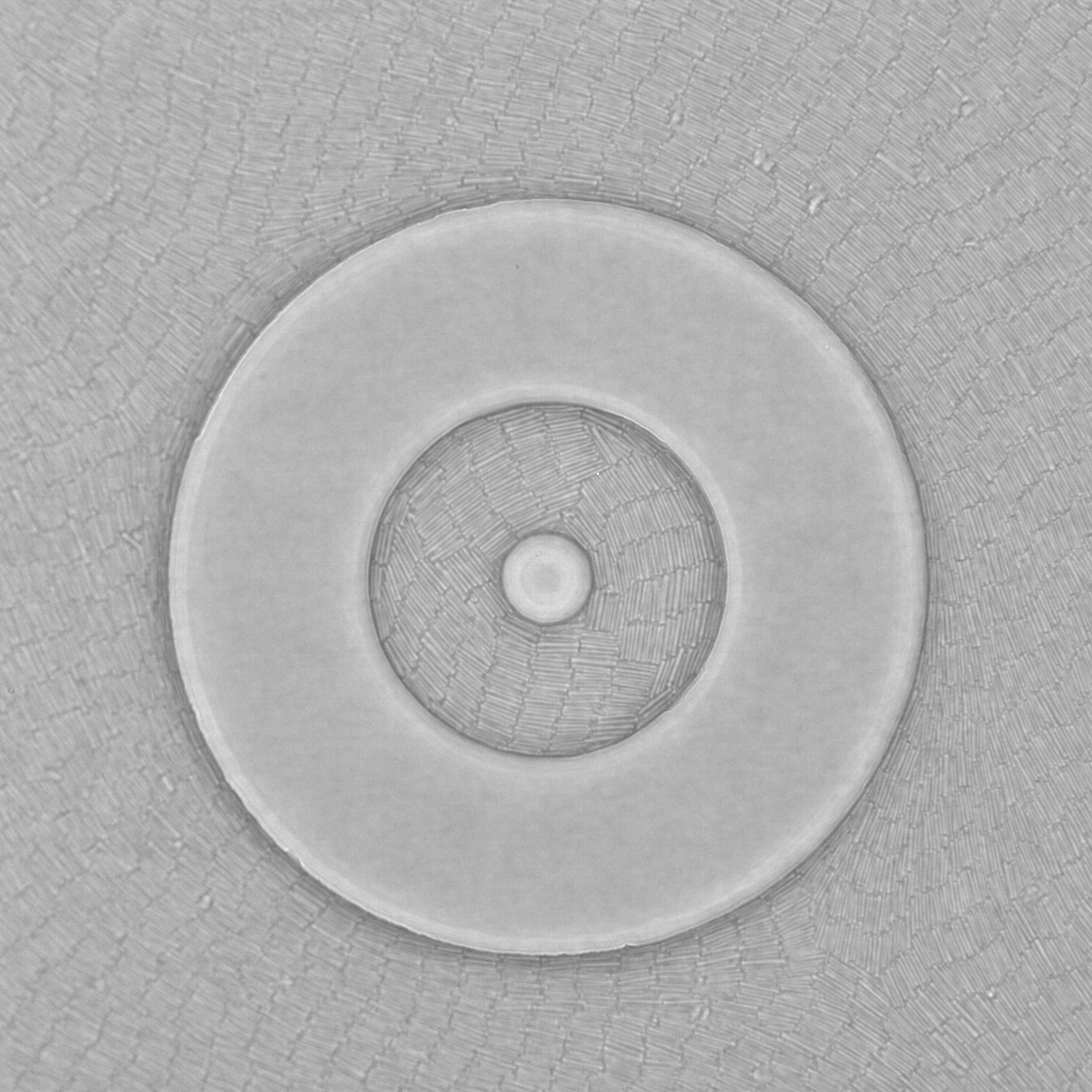

Supplement: Supplementary file 5 — Supplementary Data 2 [file 41467_2020_20842_MOESM5_ESM.zip › rawdata/size4/03_02.tif]

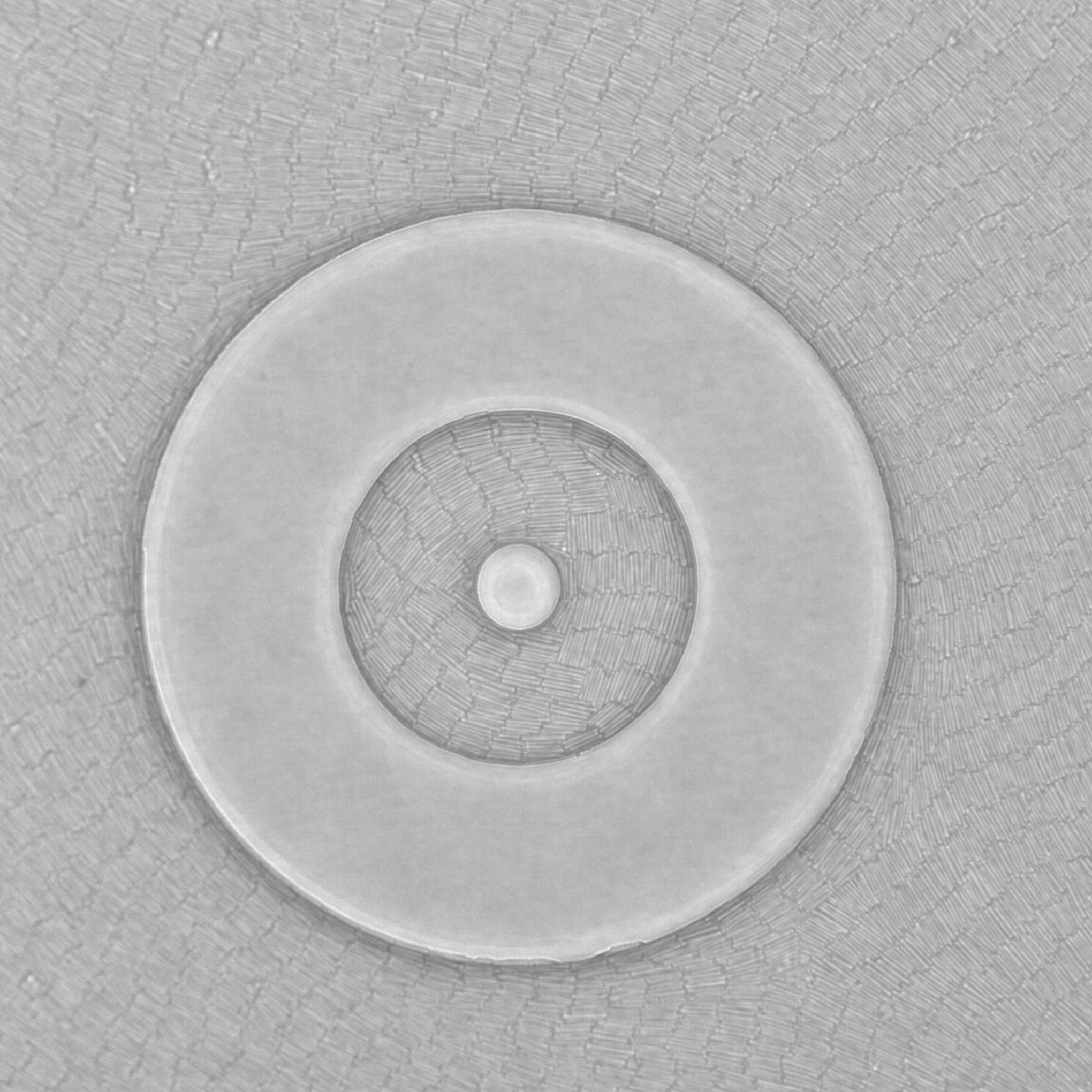

Supplement: Supplementary file 5 — Supplementary Data 2 [file 41467_2020_20842_MOESM5_ESM.zip › rawdata/size4/03_01.tif]

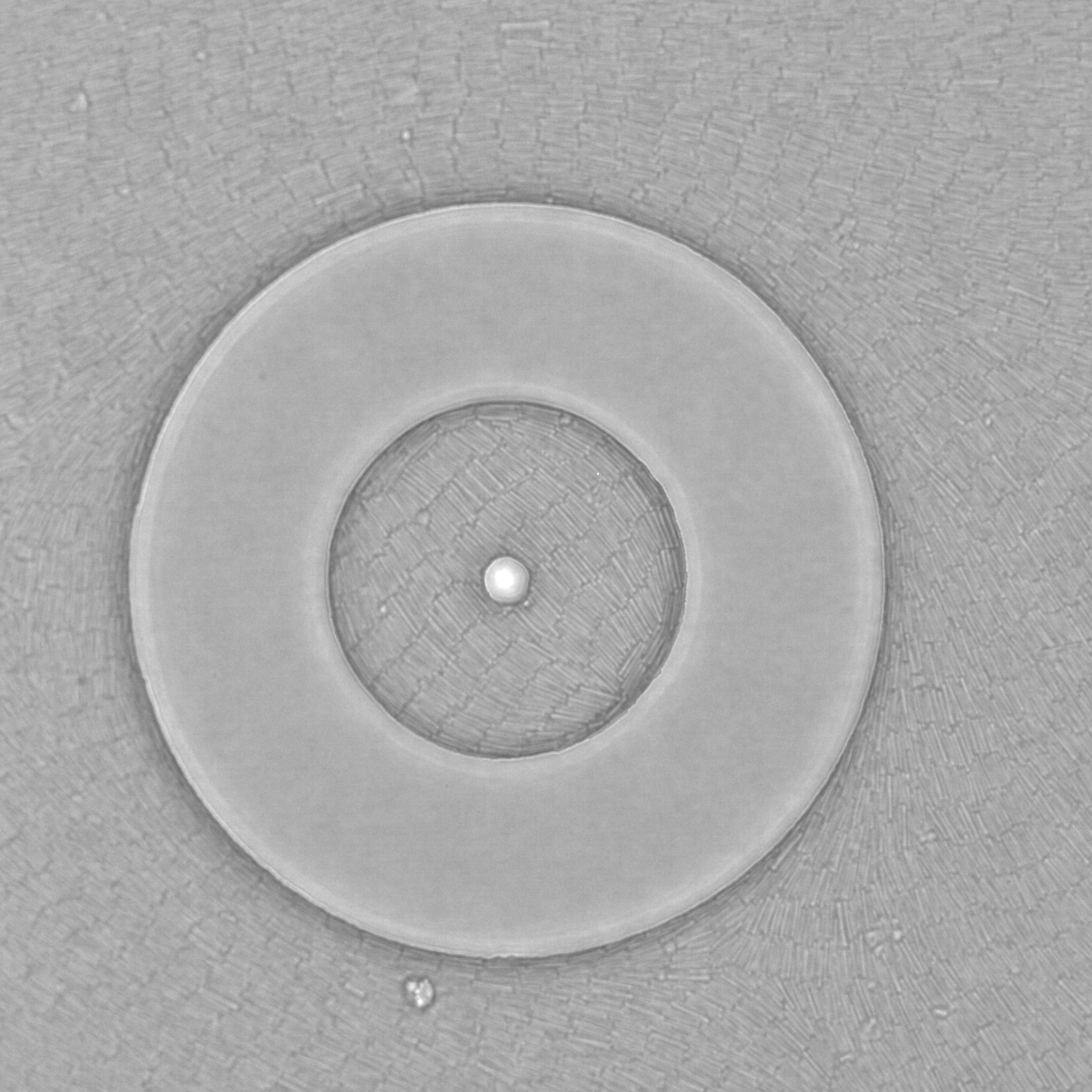

Supplement: Supplementary file 5 — Supplementary Data 2 [file 41467_2020_20842_MOESM5_ESM.zip › rawdata/size4/02_06.tif]

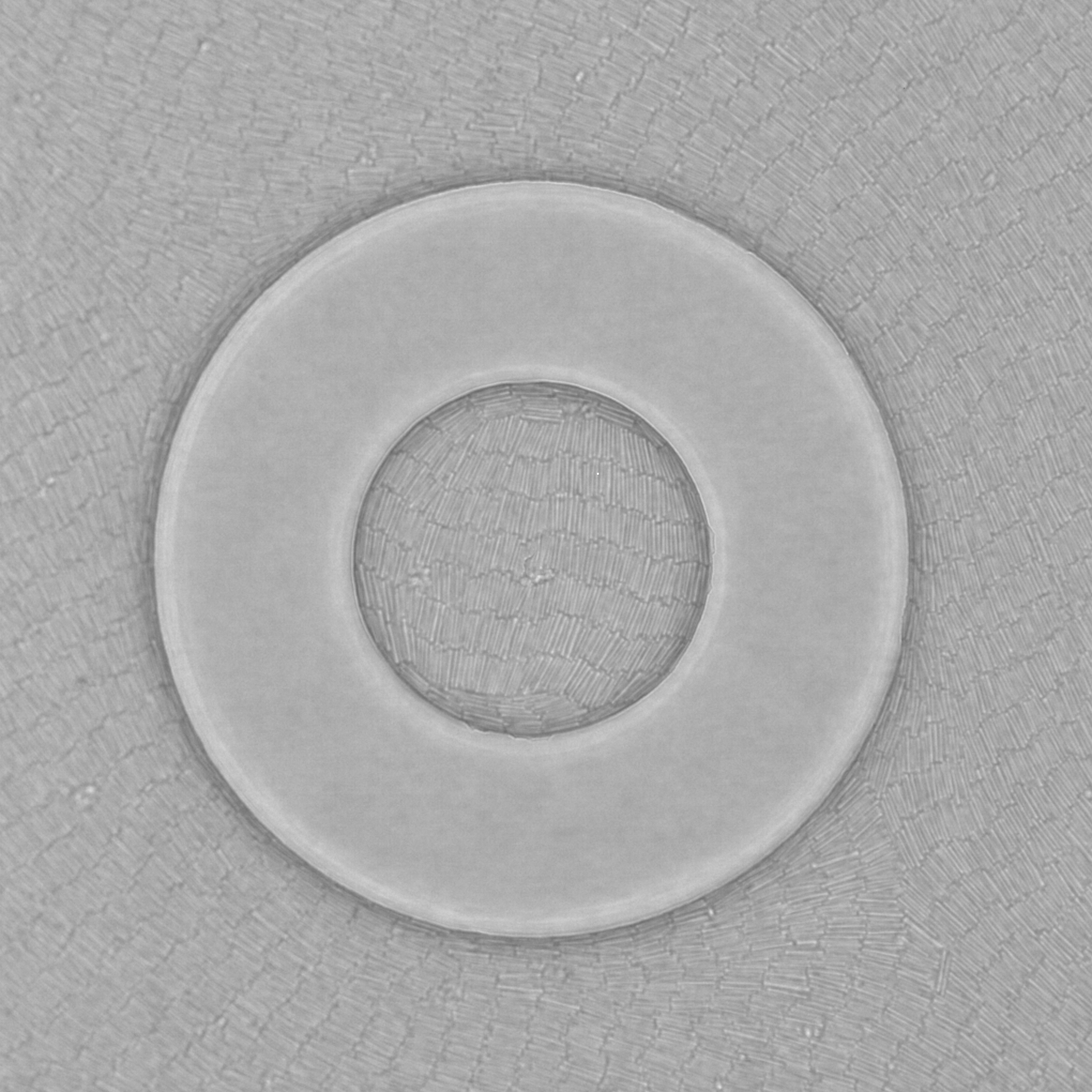

Supplement: Supplementary file 5 — Supplementary Data 2 [file 41467_2020_20842_MOESM5_ESM.zip › rawdata/size4/02_05.tif]

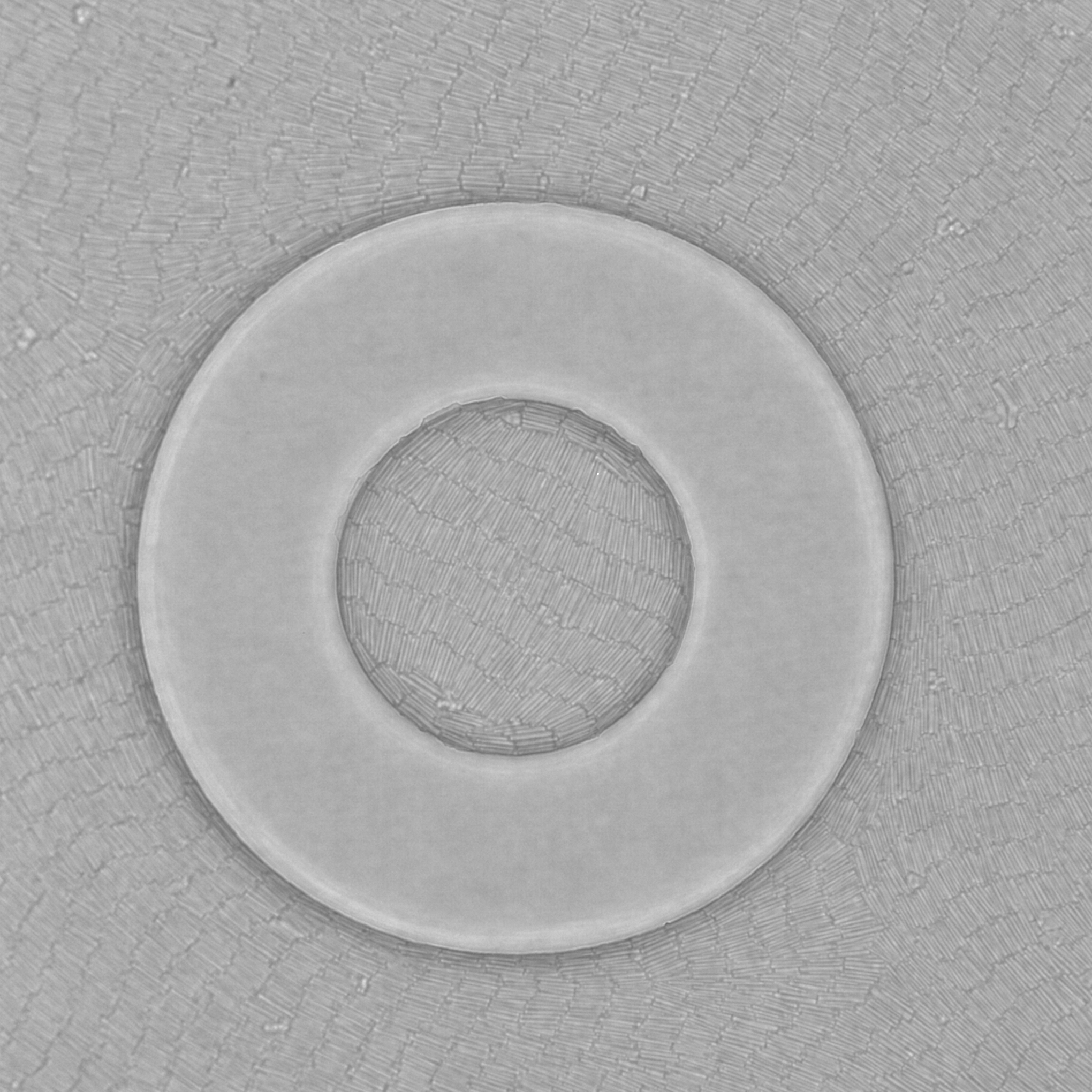

Supplement: Supplementary file 5 — Supplementary Data 2 [file 41467_2020_20842_MOESM5_ESM.zip › rawdata/size4/02_04.tif]

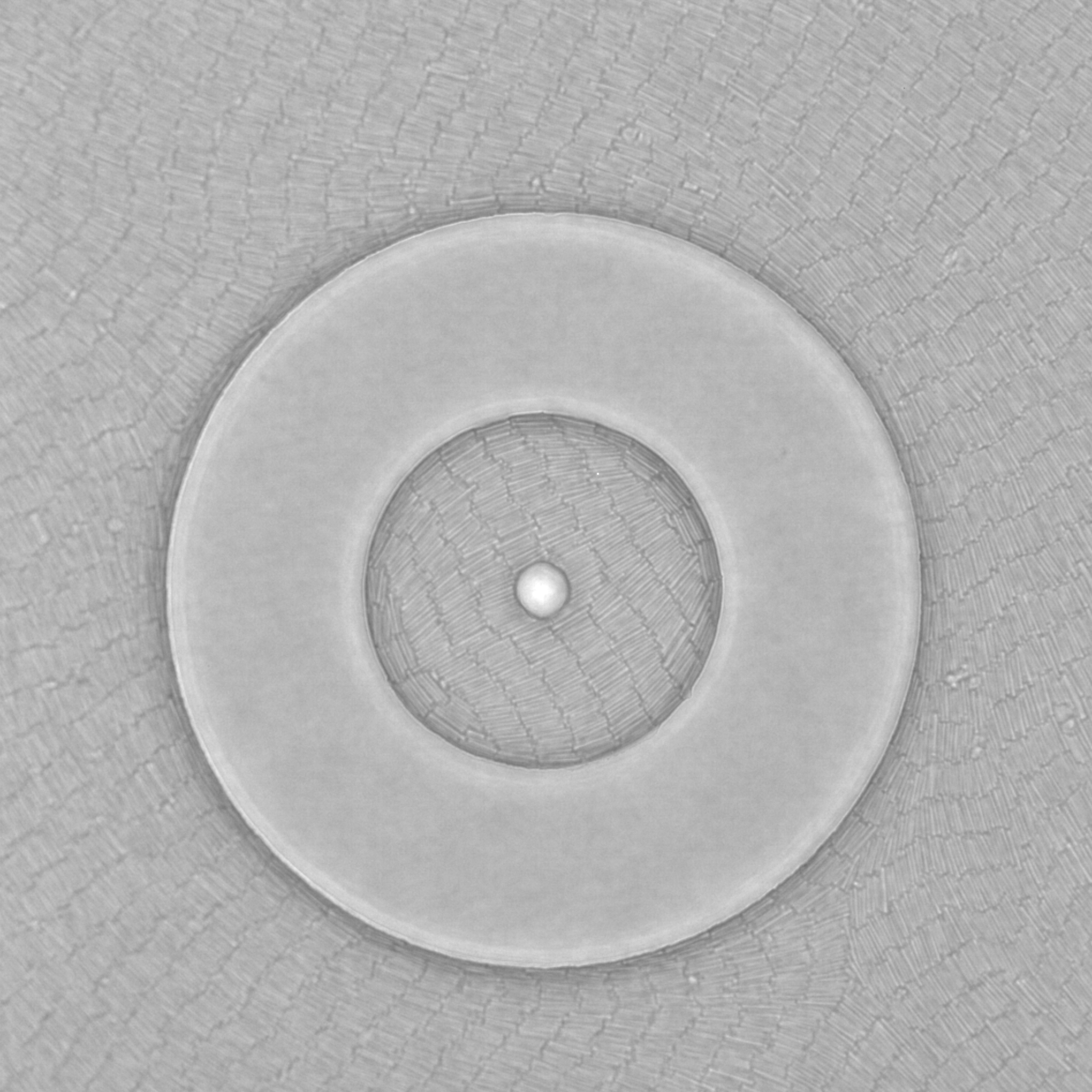

Supplement: Supplementary file 5 — Supplementary Data 2 [file 41467_2020_20842_MOESM5_ESM.zip › rawdata/size4/02_03.tif]

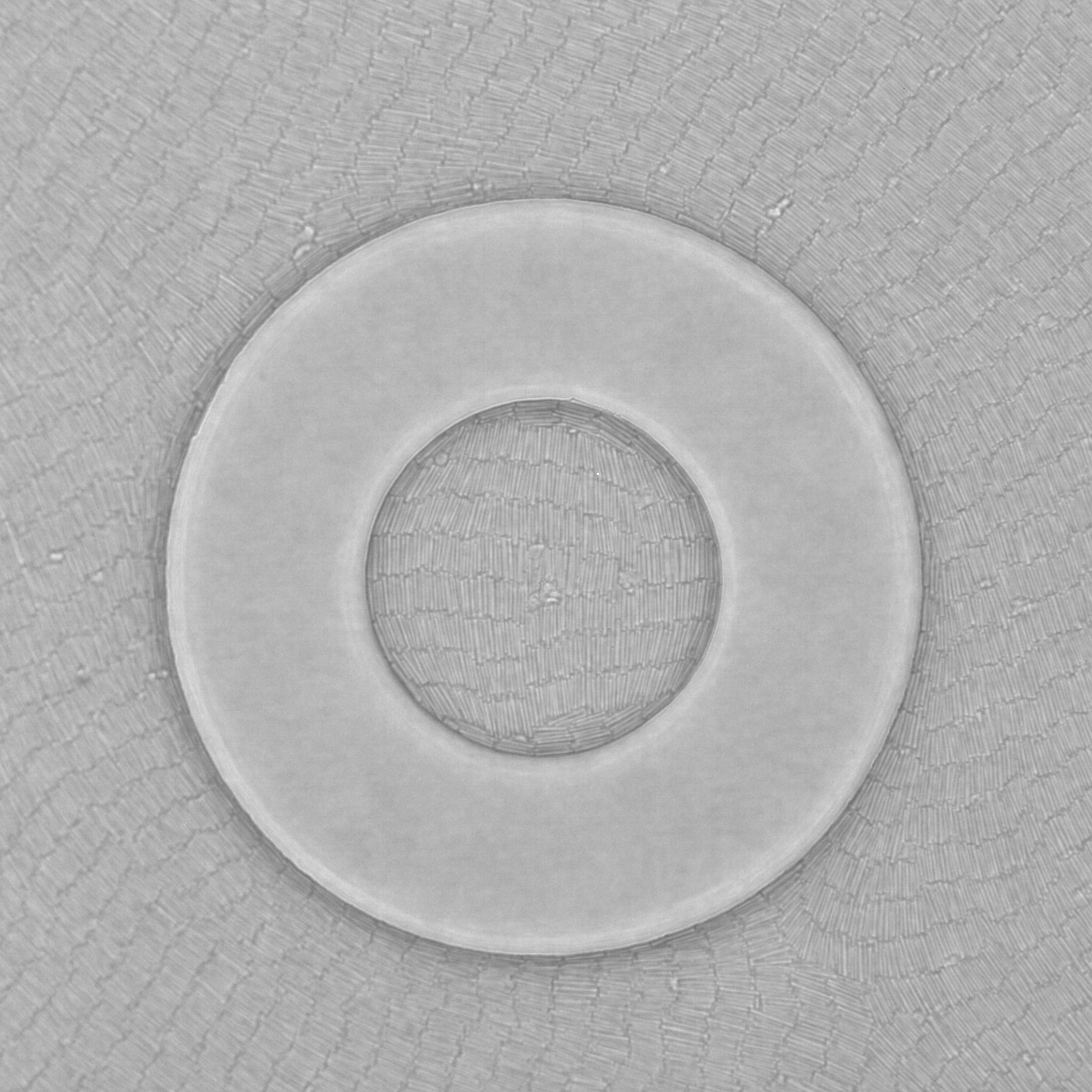

Supplement: Supplementary file 5 — Supplementary Data 2 [file 41467_2020_20842_MOESM5_ESM.zip › rawdata/size4/02_02.tif]

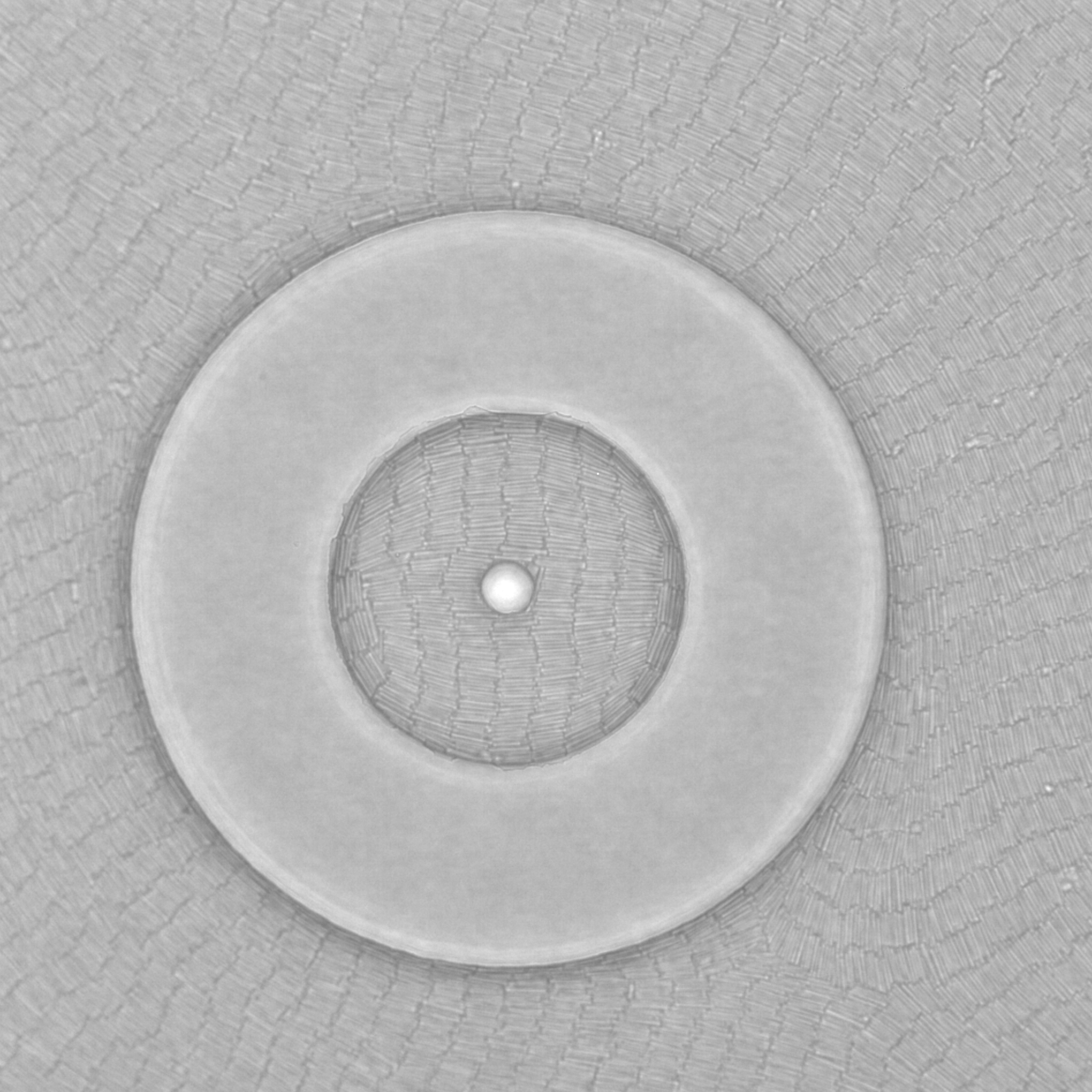

Supplement: Supplementary file 5 — Supplementary Data 2 [file 41467_2020_20842_MOESM5_ESM.zip › rawdata/size4/02_01.tif]

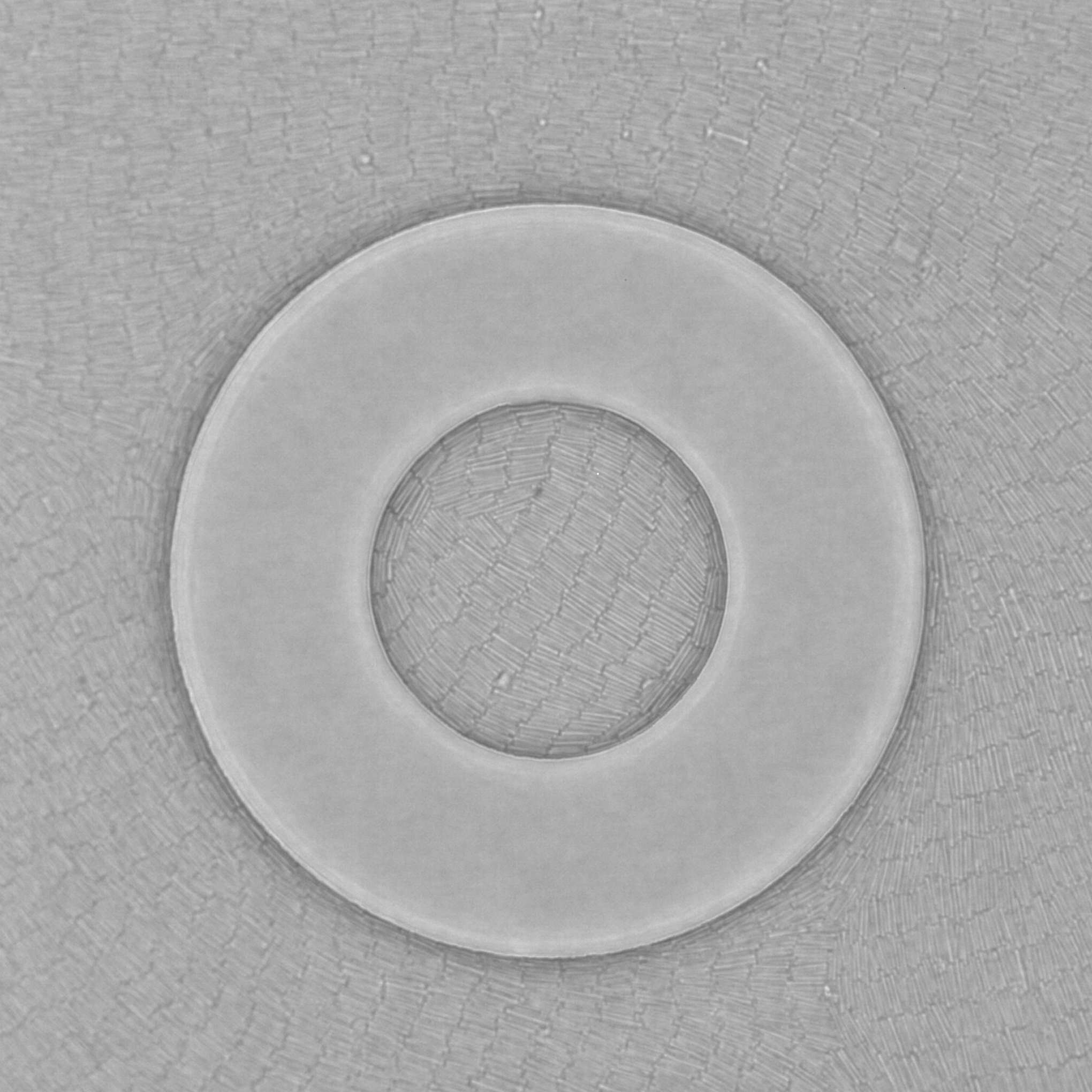

Supplement: Supplementary file 5 — Supplementary Data 2 [file 41467_2020_20842_MOESM5_ESM.zip › rawdata/size4/01_06.tif]

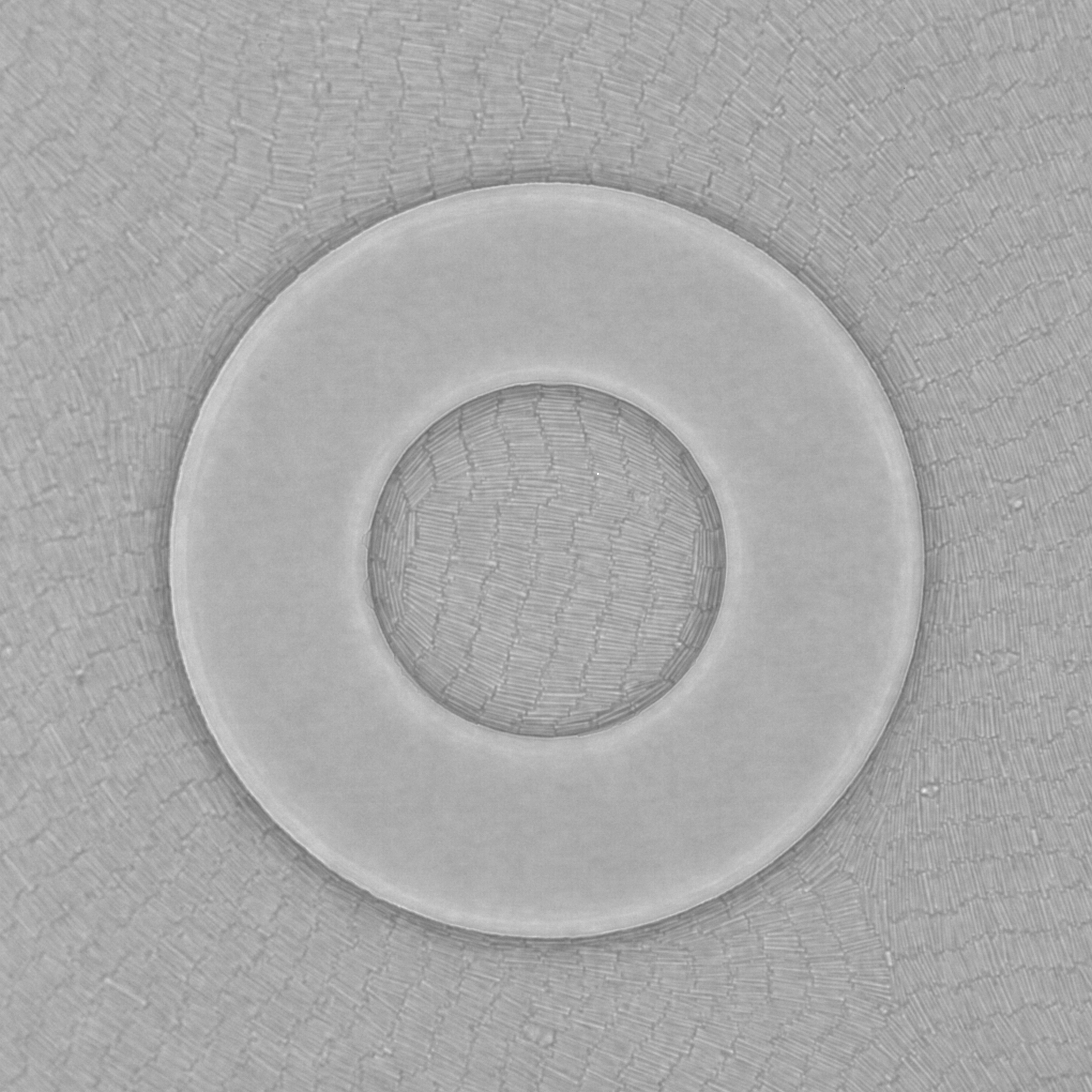

Supplement: Supplementary file 5 — Supplementary Data 2 [file 41467_2020_20842_MOESM5_ESM.zip › rawdata/size4/01_05.tif]

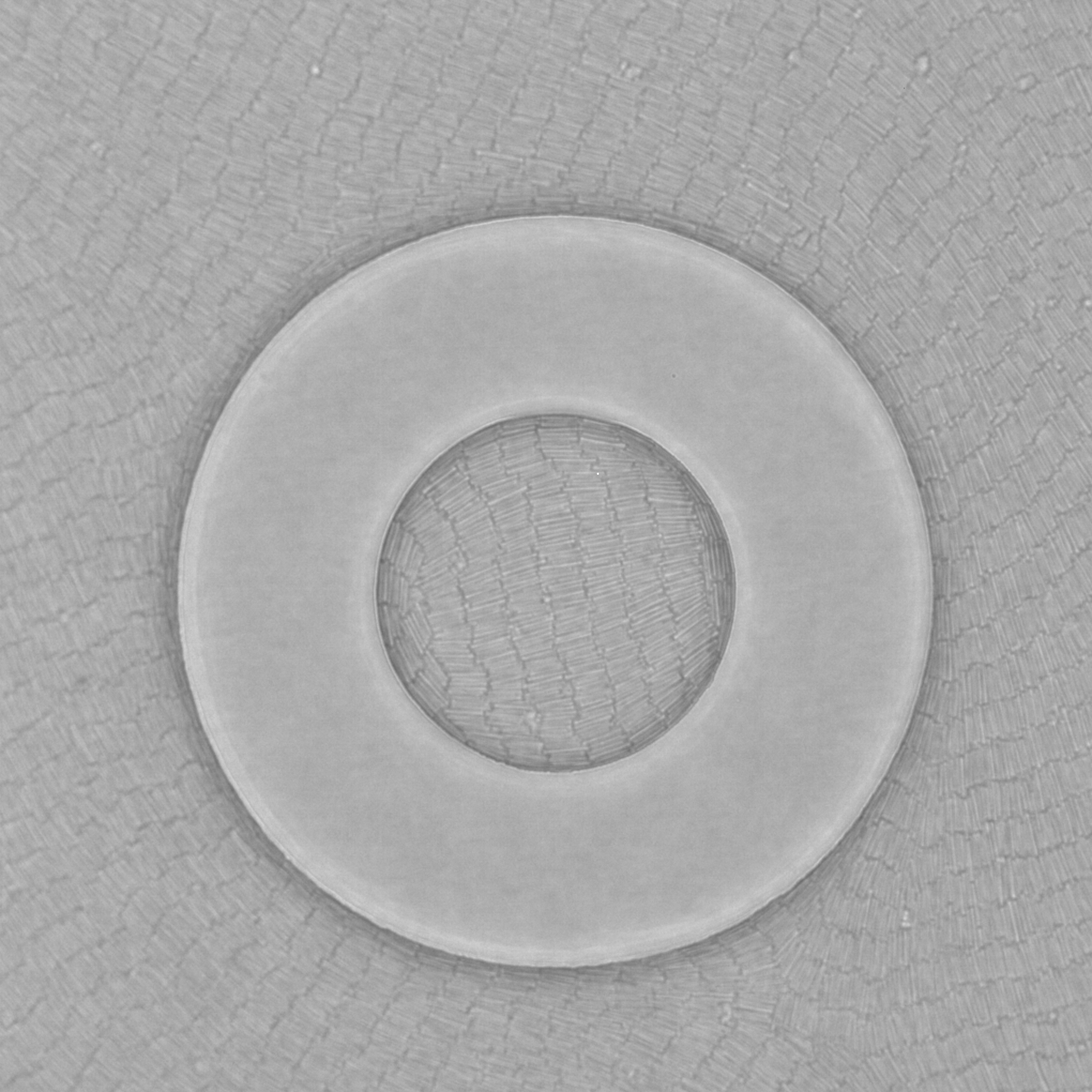

Supplement: Supplementary file 5 — Supplementary Data 2 [file 41467_2020_20842_MOESM5_ESM.zip › rawdata/size4/01_04.tif]

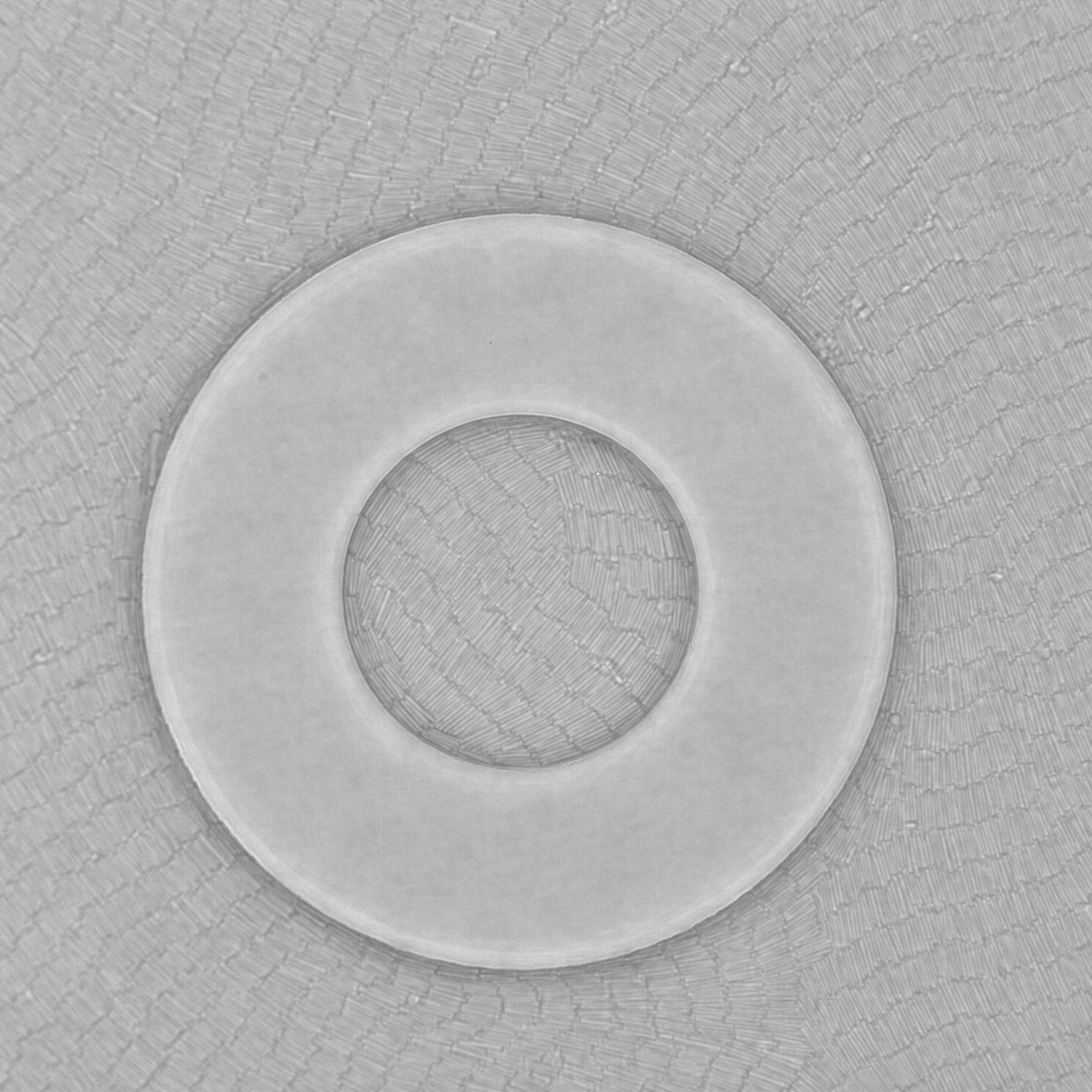

Supplement: Supplementary file 5 — Supplementary Data 2 [file 41467_2020_20842_MOESM5_ESM.zip › rawdata/size4/01_03.tif]

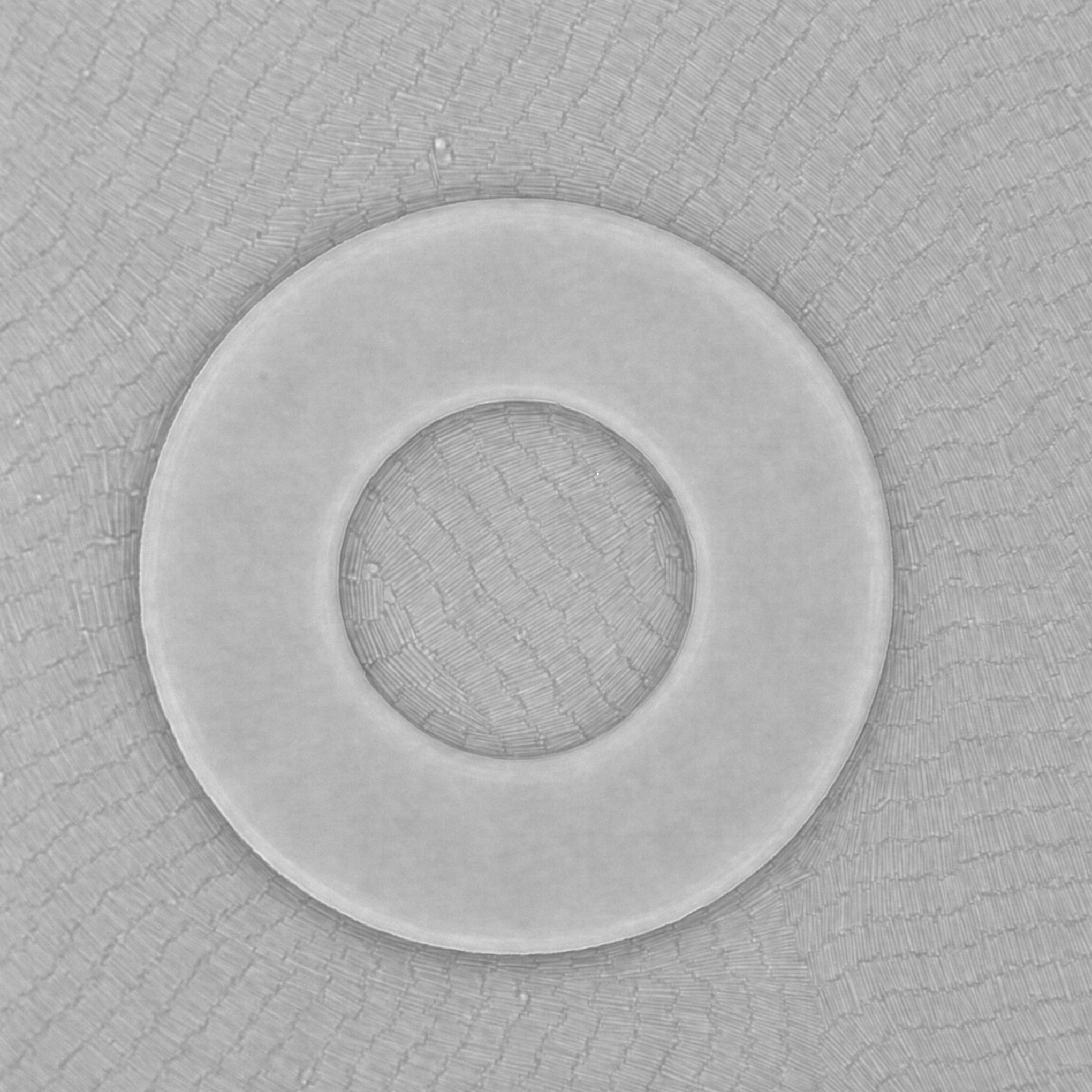

Supplement: Supplementary file 5 — Supplementary Data 2 [file 41467_2020_20842_MOESM5_ESM.zip › rawdata/size4/01_02.tif]

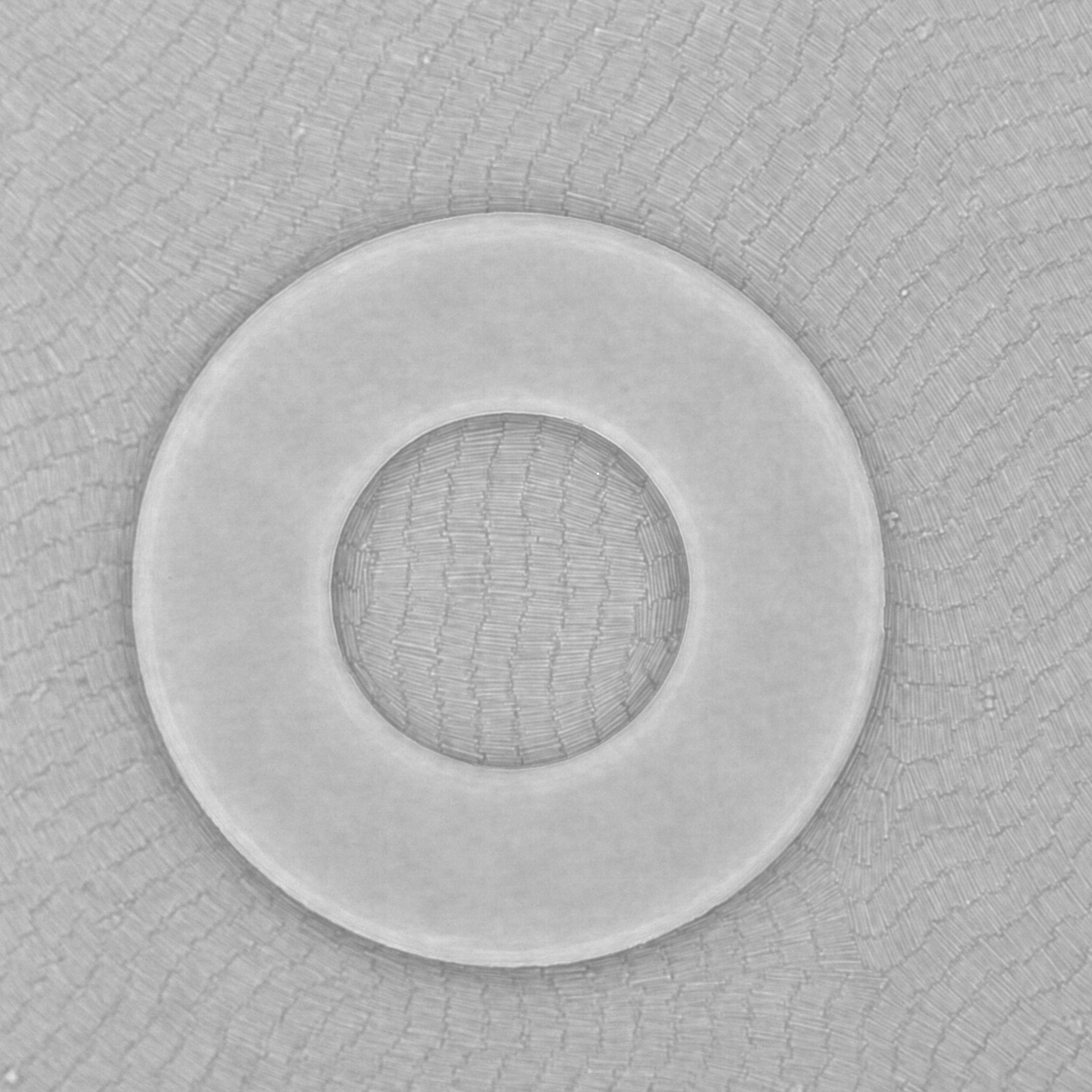

Supplement: Supplementary file 5 — Supplementary Data 2 [file 41467_2020_20842_MOESM5_ESM.zip › rawdata/size4/01_01.tif]

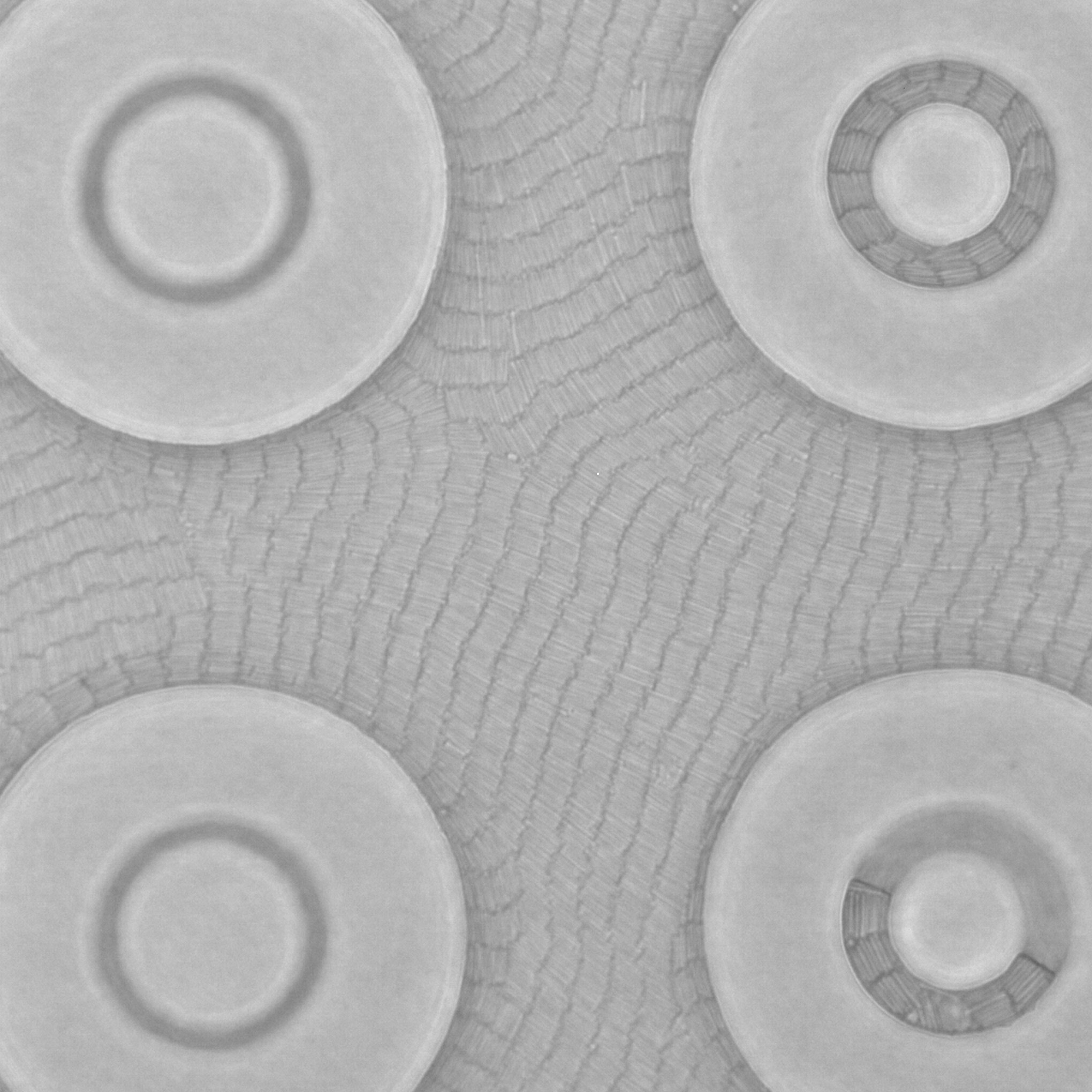

Supplement: Supplementary file 5 — Supplementary Data 2 [file 41467_2020_20842_MOESM5_ESM.zip › rawdata/size3/03_06.tif]

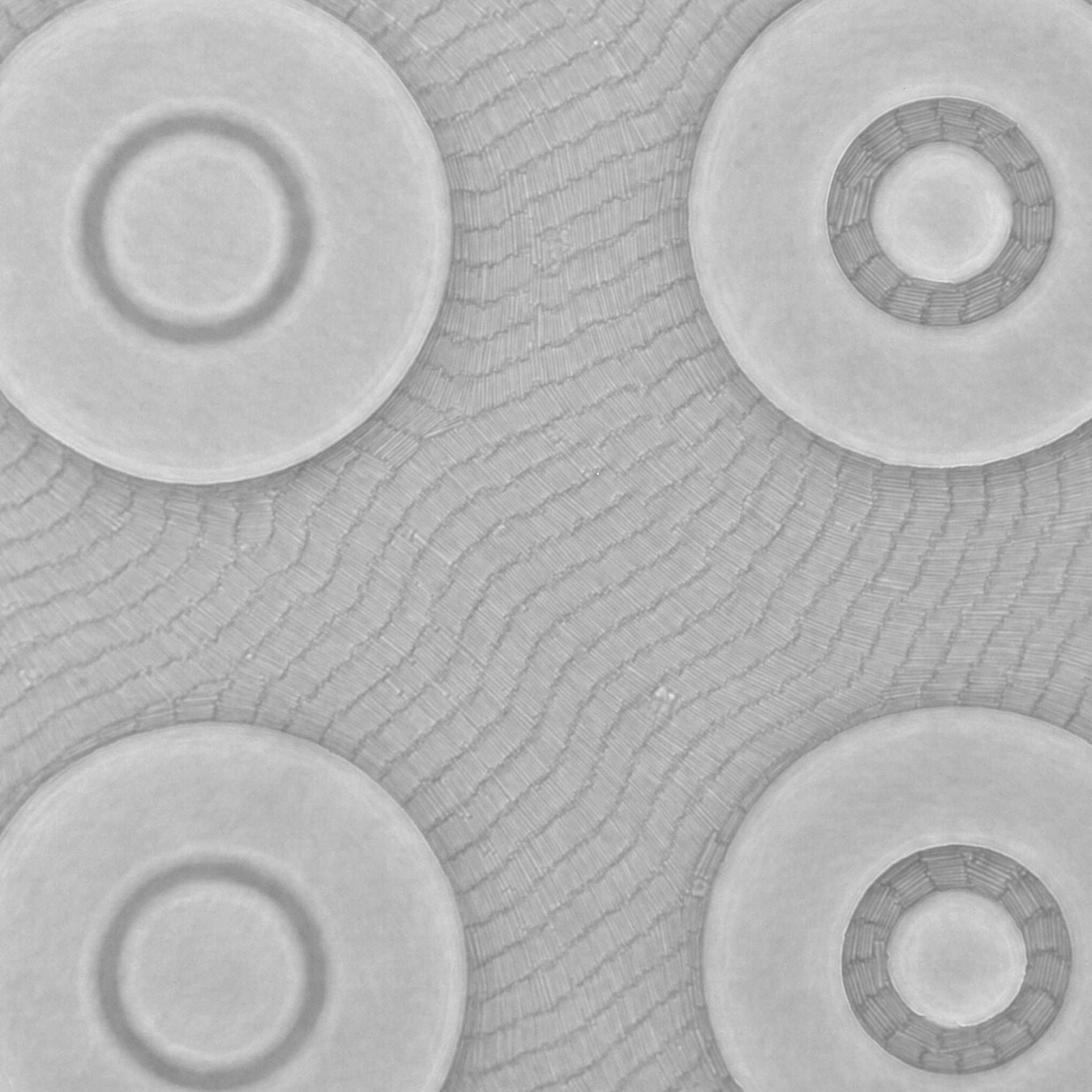

Supplement: Supplementary file 5 — Supplementary Data 2 [file 41467_2020_20842_MOESM5_ESM.zip › rawdata/size3/03_05.tif]

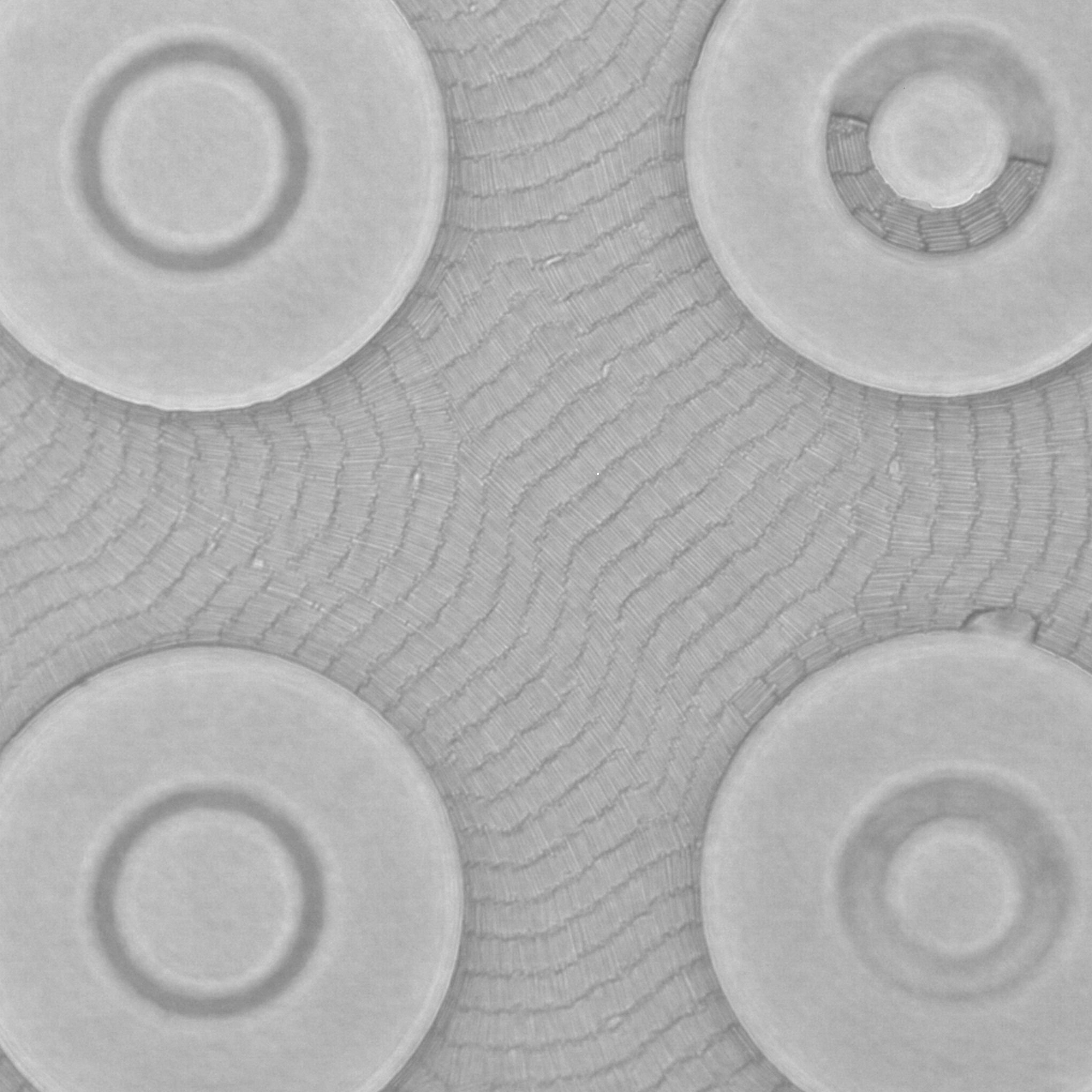

Supplement: Supplementary file 5 — Supplementary Data 2 [file 41467_2020_20842_MOESM5_ESM.zip › rawdata/size3/03_04.tif]

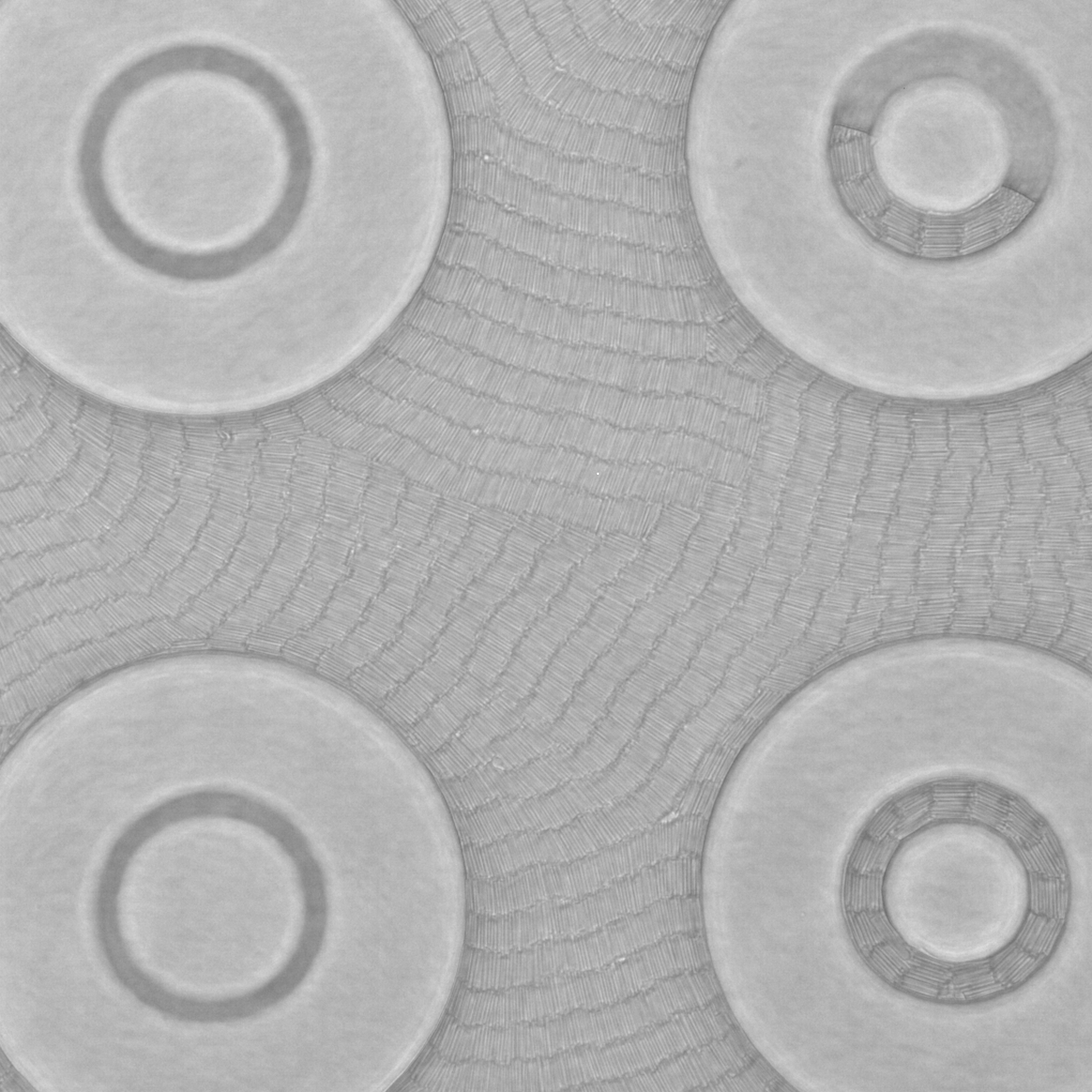

Supplement: Supplementary file 5 — Supplementary Data 2 [file 41467_2020_20842_MOESM5_ESM.zip › rawdata/size3/03_03.tif]

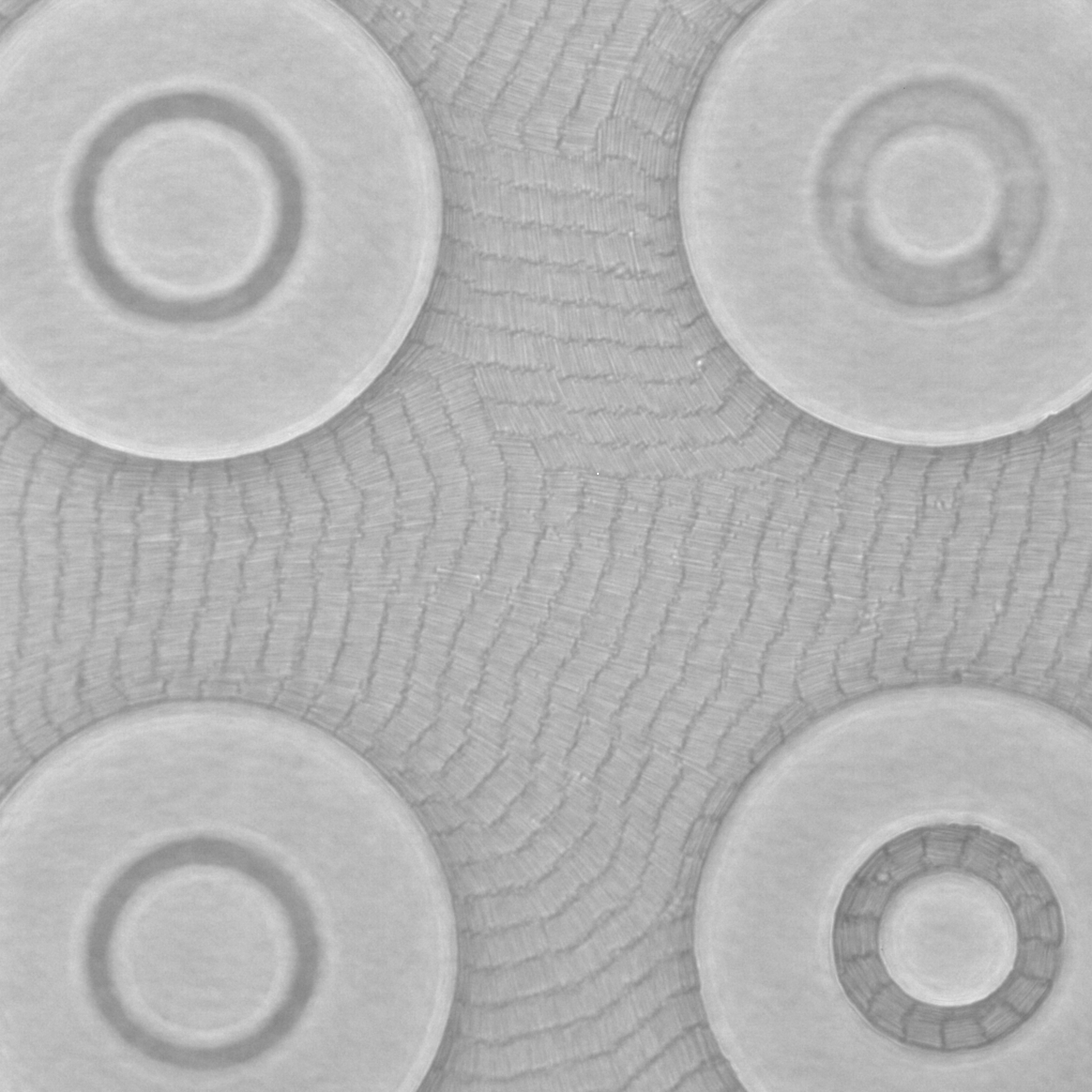

Supplement: Supplementary file 5 — Supplementary Data 2 [file 41467_2020_20842_MOESM5_ESM.zip › rawdata/size3/03_02.tif]

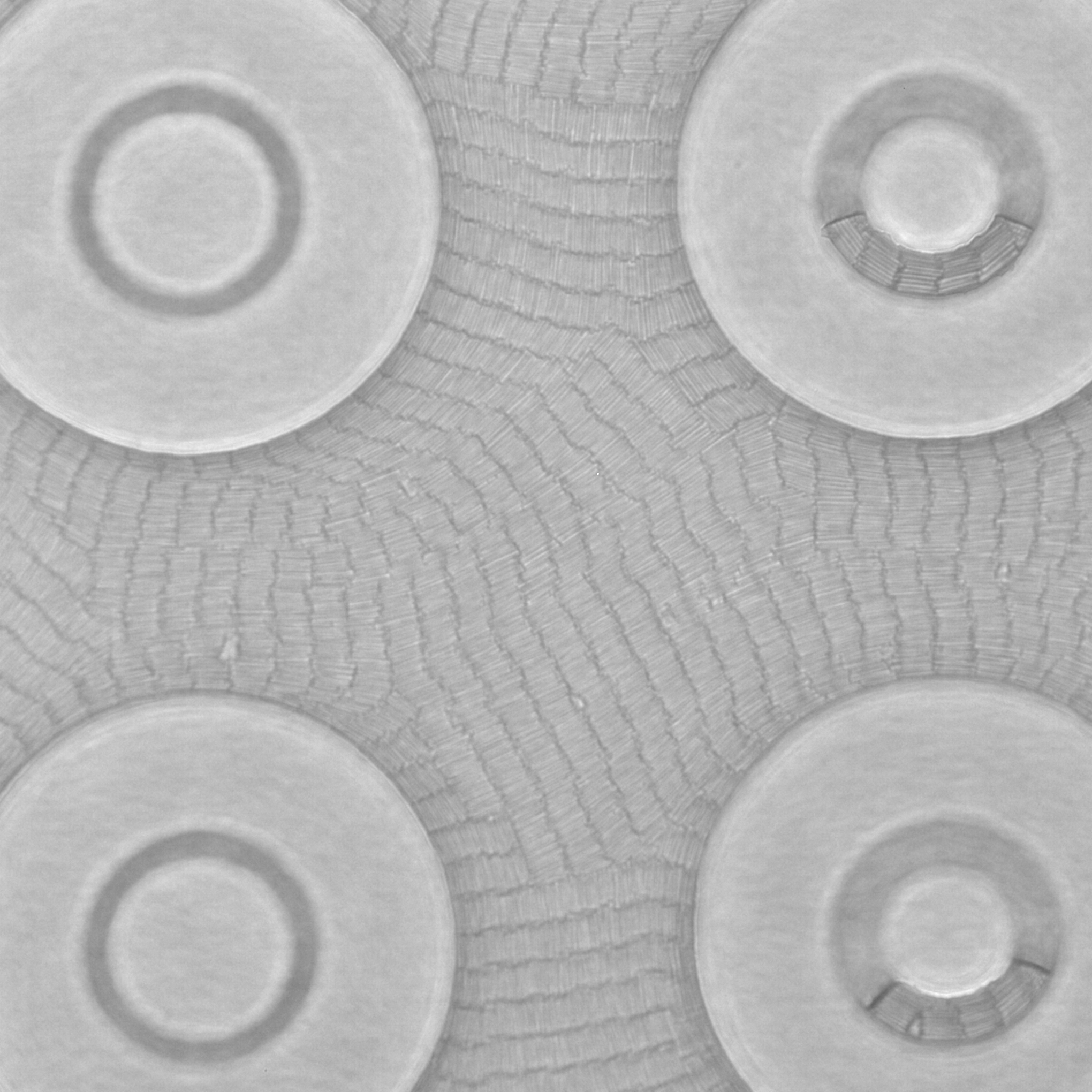

Supplement: Supplementary file 5 — Supplementary Data 2 [file 41467_2020_20842_MOESM5_ESM.zip › rawdata/size3/03_01.tif]

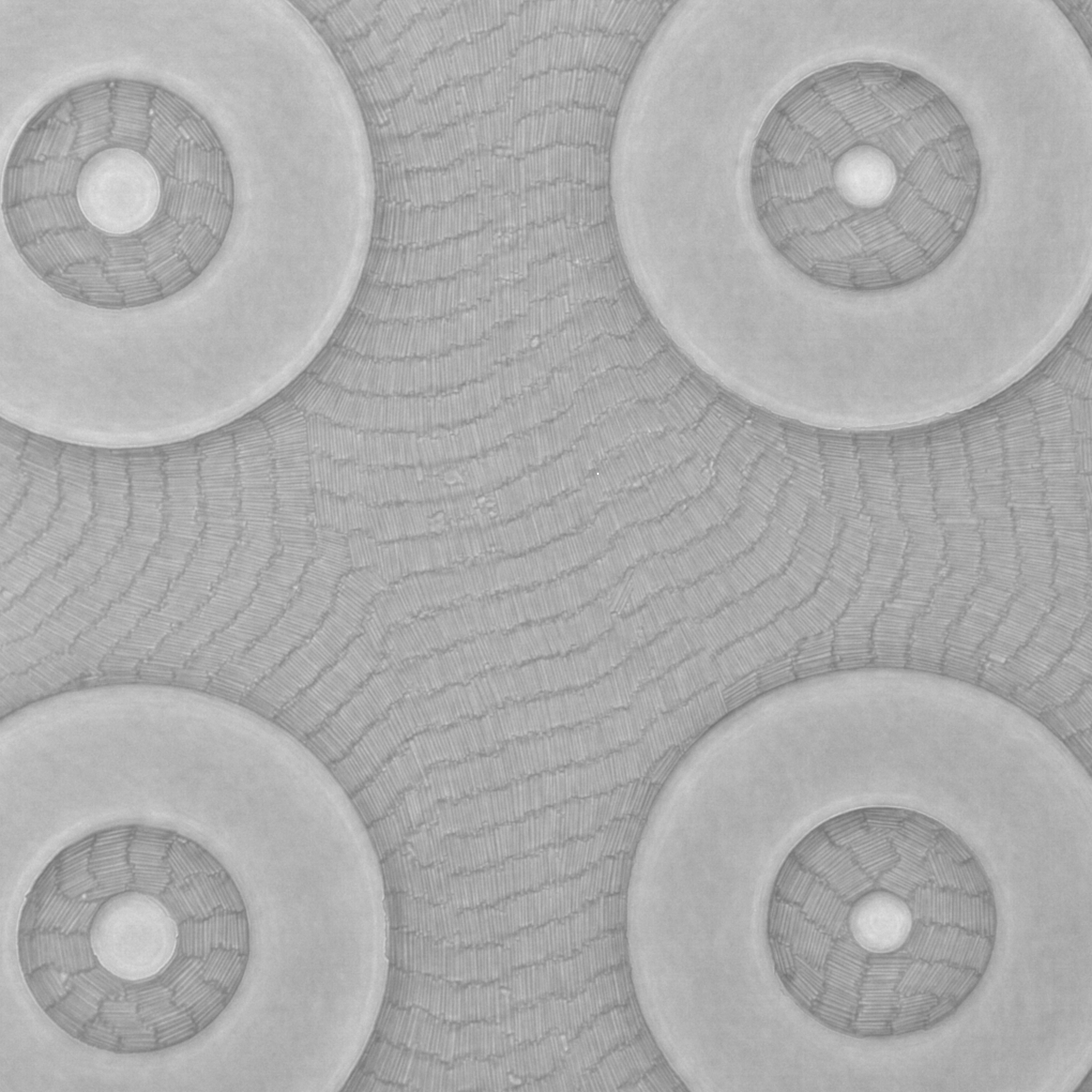

Supplement: Supplementary file 5 — Supplementary Data 2 [file 41467_2020_20842_MOESM5_ESM.zip › rawdata/size3/02_06.tif]

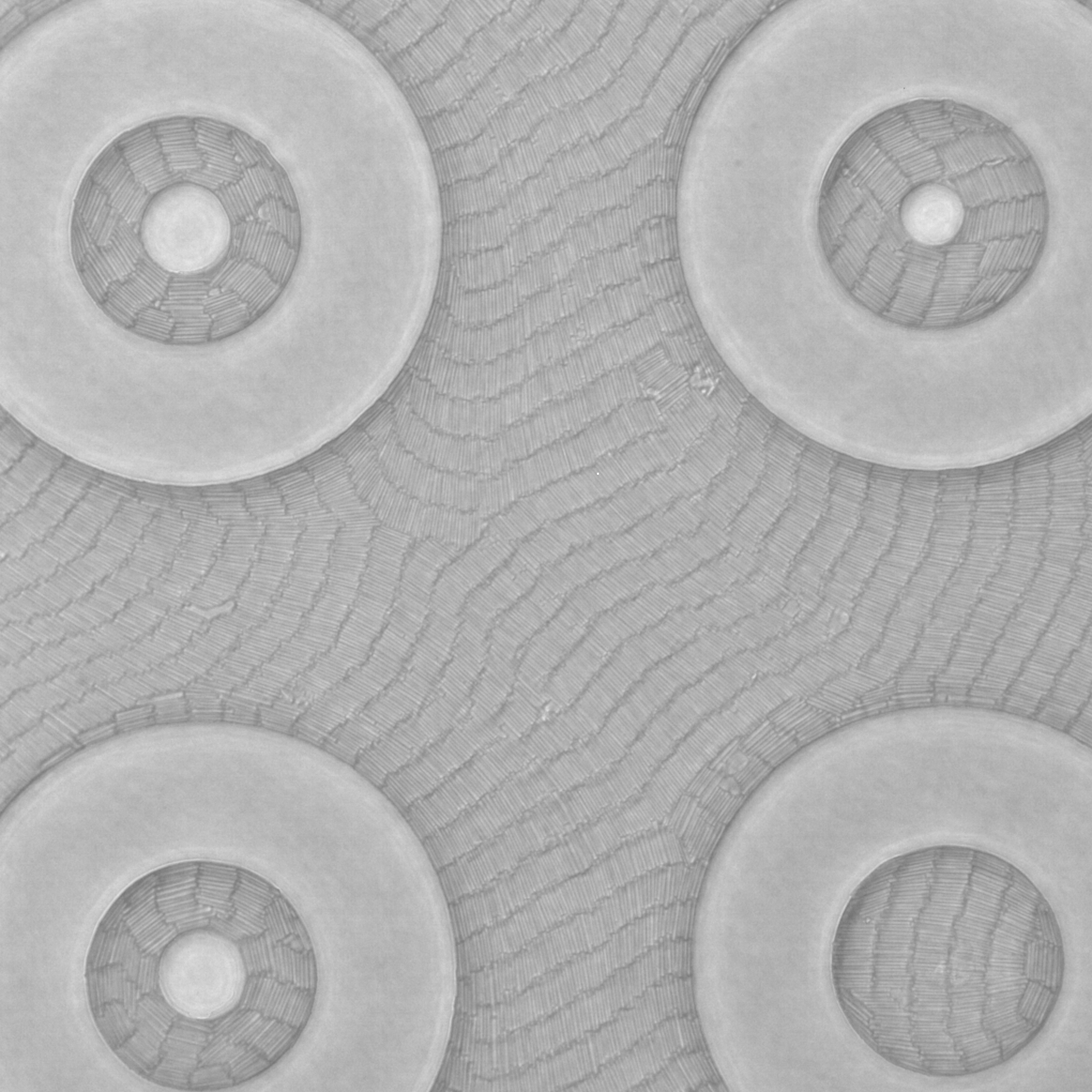

Supplement: Supplementary file 5 — Supplementary Data 2 [file 41467_2020_20842_MOESM5_ESM.zip › rawdata/size3/02_05.tif]

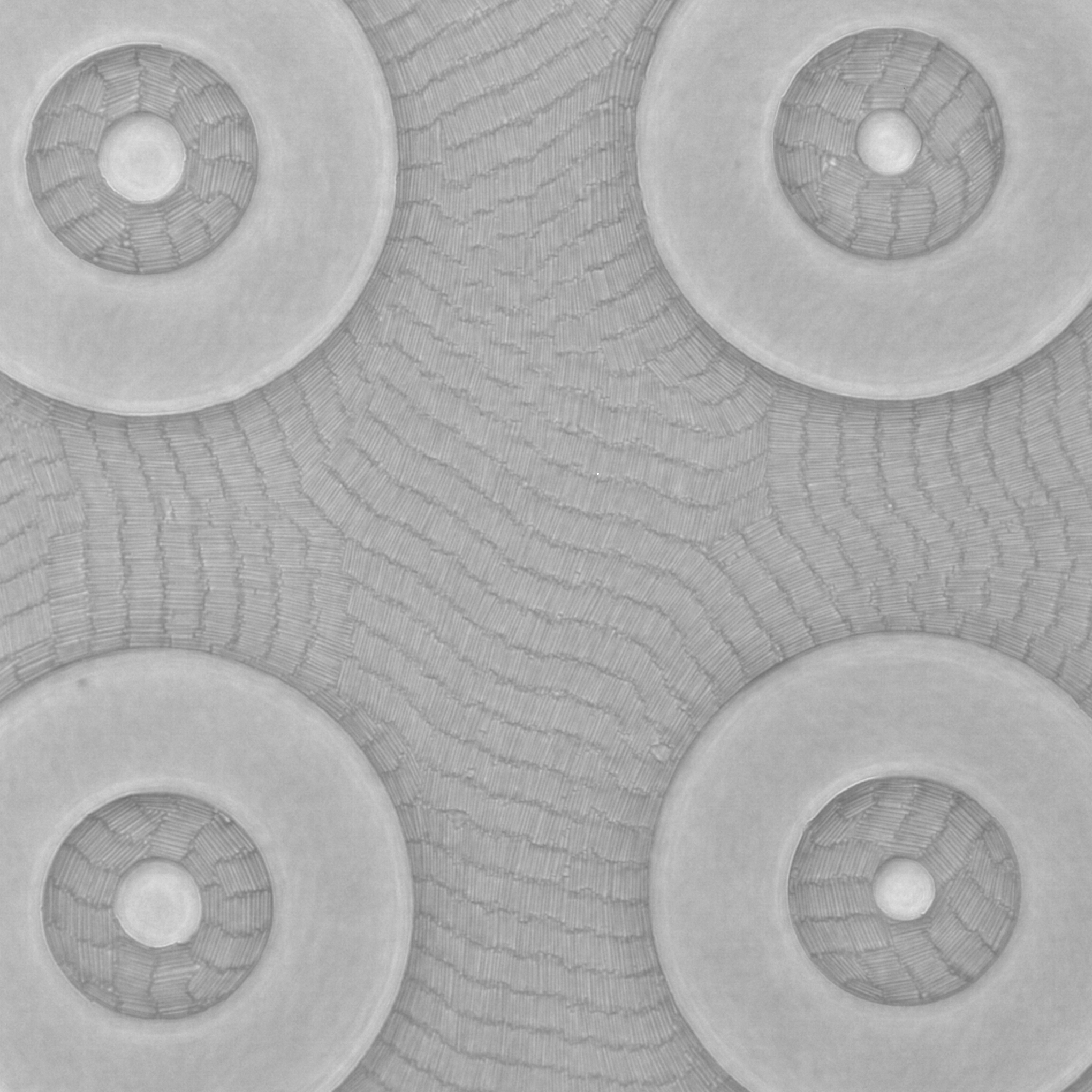

Supplement: Supplementary file 5 — Supplementary Data 2 [file 41467_2020_20842_MOESM5_ESM.zip › rawdata/size3/02_04.tif]

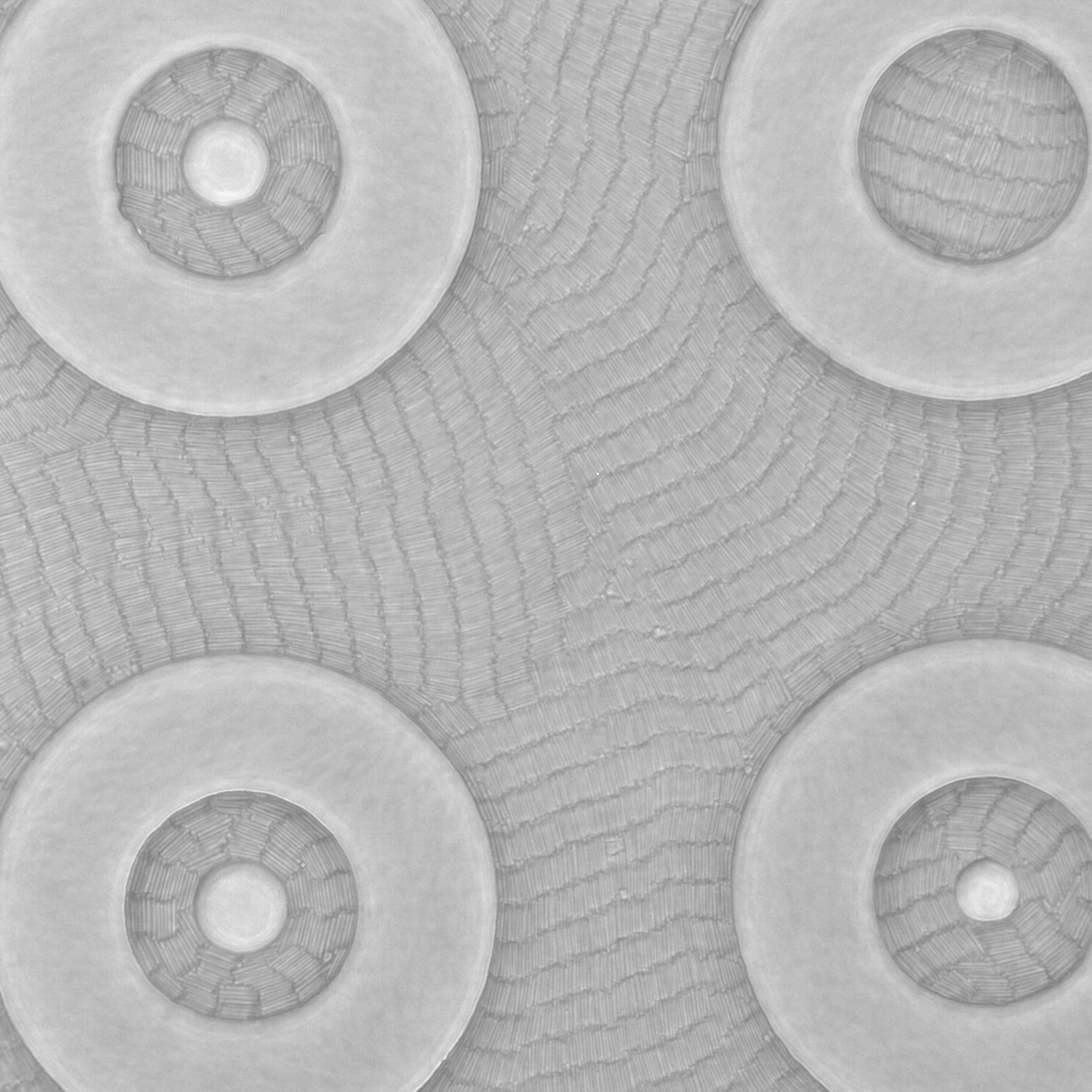

Supplement: Supplementary file 5 — Supplementary Data 2 [file 41467_2020_20842_MOESM5_ESM.zip › rawdata/size3/02_03.tif]

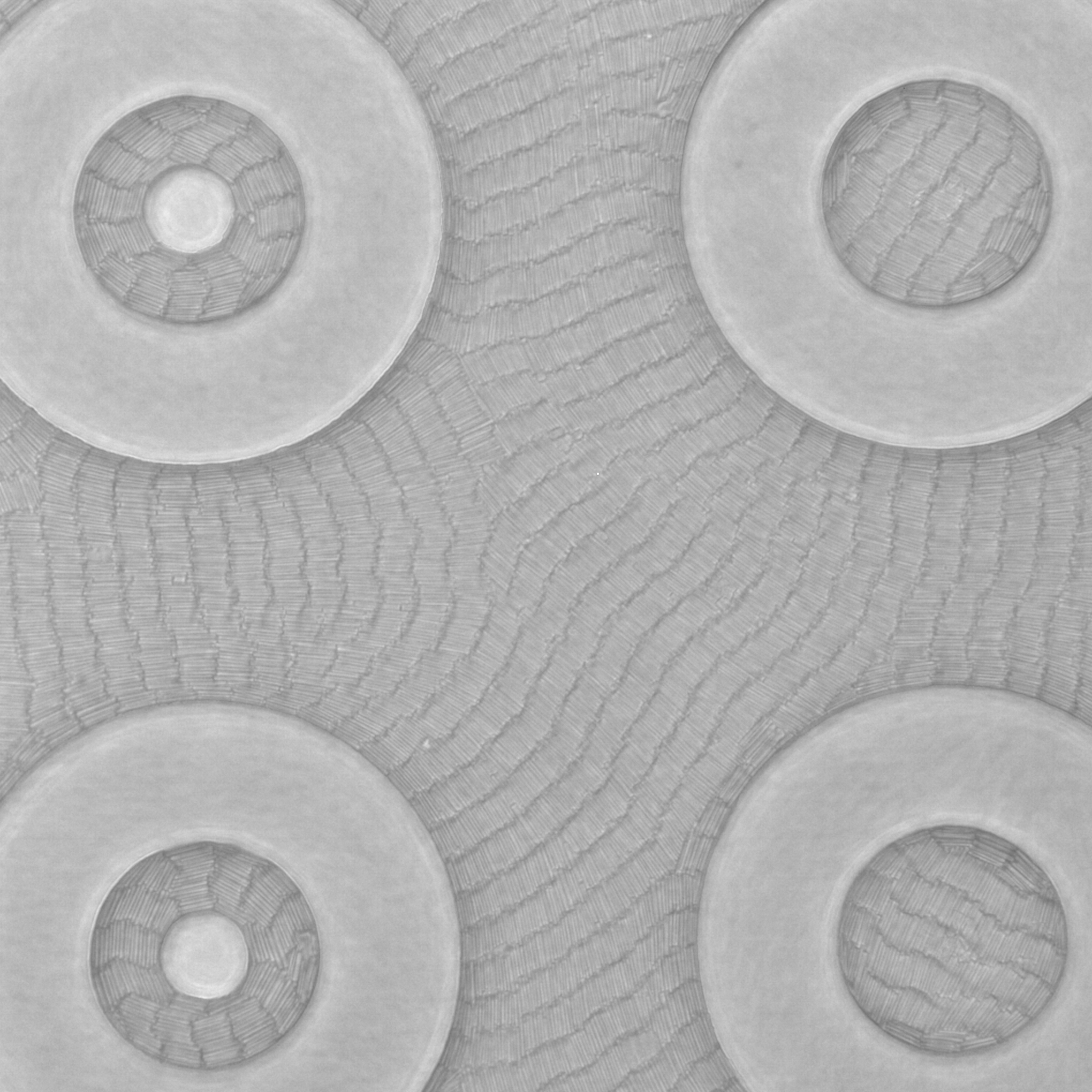

Supplement: Supplementary file 5 — Supplementary Data 2 [file 41467_2020_20842_MOESM5_ESM.zip › rawdata/size3/02_02.tif]

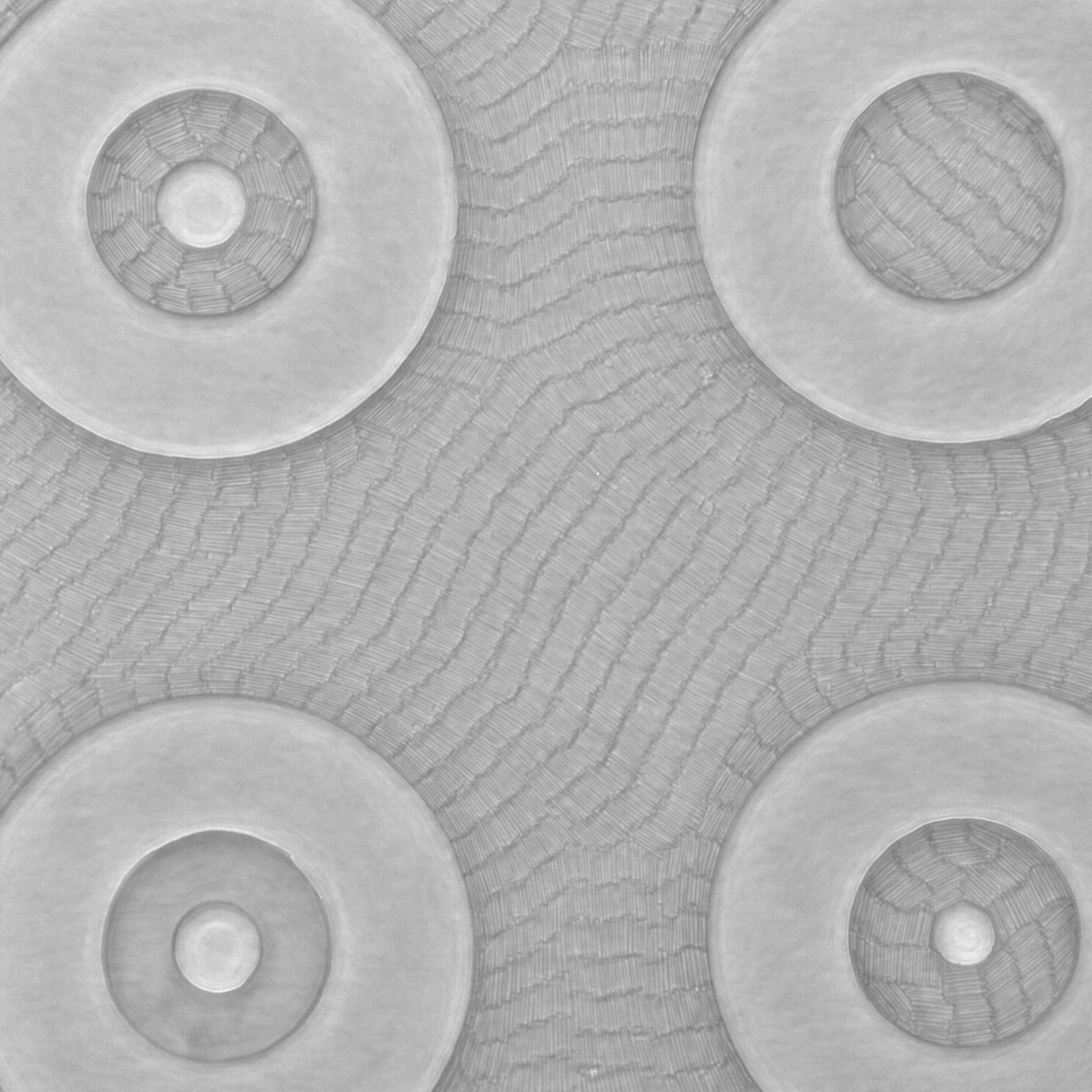

Supplement: Supplementary file 5 — Supplementary Data 2 [file 41467_2020_20842_MOESM5_ESM.zip › rawdata/size3/02_01.tif]

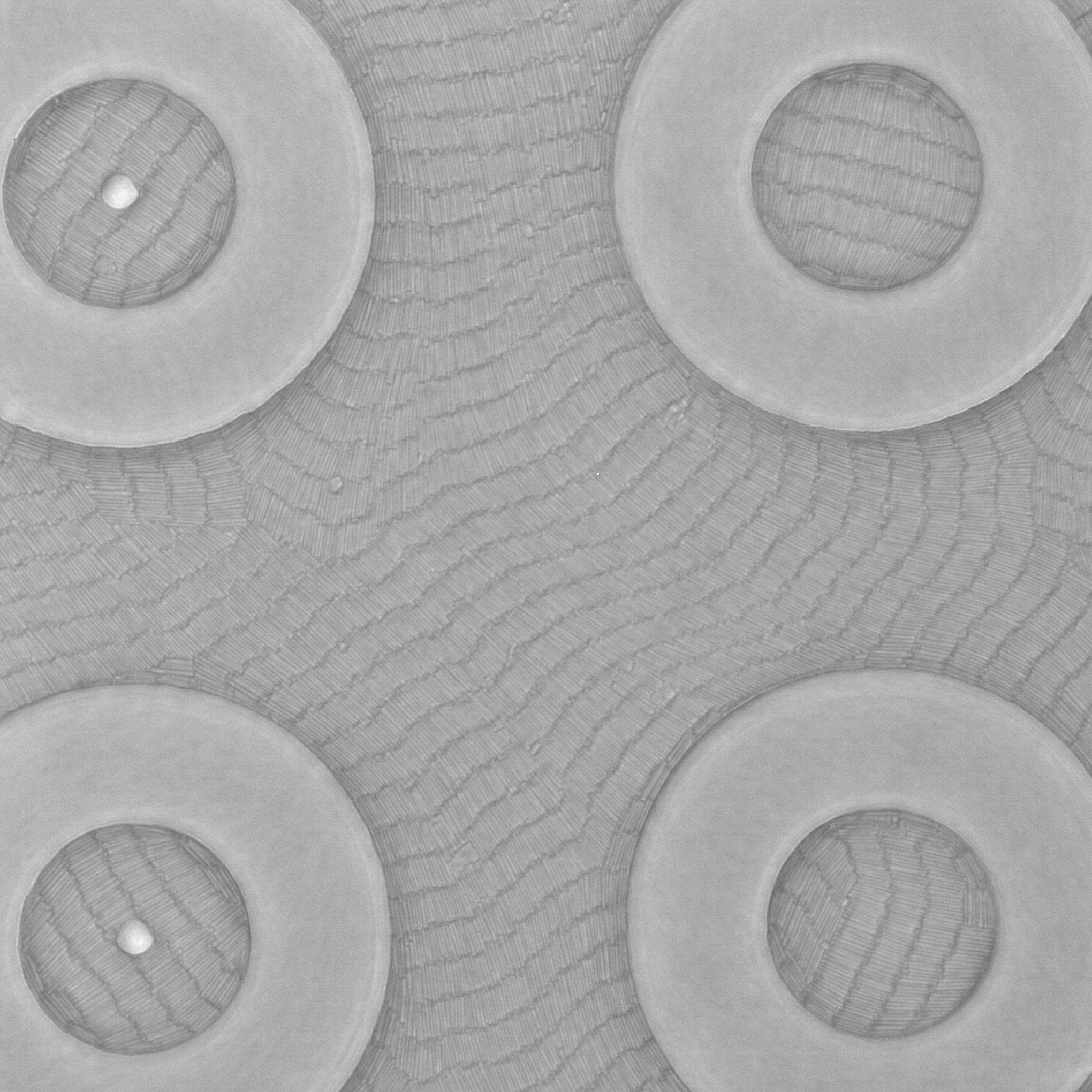

Supplement: Supplementary file 5 — Supplementary Data 2 [file 41467_2020_20842_MOESM5_ESM.zip › rawdata/size3/01_06.tif]

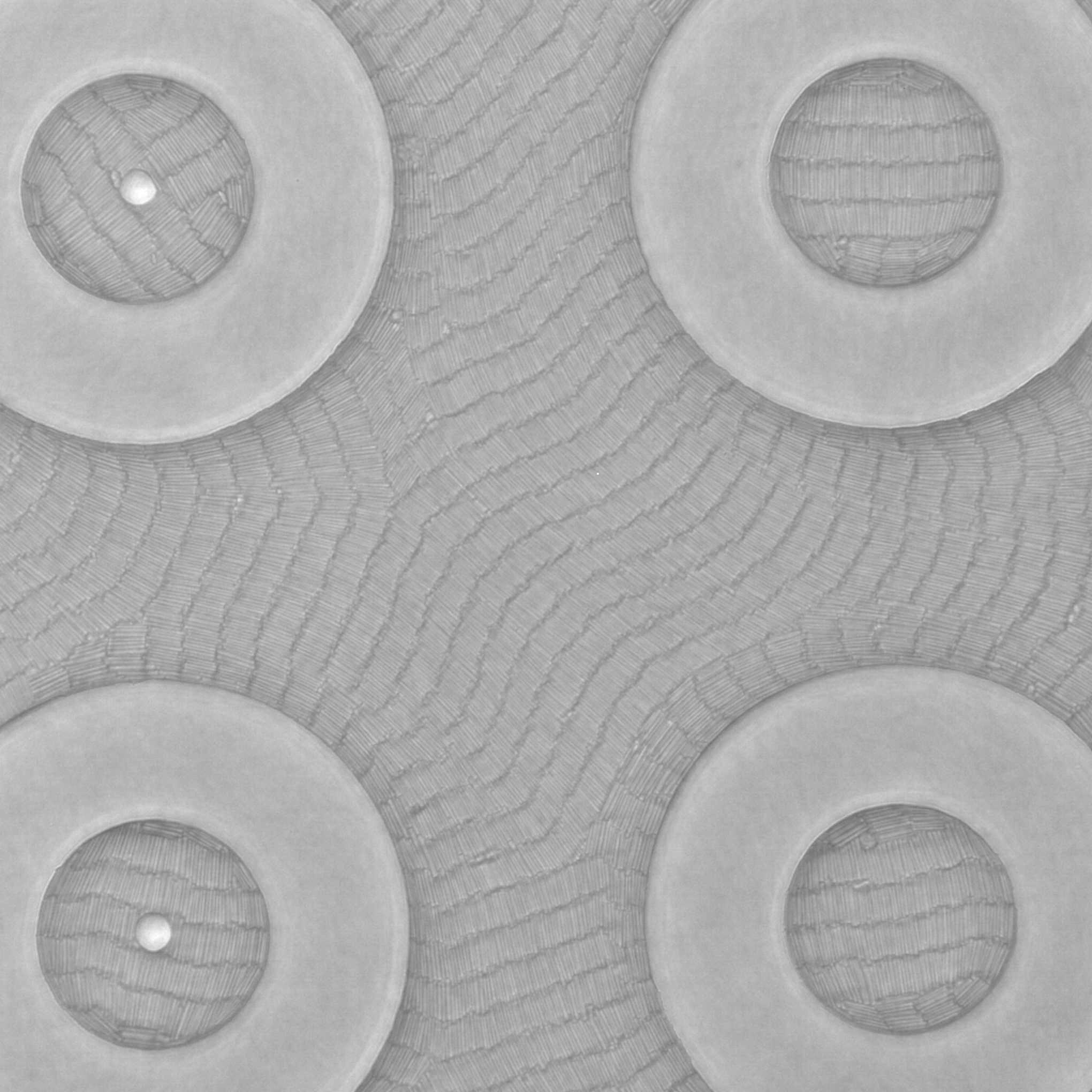

Supplement: Supplementary file 5 — Supplementary Data 2 [file 41467_2020_20842_MOESM5_ESM.zip › rawdata/size3/01_05.tif]

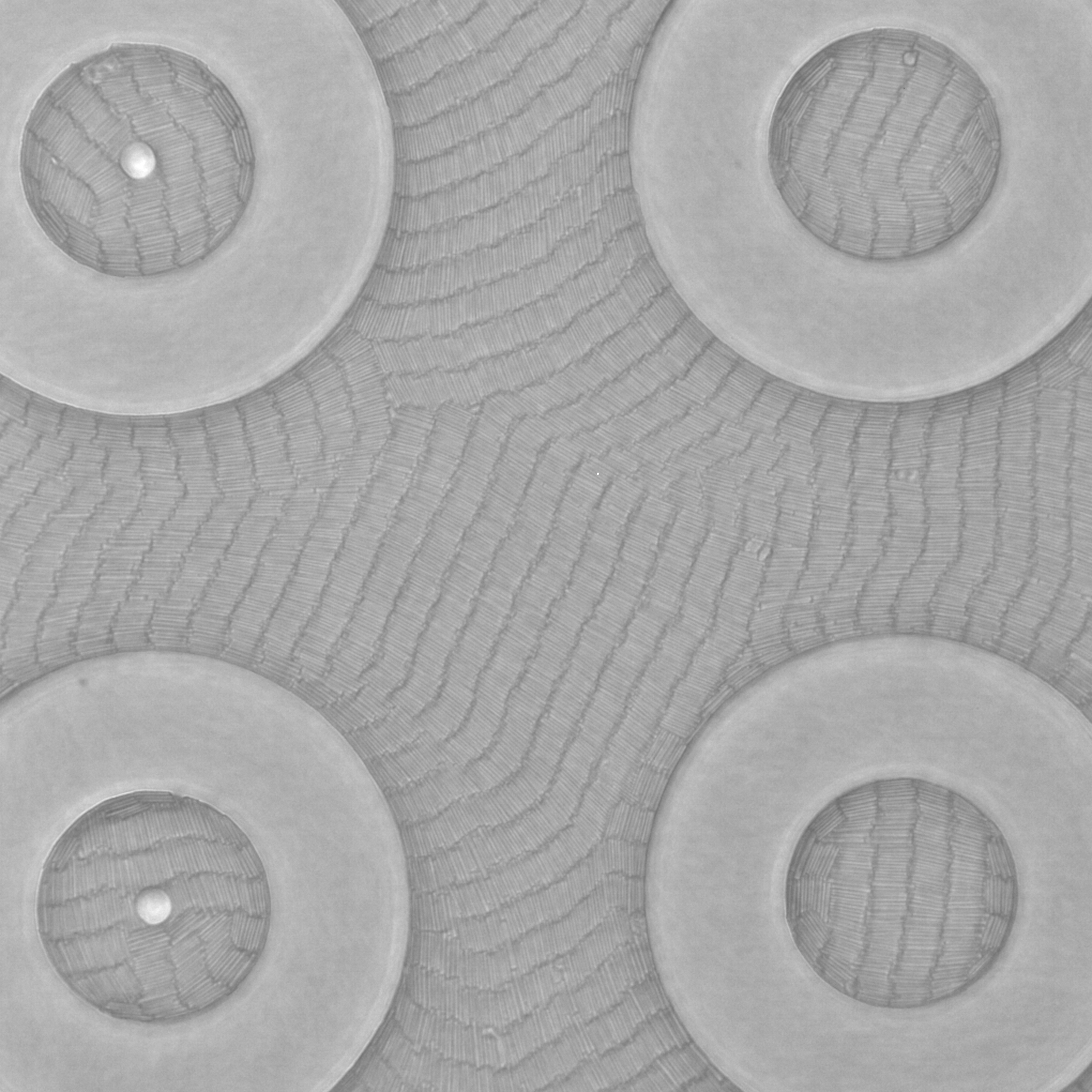

Supplement: Supplementary file 5 — Supplementary Data 2 [file 41467_2020_20842_MOESM5_ESM.zip › rawdata/size3/01_04.tif]

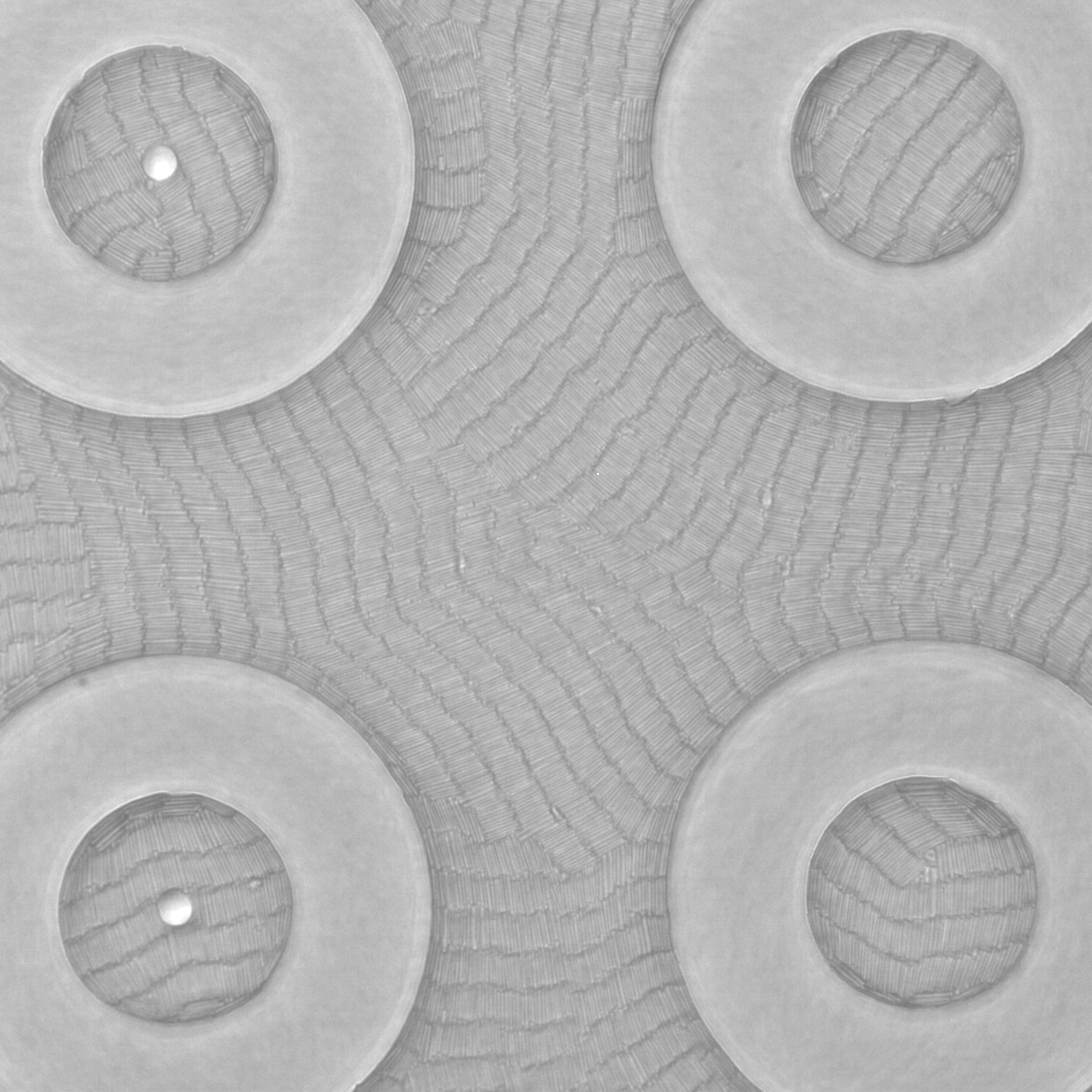

Supplement: Supplementary file 5 — Supplementary Data 2 [file 41467_2020_20842_MOESM5_ESM.zip › rawdata/size3/01_03.tif]

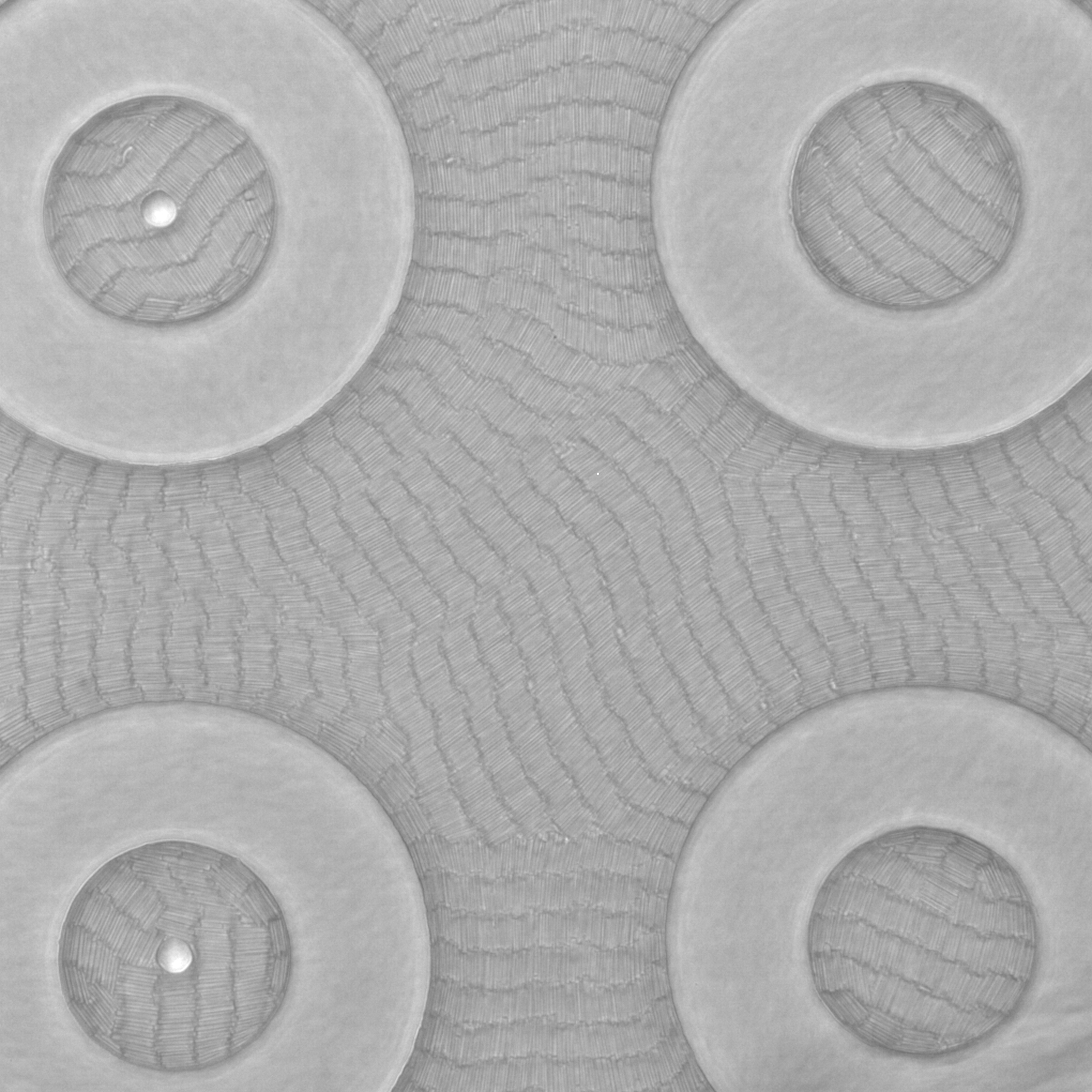

Supplement: Supplementary file 5 — Supplementary Data 2 [file 41467_2020_20842_MOESM5_ESM.zip › rawdata/size3/01_02.tif]

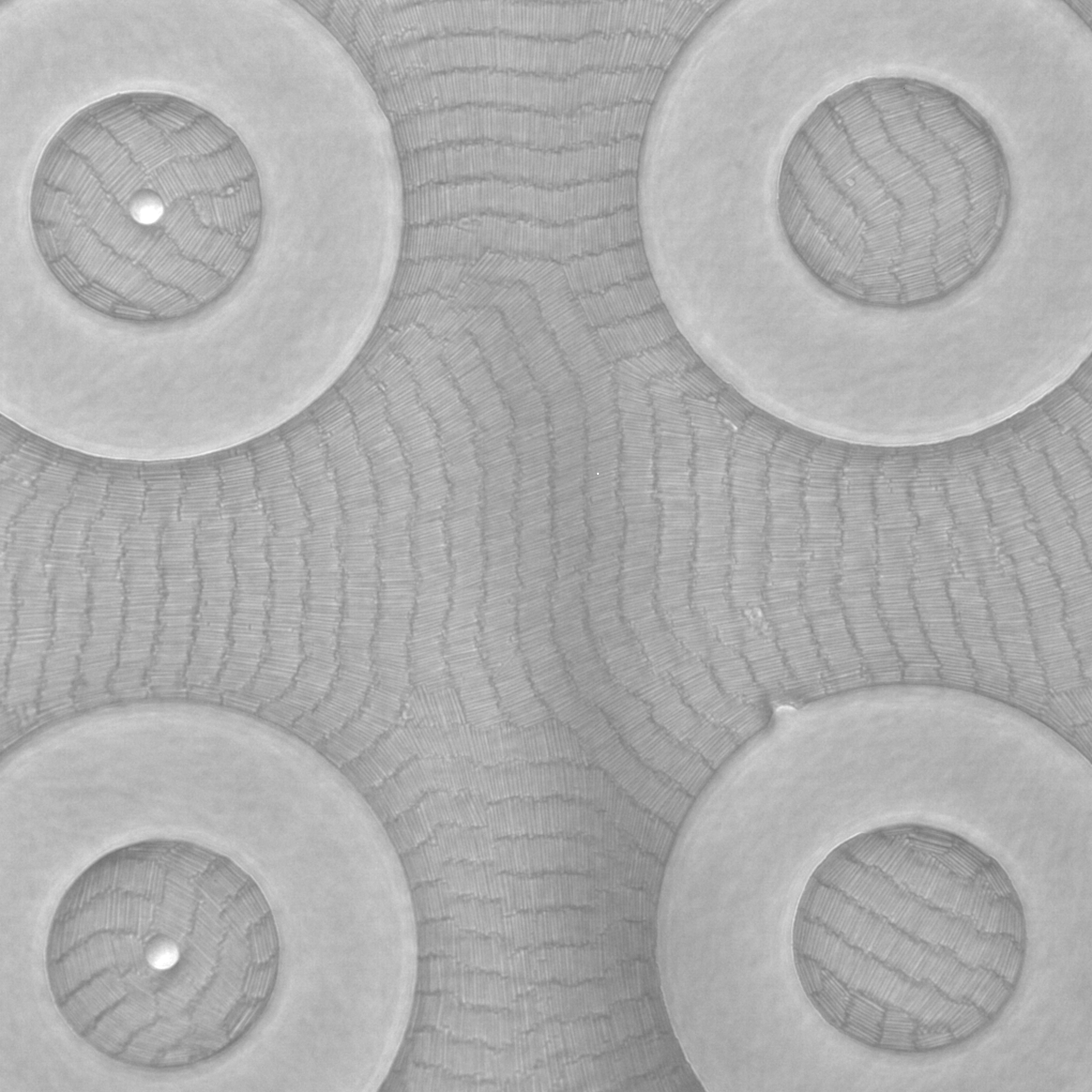

Supplement: Supplementary file 5 — Supplementary Data 2 [file 41467_2020_20842_MOESM5_ESM.zip › rawdata/size3/01_01.tif]

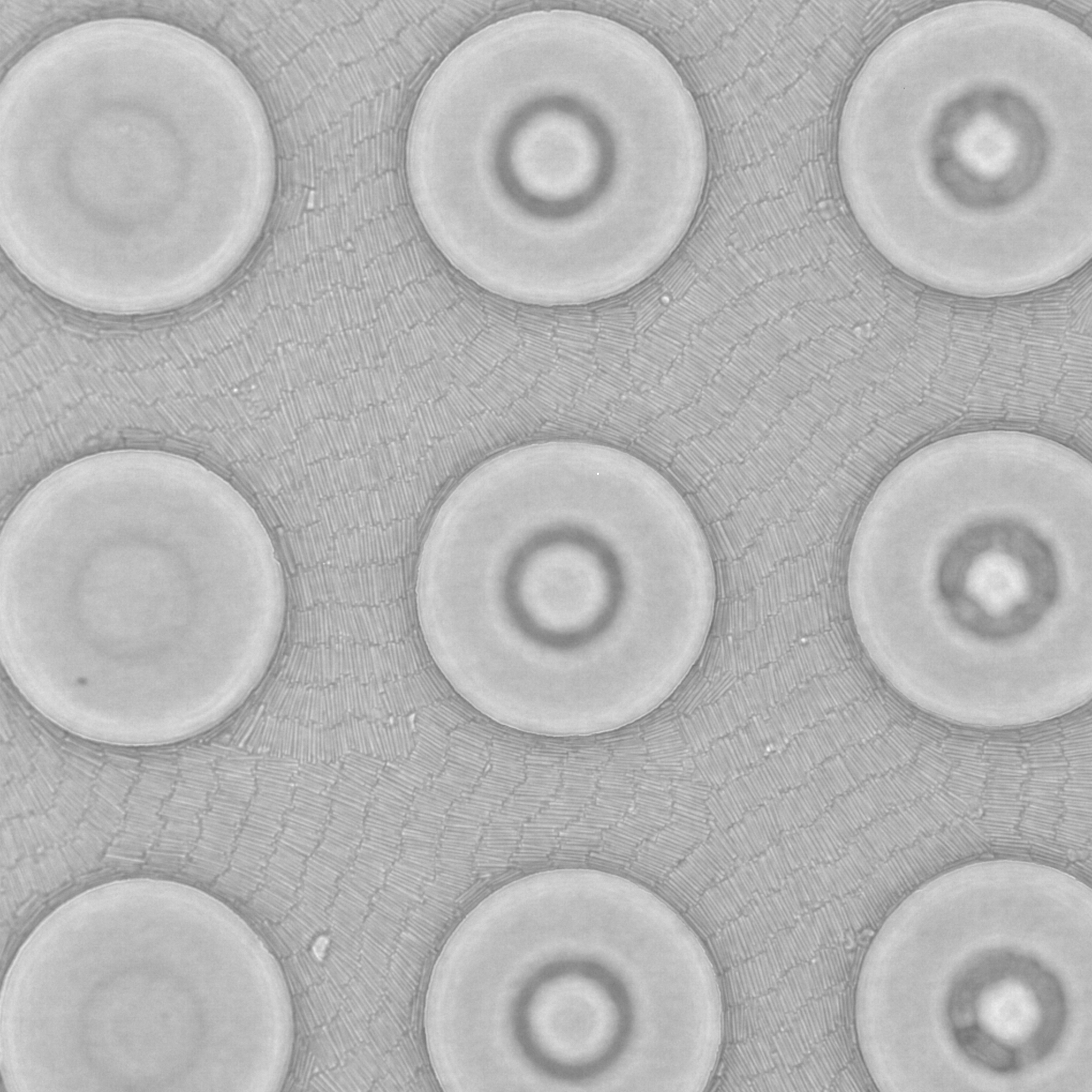

Supplement: Supplementary file 5 — Supplementary Data 2 [file 41467_2020_20842_MOESM5_ESM.zip › rawdata/size2/02_04.tif]

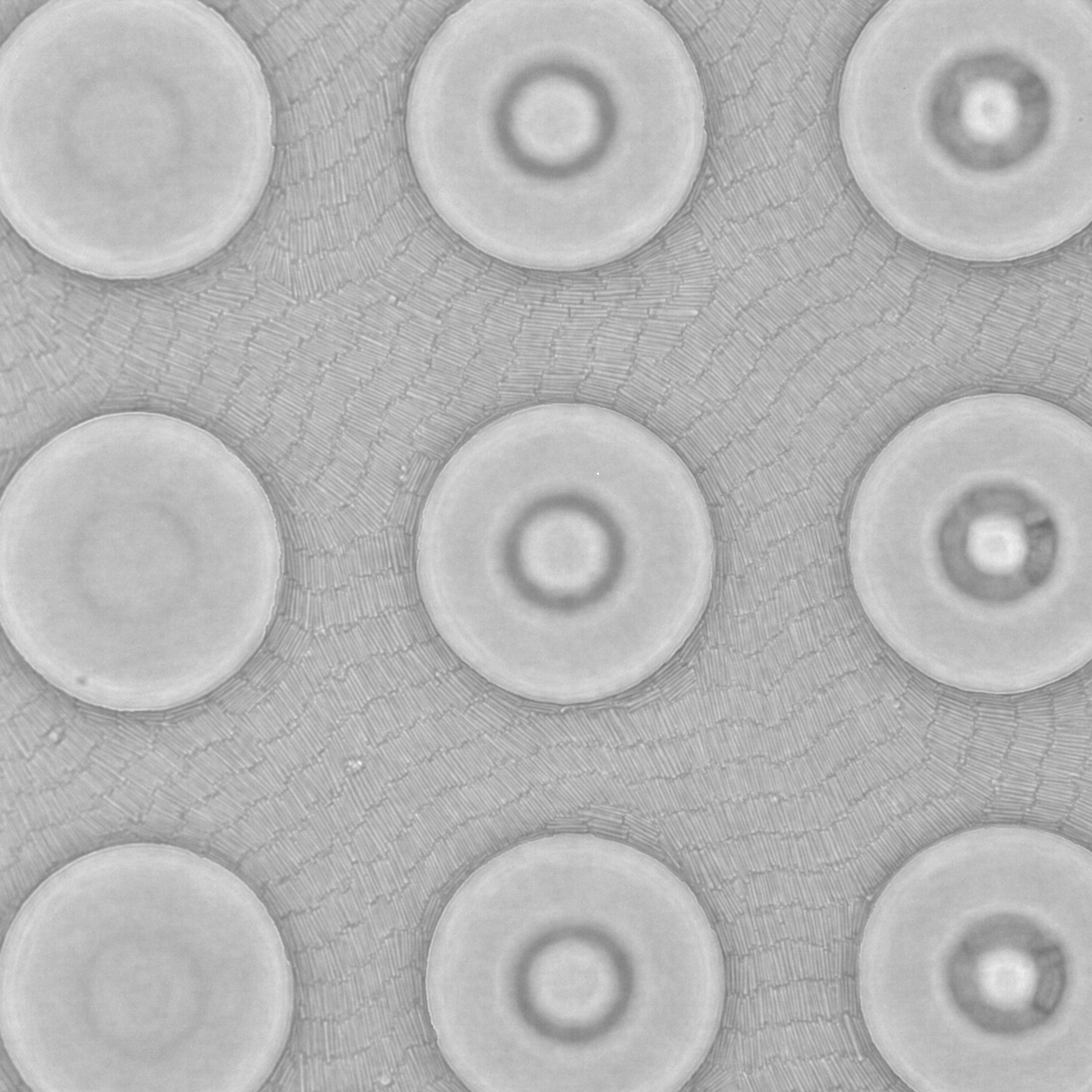

Supplement: Supplementary file 5 — Supplementary Data 2 [file 41467_2020_20842_MOESM5_ESM.zip › rawdata/size2/02_03.tif]

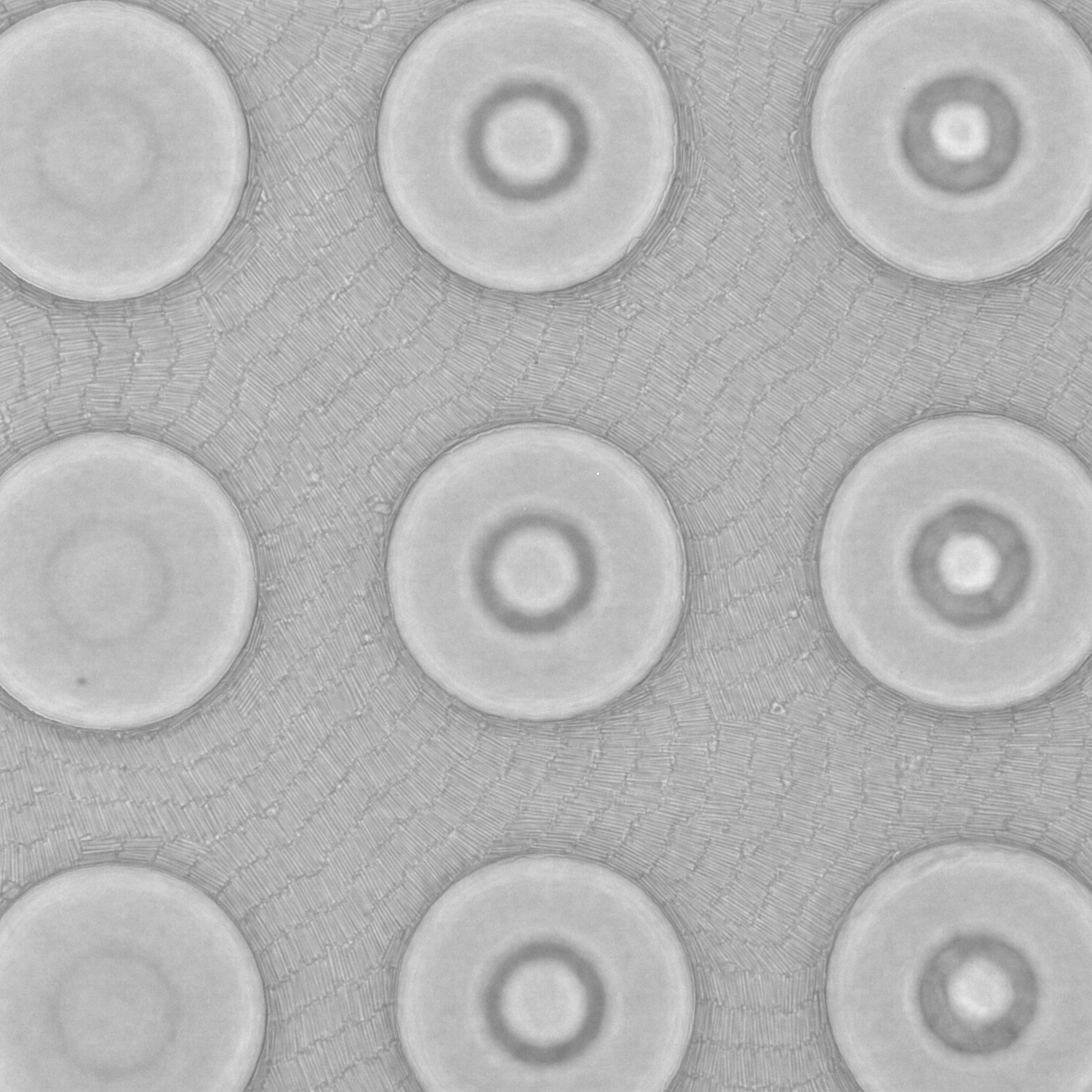

Supplement: Supplementary file 5 — Supplementary Data 2 [file 41467_2020_20842_MOESM5_ESM.zip › rawdata/size2/02_02.tif]

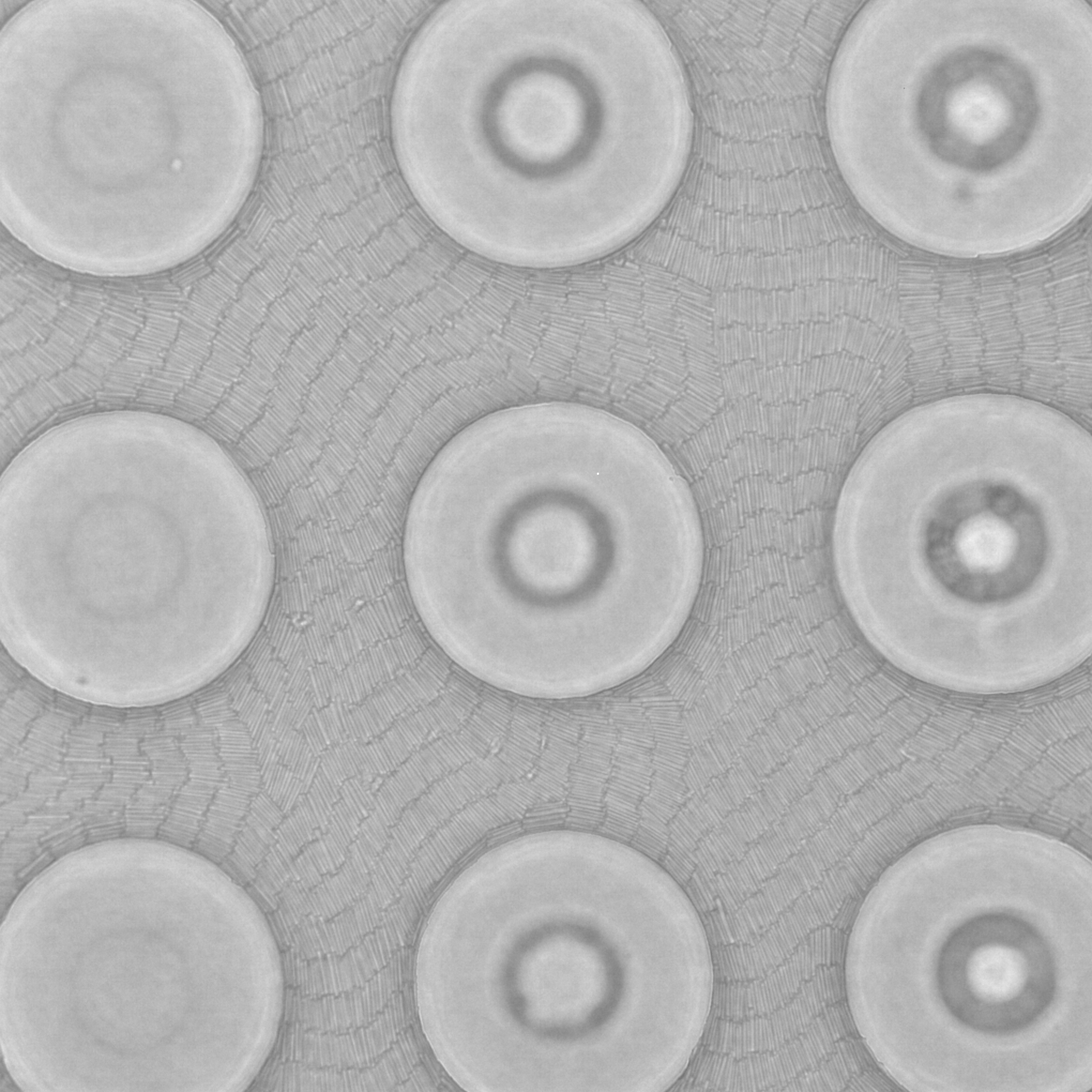

Supplement: Supplementary file 5 — Supplementary Data 2 [file 41467_2020_20842_MOESM5_ESM.zip › rawdata/size2/02_01.tif]

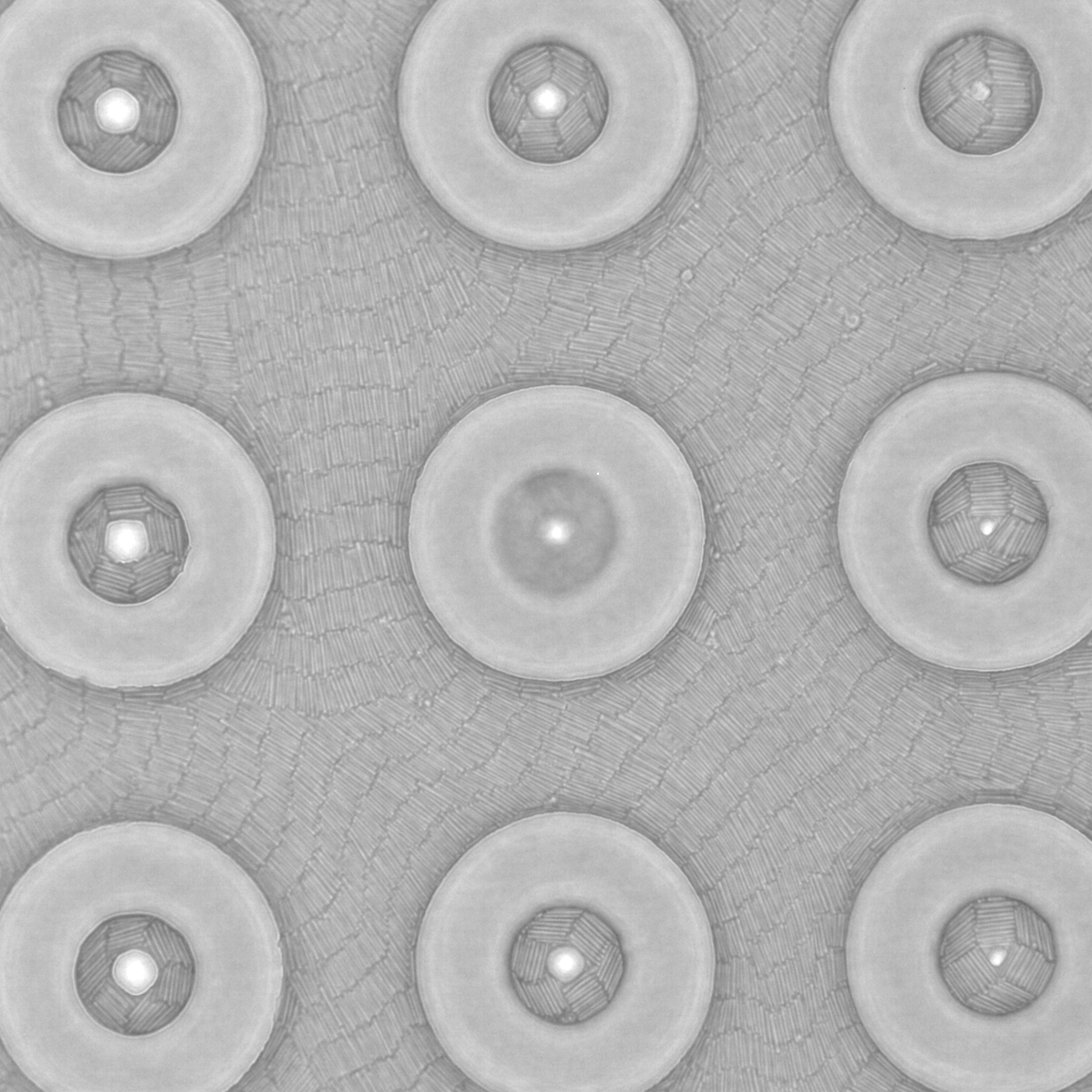

Supplement: Supplementary file 5 — Supplementary Data 2 [file 41467_2020_20842_MOESM5_ESM.zip › rawdata/size2/01_04.tif]

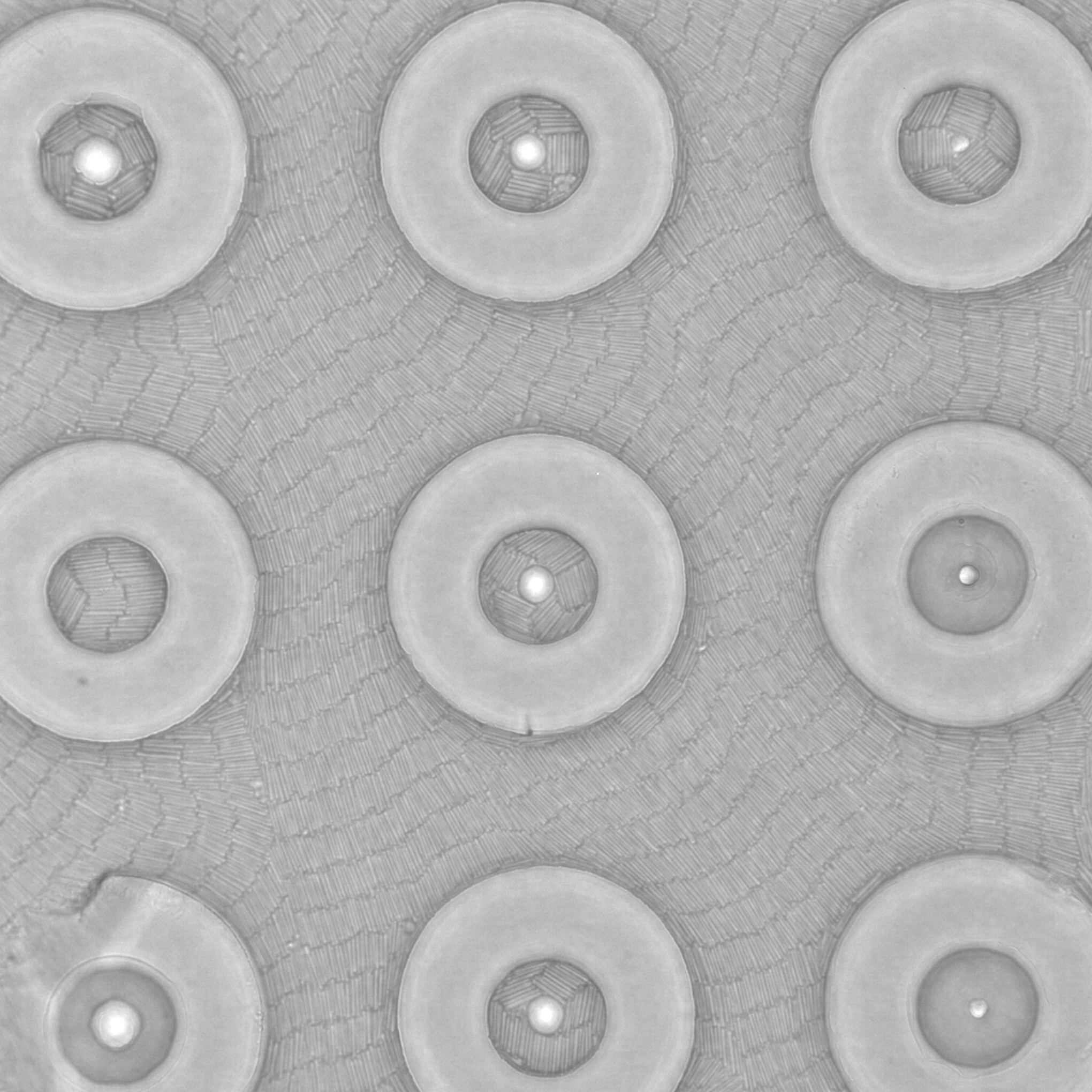

Supplement: Supplementary file 5 — Supplementary Data 2 [file 41467_2020_20842_MOESM5_ESM.zip › rawdata/size2/01_03.tif]

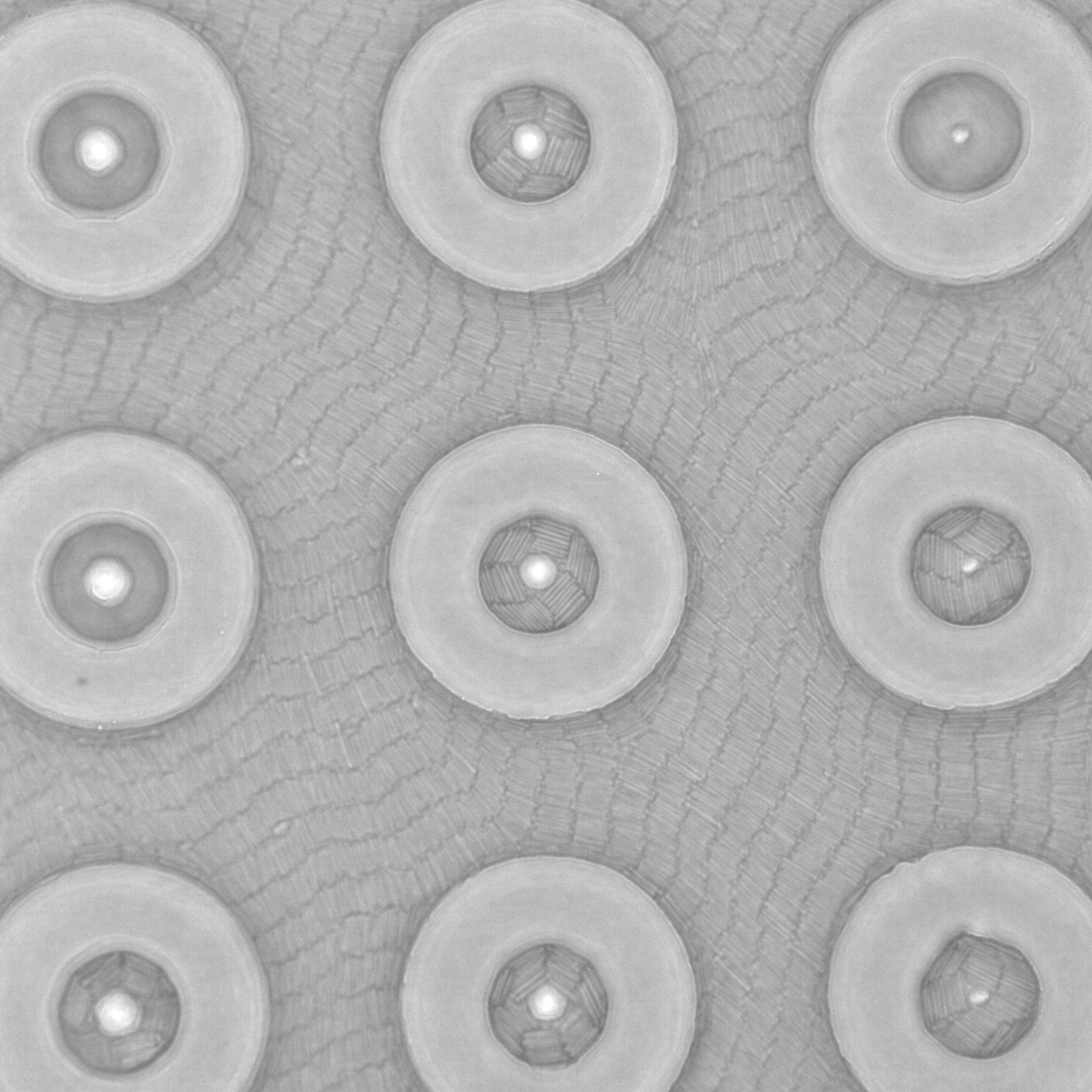

Supplement: Supplementary file 5 — Supplementary Data 2 [file 41467_2020_20842_MOESM5_ESM.zip › rawdata/size2/01_02.tif]

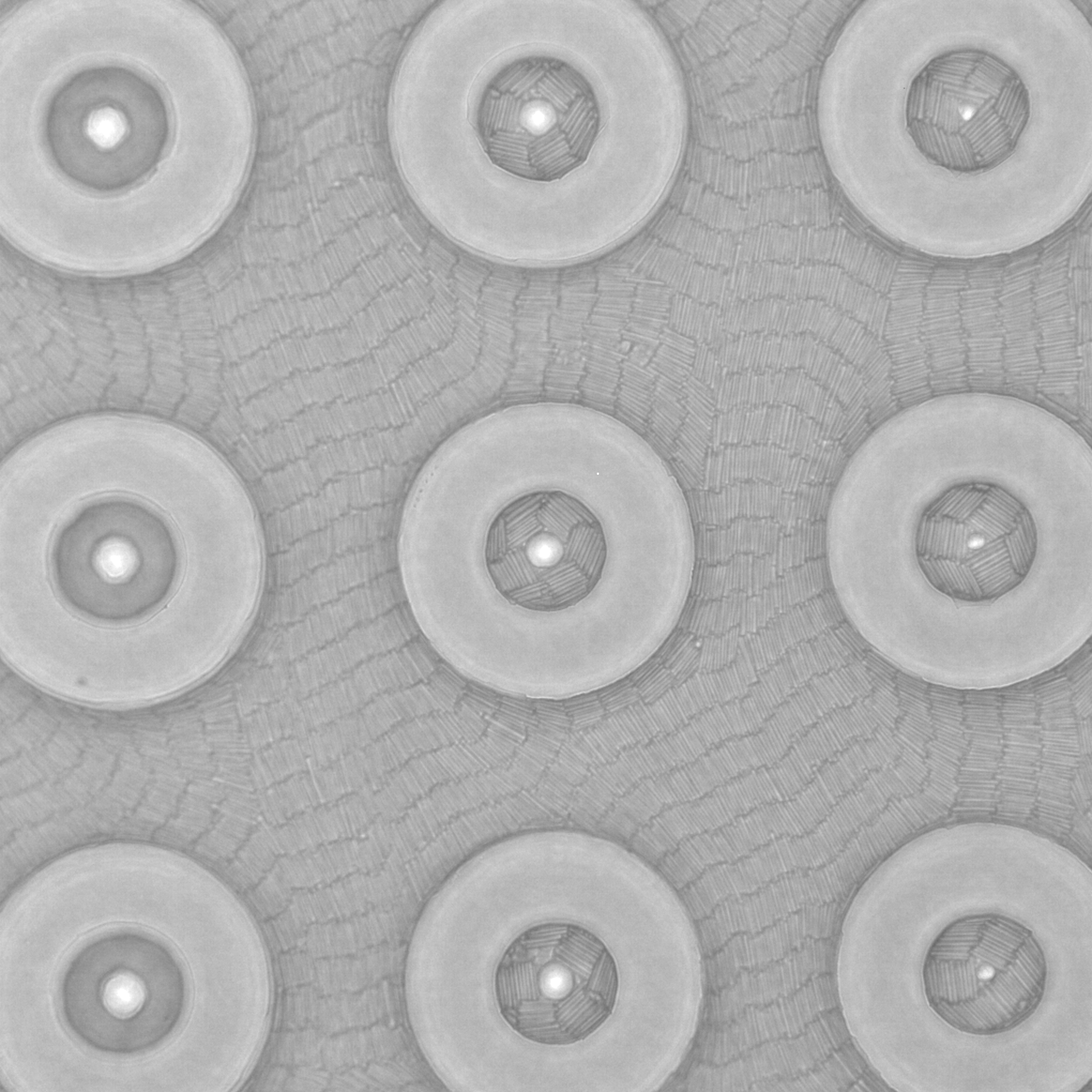

Supplement: Supplementary file 5 — Supplementary Data 2 [file 41467_2020_20842_MOESM5_ESM.zip › rawdata/size2/01_01.tif]
